# Differentially Expressed miRNA Profiles in Serum-Derived Exosomes from Cattle Infected with Lumpy Skin Disease Virus

**DOI:** 10.3390/pathogens14020176

**Published:** 2025-02-10

**Authors:** Anh Duc Truong, Ha Thi Thanh Tran, Lanh Phan, Thi Hoai Phan, Nhu Thi Chu, Thi Hao Vu, Hieu Minh Nguyen, Linh Phuong Nguyen, Chaeeun Kim, Hoang Vu Dang, Yeong Ho Hong

**Affiliations:** 1 Department of Biochemistry and Immunology, National Institute of Veterinary Research, 86 Truong Chinh, Dong Da, Hanoi 100000, Vietnam; truonganhduc84@gmail.com (A.D.T.); vet.biochem.immuno@nivr.gov.vn (H.T.T.T.); lanhphan07@gmail.com (L.P.); phanhoai271@gmail.com (T.H.P.); chunhuk58tyc@gmail.com (N.T.C.); haohao1012hua@gmail.com (T.H.V.); nmhieu.vp@gmail.com (H.M.N.); ngphuonglinhwhale2811@gmail.com (L.P.N.); 2 Department of Animal Science and Technology, Chung-Ang University, Anseong 17546, Republic of Korea; kchea77@naver.com

**Keywords:** lumpy skin disease, small RNA-seq, exosome, miRNA, pathway

## Abstract

Exosomal miRNAs from individual cells are crucial in regulating the immune response to infectious diseases. In this study, we performed small RNA sequencing (small RNA-seq) analysis to identify the expressed and associated exosomal miRNAs in the serum of cattle infected with lumpy skin disease virus (LSDV). Cattle were infected with a 10^6.5^ TCID50/mL LSDV Vietnam/HaTinh/CX01 (HT10) strain and exosomal miRNA expression in the serum of infected cattle was analyzed using small RNA sequencing (small RNA-seq). We identified 59 differentially expressed (DE) miRNAs in LSDV-infected cattle compared to uninfected controls, including 18 upregulated and 41 downregulated miRNAs. These 59 miRNAs were used to predict 7656 target genes. Gene Ontology (GO) and Kyoto Encyclopedia of Genes and Genomes (KEGG) analyses revealed that the target genes were enriched in several biological processes and pathways associated with viral replication, immune response, virus–host interactions, and signal transduction. Additionally, we identified 708 potentially novel cattle miRNAs corresponding to 710 genomic loci. The transcription levels of five miRNA genes (bta-miR-11985, bta-miR-1281, bta-miR-12034, bta-miR-let-7i, and bta-miR-17-5p) were validated using reverse transcription quantitative real-time PCR, showing consistency with the small RNA-seq results. Overall, these findings provide significant insights into the immune and protective responses during LSDV infection in cattle, offering valuable information on identifying new biomarkers and understanding the pathogenesis of LSDV.

## 1. Introduction

Lumpy skin disease (LSD) is a re-emerging transboundary viral disease that affects cattle and buffaloes, posing a significant economic threat. The World Organization for Animal Health has classified LSD as a notifiable disease [1]. The LSD virus (LSDV) is transmitted by blood-feeding vectors, such as mosquitoes and flies, facilitating its rapid spread under favorable climatic conditions [2]. The LSD epidemics in Africa, Eastern Europe, and Asia have affected millions of cattle, leading to considerable economic losses. These losses are attributed to animal mortality, reduced productivity, expenses associated with control and prevention measures, and disruptions to export markets [3]. The clinical severity of LSD ranges from subclinical to fatal, depending on the virulence of the virus strain and the susceptibility of specific cattle breeds. Typically, LSD exhibits a mortality rate of less than 10% and a morbidity range of 0% to 90% [1]. Initially identified in Zambia in 1929, LSD was historically confined to Southern and Eastern Africa [4]. The first reported outbreak outside sub-Saharan Africa occurred between 1988 and 1989 in Egypt and Israel [4]. Currently, LSD is endemic in most African countries and has spread to Southeast Europe, the Middle East, and South Asia [5]. Outbreaks have also been reported in China, India, Bangladesh, and Nepal [5,6]. In Vietnam, the first LSD outbreak was reported in October 2020 [7]. In response to the spread of LSD, vaccination programs employing live-attenuated LSDV vaccines, specifically those using the Neethling strain, have been instrumental in controlling the disease in Vietnam and Southeast Europe. 

Exosomes are formed within cells in endosomal compartments known as multivesicular bodies [8]. These multivesicular bodies fuse with the plasma membrane, releasing exosomes into the extracellular space. Exosomes typically range from 30 to 250 nanometers in diameter and play a significant role in the immune response to infectious diseases in animals [9,10]. They carry a variety of molecular contents, including proteins, lipids, RNA, DNA, and microRNA (miRNA), which significantly regulate the immune system by interacting with various immune cells such as T cells, B cells, macrophages, and natural killer (NK) cells [8,10]. Exosomes act as carriers for miRNAs, facilitating their intercellular transfer, thus modulating gene expression in immune cells [8]. Exosomal miRNAs are increasingly recognized for their crucial roles in various diseases in both humans and animals, including cancer, cardiovascular diseases, avian influenza, and neurodegenerative disorders. They can modulate the activity of immune cells, such as T cells and macrophages, thereby playing an important role in immune surveillance and inflammation [9].

Understanding the functions of exosomes is critical for developing new strategies for disease diagnosis, treatment, and prevention in veterinary medicine. However, the role of exosomes in LSDV infection remains unexplored. Given the significant role of exosomes in immunity, it is crucial to investigate the exosomal miRNAs involved in the body’s response to LSD virus infection. Studying these miRNAs is essential for both understanding their reaction to LSDV and developing effective strategies to control the disease. To address this critical gap in the literature, this study aimed to analyze the miRNA profiles, expression, and associated serum-derived exosomal miRNAs from cattle infected with LSDV.

## 2. Materials and Methods

### 2.1. LSD Virus Strain

The LSDV Vietnam/HaTinh/CX01 (HT10) strain, isolated in Ha Tinh Province, North Central Vietnam, was adapted and cultivated using the Madin-Darby bovine kidney (MDBK) cell line (KCLB#10022, Seoul, Republic of Korea). The MDBK cells were maintained in Dulbecco’s modified Eagle’s medium (DMEM, Invitrogen, CA, USA), supplemented with penicillin (100 IU/mL), streptomycin (100 mg/mL), fungizone (1 μg/mL; Sigma-Aldrich, St. Louis, MO, USA), and 5% heat-inactivated fetal bovine serum (FBS; Invitrogen). The cells were incubated at 37 °C in a humidified 5% CO_2_ incubator. For virus cultivation, the cell culture was subjected to three freeze–thaw cycles for breakdown of cells at −80 °C. The clarified supernatant was then transferred to fresh cell cultures, with multiple passages performed until characteristic cytopathogenic effects (CPEs) were observed under an inverted microscope. The appearance of CPEs and changes in viral titer during these passages demonstrated the cell culture’s susceptibility to the virus. The LSD virus titer was assessed by titration in 96-well-tissue culture plates, following a previously described method [7]. The viral titer was estimated using the Reed–Muench method and expressed in log TCID50/mL [11]. After six passages in the MDBK cell line, the stock titer of the LSD virus reached 10^7^ TCID50/mL.

### 2.2. Animal Experiment

Vietnam local yellow cattle (Bos taurus), aged between four and five months, were assigned to treatment (HT10) and control groups, with three animals per group. The cattle were housed at the National Institute of Veterinary Research facilities in Vietnam. The treatment group received the Vietnam/HaTinh/CX01 (HT10) field strain suspended in DMEM at a concentration of 10^6.5^ TCID50/mL on MDBK cells, administered via intravenous injection (2 mL per animal). The control group was injected with 2 mL of phosphate-buffered saline (PBS) per animal using the same method. The cattle were monitored daily for body temperature and clinical signs. Blood and oral swab samples were collected daily from each animal to assess the viral DNA load using real-time PCR with primers specific to the viral p32 gene, as previously described [7]. Necropsies were performed immediately if an animal died during the day, or the following morning if death occurred overnight. Tissue samples were collected during necropsy for further analysis.

### 2.3. DNA Extraction from LSDV and Real-Time PCR

The genomic DNA of LSDV was extracted using the QIAamp DNA Mini Kit (Qiagen, Hilden, Germany). LSDV DNA was detected in the samples through real-time PCR using primers specific to the viral p32 gene, as previously described [7]. Real-time PCR was performed on an Agilent AriaMx Real-Time PCR System (Agilent, Santa Clara, CA, USA) following an established protocol. DNA from LSDV-positive samples served as the positive amplification control, and nuclease-free sterile water was used as the negative amplification control.

### 2.4. Exosome Purification and Characterization

Exosomes were purified from serum using the Total Exosome Isolation Reagent (Invitrogen, Carlsbad, CA, USA) according to the manufacturer’s protocol. The particle size of the purified exosomes was measured using a nanoparticle analyzer (HORIBA, SZ-100, Kyoto, Japan). Further characterization was performed using Western blotting on pooled exosome samples, targeting three exosomal markers: CD9 (#13174; Cell Signaling Technology, Danvers, MA, USA), CD63 (sc-5275; Santa Cruz Biotechnology, Heidelberg, Germany), and CD81 (#56039; Cell Signaling Technology, Danvers, MA, USA), following previously described methods [12]. 

### 2.5. Exosomal RNA Isolation and Small RNA Sequencing

Exosomal RNA was isolated separately using the miRNeasy Serum/Plasma Kit (Qiagen, Hilden, Germany) in accordance with the manufacturer’s instructions. For both the control and infected groups, three samples were selected at 21 days post-infection (dpi) based on clinical assessments that indicated the presence of clinical, digestive, or respiratory symptoms in all LSDV-infected cattle at this time point. These samples were utilized for library construction and small RNA sequencing (RNA-seq). Library construction for small RNA-seq was conducted using the SMARTer smRNA-Seq Kit for Illumina (TAKARA Bio Inc., Otsu, Shiga, Japan), following the manufacturer’s protocol. Small RNA-seq was performed on the Illumina platform (Illumina Inc., San Diego, CA, USA) by Macrogen (Seoul, Republic of Korea).

### 2.6. Sequencing Data Analysis

The raw sequence data were initially processed using FastQC v0.11.7 (http://www.bioinformatics.babraham.ac.uk/projects/fastqc/; accessed on: 20 March 2024) to filter and perform quality checks, ensuring data integrity prior to analysis. Subsequently, Cutadapt v4.4 (https://cutadapt.readthedocs.org/en/stable/; accessed on: 20 March 2024) was employed to identify and remove adapter sequences, primers, poly-A tails, and other unwanted sequences from high-throughput sequencing reads. The final processed reads were aligned against the cattle reference genome (ARS-UCD1.2, RefSeq: GCF_002263795.3) using Bowtie 1.1.2 (https://www.mdc-berlin.de/content/mirdeep2-documentation; accessed on: 20 March 2024), miRBase v22.1 (http://www.mirbase.org/; accessed on: 20 March 2024), and RNAcentral release 22.0 (https://rnacentral.org/; accessed on: 20 March 2024). The miRDeep2 v2.0.0.8 program (https://www.mdc-berlin.de/content/mirdeep2-documentation; accessed on: 20 March 2024) was used to identify and predict the known and novel miRNAs in the samples. Differentially expressed (DE) miRNAs were statistically analyzed using fold changes and exact comparisons with edgeR (empirical analysis of digital gene expression data in R). The criteria for significant differential expression included a |fold-change| ≥ 2 and an exactTest raw *p*-value < 0.05. The target genes of DE miRNAs were predicted using the TargetScan (https://www.targetscan.org/vert_80/; accessed on: 20 May 2024) database. Target genes were predicted based on two conditions: (i) conserved sites as 8-mer or 7-mer-m8 and (ii) an aggregate PCT ≥ 0.75 or a cumulative weighted context++ score or total context++ score ≥ −0.1, following previously described methods [13]. Functional enrichment analysis of the target genes was conducted using the Gene Ontology Resource (http://geneontology.org/) for Gene Ontology (GO) analysis. Additionally, Kyoto Encyclopedia of Genes and Genomes (KEGG) pathway enrichment analysis was performed using DAVID 2021 (Dec. 2021) (https://david.ncifcrf.gov/summary.jsp#; accessed on: 20 March 2024). 

### 2.7. Reverse Transcription Quantitative Real-Time PCR (RT-qPCR)

The method for miRNA expression analysis via RT-qPCR was previously described in [12]. The primers utilized in this study are listed in Table 1. Briefly, individual miRNA-specific forward primers were designed, and a universal reverse primer was included in the Mir-X miRNA First-Strand Synthesis and TB Green kit (Takara Bio Inc., Kusatsu, Shiga, Japan). The Mir-X miRNA First-Strand Synthesis kit (TaKaRa Bio Inc.) was utilized for cDNA synthesis, and miRNA expression was determined using the Mir-X miRNA qRT-PCR TB Green kit (TaKaRa Bio Inc.), following the manufacturer’s instructions. The U6 gene of cattle was used as the control. miRNA expression levels were calculated using the 2^−ΔΔCt^ method [14]. RT-qPCR experiments were conducted in triplicate to ensure reliability.

### 2.8. Statistical Analysis

Statistical analyses were performed using SPSS software (version 25.0; IBM, Chicago, IL, USA). Data are presented as mean ± standard error of the mean (SEM). Group comparisons were conducted using a two-tailed Student’s *t*-test, with statistical significance defined as *p* < 0.05.

## 3. Results

### 3.1. Exosome Isolation and Characterization

**Figure 1:**
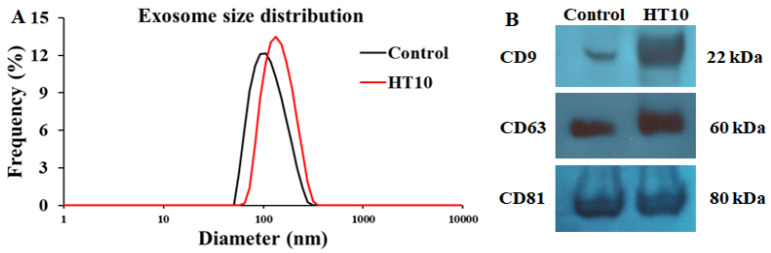
Characterization of bovine exosomes isolated from serum in the control and treatment groups. (**A**) Exosome size distribution: red and blue represent serum-derived exosomes from the control and LSDV-infected groups, respectively. (**B**) Western blot analysis: exosomes from both the control and infected groups were analyzed via Western blotting using exosomal markers (CD9, CD63, and CD81).

Exosomes isolated from the serum of cattle infected with LSDV were characterized using nanoparticle tracking analysis (NTA) and Western blotting, as depicted in Figure 1. The predominant particle diameters averaged approximately 98.5 nm in the control group and 126.1 nm in the LSDV-infected group (HT10) (Figure 1A). Particles within the size range of 20 nm to 200 nm accounted for 95.28% of the total in the control group and 86.47% of the total in the HT10 group. The presence of exosomes was further validated through the Western blot analysis of exosomal membrane markers CD63, CD9, and CD81 (Figure 1B). Purified serum-derived exosomes from both the control and LSDV-infected groups were utilized in subsequent experiments.

### 3.2. Analysis of Exosomal miRNA Expression Using Small RNA Sequencing

The total raw read count from sequencing across six libraries was 232,424,695, with an average of approximately 38,737,449.17 reads per sample. After removing linker reads, sequences containing N and poly A/T structures, length-anomalous reads, low-quality reads, and reads exceeding 35 nt or shorter than 17 nt, approximately 1.58% of the reads (ranging from 0.36% to 5.38%), were successfully aligned to the cattle reference genome (ARS-UCD 2.0) (Table 2). A total of 302 known and 703 novel miRNAs were identified, meeting the criteria of at least one transcript per million clean tags and detection in a minimum of six libraries.

**Figure 2:**
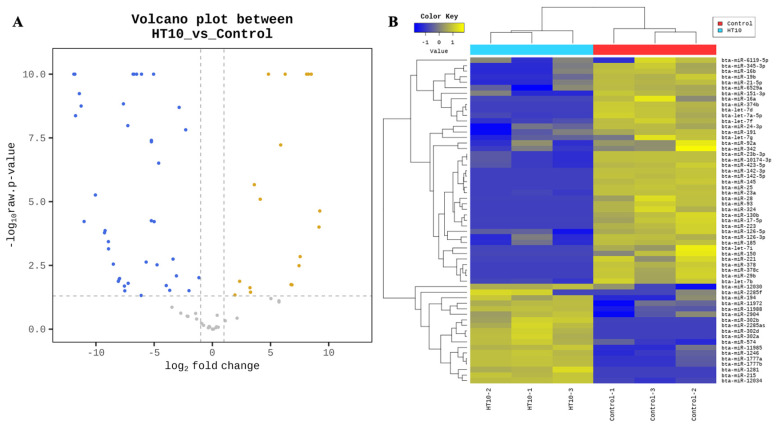
Volcano plot and heatmap illustrating miRNA expression levels in control and LSDV-infected groups. (**A**) Volcano plot: Log2 fold changes and *p*-values were computed by comparing the two groups and visualized in the plot. The X-axis represents the log2 fold change, and the Y-axis depicts the negative logarithm (base 10) of the *p*-value. Yellow dots indicate a fold change ≥ 2 and a raw *p*-value < 0.05. Blue dots indicate a fold change ≤ −2 and a raw *p*-value < 0.05. (**B**) Heatmap: hierarchical clustering analysis was conducted using the Euclidean method and complete linkage. The heatmap represents the similarity of expression levels (normalized values) of 59 mature miRNAs across six samples. The control group is denoted by a red box, and the infected group is indicated by a blue box.

Differential expression analysis of miRNAs was conducted using fold change and exactTest values in edgeR. miRNAs with a |fold change| ≥ 2 and an exact raw *p*-value < 0.05 were considered significantly differentially expressed. As illustrated in Figure 2A, a volcano plot depicts the expression of miRNAs in cattle infected with LSDV compared to the control group. Among the 302 detected miRNAs (Supplementary Table S1), 59 miRNAs were identified as significantly differentially expressed, including 18 upregulated and 41 downregulated miRNAs in LSDV-infected cattle compared to the control (Table 3). The heatmap illustrated in Figure 2B highlights the expression patterns of these DE miRNAs between LSDV-infected and control groups. Notably, five miRNAs (bta-miR-302d, bta-miR-302a, bta-miR-302b, bta-miR-1281, and bta-miR-2285as) were exclusively expressed in the LSDV-infected group, whereas 15 miRNAs (bta-miR-21-5p, bta-let-7i, bta-miR-221, bta-miR-28, bta-miR-145, bta-miR-16a, bta-miR-130b, bta-miR-29b, bta-miR-378c, bta-miR-374b, bta-miR-378, bta-miR-150, bta-miR-25, bta-miR-142-5p, and bta-miR-142-3p) were exclusively expressed in the control group (Table 3 and Supplementary Table S1). 

### 3.3. Gene Ontology Enrichment Analyses of Target Genes

The target genes of the DE miRNAs were predicted using the TargeScan Human tool (https://www.targetscan.org/vert_80/; accessed on: 20 May 2024). Subsequently, GO enrichment analysis was performed to infer the biological functions of the DE miRNAs through an examination of the functions of these target genes. Among the 59 DE miRNAs, 50 miRNAs were successfully used to predict 7656 non-repeating target genes. Bta-miR-2904 had the highest number of target genes (2904 genes), whereas bta-miR-302d was the most highly upregulated miRNA in cattle infected with LSDV, without the target gene. Conversely, in the control group, bta-miR-142-3p was the most highly downregulated miRNA in cattle infected with LSDV, with 872 predicted target genes.

**Figure 3:**
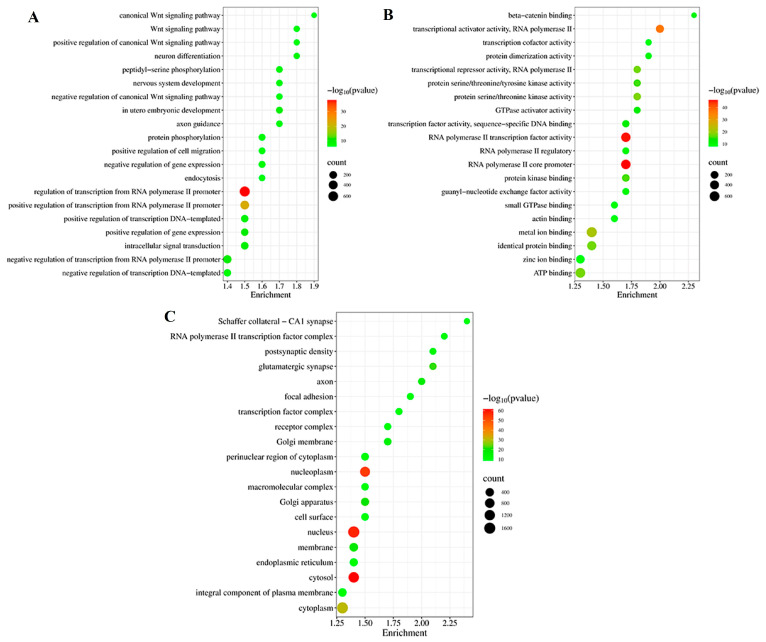
Gene Ontology (GO) enrichment analysis of target genes from 59 DE miRNAs. GO analysis was used for target genes across three categories: (**A**) biological processes, (**B**) cellular components, and (**C**) molecular functions.

The target genes of DE miRNAs were significantly enriched (*p* < 0.05, number of genes > 5) in 807 GO terms (450 biological processes, 155 cellular components, and 202 molecular functions) (Supplementary Table S2). Among biological processes, the most enriched GO terms were the regulation of transcription from RNA polymerase II promoter (680 gene counts), the positive regulation of transcription from RNA polymerase II promoter (368 gene counts), and the positive regulation of gene expression (162 gene counts) (Figure 3A and Supplementary Table S2A). For cellular components, the most enriched GO terms were the cytosol (1269 gene counts), nucleus (1650 gene counts), and nucleoplasm (970 gene counts) (Figure 3B and Supplementary Table S2B). For molecular functions, the most enriched GO terms were RNA polymerase II core promoter (524 gene counts), RNA polymerase II transcription factor activity (495 gene counts), and transcriptional activator activity, RNA polymerase II (247 gene counts) (Figure 3C and Supplementary Table S2C).

### 3.4. Pathway Enrichment Analyses of Target Genes

**Figure 4:**
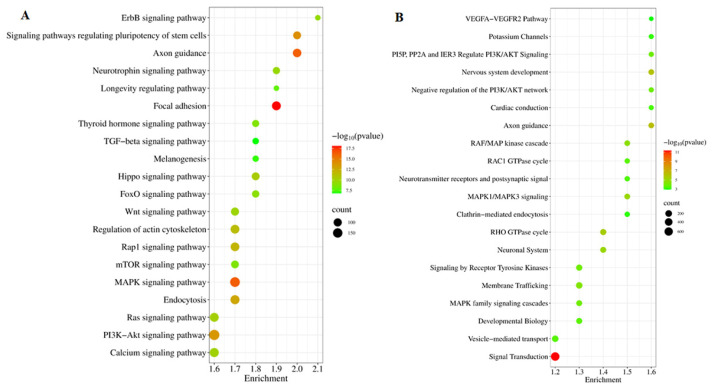
Pathway analysis of differentially expressed (DE) miRNAs. (**A**) Top 20 enriched KEGG pathways and (**B**) Reactome pathways for target genes of the 59 known DE miRNAs. Dot size reflects the gene count, and the gene ratio indicates the proportion of target genes associated with a specific KEGG/Reactome term compared to the total number of genes in that term.

The target genes of DE miRNAs, totaling 7656 genes, were subjected to pathway analysis using the KEGG database through the DAVID Bioinformatics Resources version 2021, with significance set at *p* < 0.05. Additionally, the Reactome database was utilized to assess their involvement in pathways related to cattle biology. The analysis revealed significant enrichment in 95 KEGG pathways for the target genes of DE miRNAs (Supplementary Table S3A), with the top 20 pathways illustrated in Figure 4A. These DE miRNAs exhibited significant associations with various biological processes, including immune response, signal transduction pathways (such as the signaling pathways regulating pluripotency of stem cells, MAPK signaling pathway, PI3K-Akt signaling pathway, Wnt signaling pathway, cAMP signaling pathway, and TGF-beta signaling pathway), metabolism-related pathways (including the calcium signaling pathway, sphingolipid metabolism, inositol phosphate metabolism, protein digestion, and glucagon signaling pathway), as well as processes related to pathogenesis, virus–host interaction, neurotrophin signaling, apoptosis, and autophagy (Supplementary Table S3A). These findings show that the identified DE miRNAs and their target genes play significant roles in regulating the bovine immune response to LSDV infection.

To further elucidate the immune response of cattle to LSDV infection, KEGG-enriched pathways were analyzed using the Reactome pathway. The results revealed the involvement of 100 Reactome pathways linked to the target genes of DE miRNAs in serum-derived exosomes from LSDV-infected cattle (Supplementary Table S3B). The top 20 Reactome pathways are depicted in Figure 4B. These DE miRNAs were primarily associated with signal transduction, immune response, and metabolic pathways (Figure 4B and Supplementary Table S3B).

### 3.5. Novel miRNA Discovery

Novel miRNAs were predicted using the RNAfold algorithm, based on mature, star, and loop sequences derived through miRDeep2 analysis. This analysis identified 708 potentially novel bovine miRNAs, corresponding to 710 genomic loci (Supplementary Table S4). The total read counts for these novel miRNAs were significantly higher on average compared to known cattle miRNAs cataloged in miRBase. For instance, only 40 unique sequences had a total read count below 10, whereas 123 of the newly identified miRNAs exhibited read counts exceeding 100. Given that miRNAs recognize target mRNAs via a conserved seed region comprising 6–8 nucleotides at the 5’ end, the sequences of this seed region of the potentially novel miRNAs were compared with seed regions of 25,141 known miRNAs in miRBase. Among the 708 novel miRNAs, 650 are shared identical seed sequences with known mature miRNAs, suggesting functional relevance for many of the miRNAs predicted through a miRDeep2 analysis of non-annotated reads. Additionally, the unique sequences of the predicted potentially novel miRNA were aligned with miRBase using BLAST. The results revealed 10 unique non-annotated sequences bearing significant homology with 10 known cattle miRNAs: bta-miR-183, bta-miR-10175-5p, bta-miR-12041, bta-miR-12048, bta-miR-12063, bta-miR-2332, bta-miR-2397-5p, bta-miR-2458, bta-miR-29b, and bta-miR-6523a (Supplementary Table S4). However, these sequences were not included in the differential expression analyses of exosomes from the control and LSDV-infected cattle. This exclusion supports the confidence that this study has identified previously undiscovered bovine miRNAs.

### 3.6. RT-qPCR Analysis of DE miRNAs

**Figure 5:**
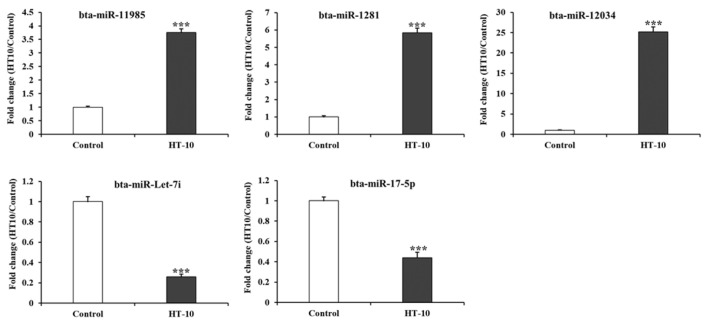
Validation of DE miRNAs using quantitative real-time polymerase chain reaction (RT-qPCR). Bar graphs depict the average fold changes observed across individual samples. miRNA expression levels were normalized to bovine U6 expression. Error bars represent the standard error of the mean (*** *p* < 0.001). Experiments were conducted in triplicate using individual samples.

To validate the findings from small RNA-seq, RT-qPCR was conducted on five miRNAs: bta-miR-11985, bta-miR-1281, bta-miR-12034, bta-miR-Let-7i, and bta-miR-17-5p. These miRNAs were selected based on their fold change (|fold change| ≥ 2) and statistical significance (*p*-value < 0.05) between the control and LSDV-infected cattle. Selection criteria also included their read counts, association with immune-related genes, pathway analysis results, and known roles in immune system function. The RT-qPCR results revealed that the expression levels of bta-miR-11985, bta-miR-1281, bta-miR-12034, bta-miR-Let-7i, and bta-miR-17-5p in the serum of LSDV-infected cattle were 3.75-, 5.84-, 25.2-, 0.26-, and 0.44-fold higher compared to the control group, respectively (Figure 5). These findings suggest that the expression patterns of the four miRNAs strongly correlate with the outcomes of the small RNA-seq analysis.

## 4. Discussion

This study used small RNA sequencing to analyze miRNA expression profiles in serum-derived exosomes from LSDV-infected cattle at 21 dpi. A total of 59 DE miRNAs were identified in these exosomes. Based on these DE miRNAs, we predicted 7656 target genes and performed Gene Ontology (GO) and Kyoto Encyclopedia of Genes and Genomes (KEGG) pathway analyses, comparing LSDV-infected cattle with control groups. Furthermore, potentially novel bovine miRNAs were identified in LSDV-infected cattle. Previous research has suggested that miRNA downregulation may be associated with the upregulation of immune genes, immune-related genes, and cytokines, leading to a significant decrease in leukocytes, hemorrhage, and animal mortality due to virulent pathogens [15]. Several studies have shown that the downregulation of miRNAs, which are key regulators of immune or immune-related genes, plays a crucial role in the immune response during infections with virulent pathogens, such as ASFV and PRRS in pigs, HPAI in chickens, and Staphylococcus and Mycobacterium in cattle [12,16,17]. These miRNAs are involved in various pathways, including the B or T-cell receptor signaling pathway, MAPK signaling pathway, JAK-STAT signaling pathway, Toll-like receptor pathway, NOD-like/RIG-I-like receptor signaling pathways, Rap1 signaling pathway, cytokine–cytokine receptor interactions, TGF-β signaling pathway, PI3K-Akt signaling pathway, mTOR signaling pathway, and Wnt signaling pathway. Our results indicate that the downregulated miRNAs in serum-derived exosomes from LSDV-infected cattle were primarily involved in the immune response to LSDV. These miRNAs were associated with several key pathways, including the MAPK signaling pathway, Rap1 signaling pathway, PI3K-Akt signaling pathway, mTOR signaling pathway, and Wnt signaling pathway. Therefore, our findings demonstrate that DE exosomal miRNAs may play a crucial role in regulating the immune response in cattle infected with virulent LSDV strains isolated from the field.

Previous studies have demonstrated that the MAPK, Rap1, PI3K-Akt, and mTOR signaling pathways play an essential role in regulating pathogen entry through biological processes in cattle infected with various pathogens, such as herpesvirus type 1 [18], type 4 infection [19], mycoplasma infection [20], hepatitis E virus [21], bovine viral diarrhea virus-infected [22], Fasciola hepatica infection [23], and parainfluenza virus [24]. The MAPK cascade gene superfamily is essential in responding to various cellular signals, transducing messages from the cell membrane to the nucleus to regulate cell proliferation, differentiation, survival, immune responses, and apoptosis. MAPK pathways are activated by several viral infections, including LSDV, coxsackievirus B3, bovine herpesvirus, bovine parainfluenza, and enterovirus. The inhibition of MAPK has been shown to reduce viral replication and transmission, highlighting its crucial role in the LSDV replication cycle [18,25,26]. The PI3K/Akt pathway is similarly critical, promoting transmission and replication in viruses, such as bovine herpesvirus, parainfluenza, enterovirus, and Ebola. Notably, the activation of the PI3K/Akt pathway during the later stages of Influenza A infection is essential for efficient replication [18,25,26]. The Rap1 signaling pathway facilitates cell proliferation, migration, and metastasis, interacting with MAPK, PI3K/Akt, and other pathways. However, a comprehensive understanding of its roles in animals is lacking [27]. The mTOR pathway, critical for cell growth and immunity, is activated by infections such as HBV, MERS-CoV, SARS-CoV-2, and Mycoplasma bovis. Increased mTOR activity has been shown to reduce MERS-CoV infection by up to 60% [28,29,30]. Our results suggest that miRNAs in serum-derived exosomes may regulate LSDV replication through immune or signaling pathways, including the MAPK/Rap1/PI3K-Akt/mTOR signaling pathways.

Furthermore, 708 potentially novel bovine miRNAs, corresponding to 710 genomic loci, were identified in cattle, with 123 novel miRNAs showing high read counts in samples (greater than 100). Several potentially novel miRNAs were identified, associated with responses to stress, cellular processes, binding, membrane function, and ATP activity, and are explicitly involved in the immune response. Previous studies have shown that several of the 59 DE miRNAs in this study are linked to viral infections and immune system regulation. For instance, the overexpression of bta-miR-223 can mitigate inflammation-mediated damage by targeting CBLB to inhibit the PI3K/AKT/NF-κB pathway, thereby suppressing cytokine expression [31,32]. Additionally, bta-miR-223 plays a role in the immune response to mastitis in dairy cows by targeting the HMGB1 gene [33]. Conversely, bta-miR-16a is upregulated in the mammary tissues and blood neutrophils of cows infected with mastitis. The bta-miR-16a cluster contributes to the defense against mastitis by targeting the CD163 gene and regulating the expression of both anti-inflammatory and pro-inflammatory cytokines in dairy cattle [34]. Furthermore, the downregulation of miR-145 in response to Staphylococcus aureus-induced mastitis, through the targeting of FSCN1 in dairy cows, plays a crucial role in regulating immune response [35]. Similarly, the bovine viral diarrhea virus (BVDV) strain NADL induces the upregulation of bta-miR-2904 in MDBK cells, which significantly reduces BVDV replication by inhibiting autophagy in MDBK cells through the targeting of the autophagy-related gene 13 (ATG13) [36]. 

Exosomal sequencing results revealed that the 3 miR-302 family, including bta-miR-302a, bta-miR-302b, and bta-miR-302d, were upregulated in serum-derived exosomes from LSDV-infected cattle compared to the control group. Recent research has highlighted the importance of this family in maintaining self-renewal and stemness in pluripotent cells. Additionally, it regulates differentiation, cell cycle progression, and apoptosis in embryonic stem cells and primed pluripotent stem cells [37]. Furthermore, the miR-302 family has been shown to be involved in cell proliferation, the inhibition of pathogenic replication, and the induction of immune responses to pathogens [38]. For instance, miR-302b is caused by Toll-like receptor 2 (TLR2) and TLR4 through the ERK-p38-NF-κB signaling pathway during infection with the Gram-negative bacterium Pseudomonas aeruginosa [39]. Influenza A virus infection has been shown to downregulate miR-302 expression, which in turn, activates the expression of IFNβ, TNF-α, IL-6, IL-8, CCL2, and CCL5 while inhibiting viral replication [38].

Moreover, the downregulation of the bta-let-7 miRNAs during LPS induction may partially inhibit the NF-κB pathway by targeting and interacting with Ras, a key component of the NF-κB cascade, thereby influencing the regulation of both anti-inflammatory and pro-inflammatory cytokine expression [40]. Additionally, these bta-let-7 miRNA families play a pivotal role in the replication and modulation of various infectious diseases, including HIV, PRRS, highly pathogenic avian influenza virus, swine influenza virus, SARS-CoV-2, hepatitis B and C viruses, Ebola virus, Enterovirus 71, and Pestivirus [12,41,42,43]. This downregulation impacts the immune system by regulating pathways such as PI3K-Akt signaling, mTOR signaling, Ras1 signaling, and MAPK signaling [40,41,43]. We identified six let-7 family miRNAs, all of which were downregulated in the LSDV-infected cattle compared to the non-infected control. Furthermore, the miR-let-7 family has been shown to induce the antiviral IFN response and inhibit protease activity by regulating the expression of Bach1 and BCL-2 [42]. The target genes predicted in this study indicate that miRNA-let-7 is associated with various immune and immune-related genes, such as cytokine, chemokines, TNF family, MAPK family, STAT, cytotoxic, or regulatory genes. Therefore, the miRNA-let-7 family may interact with the regulators, immune system control, and replication of LSDV in cattle. Our findings indicate that these DE miRNAs may play critical roles in regulating LSDV replication and regulating the host immune response.

## 5. Conclusions

In summary, we conducted a comprehensive analysis of miRNA profiles in exosomes derived from both control and LSDV-infected cattle. Among the 302 mature miRNAs examined, 59 miRNAs exhibited significant differential expression between the control and LSDV-infected groups at 21 dpi, with 18 miRNAs upregulated and 41 downregulated. Additionally, we identified 708 potentially novel cattle miRNAs corresponding to 710 genomic loci. Furthermore, through GO functional enrichment analysis and KEGG/Reactome signaling pathway analysis, we determined that these miRNAs are involved in biological processes relevant to viral replication, immune response, virus–host interactions, and signal transduction. Consequently, our study provides valuable insights into the regulatory mechanisms of exosomes in the host response to LSDV infection, offering insights that could inform the identification of new biomarkers and the understanding of the pathogenesis of LSDV.

## Figures and Tables

**Table 1:** Primers used for reverse transcription quantitative real-time PCR (RT-qPCR) in this study.

miRNAs	Mature Accession	Sequences (5′→3′)
bta-miR-1281	MIMAT0009962	TCGCCTCCTCCTCTCCC
bta-miR-12034	MIMAT0046727	CCCCGGGGAGCCCGGCGGT
bta-miR-11985	MIMAT0046381	CCCACCGCTCTCCTCCCGCC
bta-let-7i	MIMAT0003851	TGAGGTAGTAGTTTGTGCTGTT
bta-miR-17-5p	MIMAT0003815	CAAAGTGCTTACAGTGCAGGTAGT
Bovine U6	Forward	CTCGCTTCGGCAGCACATATACT
	Reverse	ACGCTTCACGAATTTGCGTGTC

**Table 2:** Data statistics for miRNA-seq analysis in control and LSDV-infected bovines.

Sample ID	Total Read Bases	Total Reads	Processed Reads	Mapped Reads	Q20 (%)	Known miRNA	Novel miRNA Candidates	Known miRNA in Species (miRBase v22.1)
Control-1	915,554,958	17,952,058	2,117,434	7651 (0.36%)	90.12	71	161	1030
Control-2	2,150,018,730	42,157,230	10,798,041	580,689 (5.38%)	92.17	245	223	1030
Control-3	2,385,501,183	46,774,533	5,522,022	17,320 (0.31%)	90.43	87	226	1030
HT10-1	2,195,912,253	43,057,103	19,501,812	218,735 (1.12%)	92.94	68	73	1030
HT10-2	2,075,959,539	40,705,089	19,911,858	223,989 (1.12%)	93.38	60	88	1030
HT10-3	2,130,712,782	41,778,682	19,789,907	236,703 (1.2%)	92.70	69	58	1030

**Table 3:** Differential expression of mature miRNA genes in serum samples of LSDV-infected bovines compared to their expression in the non-infected control group.

No.	Mature miRNA	Sequence Length	miRBase Sequence	Fold Change (HT10/Control)	*p*-Value
1	bta-miR-302d	21	UAAGUGCUUCCAUGUUUUAGU	601.41	2.32 × 10^−5^
2	bta-miR-302a	22	AAGUGCUUCCAUGUUUUAGUGA	573.38	9.93 × 10^−5^
3	bta-miR-1777b	20	GGGGGCGGUGGGGGGCGGGG	361.52	3.04 × 10^−26^
4	bta-miR-1777a	20	UGGGGGCGGUGGGGGGCGGG	305.37	9.16 × 10^−26^
5	bta-miR-11988	22	AAGGGGACGACAGAGGAUGAGA	275.14	6.33 × 10^−47^
6	bta-miR-215	22	AUGACCUAUGAAUUGACAGACA	186.85	1.43 × 10^−3^
7	bta-miR-302b	23	UAAGUGCUUCCAUGUUUUAGUAG	174.71	3.24 × 10^−3^
8	bta-miR-1281	17	UCGCCUCCUCCUCUCCC	114.72	1.83 × 10^−2^
9	bta-miR-2285as	22	AAAAAGUUCGUUCGGGUUUUCU	108.36	1.79 × 10^−2^
10	bta-miR-12034	19	CCCCGGGGAGCCCGGCGGU	76.49	2.88 × 10^−44^
11	bta-miR-11985	20	CCCACCGCUCUCCUCCCGCC	58.55	5.97 × 10^−8^
12	bta-miR-1246	19	AAUGGAUUUUUGGAGCAGG	28.25	1.45 × 10^−14^
13	bta-miR-574	24	UGAGUGUGUGUGUGUGAGUGUGUG	17.35	8.04 × 10^−6^
14	bta-miR-11972	21	GGGGCGGGAGCGGCCGGGGUC	12.19	2.16 × 10^−6^
15	bta-miR-2285f	22	AAAACCUGAAUGAACUUUUUGG	9.72	3.56 × 10^−2^
16	bta-miR-194	22	UGUAACAGCAACUCCAUGUGGA	9.41	2.37 × 10^−2^
17	bta-miR-2904	19	GGGAGCCUCGGUUGGCCUC	5.08	1.33 × 10^−2^
18	bta-miR-12030	19	CCCGGGGCCCGGAGCGGCC	3.84	4.59 × 10^−2^
19	bta-let-7b	22	UGAGGUAGUAGGUUGUGUGGUU	−2.22	9.60 × 10^−3^
20	bta-miR-342	25	UCUCACACAGAAAUCGCACCCAUCU	−4.02	3.10 × 10^−2^
21	bta-let-7a-5p	22	UGAGGUAGUAGGUUGUAUAGUU	−4.89	1.52 × 10^−8^
22	bta-let-7f	22	UGAGGUAGUAGAUUGUAUAGUU	−7.22	2.01 × 10^−9^
23	bta-miR-6529a	21	GAGAGAUCAGAGGCGCAGAGU	−8.50	8.14 × 10^−3^
24	bta-let-7g	22	UGAGGUAGUAGUUUGUACAGUU	−10.39	1.79 × 10^−3^
25	bta-miR-6119-5p	23	AGAGGUAAAAAAUUGAUUUGACU	−12.67	3.00 × 10^−2^
26	bta-miR-16b	21	UAGCAGCACGUAAAUAUUGGC	−15.57	1.95 × 10^−2^
27	bta-miR-191	23	CAACGGAAUCCCAAAAGCAGCUG	−24.45	3.08 × 10^−7^
28	bta-miR-345-3p	21	GCUGACUCCUAGUCCAGUGCU	−26.95	3.01 × 10^−3^
29	bta-miR-21-5p	24	UAGCUUAUCAGACUGAUGUUGACU	−32.02	6.08 × 10^−5^
30	bta-miR-24-3p	22	UGGCUCAGUUCAGCAGGAACAG	−33.08	9.37 × 10^−11^
31	bta-miR-23b-3p	21	GGGUUCCUGGCAUGCUGAUUU	−37.68	4.48 × 10^−8^
32	bta-miR-10174-3p	21	GGGUUCCUGGCAUGCUGAUUU	−37.68	3.94 × 10^−8^
33	bta-miR-151-3p	21	CUAGACUGAAGCUCCUUGAGG	−37.80	5.59 × 10^−5^
34	bta-miR-92a	22	UAUUGCACUUGUCCCGGCCUGU	−51.57	2.34 × 10^−3^
35	bta-miR-423-5p	23	UGAGGGGCAGAGAGCGAGACUUU	−67.91	5.16 × 10^−20^
36	bta-miR-324	25	UCUCACACAGAAAUCGCACCCAUCU	−69.32	4.77 × 10^−2^
37	bta-miR-126-3p	21	CAUUAUUACUUUUGGUACGCG	−91.12	2.72 × 10^−12^
38	bta-miR-23a	22	AUCACAUUGCCAGGGAUUUCCA	−106.75	4.99 × 10^−22^
39	bta-miR-126-5p	21	CAUUAUUACUUUUGGUACGCG	−112.33	2.59 × 10^−11^
40	bta-let-7i	22	UGAGGUAGUAGUUUGUGCUGUU	−150.39	1.59 × 10^−2^
41	bta-miR-185	22	UGGAGAGAAAGGCAGUUCCUGA	−153.36	1.04 × 10^−8^
42	bta-miR-221	22	AGCUACAUUGUCUGCUGGGUUU	−183.86	3.14 × 10^−2^
43	bta-miR-28	22	AAGGAGCUCACAGUCUAUUGAG	−188.08	2.04 × 10^−2^
44	bta-miR-19b	23	UGUGCAAAUCCAUGCAAAACUGA	−199.49	1.46 × 10^−9^
45	bta-miR-145	23	GUCCAGUUUUCCCAGGAAUCCCU	−250.78	1.03 × 10^−2^
46	bta-miR-16a	22	UAGCAGCACGUAAAUAUUGGUG	−256.99	1.09 × 10^−2^
47	bta-miR-130b	22	CAGUGCAAUGAUGAAAGGGCAU	−270.20	1.33 × 10^−2^
48	bta-miR-17-5p	24	CAAAGUGCUUACAGUGCAGGUAGU	−364.05	2.82 × 10^−3^
49	bta-miR-29b	23	UAGCACCAUUUGAAAUCAGUGUU	−484.43	7.10 × 10^−4^
50	bta-miR-378c	21	ACUGGACUUGGAGUCAGAAGU	−488.46	3.70 × 10^−4^
51	bta-miR-93	22	CAAAGUGCUGUUCGUGCAGGUA	−594.37	1.37 × 10^−4^
52	bta-miR-374b	22	AUAUAAUACAACCUGCUAAGUG	−614.88	1.66 × 10^−4^
53	bta-miR-378	22	ACUUGACUUGGAGUCAGAAGGC	−1058.64	5.48 × 10^−6^
54	bta-miR-150	23	UCUCCCAACCCUUGUACCAGUGU	−2074.21	6.00 × 10^−5^
55	bta-miR-25	22	CAUUGCACUUGUCUCGGUCUGA	−2478.04	1.77 × 10^−9^
56	bta-miR-142-5p	20	CAUAAAGUAGAAAGCACUAC	−2755.21	5.80 × 10^−10^
57	bta-miR-223	22	UGUCAGUUUGUCAAAUACCCCA	−3456.07	4.25 × 10^−09^
58	bta-let-7d	22	AGAGGUAGUAGGUUGCAUAGUU	−3596.72	5.78 × 10^−11^
59	bta-miR-142-3p	22	AGUGUUUCCUACUUUAUGGAUG	−3816.75	3.53 × 10^−20^

## Data Availability

The original contributions presented in the study are included in the article. Further inquiries can be directed to the corresponding authors.

## Supplementary Materials

The following supporting information can be downloaded at: https://www.mdpi.com/article/10.3390/pathogens14020176/s1, Table S1: Total and normalized sequence counts for known bovine miRNAs expressed in serum samples of LSDV-infected and control groups; Table S2: Gene Ontology (GO) enrichment analysis based on DE miRNAs in the serum of LSDV-infected bovines compared to their expression in the non-infected control. Criteria: *p* < 0.05, number of genes in each GO term > 5, and fold change of Log2-treatment/control ≥ 2. (A) Biological process, (B) cellular component, and (C) molecular function; Table S3: (A) KEGG pathway and (B) Reactome pathway enrichment analyses based on DE miRNAs in the serum of LSDV-infected bovines compared to their expression in the non-infected controls. Criteria: *p* < 0.05, number of genes in each pathway term >5, and fold change of Log2-treatment/control ≥ 2; Table S4: Identification of novel bovine miRNAs based on seed sequence alignments.

## Abbreviations

The following abbreviations are used in this manuscript:

miRNA	Micro RNA
LSDV	Lumpy skin disease virus
GO	Gene Ontology
MDBK	Madin-Darby bovine kidney
NTA	Nanoparticle tracking analysis
DE	Differentially expressed
MAPK	Mitogen-activated protein kinase
PI3K-AKT	Phosphatidylinositol 3-kinase- protein kinase B
ncRNAs	Non-coding RNAs
MHC	Major histocompatibility complex
snRNA	Small nuclear RNA
snoRNA	Small nucleolar RNA
TLR	Toll-like receptor
IL	Interleukin
IFN	Interferon
NF-κB	Nuclear factor kappa B
RT-qPCR	Reverse transcription-quantitative polymerase chain reaction
PRR	Pattern-recognition receptor
JAK	Janus kinase
STAT	Signal transducers and activators of transcription
PBS	Phosphate-buffered saline
DMEM	Dulbecco’s modified essential medium
RPMI	Roswell Park Memorial Institute
FBS	Fetal bovine serum
ANOVA	Analysis of variance
SEM	Standard error of the mean
CCL	Chemokine (C-C motif) ligand
CXCR	C-X-C chemokine receptor
