# Supplementary material for: Differentially Expressed miRNA Profiles in Serum-Derived Exosomes from Cattle Infected with Lumpy Skin Disease Virus
Source: Pathogens. 2025 Feb 10;14(2):176. doi: 10.3390/pathogens14020176 (PMC11858326; doi:10.3390/pathogens14020176)
Supplement: Supplementary file 1 [file pathogens-14-00176-s001.zip › pathogens-3467702-supplementary.pdf]

Table S1: Total and normalized sequence counts for known bovine miRNAs expressed in serum samples of LSDV-infected and control groups.

No	Mature_ID	Mature_Accession	RPM						Read_Count					
			Ctrl-01	Ctrl-02	Ctrl-03	HT10-01	HT10-02	HT10-03	Ctrl-01	Ctrl-02	Ctrl-03	HT10-01	HT10-02	HT10-03
1	bta-miR-1246	MIMAT0024567	539,878.3	800,890.6	644,209.9	906,397.5	894,322.9	826,758.6	3,371.0	431,656.0	9,218.0	188,731.0	190,125.0	185,876.0
2	bta-miR-142-3p	MIMAT0003791	12,492.0	5,297.1	9,504.5	-	-	-	78.0	2,855.0	136.0	-	-	-
3	bta-miR-142-5p	MIMAT0003790	10,249.8	3,965.0	5,590.9	-	-	-	64.0	2,137.0	80.0	-	-	-
4	bta-miR-25	MIMAT0003853	8,007.7	2,387.9	8,875.5	-	-	-	50.0	1,287.0	127.0	-	-	-
5	bta-miR-21-5p	MIMAT0003528	4,164.0	1,434.2	2,376.1	-	-	8.9	26.0	773.0	34.0	-	-	2.0
6	bta-miR-378	MIMAT0009305	4,003.8	1,825.7	1,327.8	-	-	-	25.0	984.0	19.0	-	-	-
7	bta-miR-374b	MIMAT0009302	3,203.1	408.2	1,747.2	-	-	-	20.0	220.0	25.0	-	-	-
8	bta-miR-378c	MIMAT0025551	1,921.8	886.9	419.3	-	-	-	12.0	478.0	6.0	-	-	-
9	bta-miR-29b	MIMAT0003828	1,761.7	950.0	349.4	-	-	-	11.0	512.0	5.0	-	-	-
10	bta-miR-221	MIMAT0003529	800.8	339.5	69.9	-	-	-	5.0	183.0	1.0	-	-	-
11	bta-miR-145	MIMAT0003542	640.6	363.7	698.9	-	-	-	4.0	196.0	10.0	-	-	-
12	bta-miR-16a	MIMAT0009242	480.5	26.0	1,747.2	-	-	-	3.0	14.0	25.0	-	-	-
13	bta-miR-130b	MIMAT0009224	320.3	575.2	559.1	-	-	-	2.0	310.0	8.0	-	-	-
14	bta-let-7i	MIMAT0003851	160.2	400.8	69.9	-	-	-	1.0	216.0	1.0	-	-	-
15	bta-miR-150	MIMAT0003845	160.2	6,310.2	2,026.7	-	-	-	1.0	3,401.0	29.0	-	-	-
16	bta-miR-28	MIMAT0009272	160.2	161.4	1,048.3	-	-	-	1.0	87.0	15.0	-	-	-
17	bta-miR-324	MIMAT0009285	160.2	50.1	349.4	-	-	-	1.0	27.0	5.0	-	-	-
18	bta-miR-302a	MIMAT0009278	-	-	-	81.6	37.6	22.2	-	-	-	17.0	8.0	5.0
19	bta-miR-302d	MIMAT0009279	-	-	-	81.6	37.6	31.1	-	-	-	17.0	8.0	7.0
20	bta-miR-302b	MIMAT0009280	-	-	-	19.2	4.7	22.2	-	-	-	4.0	1.0	5.0
21	bta-miR-2285as	MIMAT0046338	-	-	-	14.4	4.7	8.9	-	-	-	3.0	1.0	2.0
22	bta-miR-1281	MIMAT0009962	-	-	-	4.8	4.7	22.2	-	-	-	1.0	1.0	5.0
23	bta-miR-12034	MIMAT0046727	67,905.2	23,899.3	32,287.4	88,065.1	100,498.1	166,596.2	424.0	12,881.0	462.0	18,337.0	21,365.0	37,455.0
24	bta-let-7a-5p	MIMAT0003844	49,647.7	10,115.6	34,034.5	163.3	230.5	240.2	310.0	5,452.0	487.0	34.0	49.0	54.0
25	bta-miR-2887	MIMAT0013845	30,909.7	6,495.7	17,471.5	710.8	733.8	978.5	193.0	3,501.0	250.0	148.0	156.0	220.0
26	bta-miR-423-5p	MIMAT0012537	30,108.9	20,535.5	26,906.1	9.6	28.2	8.9	188.0	11,068.0	385.0	2.0	6.0	2.0
27	bta-let-7f	MIMAT0003519	28,187.1	7,920.7	30,330.6	86.4	80.0	151.2	176.0	4,269.0	434.0	18.0	17.0	34.0
28	bta-miR-126-3p	MIMAT0003540	23,382.4	3,323.0	16,423.2	14.4	-	-	146.0	1,791.0	235.0	3.0	-	-
29	bta-let-7d	MIMAT0003810	20,659.8	1,484.3	10,692.6	-	-	-	129.0	800.0	153.0	-	-	-
30	bta-miR-23a	MIMAT0003827	19,218.4	6,770.3	16,143.7	4.8	4.7	4.4	120.0	3,649.0	231.0	1.0	1.0	1.0
31	bta-miR-24-3p	MIMAT0003840	18,738.0	2,495.5	14,117.0	19.2	-	13.3	117.0	1,345.0	202.0	4.0	-	3.0
32	bta-let-7b	MIMAT0004331	12,171.7	3,710.8	7,198.3	105.7	127.0	133.4	76.0	2,000.0	103.0	22.0	27.0	30.0
33	bta-miR-10174-3p	MIMAT0040928	11,531.1	3,796.1	9,784.1	4.8	14.1	4.4	72.0	2,046.0	140.0	1.0	3.0	1.0
34	bta-miR-23b-3p	MIMAT0003852	11,531.1	3,796.1	9,784.1	4.8	14.1	4.4	72.0	2,046.0	140.0	1.0	3.0	1.0
35	bta-miR-185	MIMAT0009247	10,730.3	3,978.0	6,219.9	4.8	-	-	67.0	2,144.0	89.0	1.0	-	-

36	bta-miR-19b	MIMAT0004337	9,609.2	7,145.1	5,031.8	-	-	4.4	60.0	3,851.0	72.0	-	-	1.0
37	bta-miR-151-3p	MIMAT0003524	8,007.7	797.8	3,284.6	-	9.4	-	50.0	430.0	47.0	-	2.0	-
38	bta-miR-191	MIMAT0003819	6,406.1	5,987.3	10,413.0	4.8	-	35.6	40.0	3,227.0	149.0	1.0	-	8.0
39	bta-miR-126-5p	MIMAT0004328	5,765.5	9,328.9	7,058.5	4.8	4.7	-	36.0	5,028.0	101.0	1.0	1.0	-
40	bta-let-7c	MIMAT0004332	4,644.5	458.3	698.9	-	23.5	44.5	29.0	247.0	10.0	-	5.0	10.0
41	bta-miR-223	MIMAT0009270	4,484.3	8,186.0	5,730.7	-	-	-	28.0	4,412.0	82.0	-	-	-
42	bta-miR-6529a	MIMAT0025565	3,843.7	692.1	2,236.4	-	4.7	22.2	24.0	373.0	32.0	-	1.0	5.0
43	bta-miR-342	MIMAT0003846	2,882.8	4,055.9	2,585.8	52.8	28.2	31.1	18.0	2,186.0	37.0	11.0	6.0	7.0
44	bta-miR-12030	MIMAT0046723	2,402.3	98.3	559.1	76.8	94.1	137.9	15.0	53.0	8.0	16.0	20.0	31.0
45	bta-miR-143	MIMAT0009233	2,402.3	81.6	-	28.8	32.9	13.3	15.0	44.0	-	6.0	7.0	3.0
46	bta-miR-11980	MIMAT0046368	1,921.8	16.7	-	43.2	-	17.8	12.0	9.0	-	9.0	-	4.0
47	bta-miR-1	MIMAT0009214	1,761.7	115.0	-	-	-	-	11.0	62.0	-	-	-	-
48	bta-miR-214	MIMAT0003825	1,601.5	40.8	-	-	9.4	-	10.0	22.0	-	-	2.0	-
49	bta-miR-345-3p	MIMAT0012535	1,601.5	252.3	2,166.5	-	-	4.4	10.0	136.0	31.0	-	-	1.0
50	bta-miR-6528	MIMAT0025562	1,601.5	-	1,118.2	14.4	-	-	10.0	-	16.0	3.0	-	-
51	bta-miR-574	MIMAT0024577	1,441.4	116.9	419.3	341.0	291.6	258.0	9.0	63.0	6.0	71.0	62.0	58.0
52	bta-let-7g	MIMAT0003838	1,121.1	1,781.2	8,316.4	14.4	4.7	22.2	7.0	960.0	119.0	3.0	1.0	5.0
53	bta-miR-16b	MIMAT0003525	1,121.1	189.2	978.4	-	-	4.4	7.0	102.0	14.0	-	-	1.0
54	bta-miR-4444	MIMAT0036978	1,121.1	-	-	-	-	-	7.0	-	-	-	-	-
55	bta-miR-93	MIMAT0003837	1,121.1	603.0	2,655.7	-	-	-	7.0	325.0	38.0	-	-	-
56	bta-miR-130a	MIMAT0009223	960.9	113.2	-	9.6	28.2	31.1	6.0	61.0	-	2.0	6.0	7.0
57	bta-miR-140	MIMAT0003789	960.9	259.8	-	-	-	-	6.0	140.0	-	-	-	-
58	bta-miR-320a	MIMAT0003534	960.9	96.5	-	-	-	-	6.0	52.0	-	-	-	-
59	bta-miR-330	MIMAT0009290	960.9	68.6	-	-	-	-	6.0	37.0	-	-	-	-
60	bta-miR-98	MIMAT0003809	960.9	439.7	69.9	-	-	35.6	6.0	237.0	1.0	-	-	8.0
61	bta-miR-11975	MIMAT0046354	800.8	1,200.4	2,865.3	72.0	37.6	62.3	5.0	647.0	41.0	15.0	8.0	14.0
62	bta-miR-11976	MIMAT0046357	800.8	1,174.5	2,725.6	62.4	37.6	62.3	5.0	633.0	39.0	13.0	8.0	14.0
63	bta-miR-103	MIMAT0003521	640.6	380.4	-	-	-	-	4.0	205.0	-	-	-	-
64	bta-miR-12002b	MIMAT0046694	640.6	-	-	-	-	-	4.0	-	-	-	-	-
65	bta-miR-186	MIMAT0003818	640.6	539.9	-	4.8	-	-	4.0	291.0	-	1.0	-	-
66	bta-miR-92a	MIMAT0009383	640.6	6,749.9	349.4	9.6	-	-	4.0	3,638.0	5.0	2.0	-	-
67	bta-miR-181a	MIMAT0003543	480.5	274.6	-	4.8	-	-	3.0	148.0	-	1.0	-	-
68	bta-miR-345-5p	MIMAT0003800	480.5	215.2	-	-	-	-	3.0	116.0	-	-	-	-
69	bta-miR-421	MIMAT0009314	480.5	81.6	-	-	9.4	4.4	3.0	44.0	-	-	2.0	1.0
70	bta-miR-17-5p	MIMAT0003815	320.3	723.6	978.4	-	-	-	2.0	390.0	14.0	-	-	-
71	bta-miR-2484	MIMAT0012077	320.3	-	-	-	4.7	-	2.0	-	-	-	1.0	-
72	bta-miR-11972	MIMAT0046341	160.2	866.5	2,795.4	595.5	508.0	916.3	1.0	467.0	40.0	124.0	108.0	206.0
73	bta-miR-149-3p	MIMAT0024571	160.2	-	-	-	-	-	1.0	-	-	-	-	-
74	bta-miR-2904	MIMAT0013862	160.2	868.3	698.9	148.9	98.8	262.4	1.0	468.0	10.0	31.0	21.0	59.0

75	bta-miR-3141	MIMAT0024573	160.2	-	209.7	-	-	-	1.0	-	3.0	-	-	-
76	bta-miR-338	MIMAT0009292	160.2	20.4	-	-	-	-	1.0	11.0	-	-	-	-
77	bta-miR-486	MIMAT0009329	-	3,016.9	1,747.2	-	-	-	-	1,626.0	25.0	-	-	-
78	bta-miR-19a	MIMAT0004336	-	1,484.3	1,118.2	-	-	-	-	800.0	16.0	-	-	-
79	bta-miR-29a	MIMAT0003518	-	1,094.7	-	-	-	-	-	590.0	-	-	-	-
80	bta-miR-326	MIMAT0009286	-	968.5	1,118.2	-	-	-	-	522.0	16.0	-	-	-
81	bta-miR-101	MIMAT0003520	-	690.2	139.8	-	-	-	-	372.0	2.0	-	-	-
82	bta-miR-133a	MIMAT0009225	-	688.4	-	-	-	-	-	371.0	-	-	-	-
83	bta-miR-301a	MIMAT0009276	-	686.5	69.9	-	-	-	-	370.0	1.0	-	-	-
84	bta-miR-133b	MIMAT0009226	-	680.9	-	-	-	-	-	367.0	-	-	-	-
85	bta-miR-22-3p	MIMAT0012536	-	538.1	279.5	9.6	4.7	17.8	-	290.0	4.0	2.0	1.0	4.0
86	bta-miR-205	MIMAT0003545	-	458.3	-	-	-	-	-	247.0	-	-	-	-
87	bta-miR-30c	MIMAT0003850	-	434.2	-	-	-	-	-	234.0	-	-	-	-
88	bta-miR-6119-5p	MIMAT0024588	-	432.3	2,585.8	-	4.7	4.4	-	233.0	37.0	-	1.0	1.0
89	bta-miR-29c	MIMAT0003829	-	411.9	838.6	-	-	-	-	222.0	12.0	-	-	-
90	bta-miR-423-3p	MIMAT0003831	-	369.2	-	-	-	-	-	199.0	-	-	-	-
91	bta-miR-1468	MIMAT0013592	-	315.4	419.3	-	-	-	-	170.0	6.0	-	-	-
92	bta-miR-484	MIMAT0003535	-	302.4	-	-	-	-	-	163.0	-	-	-	-
93	bta-miR-425-5p	MIMAT0003832	-	280.2	139.8	-	-	-	-	151.0	2.0	-	-	-
94	bta-miR-6119-3p	MIMAT0024589	-	280.2	-	-	-	-	-	151.0	-	-	-	-
95	bta-miR-2285bg	MIMAT0046386	-	276.5	-	-	-	-	-	149.0	-	-	-	-
96	bta-let-7e	MIMAT0004333	-	265.3	1,677.3	14.4	4.7	8.9	-	143.0	24.0	3.0	1.0	2.0
97	bta-miR-144	MIMAT0009234	-	256.0	-	-	-	-	-	138.0	-	-	-	-
98	bta-miR-125a	MIMAT0003538	-	235.6	-	-	-	-	-	127.0	-	-	-	-
99	bta-miR-328	MIMAT0009287	-	215.2	-	-	-	-	-	116.0	-	-	-	-
100	bta-miR-2285o	MIMAT0025530	-	213.4	-	-	-	-	-	115.0	-	-	-	-
101	bta-miR-331-3p	MIMAT0004339	-	205.9	209.7	-	-	-	-	111.0	3.0	-	-	-
102	bta-miR-92b	MIMAT0009384	-	200.4	-	-	-	-	-	108.0	-	-	-	-
103	bta-miR-30b-5p	MIMAT0003547	-	196.7	-	-	-	-	-	106.0	-	-	-	-
104	bta-miR-151-5p	MIMAT0003523	-	180.0	-	-	-	-	-	97.0	-	-	-	-
105	bta-miR-192	MIMAT0003820	-	178.1	559.1	4.8	32.9	4.4	-	96.0	8.0	1.0	7.0	1.0
106	bta-miR-1306	MIMAT0009974	-	174.4	489.2	-	-	-	-	94.0	7.0	-	-	-
107	bta-miR-219-5p	MIMAT0009269	-	161.4	-	-	-	-	-	87.0	-	-	-	-
108	bta-miR-26b	MIMAT0003531	-	161.4	-	-	-	-	-	87.0	-	-	-	-
109	bta-miR-339a	MIMAT0009293	-	161.4	-	-	-	-	-	87.0	-	-	-	-
110	bta-miR-339b	MIMAT0012038	-	161.4	-	-	-	-	-	87.0	-	-	-	-
111	bta-miR-222	MIMAT0003530	-	157.7	-	-	-	-	-	85.0	-	-	-	-
112	bta-miR-139	MIMAT0003788	-	155.9	-	-	-	-	-	84.0	-	-	-	-
113	bta-miR-26a	MIMAT0003516	-	148.4	-	-	-	-	-	80.0	-	-	-	-

114	bta-miR-2285bf	MIMAT0046382	-	139.2	-	-	-	-	-	75.0	-	-	-	-
115	bta-miR-451	MIMAT0009323	-	128.0	209.7	-	-	-	-	69.0	3.0	-	-	-
116	bta-miR-20a	MIMAT0003527	-	103.9	-	-	-	-	-	56.0	-	-	-	-
117	bta-miR-2419-5p	MIMAT0011985	-	100.2	-	-	-	-	-	54.0	-	-	-	-
118	bta-miR-301b	MIMAT0009277	-	96.5	1,118.2	-	-	-	-	52.0	16.0	-	-	-
119	bta-miR-425-3p	MIMAT0003833	-	94.6	-	-	-	-	-	51.0	-	-	-	-
120	bta-miR-106a	MIMAT0003784	-	92.8	69.9	-	-	-	-	50.0	1.0	-	-	-
121	bta-miR-1343-3p	MIMAT0011840	-	92.8	-	-	-	-	-	50.0	-	-	-	-
122	bta-miR-125b	MIMAT0003539	-	89.1	-	-	-	-	-	48.0	-	-	-	-
123	bta-miR-181b	MIMAT0003793	-	89.1	-	-	-	-	-	48.0	-	-	-	-
124	bta-miR-378d	MIMAT0036972	-	89.1	-	-	-	-	-	48.0	-	-	-	-
125	bta-miR-107	MIMAT0003785	-	85.3	-	-	-	-	-	46.0	-	-	-	-
126	bta-miR-33a	MIMAT0009294	-	85.3	-	-	-	-	-	46.0	-	-	-	-
127	bta-miR-122	MIMAT0003849	-	83.5	-	-	-	-	-	45.0	-	-	-	-
128	bta-miR-664b	MIMAT0030438	-	83.5	-	4.8	-	4.4	-	45.0	-	1.0	-	1.0
129	bta-miR-1307	MIMAT0009969	-	81.6	-	-	-	-	-	44.0	-	-	-	-
130	bta-miR-11988	MIMAT0046393	-	76.1	209.7	1,426.4	1,444.1	1,565.7	-	41.0	3.0	297.0	307.0	352.0
131	bta-miR-194	MIMAT0009254	-	76.1	-	9.6	42.3	31.1	-	41.0	-	2.0	9.0	7.0
132	bta-miR-11985	MIMAT0046381	-	72.4	-	124.9	117.6	346.9	-	39.0	-	26.0	25.0	78.0
133	bta-miR-383	MIMAT0009309	-	72.4	-	-	-	-	-	39.0	-	-	-	-
134	bta-miR-874	MIMAT0009378	-	70.5	-	-	-	-	-	38.0	-	-	-	-
135	bta-miR-29d-3p	MIMAT0009275	-	68.6	139.8	-	-	-	-	37.0	2.0	-	-	-
136	bta-miR-660	MIMAT0004344	-	66.8	-	-	-	8.9	-	36.0	-	-	-	2.0
137	bta-miR-1271	MIMAT0009975	-	63.1	-	24.0	-	-	-	34.0	-	5.0	-	-
138	bta-miR-128	MIMAT0003541	-	63.1	908.5	-	-	-	-	34.0	13.0	-	-	-
139	bta-miR-27a-3p	MIMAT0003532	-	63.1	-	-	-	-	-	34.0	-	-	-	-
140	bta-miR-29d-5p	MIMAT0026919	-	61.2	-	-	-	-	-	33.0	-	-	-	-
141	bta-miR-30e-5p	MIMAT0003799	-	61.2	-	-	-	-	-	33.0	-	-	-	-
142	bta-miR-532	MIMAT0003848	-	57.5	-	-	-	-	-	31.0	-	-	-	-
143	bta-miR-197	MIMAT0009257	-	52.0	-	-	-	-	-	28.0	-	-	-	-
144	bta-miR-193a-5p	MIMAT0003794	-	50.1	139.8	9.6	-	-	-	27.0	2.0	2.0	-	-
145	bta-miR-206	MIMAT0009260	-	48.2	1,467.6	-	-	-	-	26.0	21.0	-	-	-
146	bta-miR-99a-5p	MIMAT0003537	-	48.2	-	-	-	-	-	26.0	-	-	-	-
147	bta-miR-2284x	MIMAT0017395	-	46.4	-	-	-	-	-	25.0	-	-	-	-
148	bta-miR-2284y	MIMAT0024579	-	46.4	-	-	-	-	-	25.0	-	-	-	-
149	bta-miR-2285av	MIMAT0046347	-	46.4	-	-	-	-	-	25.0	-	-	-	-
150	bta-miR-411a	MIMAT0009312	-	46.4	-	-	-	-	-	25.0	-	-	-	-
151	bta-miR-193b	MIMAT0009253	-	44.5	-	-	-	-	-	24.0	-	-	-	-
152	bta-let-7a-3p	MIMAT0004330	-	42.7	-	-	-	-	-	23.0	-	-	-	-

153	bta-miR-27b	MIMAT0003546	-	42.7	-	9.6	-	-	-	23.0	-	2.0	-	-
154	bta-miR-30b-3p	MIMAT0012534	-	42.7	-	-	-	-	-	23.0	-	-	-	-
155	bta-miR-877	MIMAT0009381	-	42.7	2,096.6	-	-	-	-	23.0	30.0	-	-	-
156	bta-miR-1839	MIMAT0009968	-	40.8	-	-	-	-	-	22.0	-	-	-	-
157	bta-miR-18a	MIMAT0003526	-	40.8	-	-	-	-	-	22.0	-	-	-	-
158	bta-miR-210	MIMAT0003824	-	40.8	-	-	-	-	-	22.0	-	-	-	-
159	bta-miR-1388-3p	MIMAT0013591	-	39.0	-	-	-	-	-	21.0	-	-	-	-
160	bta-miR-106b	MIMAT0009218	-	35.3	-	-	-	-	-	19.0	-	-	-	-
161	bta-miR-146b	MIMAT0009235	-	35.3	-	-	-	-	-	19.0	-	-	-	-
162	bta-miR-769	MIMAT0009376	-	33.4	-	-	-	-	-	18.0	-	-	-	-
163	bta-miR-1277	MIMAT0024569	-	31.5	-	-	-	-	-	17.0	-	-	-	-
164	bta-miR-296-5p	MIMAT0026918	-	31.5	-	-	-	-	-	17.0	-	-	-	-
165	bta-miR-17-3p	MIMAT0003816	-	29.7	-	-	-	-	-	16.0	-	-	-	-
166	bta-miR-22-5p	MIMAT0003826	-	29.7	-	-	-	-	-	16.0	-	-	-	-
167	bta-miR-340	MIMAT0009296	-	29.7	-	-	-	-	-	16.0	-	-	-	-
168	bta-miR-6123	MIMAT0024596	-	29.7	-	-	-	-	-	16.0	-	-	-	-
169	bta-miR-15a	MIMAT0004334	-	27.8	-	-	-	-	-	15.0	-	-	-	-
170	bta-miR-2285t	MIMAT0025577	-	27.8	-	-	-	-	-	15.0	-	-	-	-
171	bta-miR-10b	MIMAT0003839	-	26.0	-	-	-	-	-	14.0	-	-	-	-
172	bta-miR-190a	MIMAT0009251	-	26.0	-	-	-	-	-	14.0	-	-	-	-
173	bta-miR-34a	MIMAT0004340	-	26.0	-	-	-	-	-	14.0	-	-	-	-
174	bta-miR-545-5p	MIMAT0003806	-	26.0	-	-	-	8.9	-	14.0	-	-	-	2.0
175	bta-miR-148b	MIMAT0003814	-	24.1	-	-	-	-	-	13.0	-	-	-	-
176	bta-miR-2285j	MIMAT0024585	-	24.1	-	-	-	-	-	13.0	-	-	-	-
177	bta-miR-454	MIMAT0009326	-	24.1	-	-	-	-	-	13.0	-	-	-	-
178	bta-miR-95	MIMAT0009387	-	24.1	-	-	-	-	-	13.0	-	-	-	-
179	bta-miR-100	MIMAT0009215	-	22.3	-	-	-	-	-	12.0	-	-	-	-
180	bta-miR-11973	MIMAT0046349	-	22.3	-	-	-	-	-	12.0	-	-	-	-
181	bta-miR-146a	MIMAT0009236	-	22.3	-	-	-	-	-	12.0	-	-	-	-
182	bta-miR-3432a	MIMAT0017396	-	22.3	-	-	-	13.3	-	12.0	-	-	-	3.0
183	bta-miR-7	MIMAT0003843	-	22.3	-	-	-	-	-	12.0	-	-	-	-
184	bta-miR-760-3p	MIMAT0022951	-	22.3	-	-	-	-	-	12.0	-	-	-	-
185	bta-miR-1777a	MIMAT0012032	-	20.4	-	432.2	390.4	395.9	-	11.0	-	90.0	83.0	89.0
186	bta-miR-335	MIMAT0009291	-	20.4	69.9	-	-	-	-	11.0	1.0	-	-	-
187	bta-miR-505	MIMAT0009341	-	20.4	-	-	-	-	-	11.0	-	-	-	-
188	bta-miR-545-3p	MIMAT0003807	-	20.4	-	-	-	-	-	11.0	-	-	-	-
189	bta-miR-1777b	MIMAT0012046	-	18.6	-	451.4	437.5	444.8	-	10.0	-	94.0	93.0	100.0
190	bta-miR-2355-3p	MIMAT0011892	-	18.6	-	-	-	-	-	10.0	-	-	-	-
191	bta-miR-2411-3p	MIMAT0011973	-	18.6	-	-	-	-	-	10.0	-	-	-	-

192	bta-miR-32	MIMAT0009283	-	18.6	-	-	-	-	-	10.0	-	-	-	-
193	bta-miR-424-5p	MIMAT0013593	-	18.6	-	-	-	-	-	10.0	-	-	-	-
194	bta-miR-6120-3p	MIMAT0024591	-	18.6	-	-	-	-	-	10.0	-	-	-	-
195	bta-miR-652	MIMAT0024578	-	18.6	-	-	-	-	-	10.0	-	-	-	-
196	bta-miR-15b	MIMAT0003792	-	16.7	838.6	-	-	-	-	9.0	12.0	-	-	-
197	bta-miR-208b	MIMAT0009262	-	16.7	-	-	-	-	-	9.0	-	-	-	-
198	bta-miR-1260b	MIMAT0024568	-	14.8	-	-	-	-	-	8.0	-	-	-	-
199	bta-miR-149-5p	MIMAT0024570	-	14.8	-	-	-	-	-	8.0	-	-	-	-
200	bta-miR-188	MIMAT0009249	-	14.8	-	-	-	-	-	8.0	-	-	-	-
201	bta-miR-199b	MIMAT0003821	-	14.8	-	-	-	-	-	8.0	-	-	-	-
202	bta-miR-2285f	MIMAT0024581	-	14.8	-	9.6	14.1	-	-	8.0	-	2.0	3.0	-
203	bta-miR-363	MIMAT0003855	-	14.8	209.7	-	-	-	-	8.0	3.0	-	-	-
204	bta-miR-2285bn	MIMAT0046625	-	13.0	-	-	-	-	-	7.0	-	-	-	-
205	bta-miR-2285ce	MIMAT0046657	-	13.0	-	-	-	-	-	7.0	-	-	-	-
206	bta-miR-2285n	MIMAT0025531	-	13.0	-	-	-	-	-	7.0	-	-	-	-
207	bta-miR-374a	MIMAT0004342	-	13.0	-	-	-	-	-	7.0	-	-	-	-
208	bta-miR-380-3p	MIMAT0003804	-	13.0	-	-	-	-	-	7.0	-	-	-	-
209	bta-miR-7857-3p	MIMAT0040925	-	13.0	-	-	-	-	-	7.0	-	-	-	-
210	bta-miR-224	MIMAT0009271	-	11.1	-	-	-	-	-	6.0	-	-	-	-
211	bta-miR-296-3p	MIMAT0009273	-	11.1	139.8	-	-	-	-	6.0	2.0	-	-	-
212	bta-miR-29e	MIMAT0009953	-	11.1	-	-	-	-	-	6.0	-	-	-	-
213	bta-miR-885	MIMAT0009382	-	11.1	-	-	-	-	-	6.0	-	-	-	-
214	bta-miR-1249	MIMAT0009976	-	9.3	-	-	-	-	-	5.0	-	-	-	-
215	bta-miR-1388-5p	MIMAT0013590	-	9.3	-	-	-	-	-	5.0	-	-	-	-
216	bta-miR-2284j	MIMAT0011827	-	9.3	-	-	-	-	-	5.0	-	-	-	-
217	bta-miR-2284m	MIMAT0011976	-	9.3	-	-	-	-	-	5.0	-	-	-	-
218	bta-miR-2285au	MIMAT0046346	-	9.3	-	-	-	-	-	5.0	-	-	-	-
219	bta-miR-2285bo	MIMAT0046627	-	9.3	-	-	-	-	-	5.0	-	-	-	-
220	bta-miR-2285i	MIMAT0024584	-	9.3	-	-	-	-	-	5.0	-	-	-	-
221	bta-miR-2885	MIMAT0013843	-	9.3	-	-	-	-	-	5.0	-	-	-	-
222	bta-miR-11971	MIMAT0046336	-	7.4	-	-	-	-	-	4.0	-	-	-	-
223	bta-miR-12023	MIMAT0046716	-	7.4	-	-	14.1	-	-	4.0	-	-	3.0	-
224	bta-miR-138	MIMAT0003813	-	7.4	-	-	-	-	-	4.0	-	-	-	-
225	bta-miR-199a-3p	MIMAT0003746	-	7.4	-	-	-	-	-	4.0	-	-	-	-
226	bta-miR-199c	MIMAT0011871	-	7.4	-	-	-	-	-	4.0	-	-	-	-
227	bta-miR-2284w	MIMAT0017393	-	7.4	-	-	-	-	-	4.0	-	-	-	-
228	bta-miR-2285at	MIMAT0046342	-	7.4	-	-	-	-	-	4.0	-	-	-	-
229	bta-miR-2285bq	MIMAT0046635	-	7.4	-	-	-	-	-	4.0	-	-	-	-
230	bta-miR-2285da	MIMAT0046707	-	7.4	-	-	-	-	-	4.0	-	-	-	-

231	bta-miR-2332	MIMAT0011865	-	7.4	-	-	-	-	-	4.0	-	-	-	-
232	bta-miR-2422	MIMAT0011989	-	7.4	-	-	-	-	-	4.0	-	-	-	-
233	bta-miR-2478	MIMAT0012070	-	7.4	-	-	-	-	-	4.0	-	-	-	-
234	bta-miR-30d	MIMAT0003533	-	7.4	-	-	-	-	-	4.0	-	-	-	-
235	bta-miR-33b	MIMAT0009295	-	7.4	-	-	-	-	-	4.0	-	-	-	-
236	bta-miR-1296	MIMAT0009964	-	5.6	-	-	-	-	-	3.0	-	-	-	-
237	bta-miR-208a	MIMAT0009261	-	5.6	-	-	-	-	-	3.0	-	-	-	-
238	bta-miR-2285dd	MIMAT0046712	-	5.6	-	-	-	-	-	3.0	-	-	-	-
239	bta-miR-2285k	MIMAT0024586	-	5.6	-	-	-	-	-	3.0	-	-	-	-
240	bta-miR-331-5p	MIMAT0026716	-	5.6	-	-	-	-	-	3.0	-	-	-	-
241	bta-miR-658	MIMAT0009362	-	5.6	-	-	-	-	-	3.0	-	-	-	-
242	bta-miR-148a	MIMAT0003522	-	3.7	-	-	-	-	-	2.0	-	-	-	-
243	bta-miR-152	MIMAT0009238	-	3.7	-	-	-	-	-	2.0	-	-	-	-
244	bta-miR-196a	MIMAT0009255	-	3.7	-	-	-	-	-	2.0	-	-	-	-
245	bta-miR-20b	MIMAT0003796	-	3.7	-	-	-	-	-	2.0	-	-	-	-
246	bta-miR-3533	MIMAT0036975	-	3.7	1,118.2	-	-	-	-	2.0	16.0	-	-	-
247	bta-miR-935	MIMAT0009385	-	3.7	-	-	-	-	-	2.0	-	-	-	-
248	bta-miR-10167-3p	MIMAT0040914	-	1.9	-	-	-	-	-	1.0	-	-	-	-
249	bta-miR-11977	MIMAT0046359	-	1.9	-	-	-	-	-	1.0	-	-	-	-
250	bta-miR-1949	MIMAT0046755	-	1.9	-	-	-	-	-	1.0	-	-	-	-
251	bta-miR-2305	MIMAT0011817	-	1.9	-	14.4	-	4.4	-	1.0	-	3.0	-	1.0
252	bta-miR-2436-3p	MIMAT0012009	-	1.9	-	-	-	-	-	1.0	-	-	-	-
253	bta-miR-2448-3p	MIMAT0012026	-	1.9	-	-	-	-	-	1.0	-	-	-	-
254	bta-miR-30f	MIMAT0009282	-	1.9	-	-	-	-	-	1.0	-	-	-	-
255	bta-miR-361	MIMAT0003830	-	1.9	-	-	-	-	-	1.0	-	-	-	-
256	bta-miR-494	MIMAT0009334	-	1.9	-	14.4	-	-	-	1.0	-	3.0	-	-
257	bta-miR-2458	MIMAT0012043	-	-	1,118.2	-	-	-	-	-	16.0	-	-	-
258	bta-miR-2336	MIMAT0011869	-	-	1,048.3	-	-	-	-	-	15.0	-	-	-
259	bta-miR-6531	MIMAT0025567	-	-	1,048.3	-	-	-	-	-	15.0	-	-	-
260	bta-miR-2340	MIMAT0011875	-	-	209.7	4.8	4.7	13.3	-	-	3.0	1.0	1.0	3.0
261	bta-miR-10225a	MIMAT0041126	-	-	69.9	-	-	-	-	-	1.0	-	-	-
262	bta-miR-10225b	MIMAT0041127	-	-	69.9	-	-	-	-	-	1.0	-	-	-
263	bta-miR-6518	MIMAT0025536	-	-	-	24.0	-	13.3	-	-	-	5.0	-	3.0
264	bta-miR-2300b-3p	MIMAT0011810	-	-	-	19.2	-	-	-	-	-	4.0	-	-
265	bta-miR-2285cc	MIMAT0046652	-	-	-	14.4	-	-	-	-	-	3.0	-	-
266	bta-miR-12012	MIMAT0046680	-	-	-	9.6	9.4	-	-	-	-	2.0	2.0	-
267	bta-miR-200c	MIMAT0003823	-	-	-	9.6	4.7	-	-	-	-	2.0	1.0	-
268	bta-miR-215	MIMAT0003797	-	-	-	9.6	18.8	22.2	-	-	-	2.0	4.0	5.0
269	bta-miR-2322-3p	MIMAT0011851	-	-	-	9.6	-	-	-	-	-	2.0	-	-

270	bta-miR-26c	MIMAT0016938	-	-	-	9.6	-	-	-	-	-	2.0	-	-
271	bta-miR-7180	MIMAT0046626	-	-	-	9.6	-	-	-	-	-	2.0	-	-
272	bta-miR-10a	MIMAT0003786	-	-	-	4.8	-	-	-	-	-	1.0	-	-
273	bta-miR-12042	MIMAT0046739	-	-	-	4.8	-	-	-	-	-	1.0	-	-
274	bta-miR-2285ag-3p	MIMAT0040923	-	-	-	4.8	-	4.4	-	-	-	1.0	-	1.0
275	bta-miR-2293	MIMAT0011801	-	-	-	4.8	-	-	-	-	-	1.0	-	-
276	bta-miR-2304	MIMAT0011816	-	-	-	4.8	4.7	4.4	-	-	-	1.0	1.0	1.0
277	bta-miR-2309	MIMAT0011821	-	-	-	4.8	-	-	-	-	-	1.0	-	-
278	bta-miR-2320-3p	MIMAT0011845	-	-	-	4.8	-	-	-	-	-	1.0	-	-
279	bta-miR-2374	MIMAT0011920	-	-	-	4.8	4.7	4.4	-	-	-	1.0	1.0	1.0
280	bta-miR-199a-5p	MIMAT0003544	-	-	-	-	18.8	8.9	-	-	-	-	4.0	2.0
281	bta-miR-187	MIMAT0009248	-	-	-	-	9.4	-	-	-	-	-	2.0	-
282	bta-miR-3431	MIMAT0017394	-	-	-	-	9.4	-	-	-	-	-	2.0	-
283	bta-miR-11987	MIMAT0046387	-	-	-	-	4.7	-	-	-	-	-	1.0	-
284	bta-miR-200a	MIMAT0003822	-	-	-	-	4.7	-	-	-	-	-	1.0	-
285	bta-miR-2382-5p	MIMAT0011930	-	-	-	-	4.7	-	-	-	-	-	1.0	-
286	bta-miR-2881	MIMAT0013839	-	-	-	-	4.7	13.3	-	-	-	-	1.0	3.0
287	bta-miR-2888	MIMAT0013846	-	-	-	-	4.7	-	-	-	-	-	1.0	-
288	bta-miR-568	MIMAT0009350	-	-	-	-	4.7	-	-	-	-	-	1.0	-
289	bta-miR-669	MIMAT0013838	-	-	-	-	4.7	-	-	-	-	-	1.0	-
290	bta-miR-6775	MIMAT0046673	-	-	-	-	4.7	-	-	-	-	-	1.0	-
291	bta-miR-12031	MIMAT0046724	-	-	-	-	-	17.8	-	-	-	-	-	4.0
292	bta-miR-31	MIMAT0003548	-	-	-	-	-	13.3	-	-	-	-	-	3.0
293	bta-miR-599	MIMAT0009354	-	-	-	-	-	8.9	-	-	-	-	-	2.0
294	bta-miR-2284aa	MIMAT0025560	-	-	-	-	-	4.4	-	-	-	-	-	1.0
295	bta-miR-2356	MIMAT0011893	-	-	-	-	-	4.4	-	-	-	-	-	1.0
296	bta-miR-2370-3p	MIMAT0011915	-	-	-	-	-	4.4	-	-	-	-	-	1.0
297	bta-miR-2406	MIMAT0011964	-	-	-	-	-	4.4	-	-	-	-	-	1.0
298	bta-miR-382	MIMAT0009308	-	-	-	-	-	4.4	-	-	-	-	-	1.0
299	bta-miR-493	MIMAT0009333	-	-	-	-	-	4.4	-	-	-	-	-	1.0
300	bta-miR-499	MIMAT0003536	-	-	-	-	-	4.4	-	-	-	-	-	1.0
301	bta-miR-6715	MIMAT0046369	-	-	-	-	-	4.4	-	-	-	-	-	1.0

Table S2A. Gene ontology (GO) enrichment analysis based on DE miRNAs in the serum of LSDV-infected bovines compared to their expression in the non-infected control. Criteria: P < 0.05, number of genes in each GO term > 5, and fold change of Log2-treatment/control ≥ 2. (A) Biological process

No	Category	Term	Fold Enri	P-Value	Count	%	Bonferroni	Benjamin	FDR	Fisher Exact
1	GOTERM_BP_DIRECT	regulation of transcription from RNA polymerase II promoter	1.5	1.30E-38	680	10.1	1.00E-34	1.00E-34	1.00E-34	6.30E-39
2	GOTERM_BP_DIRECT	positive regulation of transcription from RNA polymerase II promoter	1.5	3.70E-23	368	5.4	3.00E-19	1.50E-19	1.50E-19	1.80E-23
3	GOTERM_BP_DIRECT	positive regulation of gene expression	1.5	7.70E-11	162	2.4	6.20E-07	2.10E-07	2.10E-07	3.60E-11
4	GOTERM_BP_DIRECT	negative regulation of transcription from RNA polymerase II promoter	1.4	1.10E-10	282	4.2	8.80E-07	2.20E-07	2.20E-07	6.40E-11
5	GOTERM_BP_DIRECT	in utero embryonic development	1.7	5.60E-10	104	1.5	4.60E-06	9.10E-07	9.00E-07	2.20E-10
6	GOTERM_BP_DIRECT	intracellular signal transduction	1.5	6.80E-10	183	2.7	5.50E-06	9.10E-07	9.00E-07	3.50E-10
7	GOTERM_BP_DIRECT	positive regulation of transcription DNA-templated	1.5	2.60E-09	158	2.3	2.10E-05	3.00E-06	3.00E-06	1.30E-09
8	GOTERM_BP_DIRECT	neuron differentiation	1.8	1.30E-08	65	1	1.00E-04	1.20E-05	1.20E-05	4.00E-09
9	GOTERM_BP_DIRECT	peptidyl-serine phosphorylation	1.7	1.30E-08	88	1.3	1.10E-04	1.20E-05	1.20E-05	5.30E-09
10	GOTERM_BP_DIRECT	nervous system development	1.7	3.10E-08	79	1.2	2.50E-04	2.50E-05	2.40E-05	1.20E-08
11	GOTERM_BP_DIRECT	negative regulation of gene expression	1.6	6.30E-08	106	1.6	5.10E-04	4.60E-05	4.60E-05	2.90E-08
12	GOTERM_BP_DIRECT	Wnt signaling pathway	1.8	7.40E-08	62	0.9	6.00E-04	4.90E-05	4.80E-05	2.50E-08
13	GOTERM_BP_DIRECT	axon guidance	1.7	7.90E-08	78	1.2	6.40E-04	4.90E-05	4.80E-05	3.10E-08
14	GOTERM_BP_DIRECT	protein phosphorylation	1.6	8.50E-08	100	1.5	6.90E-04	4.90E-05	4.80E-05	3.80E-08
15	GOTERM_BP_DIRECT	negative regulation of transcription DNA-templated	1.4	9.50E-08	148	2.2	7.60E-04	5.10E-05	5.00E-05	5.00E-08
16	GOTERM_BP_DIRECT	positive regulation of canonical Wnt signaling pathway	1.8	1.00E-07	57	0.8	8.20E-04	5.10E-05	5.10E-05	3.20E-08
17	GOTERM_BP_DIRECT	canonical Wnt signaling pathway	1.9	1.60E-07	46	0.7	1.30E-03	7.50E-05	7.40E-05	4.20E-08
18	GOTERM_BP_DIRECT	positive regulation of cell migration	1.6	2.10E-07	88	1.3	1.70E-03	9.40E-05	9.30E-05	9.10E-08
19	GOTERM_BP_DIRECT	negative regulation of canonical Wnt signaling pathway	1.7	4.70E-07	68	1	3.80E-03	2.00E-04	2.00E-04	1.80E-07
20	GOTERM_BP_DIRECT	endocytosis	1.6	7.30E-07	72	1.1	5.90E-03	2.90E-04	2.90E-04	3.00E-07
21	GOTERM_BP_DIRECT	activation of GTPase activity	1.8	9.10E-07	55	0.8	7.30E-03	3.50E-04	3.40E-04	3.10E-07
22	GOTERM_BP_DIRECT	neuron migration	1.7	9.60E-07	60	0.9	7.70E-03	3.50E-04	3.50E-04	3.50E-07
23	GOTERM_BP_DIRECT	cell fate commitment	2	1.20E-06	37	0.5	1.00E-02	4.40E-04	4.30E-04	3.10E-07
24	GOTERM_BP_DIRECT	negative regulation of cell proliferation	1.4	1.40E-06	119	1.8	1.10E-02	4.60E-04	4.50E-04	7.20E-07
25	GOTERM_BP_DIRECT	anterior/posterior pattern specification	1.8	1.90E-06	52	0.8	1.50E-02	6.00E-04	5.90E-04	6.40E-07
26	GOTERM_BP_DIRECT	regulation of ion transmembrane transport	1.7	2.10E-06	56	0.8	1.70E-02	6.60E-04	6.60E-04	7.80E-07
27	GOTERM_BP_DIRECT	protein autophosphorylation	1.6	3.70E-06	71	1	2.90E-02	1.10E-03	1.10E-03	1.60E-06
28	GOTERM_BP_DIRECT	cell migration	1.5	5.80E-06	101	1.5	4.60E-02	1.70E-03	1.60E-03	3.00E-06
29	GOTERM_BP_DIRECT	response to xenobiotic stimulus	1.9	7.50E-06	39	0.6	5.80E-02	2.10E-03	2.10E-03	2.20E-06
30	GOTERM_BP_DIRECT	protein transport	1.4	1.10E-05	129	1.9	8.30E-02	2.90E-03	2.90E-03	6.20E-06
31	GOTERM_BP_DIRECT	extracellular matrix organization	1.5	1.30E-05	75	1.1	1.00E-01	3.40E-03	3.40E-03	6.20E-06
32	GOTERM_BP_DIRECT	modulation of synaptic transmission	1.9	1.70E-05	34	0.5	1.30E-01	4.20E-03	4.20E-03	4.80E-06
33	GOTERM_BP_DIRECT	negative regulation of MAPK cascade	2.1	1.70E-05	25	0.4	1.30E-01	4.30E-03	4.20E-03	3.50E-06
34	GOTERM_BP_DIRECT	regulation of GTPase activity	1.8	1.90E-05	41	0.6	1.40E-01	4.40E-03	4.30E-03	6.30E-06
35	GOTERM_BP_DIRECT	heart looping	2	1.90E-05	30	0.4	1.40E-01	4.40E-03	4.30E-03	4.80E-06
36	GOTERM_BP_DIRECT	transforming growth factor beta receptor signaling pathway	1.7	2.60E-05	43	0.6	1.90E-01	5.80E-03	5.70E-03	9.20E-06
37	GOTERM_BP_DIRECT	negative regulation of transforming growth factor beta receptor signaling pathway	1.9	2.80E-05	35	0.5	2.00E-01	6.20E-03	6.10E-03	8.60E-06
38	GOTERM_BP_DIRECT	negative regulation of cell migration	1.7	3.50E-05	49	0.7	2.50E-01	7.50E-03	7.40E-03	1.40E-05
39	GOTERM_BP_DIRECT	neural crest cell migration	2	3.70E-05	29	0.4	2.60E-01	7.60E-03	7.50E-03	9.60E-06

40	GOTERM_BP_DIRECT	positive regulation of protein binding	1.8	3.90E-05	38	0.6	2.70E-01	7.80E-03	7.80E-03	1.30E-05
41	GOTERM_BP_DIRECT	skeletal system development	1.9	5.80E-05	32	0.5	3.80E-01	1.20E-02	1.10E-02	1.80E-05
42	GOTERM_BP_DIRECT	angiogenesis	1.5	6.60E-05	74	1.1	4.10E-01	1.30E-02	1.30E-02	3.30E-05
43	GOTERM_BP_DIRECT	learning	2.1	7.40E-05	23	0.3	4.50E-01	1.30E-02	1.30E-02	1.60E-05
44	GOTERM_BP_DIRECT	vesicle fusion	2	7.50E-05	26	0.4	4.50E-01	1.30E-02	1.30E-02	1.90E-05
45	GOTERM_BP_DIRECT	transmembrane receptor protein tyrosine kinase signaling pathway	1.6	7.50E-05	56	0.8	4.60E-01	1.30E-02	1.30E-02	3.40E-05
46	GOTERM_BP_DIRECT	positive regulation of Notch signaling pathway	2.1	7.60E-05	24	0.4	4.60E-01	1.30E-02	1.30E-02	1.70E-05
47	GOTERM_BP_DIRECT	multicellular organism growth	1.7	7.90E-05	45	0.7	4.70E-01	1.40E-02	1.30E-02	3.10E-05
48	GOTERM_BP_DIRECT	intracellular protein transport	1.3	9.90E-05	120	1.8	5.50E-01	1.70E-02	1.60E-02	6.00E-05
49	GOTERM_BP_DIRECT	positive regulation of vascular smooth muscle cell proliferation	2.4	1.10E-04	17	0.3	5.80E-01	1.80E-02	1.80E-02	1.60E-05
50	GOTERM_BP_DIRECT	positive regulation of GTPase activity	1.5	1.10E-04	64	0.9	6.00E-01	1.80E-02	1.80E-02	5.50E-05
51	GOTERM_BP_DIRECT	protein localization to plasma membrane	1.5	1.40E-04	65	1	6.60E-01	2.10E-02	2.10E-02	6.70E-05
52	GOTERM_BP_DIRECT	osteoblast differentiation	1.7	1.70E-04	37	0.5	7.50E-01	2.70E-02	2.60E-02	6.30E-05
53	GOTERM_BP_DIRECT	regulation of cell shape	1.5	2.00E-04	64	0.9	8.00E-01	3.00E-02	3.00E-02	1.00E-04
54	GOTERM_BP_DIRECT	positive regulation of angiogenesis	1.5	2.00E-04	53	0.8	8.10E-01	3.00E-02	3.00E-02	9.40E-05
55	GOTERM_BP_DIRECT	positive regulation of cardiac muscle cell proliferation	2.4	2.10E-04	15	0.2	8.10E-01	3.00E-02	3.00E-02	2.80E-05
56	GOTERM_BP_DIRECT	positive regulation of MAPK cascade	1.5	2.30E-04	55	0.8	8.40E-01	3.30E-02	3.20E-02	1.10E-04
57	GOTERM_BP_DIRECT	negative regulation of neuron apoptotic process	1.5	2.50E-04	54	0.8	8.70E-01	3.60E-02	3.50E-02	1.20E-04
58	GOTERM_BP_DIRECT	activation of cysteine-type endopeptidase activity involved in apoptotic process	1.8	2.70E-04	32	0.5	8.90E-01	3.80E-02	3.70E-02	9.40E-05
59	GOTERM_BP_DIRECT	BMP signaling pathway	1.6	2.90E-04	40	0.6	9.00E-01	3.90E-02	3.90E-02	1.20E-04
60	GOTERM_BP_DIRECT	axonogenesis	1.7	2.90E-04	36	0.5	9.00E-01	3.90E-02	3.90E-02	1.10E-04
61	GOTERM_BP_DIRECT	dorsal/ventral pattern formation	2.1	3.00E-04	19	0.3	9.10E-01	4.00E-02	3.90E-02	6.30E-05
62	GOTERM_BP_DIRECT	peptidyl-threonine phosphorylation	1.7	3.20E-04	35	0.5	9.20E-01	4.20E-02	4.10E-02	1.20E-04
63	GOTERM_BP_DIRECT	palate development	1.7	4.30E-04	32	0.5	9.70E-01	5.40E-02	5.40E-02	1.50E-04
64	GOTERM_BP_DIRECT	negative regulation of BMP signaling pathway	1.8	4.40E-04	26	0.4	9.70E-01	5.40E-02	5.40E-02	1.30E-04
65	GOTERM_BP_DIRECT	potassium ion import across plasma membrane	1.8	4.40E-04	26	0.4	9.70E-01	5.40E-02	5.40E-02	1.30E-04
66	GOTERM_BP_DIRECT	ventricular trabecula myocardium morphogenesis	2.9	4.60E-04	10	0.1	9.80E-01	5.50E-02	5.50E-02	2.30E-05
67	GOTERM_BP_DIRECT	positive regulation of cell proliferation	1.3	4.60E-04	142	2.1	9.80E-01	5.50E-02	5.50E-02	3.10E-04
68	GOTERM_BP_DIRECT	neuron projection morphogenesis	1.9	4.70E-04	25	0.4	9.80E-01	5.50E-02	5.50E-02	1.40E-04
69	GOTERM_BP_DIRECT	respiratory gaseous exchange	2.4	4.70E-04	14	0.2	9.80E-01	5.50E-02	5.50E-02	6.80E-05
70	GOTERM_BP_DIRECT	positive regulation of protein catabolic process	1.7	4.90E-04	35	0.5	9.80E-01	5.60E-02	5.60E-02	1.90E-04
71	GOTERM_BP_DIRECT	proteasome-mediated ubiquitin-dependent protein catabolic process	1.4	5.30E-04	74	1.1	9.90E-01	6.00E-02	5.90E-02	2.90E-04
72	GOTERM_BP_DIRECT	cellular response to transforming growth factor beta stimulus	2	5.80E-04	21	0.3	9.90E-01	6.50E-02	6.40E-02	1.50E-04
73	GOTERM_BP_DIRECT	blood vessel remodeling	2	6.10E-04	19	0.3	9.90E-01	6.60E-02	6.60E-02	1.40E-04
74	GOTERM_BP_DIRECT	forebrain development	2	6.10E-04	19	0.3	9.90E-01	6.60E-02	6.60E-02	1.40E-04
75	GOTERM_BP_DIRECT	central nervous system development	1.6	6.80E-04	39	0.6	1.00E+00	7.40E-02	7.30E-02	2.90E-04
76	GOTERM_BP_DIRECT	cellular calcium ion homeostasis	1.6	7.60E-04	38	0.6	1.00E+00	8.10E-02	8.00E-02	3.20E-04
77	GOTERM_BP_DIRECT	small GTPase mediated signal transduction	1.5	8.50E-04	46	0.7	1.00E+00	8.90E-02	8.80E-02	4.00E-04
78	GOTERM_BP_DIRECT	positive regulation of neuron differentiation	1.7	9.00E-04	33	0.5	1.00E+00	9.10E-02	9.10E-02	3.50E-04
79	GOTERM_BP_DIRECT	regulation of protein stability	1.7	9.00E-04	33	0.5	1.00E+00	9.10E-02	9.10E-02	3.50E-04
80	GOTERM_BP_DIRECT	blood vessel development	1.9	9.20E-04	23	0.3	1.00E+00	9.10E-02	9.10E-02	2.80E-04
81	GOTERM_BP_DIRECT	activin receptor signaling pathway	2.5	9.20E-04	12	0.2	1.00E+00	9.10E-02	9.10E-02	1.10E-04
82	GOTERM_BP_DIRECT	negative regulation of sequence-specific DNA binding transcription factor activity	1.7	9.90E-04	32	0.5	1.00E+00	9.80E-02	9.70E-02	3.90E-04

83	GOTERM_BP_DIRECT	peripheral nervous system development	2.4	1.10E-03	13	0.2	1.00E+00	1.00E-01	1.00E-01	1.60E-04
84	GOTERM_BP_DIRECT	neuron fate specification	2.4	1.10E-03	13	0.2	1.00E+00	1.00E-01	1.00E-01	1.60E-04
85	GOTERM_BP_DIRECT	cell-cell junction assembly	1.9	1.10E-03	20	0.3	1.00E+00	1.00E-01	1.00E-01	3.00E-04
86	GOTERM_BP_DIRECT	outflow tract morphogenesis	1.9	1.10E-03	20	0.3	1.00E+00	1.00E-01	1.00E-01	3.00E-04
87	GOTERM_BP_DIRECT	protein homooligomerization	1.4	1.10E-03	56	0.8	1.00E+00	1.00E-01	1.00E-01	5.90E-04
88	GOTERM_BP_DIRECT	protein localization to cell surface	2	1.20E-03	19	0.3	1.00E+00	1.00E-01	1.00E-01	3.00E-04
89	GOTERM_BP_DIRECT	protein tetramerization	2.3	1.20E-03	14	0.2	1.00E+00	1.00E-01	1.00E-01	2.10E-04
90	GOTERM_BP_DIRECT	pancreas development	2.3	1.20E-03	14	0.2	1.00E+00	1.00E-01	1.00E-01	2.10E-04
91	GOTERM_BP_DIRECT	branching involved in blood vessel morphogenesis	2	1.20E-03	18	0.3	1.00E+00	1.10E-01	1.00E-01	3.00E-04
92	GOTERM_BP_DIRECT	long-chain fatty-acyl-CoA biosynthetic process	2.9	1.20E-03	9	0.1	1.00E+00	1.10E-01	1.00E-01	6.60E-05
93	GOTERM_BP_DIRECT	endosomal transport	1.7	1.20E-03	30	0.4	1.00E+00	1.10E-01	1.00E-01	4.70E-04
94	GOTERM_BP_DIRECT	positive regulation of osteoblast differentiation	1.7	1.20E-03	30	0.4	1.00E+00	1.10E-01	1.00E-01	4.70E-04
95	GOTERM_BP_DIRECT	cellular response to insulin stimulus	1.6	1.30E-03	33	0.5	1.00E+00	1.10E-01	1.10E-01	5.40E-04
96	GOTERM_BP_DIRECT	actin cytoskeleton reorganization	1.7	1.40E-03	29	0.4	1.00E+00	1.10E-01	1.10E-01	5.20E-04
97	GOTERM_BP_DIRECT	insulin receptor signaling pathway	1.7	1.50E-03	28	0.4	1.00E+00	1.20E-01	1.20E-01	5.60E-04
98	GOTERM_BP_DIRECT	signal transduction	1.2	1.50E-03	190	2.8	1.00E+00	1.20E-01	1.20E-01	1.10E-03
99	GOTERM_BP_DIRECT	somitogenesis	1.8	1.50E-03	23	0.3	1.00E+00	1.20E-01	1.20E-01	5.00E-04
100	GOTERM_BP_DIRECT	protein polyubiquitination	1.4	1.60E-03	68	1	1.00E+00	1.30E-01	1.30E-01	9.20E-04
101	GOTERM_BP_DIRECT	positive regulation of epithelial cell proliferation	1.7	1.70E-03	27	0.4	1.00E+00	1.30E-01	1.30E-01	6.20E-04
102	GOTERM_BP_DIRECT	cellular response to glucose starvation	1.7	1.70E-03	27	0.4	1.00E+00	1.30E-01	1.30E-01	6.20E-04
103	GOTERM_BP_DIRECT	lung alveolus development	1.9	1.80E-03	21	0.3	1.00E+00	1.40E-01	1.40E-01	5.60E-04
104	GOTERM_BP_DIRECT	negative regulation of protein binding	1.6	1.80E-03	30	0.4	1.00E+00	1.40E-01	1.40E-01	7.30E-04
105	GOTERM_BP_DIRECT	regulation of transcription, DNA-templated	1.2	1.80E-03	189	2.8	1.00E+00	1.40E-01	1.40E-01	1.30E-03
106	GOTERM_BP_DIRECT	negative regulation of osteoblast differentiation	1.7	1.80E-03	26	0.4	1.00E+00	1.40E-01	1.40E-01	6.70E-04
107	GOTERM_BP_DIRECT	cell differentiation	1.2	2.10E-03	180	2.7	1.00E+00	1.60E-01	1.50E-01	1.50E-03
108	GOTERM_BP_DIRECT	embryonic forelimb morphogenesis	1.9	2.10E-03	19	0.3	1.00E+00	1.60E-01	1.50E-01	6.00E-04
109	GOTERM_BP_DIRECT	positive regulation of sequence-specific DNA binding transcription factor activity	1.5	2.10E-03	38	0.6	1.00E+00	1.60E-01	1.50E-01	9.80E-04
110	GOTERM_BP_DIRECT	miRNA mediated inhibition of translation	2.5	2.10E-03	11	0.2	1.00E+00	1.60E-01	1.50E-01	2.90E-04
111	GOTERM_BP_DIRECT	heart development	1.4	2.20E-03	59	0.9	1.00E+00	1.60E-01	1.60E-01	1.20E-03
112	GOTERM_BP_DIRECT	neuron projection development	1.5	2.20E-03	43	0.6	1.00E+00	1.60E-01	1.60E-01	1.10E-03
113	GOTERM_BP_DIRECT	embryonic organ development	2	2.30E-03	17	0.3	1.00E+00	1.70E-01	1.70E-01	6.10E-04
114	GOTERM_BP_DIRECT	embryonic skeletal system development	2	2.40E-03	16	0.2	1.00E+00	1.70E-01	1.70E-01	6.00E-04
115	GOTERM_BP_DIRECT	regulation of small GTPase mediated signal transduction	2	2.40E-03	16	0.2	1.00E+00	1.70E-01	1.70E-01	6.00E-04
116	GOTERM_BP_DIRECT	proximal/distal pattern formation	2	2.40E-03	16	0.2	1.00E+00	1.70E-01	1.70E-01	6.00E-04
117	GOTERM_BP_DIRECT	skeletal muscle cell differentiation	1.8	2.50E-03	23	0.3	1.00E+00	1.70E-01	1.70E-01	8.50E-04
118	GOTERM_BP_DIRECT	lactation	2.1	2.50E-03	15	0.2	1.00E+00	1.70E-01	1.70E-01	5.80E-04
119	GOTERM_BP_DIRECT	negative regulation of neuron differentiation	1.7	2.50E-03	27	0.4	1.00E+00	1.70E-01	1.70E-01	9.80E-04
120	GOTERM_BP_DIRECT	negative regulation of stress fiber assembly	2.1	2.50E-03	14	0.2	1.00E+00	1.70E-01	1.70E-01	5.40E-04
121	GOTERM_BP_DIRECT	regulation of protein localization	1.5	2.70E-03	36	0.5	1.00E+00	1.80E-01	1.80E-01	1.20E-03
122	GOTERM_BP_DIRECT	receptor internalization	1.8	3.00E-03	21	0.3	1.00E+00	2.00E-01	1.90E-01	9.80E-04
123	GOTERM_BP_DIRECT	cell-cell signaling	1.6	3.00E-03	29	0.4	1.00E+00	2.00E-01	1.90E-01	1.20E-03
124	GOTERM_BP_DIRECT	thymus development	1.7	3.10E-03	25	0.4	1.00E+00	2.00E-01	2.00E-01	1.20E-03
125	GOTERM_BP_DIRECT	phosphatidylinositol 3-kinase signaling	1.8	3.30E-03	20	0.3	1.00E+00	2.10E-01	2.10E-01	1.00E-03

126	GOTERM_BP_DIRECT	regulation of neurogenesis	1.8	3.30E-03	20	0.3	1.00E+00	2.10E-01	2.10E-01	1.00E-03
127	GOTERM_BP_DIRECT	lung development	1.6	3.40E-03	31	0.5	1.00E+00	2.10E-01	2.10E-01	1.50E-03
128	GOTERM_BP_DIRECT	phosphorylation	1.4	3.40E-03	57	0.8	1.00E+00	2.10E-01	2.10E-01	1.90E-03
129	GOTERM_BP_DIRECT	liver development	1.7	3.50E-03	24	0.4	1.00E+00	2.10E-01	2.10E-01	1.30E-03
130	GOTERM_BP_DIRECT	odontogenesis of dentin-containing tooth	1.7	3.50E-03	24	0.4	1.00E+00	2.10E-01	2.10E-01	1.30E-03
131	GOTERM_BP_DIRECT	positive regulation of proteasomal ubiquitin-dependent protein catabolic process	1.5	3.80E-03	33	0.5	1.00E+00	2.30E-01	2.30E-01	1.70E-03
132	GOTERM_BP_DIRECT	substrate adhesion-dependent cell spreading	1.6	3.80E-03	30	0.4	1.00E+00	2.30E-01	2.30E-01	1.60E-03
133	GOTERM_BP_DIRECT	fat cell differentiation	1.6	3.80E-03	30	0.4	1.00E+00	2.30E-01	2.30E-01	1.60E-03
134	GOTERM_BP_DIRECT	positive regulation of protein phosphorylation	1.4	3.80E-03	58	0.9	1.00E+00	2.30E-01	2.30E-01	2.20E-03
135	GOTERM_BP_DIRECT	regulation of presynapse assembly	1.9	3.80E-03	18	0.3	1.00E+00	2.30E-01	2.30E-01	1.20E-03
136	GOTERM_BP_DIRECT	hair follicle morphogenesis	1.9	4.10E-03	17	0.3	1.00E+00	2.40E-01	2.40E-01	1.20E-03
137	GOTERM_BP_DIRECT	positive regulation of epithelial cell migration	1.9	4.10E-03	17	0.3	1.00E+00	2.40E-01	2.40E-01	1.20E-03
138	GOTERM_BP_DIRECT	semaphorin-plexin signaling pathway	1.9	4.10E-03	17	0.3	1.00E+00	2.40E-01	2.40E-01	1.20E-03
139	GOTERM_BP_DIRECT	positive regulation of cell-substrate adhesion	1.9	4.10E-03	17	0.3	1.00E+00	2.40E-01	2.40E-01	1.20E-03
140	GOTERM_BP_DIRECT	morphogenesis of an epithelial sheet	2.6	4.20E-03	9	0.1	1.00E+00	2.40E-01	2.40E-01	4.60E-04
141	GOTERM_BP_DIRECT	cranial skeletal system development	2.6	4.20E-03	9	0.1	1.00E+00	2.40E-01	2.40E-01	4.60E-04
142	GOTERM_BP_DIRECT	cell-cell adhesion	1.4	4.20E-03	53	0.8	1.00E+00	2.40E-01	2.40E-01	2.40E-03
143	GOTERM_BP_DIRECT	neuronal action potential	1.9	4.40E-03	16	0.2	1.00E+00	2.50E-01	2.50E-01	1.20E-03
144	GOTERM_BP_DIRECT	animal organ morphogenesis	1.5	4.60E-03	39	0.6	1.00E+00	2.60E-01	2.60E-01	2.30E-03
145	GOTERM_BP_DIRECT	axon extension	2	4.70E-03	15	0.2	1.00E+00	2.60E-01	2.60E-01	1.20E-03
146	GOTERM_BP_DIRECT	synaptic membrane adhesion	2	4.70E-03	15	0.2	1.00E+00	2.60E-01	2.60E-01	1.20E-03
147	GOTERM_BP_DIRECT	endodermal cell differentiation	2	4.70E-03	15	0.2	1.00E+00	2.60E-01	2.60E-01	1.20E-03
148	GOTERM_BP_DIRECT	vasculogenesis	1.6	4.80E-03	28	0.4	1.00E+00	2.60E-01	2.60E-01	2.00E-03
149	GOTERM_BP_DIRECT	anatomical structure morphogenesis	1.6	4.80E-03	28	0.4	1.00E+00	2.60E-01	2.60E-01	2.00E-03
150	GOTERM_BP_DIRECT	negative regulation of epithelial cell migration	2.4	4.80E-03	10	0.1	1.00E+00	2.60E-01	2.60E-01	7.10E-04
151	GOTERM_BP_DIRECT	basement membrane organization	2.4	4.80E-03	10	0.1	1.00E+00	2.60E-01	2.60E-01	7.10E-04
152	GOTERM_BP_DIRECT	cellular response to beta-amyloid	2	5.00E-03	14	0.2	1.00E+00	2.60E-01	2.60E-01	1.20E-03
153	GOTERM_BP_DIRECT	insulin-like growth factor receptor signaling pathway	2.3	5.20E-03	11	0.2	1.00E+00	2.60E-01	2.60E-01	9.20E-04
154	GOTERM_BP_DIRECT	pathway-restricted SMAD protein phosphorylation	2.3	5.20E-03	11	0.2	1.00E+00	2.60E-01	2.60E-01	9.20E-04
155	GOTERM_BP_DIRECT	innervation	2.3	5.20E-03	11	0.2	1.00E+00	2.60E-01	2.60E-01	9.20E-04
156	GOTERM_BP_DIRECT	regulation of long-term neuronal synaptic plasticity	2.3	5.20E-03	11	0.2	1.00E+00	2.60E-01	2.60E-01	9.20E-04
157	GOTERM_BP_DIRECT	enteric nervous system development	2.3	5.20E-03	11	0.2	1.00E+00	2.60E-01	2.60E-01	9.20E-04
158	GOTERM_BP_DIRECT	positive regulation of phosphatidylinositol 3-kinase signaling	1.5	5.20E-03	33	0.5	1.00E+00	2.60E-01	2.60E-01	2.40E-03
159	GOTERM_BP_DIRECT	positive regulation of epithelial to mesenchymal transition	1.8	5.20E-03	20	0.3	1.00E+00	2.60E-01	2.60E-01	1.80E-03
160	GOTERM_BP_DIRECT	positive regulation of chondrocyte differentiation	2.2	5.20E-03	12	0.2	1.00E+00	2.60E-01	2.60E-01	1.10E-03
161	GOTERM_BP_DIRECT	ubiquitin-dependent protein catabolic process	1.3	5.40E-03	94	1.4	1.00E+00	2.70E-01	2.60E-01	3.60E-03
162	GOTERM_BP_DIRECT	negative regulation of ERK1 and ERK2 cascade	1.6	5.40E-03	27	0.4	1.00E+00	2.70E-01	2.60E-01	2.30E-03
163	GOTERM_BP_DIRECT	vesicle-mediated transport	1.3	5.40E-03	77	1.1	1.00E+00	2.70E-01	2.60E-01	3.40E-03
164	GOTERM_BP_DIRECT	cellular response to growth factor stimulus	1.8	5.80E-03	19	0.3	1.00E+00	2.80E-01	2.80E-01	1.90E-03
165	GOTERM_BP_DIRECT	visual learning	1.8	5.80E-03	19	0.3	1.00E+00	2.80E-01	2.80E-01	1.90E-03
166	GOTERM_BP_DIRECT	cell fate specification	1.8	5.80E-03	19	0.3	1.00E+00	2.80E-01	2.80E-01	1.90E-03
167	GOTERM_BP_DIRECT	chondrocyte differentiation	1.7	5.80E-03	23	0.3	1.00E+00	2.80E-01	2.80E-01	2.20E-03
168	GOTERM_BP_DIRECT	protein deubiquitination	1.5	5.80E-03	37	0.5	1.00E+00	2.80E-01	2.80E-01	2.90E-03

169	GOTERM_BP_DIRECT	positive regulation of apoptotic process	1.3	5.90E-03	88	1.3	1.00E+00	2.80E-01	2.80E-01	3.90E-03
170	GOTERM_BP_DIRECT	positive regulation of pathway-restricted SMAD protein phosphorylation	1.6	6.00E-03	26	0.4	1.00E+00	2.90E-01	2.80E-01	2.50E-03
171	GOTERM_BP_DIRECT	actin cytoskeleton organization	1.3	6.20E-03	63	0.9	1.00E+00	2.90E-01	2.90E-01	3.80E-03
172	GOTERM_BP_DIRECT	embryonic cranial skeleton morphogenesis	1.8	6.30E-03	18	0.3	1.00E+00	3.00E-01	2.90E-01	2.10E-03
173	GOTERM_BP_DIRECT	negative regulation of cell growth	1.4	6.40E-03	43	0.6	1.00E+00	3.00E-01	2.90E-01	3.40E-03
174	GOTERM_BP_DIRECT	positive regulation of protein complex assembly	1.7	6.50E-03	22	0.3	1.00E+00	3.00E-01	2.90E-01	2.50E-03
175	GOTERM_BP_DIRECT	branching involved in ureteric bud morphogenesis	1.7	6.50E-03	22	0.3	1.00E+00	3.00E-01	2.90E-01	2.50E-03
176	GOTERM_BP_DIRECT	positive regulation of pri-miRNA transcription from RNA polymerase II promoter	1.7	6.50E-03	22	0.3	1.00E+00	3.00E-01	2.90E-01	2.50E-03
177	GOTERM_BP_DIRECT	thyroid gland development	1.8	7.00E-03	17	0.3	1.00E+00	3.20E-01	3.10E-01	2.20E-03
178	GOTERM_BP_DIRECT	osteoclast differentiation	1.8	7.00E-03	17	0.3	1.00E+00	3.20E-01	3.10E-01	2.20E-03
179	GOTERM_BP_DIRECT	kidney development	1.5	7.30E-03	35	0.5	1.00E+00	3.30E-01	3.30E-01	3.70E-03
180	GOTERM_BP_DIRECT	negative regulation of I-kappaB kinase/NF-kappaB signaling	1.6	7.60E-03	24	0.4	1.00E+00	3.40E-01	3.30E-01	3.10E-03
181	GOTERM_BP_DIRECT	negative regulation of axon extension involved in axon guidance	1.9	7.60E-03	16	0.2	1.00E+00	3.40E-01	3.30E-01	2.30E-03
182	GOTERM_BP_DIRECT	cytoskeleton organization	1.5	7.60E-03	37	0.5	1.00E+00	3.40E-01	3.30E-01	3.90E-03
183	GOTERM_BP_DIRECT	positive regulation of kinase activity	1.5	7.80E-03	32	0.5	1.00E+00	3.50E-01	3.40E-01	3.80E-03
184	GOTERM_BP_DIRECT	response to osmotic stress	2.9	8.10E-03	7	0.1	1.00E+00	3.50E-01	3.50E-01	5.60E-04
185	GOTERM_BP_DIRECT	response to organic cyclic compound	2.9	8.10E-03	7	0.1	1.00E+00	3.50E-01	3.50E-01	5.60E-04
186	GOTERM_BP_DIRECT	sphingomyelin biosynthetic process	2.9	8.10E-03	7	0.1	1.00E+00	3.50E-01	3.50E-01	5.60E-04
187	GOTERM_BP_DIRECT	heparan sulfate proteoglycan biosynthetic process, polysaccharide chain biosynthetic p	2.9	8.10E-03	7	0.1	1.00E+00	3.50E-01	3.50E-01	5.60E-04
188	GOTERM_BP_DIRECT	regulation of cell migration	1.4	8.20E-03	45	0.7	1.00E+00	3.50E-01	3.50E-01	4.50E-03
189	GOTERM_BP_DIRECT	calcium-mediated signaling	1.5	8.30E-03	29	0.4	1.00E+00	3.50E-01	3.50E-01	3.80E-03
190	GOTERM_BP_DIRECT	long-term memory	1.9	8.30E-03	15	0.2	1.00E+00	3.50E-01	3.50E-01	2.40E-03
191	GOTERM_BP_DIRECT	regulation of synaptic transmission, glutamatergic	1.9	8.30E-03	15	0.2	1.00E+00	3.50E-01	3.50E-01	2.40E-03
192	GOTERM_BP_DIRECT	neural tube closure	1.5	8.80E-03	31	0.5	1.00E+00	3.70E-01	3.60E-01	4.20E-03
193	GOTERM_BP_DIRECT	regulation of heart rate by cardiac conduction	1.7	9.00E-03	19	0.3	1.00E+00	3.70E-01	3.60E-01	3.30E-03
194	GOTERM_BP_DIRECT	response to wounding	1.7	9.00E-03	19	0.3	1.00E+00	3.70E-01	3.60E-01	3.30E-03
195	GOTERM_BP_DIRECT	G1/S transition of mitotic cell cycle	1.7	9.00E-03	19	0.3	1.00E+00	3.70E-01	3.60E-01	3.30E-03
196	GOTERM_BP_DIRECT	adenylate cyclase-inhibiting G-protein coupled receptor signaling pathway	1.7	9.00E-03	19	0.3	1.00E+00	3.70E-01	3.60E-01	3.30E-03
197	GOTERM_BP_DIRECT	epithelial to mesenchymal transition	1.7	9.00E-03	19	0.3	1.00E+00	3.70E-01	3.60E-01	3.30E-03
198	GOTERM_BP_DIRECT	glucose homeostasis	1.4	9.20E-03	46	0.7	1.00E+00	3.70E-01	3.70E-01	5.20E-03
199	GOTERM_BP_DIRECT	protein dephosphorylation	1.5	9.30E-03	33	0.5	1.00E+00	3.80E-01	3.70E-01	4.60E-03
200	GOTERM_BP_DIRECT	cellular response to xenobiotic stimulus	1.6	9.50E-03	22	0.3	1.00E+00	3.80E-01	3.80E-01	3.80E-03
201	GOTERM_BP_DIRECT	positive regulation of neuroblast proliferation	2	9.60E-03	13	0.2	1.00E+00	3.80E-01	3.80E-01	2.50E-03
202	GOTERM_BP_DIRECT	regulation of axonogenesis	2	9.60E-03	13	0.2	1.00E+00	3.80E-01	3.80E-01	2.50E-03
203	GOTERM_BP_DIRECT	outflow tract septum morphogenesis	2	9.60E-03	13	0.2	1.00E+00	3.80E-01	3.80E-01	2.50E-03
204	GOTERM_BP_DIRECT	regulation of cell-matrix adhesion	2.6	9.90E-03	8	0.1	1.00E+00	3.90E-01	3.80E-01	1.20E-03
205	GOTERM_BP_DIRECT	heparin biosynthetic process	2.6	9.90E-03	8	0.1	1.00E+00	3.90E-01	3.80E-01	1.20E-03
206	GOTERM_BP_DIRECT	positive regulation of Arp2/3 complex-mediated actin nucleation	2.6	9.90E-03	8	0.1	1.00E+00	3.90E-01	3.80E-01	1.20E-03
207	GOTERM_BP_DIRECT	cardiac epithelial to mesenchymal transition	2.6	9.90E-03	8	0.1	1.00E+00	3.90E-01	3.80E-01	1.20E-03
208	GOTERM_BP_DIRECT	skin morphogenesis	2.6	9.90E-03	8	0.1	1.00E+00	3.90E-01	3.80E-01	1.20E-03
209	GOTERM_BP_DIRECT	phosphatidylinositol dephosphorylation	1.7	1.00E-02	18	0.3	1.00E+00	3.90E-01	3.80E-01	3.50E-03
210	GOTERM_BP_DIRECT	positive regulation of filopodium assembly	2.1	1.00E-02	12	0.2	1.00E+00	3.90E-01	3.80E-01	2.50E-03
211	GOTERM_BP_DIRECT	focal adhesion assembly	2.1	1.00E-02	12	0.2	1.00E+00	3.90E-01	3.80E-01	2.50E-03

212	GOTERM_BP_DIRECT	endothelial cell proliferation	2.1	1.00E-02	12	0.2	1.00E+00	3.90E-01	3.80E-01	2.50E-03
213	GOTERM_BP_DIRECT	post-anal tail morphogenesis	2.1	1.00E-02	12	0.2	1.00E+00	3.90E-01	3.80E-01	2.50E-03
214	GOTERM_BP_DIRECT	cell cycle	1.3	1.00E-02	82	1.2	1.00E+00	3.90E-01	3.90E-01	6.90E-03
215	GOTERM_BP_DIRECT	adenylate cyclase-modulating G-protein coupled receptor signaling pathway	1.7	1.10E-02	21	0.3	1.00E+00	4.00E-01	4.00E-01	4.30E-03
216	GOTERM_BP_DIRECT	male genitalia development	2.1	1.10E-02	11	0.2	1.00E+00	4.00E-01	4.00E-01	2.40E-03
217	GOTERM_BP_DIRECT	collagen fibril organization	1.6	1.10E-02	24	0.4	1.00E+00	4.00E-01	4.00E-01	4.70E-03
218	GOTERM_BP_DIRECT	positive regulation of branching involved in ureteric bud morphogenesis	2.2	1.10E-02	10	0.1	1.00E+00	4.00E-01	4.00E-01	2.10E-03
219	GOTERM_BP_DIRECT	embryonic digestive tract morphogenesis	2.2	1.10E-02	10	0.1	1.00E+00	4.00E-01	4.00E-01	2.10E-03
220	GOTERM_BP_DIRECT	definitive hemopoiesis	2.2	1.10E-02	10	0.1	1.00E+00	4.00E-01	4.00E-01	2.10E-03
221	GOTERM_BP_DIRECT	glial cell differentiation	2.2	1.10E-02	10	0.1	1.00E+00	4.00E-01	4.00E-01	2.10E-03
222	GOTERM_BP_DIRECT	regulation of cell cycle	1.3	1.10E-02	73	1.1	1.00E+00	4.00E-01	4.00E-01	7.30E-03
223	GOTERM_BP_DIRECT	Golgi organization	1.4	1.10E-02	42	0.6	1.00E+00	4.20E-01	4.10E-01	6.40E-03
224	GOTERM_BP_DIRECT	circadian regulation of gene expression	1.5	1.20E-02	31	0.5	1.00E+00	4.20E-01	4.20E-01	5.80E-03
225	GOTERM_BP_DIRECT	embryonic digit morphogenesis	1.5	1.20E-02	26	0.4	1.00E+00	4.20E-01	4.20E-01	5.40E-03
226	GOTERM_BP_DIRECT	neuron apoptotic process	1.6	1.20E-02	23	0.3	1.00E+00	4.30E-01	4.30E-01	5.20E-03
227	GOTERM_BP_DIRECT	inner ear morphogenesis	1.6	1.20E-02	23	0.3	1.00E+00	4.30E-01	4.30E-01	5.20E-03
228	GOTERM_BP_DIRECT	response to calcium ion	1.8	1.20E-02	16	0.2	1.00E+00	4.30E-01	4.30E-01	4.20E-03
229	GOTERM_BP_DIRECT	embryonic hindlimb morphogenesis	1.8	1.20E-02	16	0.2	1.00E+00	4.30E-01	4.30E-01	4.20E-03
230	GOTERM_BP_DIRECT	skeletal system morphogenesis	1.8	1.20E-02	16	0.2	1.00E+00	4.30E-01	4.30E-01	4.20E-03
231	GOTERM_BP_DIRECT	positive regulation of peptidyl-tyrosine phosphorylation	1.5	1.30E-02	28	0.4	1.00E+00	4.40E-01	4.40E-01	6.00E-03
232	GOTERM_BP_DIRECT	memory	1.5	1.30E-02	25	0.4	1.00E+00	4.60E-01	4.60E-01	6.00E-03
233	GOTERM_BP_DIRECT	synaptic vesicle endocytosis	1.7	1.30E-02	19	0.3	1.00E+00	4.60E-01	4.60E-01	5.20E-03
234	GOTERM_BP_DIRECT	mRNA transcription from RNA polymerase II promoter	1.7	1.30E-02	19	0.3	1.00E+00	4.60E-01	4.60E-01	5.20E-03
235	GOTERM_BP_DIRECT	synapse assembly	1.7	1.30E-02	19	0.3	1.00E+00	4.60E-01	4.60E-01	5.20E-03
236	GOTERM_BP_DIRECT	hemopoiesis	1.7	1.30E-02	19	0.3	1.00E+00	4.60E-01	4.60E-01	5.20E-03
237	GOTERM_BP_DIRECT	exocytosis	1.4	1.40E-02	38	0.6	1.00E+00	4.90E-01	4.80E-01	7.90E-03
238	GOTERM_BP_DIRECT	cellular response to hypoxia	1.5	1.50E-02	29	0.4	1.00E+00	5.00E-01	5.00E-01	7.40E-03
239	GOTERM_BP_DIRECT	neuron development	1.6	1.50E-02	24	0.4	1.00E+00	5.00E-01	5.00E-01	6.80E-03
240	GOTERM_BP_DIRECT	establishment of cell polarity	1.9	1.50E-02	14	0.2	1.00E+00	5.00E-01	5.00E-01	4.70E-03
241	GOTERM_BP_DIRECT	regulation of neuron differentiation	1.9	1.50E-02	14	0.2	1.00E+00	5.00E-01	5.00E-01	4.70E-03
242	GOTERM_BP_DIRECT	response to unfolded protein	1.9	1.50E-02	14	0.2	1.00E+00	5.00E-01	5.00E-01	4.70E-03
243	GOTERM_BP_DIRECT	cell maturation	1.7	1.50E-02	18	0.3	1.00E+00	5.00E-01	5.00E-01	5.80E-03
244	GOTERM_BP_DIRECT	negative regulation of Wnt signaling pathway	1.7	1.50E-02	18	0.3	1.00E+00	5.00E-01	5.00E-01	5.80E-03
245	GOTERM_BP_DIRECT	post-embryonic development	1.5	1.50E-02	31	0.5	1.00E+00	5.00E-01	5.00E-01	7.90E-03
246	GOTERM_BP_DIRECT	myelination	1.6	1.50E-02	21	0.3	1.00E+00	5.00E-01	5.00E-01	6.50E-03
247	GOTERM_BP_DIRECT	intrinsic apoptotic signaling pathway in response to DNA damage	1.6	1.50E-02	21	0.3	1.00E+00	5.00E-01	5.00E-01	6.50E-03
248	GOTERM_BP_DIRECT	mitotic cytokinesis	1.6	1.50E-02	21	0.3	1.00E+00	5.00E-01	5.00E-01	6.50E-03
249	GOTERM_BP_DIRECT	male gonad development	1.4	1.60E-02	35	0.5	1.00E+00	5.20E-01	5.10E-01	8.60E-03
250	GOTERM_BP_DIRECT	vesicle docking	1.9	1.70E-02	13	0.2	1.00E+00	5.40E-01	5.30E-01	5.00E-03
251	GOTERM_BP_DIRECT	ventricular septum morphogenesis	1.9	1.70E-02	13	0.2	1.00E+00	5.40E-01	5.30E-01	5.00E-03
252	GOTERM_BP_DIRECT	positive regulation of neuron projection development	1.5	1.70E-02	28	0.4	1.00E+00	5.40E-01	5.30E-01	8.30E-03
253	GOTERM_BP_DIRECT	positive regulation of MAP kinase activity	1.5	1.70E-02	28	0.4	1.00E+00	5.40E-01	5.30E-01	8.30E-03
254	GOTERM_BP_DIRECT	negative regulation of MAP kinase activity	1.6	1.70E-02	20	0.3	1.00E+00	5.50E-01	5.50E-01	7.20E-03

255	GOTERM_BP_DIRECT	chromatin organization	1.3	1.80E-02	73	1.1	1.00E+00	5.60E-01	5.50E-01	1.20E-02
256	GOTERM_BP_DIRECT	ossification	1.5	1.80E-02	25	0.4	1.00E+00	5.70E-01	5.60E-01	8.50E-03
257	GOTERM_BP_DIRECT	response to ischemia	1.9	1.80E-02	12	0.2	1.00E+00	5.70E-01	5.60E-01	5.20E-03
258	GOTERM_BP_DIRECT	N-glycan processing	1.9	1.80E-02	12	0.2	1.00E+00	5.70E-01	5.60E-01	5.20E-03
259	GOTERM_BP_DIRECT	aorta development	1.9	1.80E-02	12	0.2	1.00E+00	5.70E-01	5.60E-01	5.20E-03
260	GOTERM_BP_DIRECT	neuroblast proliferation	1.9	1.80E-02	12	0.2	1.00E+00	5.70E-01	5.60E-01	5.20E-03
261	GOTERM_BP_DIRECT	regulation of exocytosis	1.7	1.90E-02	16	0.2	1.00E+00	5.80E-01	5.70E-01	7.00E-03
262	GOTERM_BP_DIRECT	dendrite morphogenesis	1.7	1.90E-02	16	0.2	1.00E+00	5.80E-01	5.70E-01	7.00E-03
263	GOTERM_BP_DIRECT	positive regulation of cytokinesis	1.7	1.90E-02	16	0.2	1.00E+00	5.80E-01	5.70E-01	7.00E-03
264	GOTERM_BP_DIRECT	regulation of neuron projection development	1.7	1.90E-02	16	0.2	1.00E+00	5.80E-01	5.70E-01	7.00E-03
265	GOTERM_BP_DIRECT	endochondral ossification	1.7	1.90E-02	16	0.2	1.00E+00	5.80E-01	5.70E-01	7.00E-03
266	GOTERM_BP_DIRECT	intra-Golgi vesicle-mediated transport	1.7	1.90E-02	16	0.2	1.00E+00	5.80E-01	5.70E-01	7.00E-03
267	GOTERM_BP_DIRECT	positive regulation of cell adhesion	1.6	2.00E-02	19	0.3	1.00E+00	5.80E-01	5.70E-01	8.10E-03
268	GOTERM_BP_DIRECT	cellular response to DNA damage stimulus	1.3	2.00E-02	67	1	1.00E+00	5.80E-01	5.70E-01	1.30E-02
269	GOTERM_BP_DIRECT	mammary gland alveolus development	2	2.00E-02	11	0.2	1.00E+00	5.80E-01	5.70E-01	5.20E-03
270	GOTERM_BP_DIRECT	cellular response to organic substance	2	2.00E-02	11	0.2	1.00E+00	5.80E-01	5.70E-01	5.20E-03
271	GOTERM_BP_DIRECT	osteoblast development	2	2.00E-02	11	0.2	1.00E+00	5.80E-01	5.70E-01	5.20E-03
272	GOTERM_BP_DIRECT	labyrinthine layer blood vessel development	2	2.00E-02	11	0.2	1.00E+00	5.80E-01	5.70E-01	5.20E-03
273	GOTERM_BP_DIRECT	anterograde synaptic vesicle transport	2	2.00E-02	11	0.2	1.00E+00	5.80E-01	5.70E-01	5.20E-03
274	GOTERM_BP_DIRECT	cellular response to fibroblast growth factor stimulus	2	2.00E-02	11	0.2	1.00E+00	5.80E-01	5.70E-01	5.20E-03
275	GOTERM_BP_DIRECT	positive regulation of autophagy	1.4	2.00E-02	31	0.5	1.00E+00	5.80E-01	5.70E-01	1.00E-02
276	GOTERM_BP_DIRECT	transcription, DNA-templated	1.4	2.00E-02	35	0.5	1.00E+00	5.80E-01	5.70E-01	1.10E-02
277	GOTERM_BP_DIRECT	trigeminal nerve development	2.9	2.00E-02	6	0.1	1.00E+00	5.80E-01	5.70E-01	1.60E-03
278	GOTERM_BP_DIRECT	autophagosome docking	2.9	2.00E-02	6	0.1	1.00E+00	5.80E-01	5.70E-01	1.60E-03
279	GOTERM_BP_DIRECT	negative regulation of sodium ion transmembrane transporter activity	2.9	2.00E-02	6	0.1	1.00E+00	5.80E-01	5.70E-01	1.60E-03
280	GOTERM_BP_DIRECT	cellular response to follicle-stimulating hormone stimulus	2.9	2.00E-02	6	0.1	1.00E+00	5.80E-01	5.70E-01	1.60E-03
281	GOTERM_BP_DIRECT	dorsal aorta morphogenesis	2.9	2.00E-02	6	0.1	1.00E+00	5.80E-01	5.70E-01	1.60E-03
282	GOTERM_BP_DIRECT	prostate epithelial cord arborization involved in prostate glandular acinus morphogene	2.9	2.00E-02	6	0.1	1.00E+00	5.80E-01	5.70E-01	1.60E-03
283	GOTERM_BP_DIRECT	dorsal spinal cord development	2.9	2.00E-02	6	0.1	1.00E+00	5.80E-01	5.70E-01	1.60E-03
284	GOTERM_BP_DIRECT	trophoblast giant cell differentiation	2.1	2.10E-02	10	0.1	1.00E+00	5.80E-01	5.70E-01	5.20E-03
285	GOTERM_BP_DIRECT	Rab protein signal transduction	2.1	2.10E-02	10	0.1	1.00E+00	5.80E-01	5.70E-01	5.20E-03
286	GOTERM_BP_DIRECT	filopodium assembly	2.1	2.10E-02	10	0.1	1.00E+00	5.80E-01	5.70E-01	5.20E-03
287	GOTERM_BP_DIRECT	negative regulation of ossification	2.1	2.10E-02	10	0.1	1.00E+00	5.80E-01	5.70E-01	5.20E-03
288	GOTERM_BP_DIRECT	synaptic vesicle fusion to presynaptic active zone membrane	2.1	2.10E-02	10	0.1	1.00E+00	5.80E-01	5.70E-01	5.20E-03
289	GOTERM_BP_DIRECT	regulation of sodium ion transport	2.1	2.10E-02	10	0.1	1.00E+00	5.80E-01	5.70E-01	5.20E-03
290	GOTERM_BP_DIRECT	blood vessel morphogenesis	2.1	2.10E-02	10	0.1	1.00E+00	5.80E-01	5.70E-01	5.20E-03
291	GOTERM_BP_DIRECT	regulation of protein binding	2.1	2.10E-02	10	0.1	1.00E+00	5.80E-01	5.70E-01	5.20E-03
292	GOTERM_BP_DIRECT	somite development	2.1	2.10E-02	10	0.1	1.00E+00	5.80E-01	5.70E-01	5.20E-03
293	GOTERM_BP_DIRECT	autophagosome assembly	1.5	2.10E-02	26	0.4	1.00E+00	5.80E-01	5.70E-01	1.00E-02
294	GOTERM_BP_DIRECT	keratinocyte differentiation	1.6	2.20E-02	21	0.3	1.00E+00	5.80E-01	5.70E-01	9.60E-03
295	GOTERM_BP_DIRECT	homophilic cell adhesion via plasma membrane adhesion molecules	1.3	2.20E-02	51	0.8	1.00E+00	5.80E-01	5.70E-01	1.30E-02
296	GOTERM_BP_DIRECT	T cell activation	1.6	2.20E-02	18	0.3	1.00E+00	5.80E-01	5.70E-01	9.00E-03
297	GOTERM_BP_DIRECT	gene expression	1.4	2.20E-02	30	0.4	1.00E+00	5.80E-01	5.70E-01	1.20E-02

298	GOTERM_BP_DIRECT	negative regulation of inflammatory response	1.4	2.30E-02	36	0.5	1.00E+00	5.80E-01	5.70E-01	1.30E-02
299	GOTERM_BP_DIRECT	dendritic spine morphogenesis	2.2	2.30E-02	9	0.1	1.00E+00	5.80E-01	5.70E-01	4.80E-03
300	GOTERM_BP_DIRECT	negative regulation of epidermal growth factor-activated receptor activity	2.2	2.30E-02	9	0.1	1.00E+00	5.80E-01	5.70E-01	4.80E-03
301	GOTERM_BP_DIRECT	chondrocyte proliferation	2.2	2.30E-02	9	0.1	1.00E+00	5.80E-01	5.70E-01	4.80E-03
302	GOTERM_BP_DIRECT	negative regulation of wound healing	2.2	2.30E-02	9	0.1	1.00E+00	5.80E-01	5.70E-01	4.80E-03
303	GOTERM_BP_DIRECT	inhibitory synapse assembly	2.2	2.30E-02	9	0.1	1.00E+00	5.80E-01	5.70E-01	4.80E-03
304	GOTERM_BP_DIRECT	epithelial cell morphogenesis	2.2	2.30E-02	9	0.1	1.00E+00	5.80E-01	5.70E-01	4.80E-03
305	GOTERM_BP_DIRECT	type B pancreatic cell development	2.2	2.30E-02	9	0.1	1.00E+00	5.80E-01	5.70E-01	4.80E-03
306	GOTERM_BP_DIRECT	negative regulation of cell migration involved in sprouting angiogenesis	2.2	2.30E-02	9	0.1	1.00E+00	5.80E-01	5.70E-01	4.80E-03
307	GOTERM_BP_DIRECT	nuclear envelope reassembly	2.2	2.30E-02	9	0.1	1.00E+00	5.80E-01	5.70E-01	4.80E-03
308	GOTERM_BP_DIRECT	positive regulation of DNA-templated transcription, elongation	2.2	2.30E-02	9	0.1	1.00E+00	5.80E-01	5.70E-01	4.80E-03
309	GOTERM_BP_DIRECT	vagina development	2.5	2.30E-02	7	0.1	1.00E+00	5.80E-01	5.70E-01	3.20E-03
310	GOTERM_BP_DIRECT	embryonic viscerocranium morphogenesis	2.5	2.30E-02	7	0.1	1.00E+00	5.80E-01	5.70E-01	3.20E-03
311	GOTERM_BP_DIRECT	sympathetic ganglion development	2.5	2.30E-02	7	0.1	1.00E+00	5.80E-01	5.70E-01	3.20E-03
312	GOTERM_BP_DIRECT	fatty acid elongation, saturated fatty acid	2.5	2.30E-02	7	0.1	1.00E+00	5.80E-01	5.70E-01	3.20E-03
313	GOTERM_BP_DIRECT	endoderm formation	2.5	2.30E-02	7	0.1	1.00E+00	5.80E-01	5.70E-01	3.20E-03
314	GOTERM_BP_DIRECT	mesoderm development	2.5	2.30E-02	7	0.1	1.00E+00	5.80E-01	5.70E-01	3.20E-03
315	GOTERM_BP_DIRECT	positive regulation of phospholipase C activity	2.5	2.30E-02	7	0.1	1.00E+00	5.80E-01	5.70E-01	3.20E-03
316	GOTERM_BP_DIRECT	fatty acid elongation, monounsaturated fatty acid	2.5	2.30E-02	7	0.1	1.00E+00	5.80E-01	5.70E-01	3.20E-03
317	GOTERM_BP_DIRECT	nerve development	2.3	2.30E-02	8	0.1	1.00E+00	5.80E-01	5.70E-01	4.20E-03
318	GOTERM_BP_DIRECT	peptidyl-serine dephosphorylation	2.3	2.30E-02	8	0.1	1.00E+00	5.80E-01	5.70E-01	4.20E-03
319	GOTERM_BP_DIRECT	cellular response to leucine	2.3	2.30E-02	8	0.1	1.00E+00	5.80E-01	5.70E-01	4.20E-03
320	GOTERM_BP_DIRECT	digestive tract morphogenesis	2.3	2.30E-02	8	0.1	1.00E+00	5.80E-01	5.70E-01	4.20E-03
321	GOTERM_BP_DIRECT	DNA damage response, signal transduction by p53 class mediator resulting in transcri	2.3	2.30E-02	8	0.1	1.00E+00	5.80E-01	5.70E-01	4.20E-03
322	GOTERM_BP_DIRECT	detection of calcium ion	2.3	2.30E-02	8	0.1	1.00E+00	5.80E-01	5.70E-01	4.20E-03
323	GOTERM_BP_DIRECT	positive regulation of calcium ion-dependent exocytosis	2.3	2.30E-02	8	0.1	1.00E+00	5.80E-01	5.70E-01	4.20E-03
324	GOTERM_BP_DIRECT	spinal cord motor neuron differentiation	2.3	2.30E-02	8	0.1	1.00E+00	5.80E-01	5.70E-01	4.20E-03
325	GOTERM_BP_DIRECT	protein ubiquitination	1.2	2.30E-02	129	1.9	1.00E+00	5.80E-01	5.70E-01	1.80E-02
326	GOTERM_BP_DIRECT	embryonic pattern specification	1.8	2.40E-02	14	0.2	1.00E+00	5.90E-01	5.90E-01	8.30E-03
327	GOTERM_BP_DIRECT	positive regulation of neuron apoptotic process	1.8	2.40E-02	14	0.2	1.00E+00	5.90E-01	5.90E-01	8.30E-03
328	GOTERM_BP_DIRECT	brain development	1.3	2.40E-02	55	0.8	1.00E+00	5.90E-01	5.90E-01	1.60E-02
329	GOTERM_BP_DIRECT	regulation of cell adhesion	1.6	2.40E-02	20	0.3	1.00E+00	6.00E-01	5.90E-01	1.10E-02
330	GOTERM_BP_DIRECT	regulation of microtubule cytoskeleton organization	1.6	2.50E-02	17	0.3	1.00E+00	6.10E-01	6.00E-01	1.00E-02
331	GOTERM_BP_DIRECT	protein monoubiquitination	1.6	2.50E-02	17	0.3	1.00E+00	6.10E-01	6.00E-01	1.00E-02
332	GOTERM_BP_DIRECT	potassium ion transmembrane transport	1.3	2.70E-02	42	0.6	1.00E+00	6.40E-01	6.40E-01	1.60E-02
333	GOTERM_BP_DIRECT	pattern specification process	1.8	2.70E-02	13	0.2	1.00E+00	6.40E-01	6.40E-01	9.00E-03
334	GOTERM_BP_DIRECT	negative regulation of Ras protein signal transduction	1.8	2.70E-02	13	0.2	1.00E+00	6.40E-01	6.40E-01	9.00E-03
335	GOTERM_BP_DIRECT	positive regulation of lamellipodium assembly	1.8	2.70E-02	13	0.2	1.00E+00	6.40E-01	6.40E-01	9.00E-03
336	GOTERM_BP_DIRECT	negative regulation of GTPase activity	1.8	2.70E-02	13	0.2	1.00E+00	6.40E-01	6.40E-01	9.00E-03
337	GOTERM_BP_DIRECT	retina layer formation	1.8	2.70E-02	13	0.2	1.00E+00	6.40E-01	6.40E-01	9.00E-03
338	GOTERM_BP_DIRECT	positive regulation of fibroblast proliferation	1.6	2.80E-02	19	0.3	1.00E+00	6.60E-01	6.50E-01	1.20E-02
339	GOTERM_BP_DIRECT	anatomical structure development	1.5	2.80E-02	26	0.4	1.00E+00	6.60E-01	6.60E-01	1.40E-02
340	GOTERM_BP_DIRECT	positive regulation of protein localization to nucleus	1.7	2.80E-02	16	0.2	1.00E+00	6.60E-01	6.60E-01	1.10E-02

341	GOTERM_BP_DIRECT	regulation of synaptic vesicle exocytosis	1.7	2.80E-02	16	0.2	1.00E+00	6.60E-01	6.60E-01	1.10E-02
342	GOTERM_BP_DIRECT	positive regulation of mitotic cell cycle	1.7	2.80E-02	16	0.2	1.00E+00	6.60E-01	6.60E-01	1.10E-02
343	GOTERM_BP_DIRECT	extrinsic apoptotic signaling pathway in absence of ligand	1.7	2.80E-02	16	0.2	1.00E+00	6.60E-01	6.60E-01	1.10E-02
344	GOTERM_BP_DIRECT	sprouting angiogenesis	1.7	2.80E-02	16	0.2	1.00E+00	6.60E-01	6.60E-01	1.10E-02
345	GOTERM_BP_DIRECT	protein glycosylation	1.3	2.80E-02	50	0.7	1.00E+00	6.70E-01	6.60E-01	1.80E-02
346	GOTERM_BP_DIRECT	locomotory behavior	1.4	2.90E-02	30	0.4	1.00E+00	6.70E-01	6.60E-01	1.50E-02
347	GOTERM_BP_DIRECT	regulation of signal transduction	1.5	3.00E-02	21	0.3	1.00E+00	6.90E-01	6.80E-01	1.40E-02
348	GOTERM_BP_DIRECT	limb development	1.8	3.00E-02	12	0.2	1.00E+00	7.00E-01	6.90E-01	9.70E-03
349	GOTERM_BP_DIRECT	neurotransmitter transport	1.8	3.00E-02	12	0.2	1.00E+00	7.00E-01	6.90E-01	9.70E-03
350	GOTERM_BP_DIRECT	apoptotic process	1.2	3.10E-02	104	1.5	1.00E+00	7.10E-01	7.00E-01	2.30E-02
351	GOTERM_BP_DIRECT	embryonic limb morphogenesis	1.7	3.20E-02	15	0.2	1.00E+00	7.30E-01	7.20E-01	1.20E-02
352	GOTERM_BP_DIRECT	positive regulation of axonogenesis	1.7	3.20E-02	15	0.2	1.00E+00	7.30E-01	7.20E-01	1.20E-02
353	GOTERM_BP_DIRECT	negative regulation of TOR signaling	1.7	3.20E-02	15	0.2	1.00E+00	7.30E-01	7.20E-01	1.20E-02
354	GOTERM_BP_DIRECT	establishment of localization in cell	1.7	3.20E-02	15	0.2	1.00E+00	7.30E-01	7.20E-01	1.20E-02
355	GOTERM_BP_DIRECT	positive regulation of cell growth	1.4	3.20E-02	27	0.4	1.00E+00	7.30E-01	7.20E-01	1.70E-02
356	GOTERM_BP_DIRECT	positive regulation of tumor necrosis factor production	1.4	3.20E-02	31	0.5	1.00E+00	7.30E-01	7.20E-01	1.80E-02
357	GOTERM_BP_DIRECT	cellular response to starvation	1.4	3.20E-02	29	0.4	1.00E+00	7.30E-01	7.20E-01	1.70E-02
358	GOTERM_BP_DIRECT	actin filament organization	1.3	3.30E-02	55	0.8	1.00E+00	7.40E-01	7.40E-01	2.20E-02
359	GOTERM_BP_DIRECT	regulation of protein localization to plasma membrane	1.9	3.40E-02	11	0.2	1.00E+00	7.40E-01	7.40E-01	1.00E-02
360	GOTERM_BP_DIRECT	regulation of ARF protein signal transduction	1.9	3.40E-02	11	0.2	1.00E+00	7.40E-01	7.40E-01	1.00E-02
361	GOTERM_BP_DIRECT	negative regulation of pri-miRNA transcription from RNA polymerase II promoter	1.9	3.40E-02	11	0.2	1.00E+00	7.40E-01	7.40E-01	1.00E-02
362	GOTERM_BP_DIRECT	positive regulation of mesenchymal cell proliferation	1.9	3.40E-02	11	0.2	1.00E+00	7.40E-01	7.40E-01	1.00E-02
363	GOTERM_BP_DIRECT	intrinsic apoptotic signaling pathway by p53 class mediator	1.9	3.40E-02	11	0.2	1.00E+00	7.40E-01	7.40E-01	1.00E-02
364	GOTERM_BP_DIRECT	cell proliferation	1.3	3.40E-02	45	0.7	1.00E+00	7.40E-01	7.40E-01	2.10E-02
365	GOTERM_BP_DIRECT	camera-type eye development	1.6	3.60E-02	17	0.3	1.00E+00	7.80E-01	7.70E-01	1.50E-02
366	GOTERM_BP_DIRECT	microtubule bundle formation	1.6	3.60E-02	17	0.3	1.00E+00	7.80E-01	7.70E-01	1.50E-02
367	GOTERM_BP_DIRECT	long-term synaptic potentiation	1.6	3.60E-02	17	0.3	1.00E+00	7.80E-01	7.70E-01	1.50E-02
368	GOTERM_BP_DIRECT	eye development	1.6	3.60E-02	17	0.3	1.00E+00	7.80E-01	7.70E-01	1.50E-02
369	GOTERM_BP_DIRECT	protein import into nucleus	1.3	3.70E-02	39	0.6	1.00E+00	7.90E-01	7.80E-01	2.20E-02
370	GOTERM_BP_DIRECT	cell adhesion	1.2	3.70E-02	117	1.7	1.00E+00	7.90E-01	7.80E-01	2.80E-02
371	GOTERM_BP_DIRECT	positive regulation of protein autophosphorylation	1.9	3.70E-02	10	0.1	1.00E+00	7.90E-01	7.80E-01	1.10E-02
372	GOTERM_BP_DIRECT	potassium ion transport	1.9	3.70E-02	10	0.1	1.00E+00	7.90E-01	7.80E-01	1.10E-02
373	GOTERM_BP_DIRECT	TOR signaling	1.9	3.70E-02	10	0.1	1.00E+00	7.90E-01	7.80E-01	1.10E-02
374	GOTERM_BP_DIRECT	positive regulation of ubiquitin-protein transferase activity	1.9	3.70E-02	10	0.1	1.00E+00	7.90E-01	7.80E-01	1.10E-02
375	GOTERM_BP_DIRECT	late endosome to lysosome transport	1.9	3.70E-02	10	0.1	1.00E+00	7.90E-01	7.80E-01	1.10E-02
376	GOTERM_BP_DIRECT	pigmentation	1.9	3.70E-02	10	0.1	1.00E+00	7.90E-01	7.80E-01	1.10E-02
377	GOTERM_BP_DIRECT	zinc II ion transmembrane transport	1.9	3.70E-02	10	0.1	1.00E+00	7.90E-01	7.80E-01	1.10E-02
378	GOTERM_BP_DIRECT	oligodendrocyte development	1.9	3.70E-02	10	0.1	1.00E+00	7.90E-01	7.80E-01	1.10E-02
379	GOTERM_BP_DIRECT	dendrite self-avoidance	1.9	3.70E-02	10	0.1	1.00E+00	7.90E-01	7.80E-01	1.10E-02
380	GOTERM_BP_DIRECT	regulation of dendrite morphogenesis	1.9	3.70E-02	10	0.1	1.00E+00	7.90E-01	7.80E-01	1.10E-02
381	GOTERM_BP_DIRECT	positive regulation of protein export from nucleus	1.9	3.70E-02	10	0.1	1.00E+00	7.90E-01	7.80E-01	1.10E-02
382	GOTERM_BP_DIRECT	execution phase of apoptosis	1.9	3.70E-02	10	0.1	1.00E+00	7.90E-01	7.80E-01	1.10E-02
383	GOTERM_BP_DIRECT	protein stabilization	1.2	3.80E-02	63	0.9	1.00E+00	8.00E-01	7.90E-01	2.60E-02

384	GOTERM_BP_DIRECT	positive regulation of peptidyl-serine phosphorylation	1.3	3.80E-02	35	0.5	1.00E+00	8.10E-01	8.00E-01	2.30E-02
385	GOTERM_BP_DIRECT	positive regulation of actin filament polymerization	1.5	3.90E-02	21	0.3	1.00E+00	8.30E-01	8.20E-01	1.90E-02
386	GOTERM_BP_DIRECT	negative regulation of protein kinase activity	1.5	4.00E-02	23	0.3	1.00E+00	8.30E-01	8.20E-01	2.00E-02
387	GOTERM_BP_DIRECT	negative regulation of epithelial cell proliferation	1.5	4.00E-02	23	0.3	1.00E+00	8.30E-01	8.20E-01	2.00E-02
388	GOTERM_BP_DIRECT	negative regulation of protein ubiquitination	1.5	4.00E-02	23	0.3	1.00E+00	8.30E-01	8.20E-01	2.00E-02
389	GOTERM_BP_DIRECT	translational initiation	1.6	4.00E-02	16	0.2	1.00E+00	8.30E-01	8.20E-01	1.70E-02
390	GOTERM_BP_DIRECT	SMAD protein signal transduction	1.4	4.00E-02	29	0.4	1.00E+00	8.30E-01	8.20E-01	2.20E-02
391	GOTERM_BP_DIRECT	response to hypoxia	1.3	4.10E-02	38	0.6	1.00E+00	8.30E-01	8.20E-01	2.50E-02
392	GOTERM_BP_DIRECT	regulation of heart rate	1.7	4.10E-02	13	0.2	1.00E+00	8.30E-01	8.20E-01	1.50E-02
393	GOTERM_BP_DIRECT	positive regulation of focal adhesion assembly	1.7	4.10E-02	13	0.2	1.00E+00	8.30E-01	8.20E-01	1.50E-02
394	GOTERM_BP_DIRECT	negative regulation of anoikis	2	4.10E-02	9	0.1	1.00E+00	8.30E-01	8.20E-01	1.10E-02
395	GOTERM_BP_DIRECT	stem cell proliferation	2	4.10E-02	9	0.1	1.00E+00	8.30E-01	8.20E-01	1.10E-02
396	GOTERM_BP_DIRECT	regulation of transcription involved in G1/S transition of mitotic cell cycle	2	4.10E-02	9	0.1	1.00E+00	8.30E-01	8.20E-01	1.10E-02
397	GOTERM_BP_DIRECT	embryonic digestive tract development	2	4.10E-02	9	0.1	1.00E+00	8.30E-01	8.20E-01	1.10E-02
398	GOTERM_BP_DIRECT	layer formation in cerebral cortex	2	4.10E-02	9	0.1	1.00E+00	8.30E-01	8.20E-01	1.10E-02
399	GOTERM_BP_DIRECT	regulation of insulin secretion involved in cellular response to glucose stimulus	2	4.10E-02	9	0.1	1.00E+00	8.30E-01	8.20E-01	1.10E-02
400	GOTERM_BP_DIRECT	chondrocyte development	2	4.10E-02	9	0.1	1.00E+00	8.30E-01	8.20E-01	1.10E-02
401	GOTERM_BP_DIRECT	positive regulation of RNA splicing	2	4.10E-02	9	0.1	1.00E+00	8.30E-01	8.20E-01	1.10E-02
402	GOTERM_BP_DIRECT	positive regulation of translational initiation	2	4.10E-02	9	0.1	1.00E+00	8.30E-01	8.20E-01	1.10E-02
403	GOTERM_BP_DIRECT	regulation of neuron death	2	4.10E-02	9	0.1	1.00E+00	8.30E-01	8.20E-01	1.10E-02
404	GOTERM_BP_DIRECT	apoptotic signaling pathway	1.5	4.30E-02	18	0.3	1.00E+00	8.50E-01	8.40E-01	2.00E-02
405	GOTERM_BP_DIRECT	histone H4 acetylation	1.5	4.30E-02	18	0.3	1.00E+00	8.50E-01	8.40E-01	2.00E-02
406	GOTERM_BP_DIRECT	positive regulation of bone mineralization	1.5	4.30E-02	18	0.3	1.00E+00	8.50E-01	8.40E-01	2.00E-02
407	GOTERM_BP_DIRECT	cellular response to virus	1.5	4.30E-02	18	0.3	1.00E+00	8.50E-01	8.40E-01	2.00E-02
408	GOTERM_BP_DIRECT	positive regulation of fat cell differentiation	1.5	4.50E-02	20	0.3	1.00E+00	8.50E-01	8.40E-01	2.20E-02
409	GOTERM_BP_DIRECT	cellular response to UV	1.5	4.50E-02	20	0.3	1.00E+00	8.50E-01	8.40E-01	2.20E-02
410	GOTERM_BP_DIRECT	Ras protein signal transduction	1.5	4.50E-02	20	0.3	1.00E+00	8.50E-01	8.40E-01	2.20E-02
411	GOTERM_BP_DIRECT	regulation of epithelial to mesenchymal transition	2.1	4.50E-02	8	0.1	1.00E+00	8.50E-01	8.40E-01	1.10E-02
412	GOTERM_BP_DIRECT	embryonic heart tube development	2.1	4.50E-02	8	0.1	1.00E+00	8.50E-01	8.40E-01	1.10E-02
413	GOTERM_BP_DIRECT	aorta morphogenesis	2.1	4.50E-02	8	0.1	1.00E+00	8.50E-01	8.40E-01	1.10E-02
414	GOTERM_BP_DIRECT	cellular macromolecular complex assembly	2.1	4.50E-02	8	0.1	1.00E+00	8.50E-01	8.40E-01	1.10E-02
415	GOTERM_BP_DIRECT	peptidyl-threonine dephosphorylation	2.1	4.50E-02	8	0.1	1.00E+00	8.50E-01	8.40E-01	1.10E-02
416	GOTERM_BP_DIRECT	positive regulation of vascular endothelial cell proliferation	2.1	4.50E-02	8	0.1	1.00E+00	8.50E-01	8.40E-01	1.10E-02
417	GOTERM_BP_DIRECT	cellular response to fluid shear stress	2.1	4.50E-02	8	0.1	1.00E+00	8.50E-01	8.40E-01	1.10E-02
418	GOTERM_BP_DIRECT	retinal cone cell development	2.1	4.50E-02	8	0.1	1.00E+00	8.50E-01	8.40E-01	1.10E-02
419	GOTERM_BP_DIRECT	positive regulation of gene silencing by miRNA	2.1	4.50E-02	8	0.1	1.00E+00	8.50E-01	8.40E-01	1.10E-02
420	GOTERM_BP_DIRECT	cell-matrix adhesion	1.4	4.50E-02	28	0.4	1.00E+00	8.50E-01	8.40E-01	2.50E-02
421	GOTERM_BP_DIRECT	adult walking behavior	1.6	4.60E-02	15	0.2	1.00E+00	8.50E-01	8.40E-01	1.90E-02
422	GOTERM_BP_DIRECT	ureteric bud development	1.6	4.60E-02	15	0.2	1.00E+00	8.50E-01	8.40E-01	1.90E-02
423	GOTERM_BP_DIRECT	lamellipodium assembly	1.6	4.60E-02	15	0.2	1.00E+00	8.50E-01	8.40E-01	1.90E-02
424	GOTERM_BP_DIRECT	B cell homeostasis	1.7	4.60E-02	12	0.2	1.00E+00	8.50E-01	8.40E-01	1.70E-02
425	GOTERM_BP_DIRECT	metanephros development	1.7	4.60E-02	12	0.2	1.00E+00	8.50E-01	8.40E-01	1.70E-02
426	GOTERM_BP_DIRECT	protein K48-linked deubiquitination	1.7	4.60E-02	12	0.2	1.00E+00	8.50E-01	8.40E-01	1.70E-02

427	GOTERM_BP_DIRECT	positive regulation of phosphatidylinositol 3-kinase activity	1.7	4.60E-02	12	0.2	1.00E+00	8.50E-01	8.40E-01	1.70E-02
428	GOTERM_BP_DIRECT	negative regulation of osteoclast differentiation	1.7	4.60E-02	12	0.2	1.00E+00	8.50E-01	8.40E-01	1.70E-02
429	GOTERM_BP_DIRECT	regulation of bone mineralization	1.7	4.60E-02	12	0.2	1.00E+00	8.50E-01	8.40E-01	1.70E-02
430	GOTERM_BP_DIRECT	multicellular organism aging	1.7	4.60E-02	12	0.2	1.00E+00	8.50E-01	8.40E-01	1.70E-02
431	GOTERM_BP_DIRECT	cellular component organization	2.3	4.90E-02	7	0.1	1.00E+00	8.50E-01	8.40E-01	9.90E-03
432	GOTERM_BP_DIRECT	positive regulation of extrinsic apoptotic signaling pathway via death domain receptors	2.3	4.90E-02	7	0.1	1.00E+00	8.50E-01	8.40E-01	9.90E-03
433	GOTERM_BP_DIRECT	cardiac left ventricle morphogenesis	2.3	4.90E-02	7	0.1	1.00E+00	8.50E-01	8.40E-01	9.90E-03
434	GOTERM_BP_DIRECT	lens fiber cell differentiation	2.3	4.90E-02	7	0.1	1.00E+00	8.50E-01	8.40E-01	9.90E-03
435	GOTERM_BP_DIRECT	mesodermal cell differentiation	2.3	4.90E-02	7	0.1	1.00E+00	8.50E-01	8.40E-01	9.90E-03
436	GOTERM_BP_DIRECT	negative regulation of cardiac muscle hypertrophy	2.3	4.90E-02	7	0.1	1.00E+00	8.50E-01	8.40E-01	9.90E-03
437	GOTERM_BP_DIRECT	negative regulation of production of miRNAs involved in gene silencing by miRNA	2.3	4.90E-02	7	0.1	1.00E+00	8.50E-01	8.40E-01	9.90E-03
438	GOTERM_BP_DIRECT	negative regulation of sprouting angiogenesis	2.3	4.90E-02	7	0.1	1.00E+00	8.50E-01	8.40E-01	9.90E-03
439	GOTERM_BP_DIRECT	embryonic eye morphogenesis	2.3	4.90E-02	7	0.1	1.00E+00	8.50E-01	8.40E-01	9.90E-03
440	GOTERM_BP_DIRECT	establishment of epithelial cell polarity	2.3	4.90E-02	7	0.1	1.00E+00	8.50E-01	8.40E-01	9.90E-03
441	GOTERM_BP_DIRECT	regulation of gastrulation	2.3	4.90E-02	7	0.1	1.00E+00	8.50E-01	8.40E-01	9.90E-03
442	GOTERM_BP_DIRECT	craniofacial suture morphogenesis	2.3	4.90E-02	7	0.1	1.00E+00	8.50E-01	8.40E-01	9.90E-03
443	GOTERM_BP_DIRECT	fibroblast proliferation	2.3	4.90E-02	7	0.1	1.00E+00	8.50E-01	8.40E-01	9.90E-03
444	GOTERM_BP_DIRECT	vocalization behavior	2.3	4.90E-02	7	0.1	1.00E+00	8.50E-01	8.40E-01	9.90E-03
445	GOTERM_BP_DIRECT	regulation of potassium ion transmembrane transport	2.3	4.90E-02	7	0.1	1.00E+00	8.50E-01	8.40E-01	9.90E-03
446	GOTERM_BP_DIRECT	positive regulation of excitatory postsynaptic potential	2.3	4.90E-02	7	0.1	1.00E+00	8.50E-01	8.40E-01	9.90E-03
447	GOTERM_BP_DIRECT	renal sodium ion absorption	2.3	4.90E-02	7	0.1	1.00E+00	8.50E-01	8.40E-01	9.90E-03
448	GOTERM_BP_DIRECT	protein K11-linked deubiquitination	2.3	4.90E-02	7	0.1	1.00E+00	8.50E-01	8.40E-01	9.90E-03
449	GOTERM_BP_DIRECT	nerve growth factor signaling pathway	2.3	4.90E-02	7	0.1	1.00E+00	8.50E-01	8.40E-01	9.90E-03
450	GOTERM_BP_DIRECT	histone H3 acetylation	1.5	4.90E-02	17	0.3	1.00E+00	8.50E-01	8.40E-01	2.20E-02

Table S2B. Gene ontology (GO) enrichment analysis based on DE miRNAs in the serum of LSDV-infected bovines compared to their expression in the non-infected control. Criteria: P < 0.05, number of genes in each GO term > 5, and fold change of Log2-treatment/control ≥ 2. (B) cellular component

No	Category	Term	Fold Enri	P-Value	Count	%	Bonferroni	Benjamini	FDR	Fisher Exact
1	GOTERM_CC_DIRECT	cytosol	1.4	2.50E-62	1269	18.8	3.10E-59	3.10E-59	2.80E-59	1.40E-62
2	GOTERM_CC_DIRECT	nucleus	1.4	1.50E-58	1650	24.4	1.80E-55	8.90E-56	8.20E-56	9.20E-59
3	GOTERM_CC_DIRECT	nucleoplasm	1.5	9.70E-55	970	14.3	1.20E-51	4.00E-52	3.60E-52	5.10E-55
4	GOTERM_CC_DIRECT	cytoplasm	1.3	1.60E-31	1467	21.7	2.00E-28	5.00E-29	4.60E-29	1.20E-31
5	GOTERM_CC_DIRECT	glutamatergic synapse	2.1	5.80E-23	140	2.1	7.10E-20	1.40E-20	1.30E-20	1.60E-23
6	GOTERM_CC_DIRECT	Golgi apparatus	1.5	1.30E-21	344	5.1	1.60E-18	2.70E-19	2.50E-19	6.60E-22
7	GOTERM_CC_DIRECT	membrane	1.4	3.10E-19	457	6.8	3.70E-16	5.40E-17	4.90E-17	1.80E-19
8	GOTERM_CC_DIRECT	axon	2	1.20E-17	122	1.8	1.40E-14	1.80E-15	1.60E-15	3.60E-18
9	GOTERM_CC_DIRECT	Golgi membrane	1.7	3.70E-15	170	2.5	4.60E-12	5.10E-13	4.70E-13	1.60E-15
10	GOTERM_CC_DIRECT	postsynaptic density	2.1	3.50E-13	77	1.1	4.30E-10	4.30E-11	3.90E-11	9.20E-14
11	GOTERM_CC_DIRECT	perinuclear region of cytoplasm	1.5	2.40E-11	198	2.9	2.90E-08	2.70E-09	2.40E-09	1.20E-11
12	GOTERM_CC_DIRECT	RNA polymerase II transcription factor complex	2.2	6.40E-11	57	0.8	7.80E-08	6.50E-09	6.00E-09	1.50E-11
13	GOTERM_CC_DIRECT	focal adhesion	1.9	1.30E-10	84	1.2	1.60E-07	1.20E-08	1.10E-08	4.40E-11
14	GOTERM_CC_DIRECT	endoplasmic reticulum	1.4	1.80E-10	280	4.1	2.20E-07	1.50E-08	1.40E-08	1.10E-10
15	GOTERM_CC_DIRECT	transcription factor complex	1.8	3.40E-10	92	1.4	4.20E-07	2.80E-08	2.50E-08	1.30E-10
16	GOTERM_CC_DIRECT	receptor complex	1.7	4.90E-10	98	1.4	6.00E-07	3.70E-08	3.40E-08	1.90E-10
17	GOTERM_CC_DIRECT	macromolecular complex	1.5	7.20E-10	149	2.2	8.80E-07	5.20E-08	4.70E-08	3.50E-10
18	GOTERM_CC_DIRECT	Schaffer collateral - CA1 synapse	2.4	1.30E-09	41	0.6	1.60E-06	9.00E-08	8.30E-08	2.50E-10
19	GOTERM_CC_DIRECT	cell surface	1.5	1.60E-09	169	2.5	2.00E-06	1.10E-07	9.80E-08	8.70E-10
20	GOTERM_CC_DIRECT	integral component of plasma membrane	1.3	2.20E-09	346	5.1	2.70E-06	1.40E-07	1.30E-07	1.50E-09
21	GOTERM_CC_DIRECT	neuronal cell body	1.8	3.40E-09	82	1.2	4.20E-06	2.00E-07	1.80E-07	1.30E-09
22	GOTERM_CC_DIRECT	lamellipodium	1.8	3.70E-09	73	1.1	4.50E-06	2.00E-07	1.90E-07	1.30E-09
23	GOTERM_CC_DIRECT	dendritic spine	2	8.60E-09	52	0.8	1.10E-05	4.60E-07	4.20E-07	2.40E-09
24	GOTERM_CC_DIRECT	voltage-gated potassium channel complex	2.2	2.30E-08	41	0.6	2.80E-05	1.20E-06	1.10E-06	5.40E-09
25	GOTERM_CC_DIRECT	cytoplasmic stress granule	2.1	6.20E-08	43	0.6	7.50E-05	3.00E-06	2.80E-06	1.60E-08
26	GOTERM_CC_DIRECT	basement membrane	2.1	8.00E-08	45	0.7	9.80E-05	3.80E-06	3.50E-06	2.20E-08
27	GOTERM_CC_DIRECT	basolateral plasma membrane	1.7	8.70E-08	82	1.2	1.10E-04	4.00E-06	3.60E-06	3.70E-08
28	GOTERM_CC_DIRECT	early endosome	1.6	1.10E-07	94	1.4	1.30E-04	4.70E-06	4.30E-06	4.90E-08
29	GOTERM_CC_DIRECT	nuclear body	1.5	1.30E-07	111	1.6	1.50E-04	5.30E-06	4.90E-06	6.20E-08
30	GOTERM_CC_DIRECT	chromatin	1.5	1.40E-07	123	1.8	1.70E-04	5.60E-06	5.10E-06	7.00E-08
31	GOTERM_CC_DIRECT	neuron projection	1.5	2.00E-07	110	1.6	2.40E-04	7.70E-06	7.10E-06	9.80E-08
32	GOTERM_CC_DIRECT	neuromuscular junction	2.2	2.70E-07	37	0.5	3.30E-04	1.00E-05	9.40E-06	6.50E-08
33	GOTERM_CC_DIRECT	P-body	2	3.90E-07	45	0.7	4.80E-04	1.50E-05	1.30E-05	1.20E-07
34	GOTERM_CC_DIRECT	early endosome membrane	1.9	6.90E-07	52	0.8	8.50E-04	2.50E-05	2.30E-05	2.40E-07
35	GOTERM_CC_DIRECT	cell-cell junction	1.7	9.80E-07	66	1	1.20E-03	3.40E-05	3.10E-05	4.00E-07
36	GOTERM_CC_DIRECT	extracellular matrix	1.6	1.40E-06	91	1.3	1.70E-03	4.60E-05	4.20E-05	6.60E-07
37	GOTERM_CC_DIRECT	GABA-ergic synapse	2.3	1.40E-06	29	0.4	1.70E-03	4.70E-05	4.30E-05	3.00E-07

38	GOTERM_CC_DIRECT	presynapse	1.7	2.80E-06	57	0.8	3.40E-03	8.90E-05	8.20E-05	1.10E-06
39	GOTERM_CC_DIRECT	midbody	1.6	3.50E-06	71	1	4.30E-03	1.10E-04	1.00E-04	1.60E-06
40	GOTERM_CC_DIRECT	dendrite	1.5	6.80E-06	106	1.6	8.30E-03	2.10E-04	1.90E-04	3.70E-06
41	GOTERM_CC_DIRECT	postsynaptic membrane	1.7	8.30E-06	55	0.8	1.00E-02	2.50E-04	2.30E-04	3.40E-06
42	GOTERM_CC_DIRECT	cytoplasmic vesicle membrane	1.9	8.60E-06	41	0.6	1.00E-02	2.50E-04	2.30E-04	2.90E-06
43	GOTERM_CC_DIRECT	transport vesicle	2.2	1.10E-05	28	0.4	1.40E-02	3.20E-04	3.00E-04	2.80E-06
44	GOTERM_CC_DIRECT	nuclear speck	1.4	1.70E-05	137	2	2.00E-02	4.60E-04	4.20E-04	1.00E-05
45	GOTERM_CC_DIRECT	endosome	1.5	1.70E-05	88	1.3	2.10E-02	4.60E-04	4.20E-04	9.00E-06
46	GOTERM_CC_DIRECT	adherens junction	1.7	1.70E-05	58	0.9	2.10E-02	4.60E-04	4.20E-04	7.50E-06
47	GOTERM_CC_DIRECT	synapse	1.4	2.00E-05	139	2.1	2.50E-02	5.30E-04	4.90E-04	1.30E-05
48	GOTERM_CC_DIRECT	recycling endosome membrane	2	2.10E-05	31	0.5	2.60E-02	5.40E-04	5.00E-04	6.10E-06
49	GOTERM_CC_DIRECT	synaptic vesicle membrane	1.8	2.40E-05	40	0.6	2.90E-02	6.00E-04	5.50E-04	8.60E-06
50	GOTERM_CC_DIRECT	integral component of presynaptic membrane	2.7	3.40E-05	16	0.2	4.10E-02	8.20E-04	7.60E-04	4.30E-06
51	GOTERM_CC_DIRECT	ruffle	1.9	3.40E-05	36	0.5	4.10E-02	8.20E-04	7.60E-04	1.20E-05
52	GOTERM_CC_DIRECT	presynaptic membrane	2.2	8.10E-05	23	0.3	9.40E-02	1.90E-03	1.70E-03	2.00E-05
53	GOTERM_CC_DIRECT	lateral plasma membrane	1.9	1.10E-04	30	0.4	1.20E-01	2.50E-03	2.30E-03	3.50E-05
54	GOTERM_CC_DIRECT	synaptic vesicle	1.7	1.30E-04	46	0.7	1.50E-01	2.90E-03	2.70E-03	5.60E-05
55	GOTERM_CC_DIRECT	SNARE complex	1.9	1.60E-04	28	0.4	1.80E-01	3.50E-03	3.20E-03	5.00E-05
56	GOTERM_CC_DIRECT	centrosome	1.3	1.60E-04	168	2.5	1.80E-01	3.60E-03	3.30E-03	1.10E-04
57	GOTERM_CC_DIRECT	integral component of Golgi membrane	1.9	2.00E-04	29	0.4	2.20E-01	4.40E-03	4.00E-03	6.80E-05
58	GOTERM_CC_DIRECT	postsynapse	1.8	2.10E-04	35	0.5	2.20E-01	4.40E-03	4.00E-03	7.90E-05
59	GOTERM_CC_DIRECT	late endosome	1.6	2.20E-04	48	0.7	2.30E-01	4.50E-03	4.10E-03	9.80E-05
60	GOTERM_CC_DIRECT	phagocytic vesicle	1.8	3.10E-04	31	0.5	3.20E-01	6.40E-03	5.90E-03	1.10E-04
61	GOTERM_CC_DIRECT	cytoplasmic vesicle	1.4	3.80E-04	90	1.3	3.70E-01	7.60E-03	7.00E-03	2.30E-04
62	GOTERM_CC_DIRECT	Cul3-RING ubiquitin ligase complex	2	4.90E-04	22	0.3	4.50E-01	9.70E-03	8.90E-03	1.40E-04
63	GOTERM_CC_DIRECT	presynaptic active zone membrane	3.1	5.00E-04	10	0.1	4.60E-01	9.70E-03	8.90E-03	4.00E-05
64	GOTERM_CC_DIRECT	dendrite membrane	2.5	5.10E-04	14	0.2	4.70E-01	9.80E-03	9.00E-03	8.80E-05
65	GOTERM_CC_DIRECT	filopodium	1.8	5.60E-04	30	0.4	5.00E-01	1.10E-02	9.70E-03	2.10E-04
66	GOTERM_CC_DIRECT	cytoskeleton	1.3	6.10E-04	124	1.8	5.20E-01	1.10E-02	1.00E-02	4.00E-04
67	GOTERM_CC_DIRECT	integral component of endoplasmic reticulum membrane	1.5	6.40E-04	48	0.7	5.40E-01	1.20E-02	1.10E-02	3.10E-04
68	GOTERM_CC_DIRECT	ubiquitin ligase complex	1.7	7.20E-04	34	0.5	5.90E-01	1.30E-02	1.20E-02	3.00E-04
69	GOTERM_CC_DIRECT	endomembrane system	1.5	7.70E-04	47	0.7	6.10E-01	1.40E-02	1.30E-02	3.80E-04
70	GOTERM_CC_DIRECT	membrane raft	1.5	8.40E-04	57	0.8	6.40E-01	1.50E-02	1.30E-02	4.50E-04
71	GOTERM_CC_DIRECT	phosphatidylinositol 3-kinase complex	2.3	9.30E-04	15	0.2	6.80E-01	1.60E-02	1.50E-02	2.00E-04
72	GOTERM_CC_DIRECT	extrinsic component of cytoplasmic side of plasma membrane	1.8	9.90E-04	29	0.4	7.00E-01	1.70E-02	1.50E-02	3.80E-04
73	GOTERM_CC_DIRECT	nuclear matrix	1.7	9.90E-04	34	0.5	7.00E-01	1.70E-02	1.50E-02	4.20E-04
74	GOTERM_CC_DIRECT	plasma membrane raft	2.4	1.10E-03	14	0.2	7.30E-01	1.80E-02	1.60E-02	2.10E-04
75	GOTERM_CC_DIRECT	trans-Golgi network	1.4	1.20E-03	67	1	7.60E-01	1.90E-02	1.80E-02	6.80E-04
76	GOTERM_CC_DIRECT	clathrin-coated vesicle	1.8	1.50E-03	25	0.4	8.50E-01	2.50E-02	2.30E-02	5.70E-04
77	GOTERM_CC_DIRECT	sarcolemma	1.6	1.80E-03	34	0.5	8.90E-01	2.90E-02	2.70E-02	8.20E-04
78	GOTERM_CC_DIRECT	late endosome membrane	1.5	2.00E-03	42	0.6	9.10E-01	3.10E-02	2.80E-02	9.80E-04

79	GOTERM_CC_DIRECT	endosome membrane	1.5	2.20E-03	49	0.7	9.30E-01	3.40E-02	3.10E-02	1.20E-03
80	GOTERM_CC_DIRECT	voltage-gated sodium channel complex	2.3	2.40E-03	13	0.2	9.50E-01	3.60E-02	3.30E-02	5.10E-04
81	GOTERM_CC_DIRECT	cell-cell contact zone	2.3	2.40E-03	13	0.2	9.50E-01	3.60E-02	3.30E-02	5.10E-04
82	GOTERM_CC_DIRECT	cell cortex	1.5	2.60E-03	48	0.7	9.60E-01	3.90E-02	3.60E-02	1.40E-03
83	GOTERM_CC_DIRECT	postsynaptic density membrane	2	3.40E-03	17	0.3	9.80E-01	5.00E-02	4.60E-02	1.00E-03
84	GOTERM_CC_DIRECT	activin receptor complex	3.4	3.40E-03	7	0.1	9.90E-01	5.00E-02	4.60E-02	1.90E-04
85	GOTERM_CC_DIRECT	histone methyltransferase complex	2.6	3.50E-03	10	0.1	9.90E-01	5.10E-02	4.70E-02	5.60E-04
86	GOTERM_CC_DIRECT	cell projection	1.4	3.80E-03	53	0.8	9.90E-01	5.40E-02	5.00E-02	2.20E-03
87	GOTERM_CC_DIRECT	nucleotide-activated protein kinase complex	3	3.90E-03	8	0.1	9.90E-01	5.40E-02	5.00E-02	3.80E-04
88	GOTERM_CC_DIRECT	growth cone	1.6	4.30E-03	31	0.5	1.00E+00	6.00E-02	5.50E-02	2.00E-03
89	GOTERM_CC_DIRECT	nucleolus	1.2	4.80E-03	233	3.4	1.00E+00	6.60E-02	6.10E-02	3.90E-03
90	GOTERM_CC_DIRECT	perikaryon	1.6	5.30E-03	30	0.4	1.00E+00	7.20E-02	6.60E-02	2.50E-03
91	GOTERM_CC_DIRECT	collagen trimer	1.6	5.40E-03	27	0.4	1.00E+00	7.30E-02	6.70E-02	2.40E-03
92	GOTERM_CC_DIRECT	microtubule cytoskeleton	1.5	5.90E-03	41	0.6	1.00E+00	7.80E-02	7.20E-02	3.20E-03
93	GOTERM_CC_DIRECT	integral component of postsynaptic membrane	2.3	6.20E-03	11	0.2	1.00E+00	8.20E-02	7.50E-02	1.30E-03
94	GOTERM_CC_DIRECT	ruffle membrane	1.6	6.40E-03	29	0.4	1.00E+00	8.30E-02	7.60E-02	3.00E-03
95	GOTERM_CC_DIRECT	PML body	1.5	6.40E-03	33	0.5	1.00E+00	8.30E-02	7.60E-02	3.20E-03
96	GOTERM_CC_DIRECT	intercalated disc	1.9	6.50E-03	16	0.2	1.00E+00	8.30E-02	7.60E-02	2.10E-03
97	GOTERM_CC_DIRECT	recycling endosome	1.5	7.00E-03	40	0.6	1.00E+00	8.90E-02	8.10E-02	3.80E-03
98	GOTERM_CC_DIRECT	intrinsic component of plasma membrane	2.4	7.30E-03	10	0.1	1.00E+00	9.10E-02	8.30E-02	1.40E-03
99	GOTERM_CC_DIRECT	nBAF complex	2.4	7.30E-03	10	0.1	1.00E+00	9.10E-02	8.30E-02	1.40E-03
100	GOTERM_CC_DIRECT	immunological synapse	1.8	7.70E-03	19	0.3	1.00E+00	9.30E-02	8.50E-02	2.90E-03
101	GOTERM_CC_DIRECT	chromosome, centromeric region	1.8	7.70E-03	19	0.3	1.00E+00	9.30E-02	8.50E-02	2.90E-03
102	GOTERM_CC_DIRECT	transcriptional repressor complex	1.6	8.10E-03	25	0.4	1.00E+00	9.60E-02	8.80E-02	3.60E-03
103	GOTERM_CC_DIRECT	axon cytoplasm	1.7	8.10E-03	22	0.3	1.00E+00	9.60E-02	8.80E-02	3.40E-03
104	GOTERM_CC_DIRECT	histone deacetylase complex	1.7	8.80E-03	20	0.3	1.00E+00	1.00E-01	9.50E-02	3.50E-03
105	GOTERM_CC_DIRECT	centriolar satellite	1.4	8.90E-03	41	0.6	1.00E+00	1.00E-01	9.50E-02	5.00E-03
106	GOTERM_CC_DIRECT	ER-mitochondrion membrane contact site	2.1	9.20E-03	12	0.2	1.00E+00	1.10E-01	9.70E-02	2.40E-03
107	GOTERM_CC_DIRECT	spindle midzone	2	9.90E-03	14	0.2	1.00E+00	1.10E-01	1.00E-01	3.10E-03
108	GOTERM_CC_DIRECT	endocytic vesicle	1.7	9.90E-03	21	0.3	1.00E+00	1.10E-01	1.00E-01	4.10E-03
109	GOTERM_CC_DIRECT	caveola	1.6	1.00E-02	28	0.4	1.00E+00	1.10E-01	1.00E-01	4.90E-03
110	GOTERM_CC_DIRECT	tetraspanin-enriched microdomain	3	1.00E-02	7	0.1	1.00E+00	1.10E-01	1.00E-01	1.20E-03
111	GOTERM_CC_DIRECT	fascia adherens	3	1.00E-02	7	0.1	1.00E+00	1.10E-01	1.00E-01	1.20E-03
112	GOTERM_CC_DIRECT	heterotrimeric G-protein complex	1.7	1.10E-02	19	0.3	1.00E+00	1.20E-01	1.10E-01	4.30E-03
113	GOTERM_CC_DIRECT	nuclear membrane	1.3	1.10E-02	59	0.9	1.00E+00	1.20E-01	1.10E-01	7.30E-03
114	GOTERM_CC_DIRECT	actin cytoskeleton	1.3	1.20E-02	66	1	1.00E+00	1.30E-01	1.20E-01	7.60E-03
115	GOTERM_CC_DIRECT	endoplasmic reticulum membrane	1.2	1.30E-02	199	2.9	1.00E+00	1.30E-01	1.20E-01	1.00E-02
116	GOTERM_CC_DIRECT	bicellular tight junction	1.4	1.30E-02	40	0.6	1.00E+00	1.40E-01	1.20E-01	7.30E-03
117	GOTERM_CC_DIRECT	clathrin-coated pit	1.6	1.50E-02	22	0.3	1.00E+00	1.50E-01	1.40E-01	6.60E-03
118	GOTERM_CC_DIRECT	fibrillar center	1.3	1.50E-02	51	0.8	1.00E+00	1.50E-01	1.40E-01	9.20E-03
119	GOTERM_CC_DIRECT	presynaptic active zone	1.9	1.50E-02	14	0.2	1.00E+00	1.50E-01	1.40E-01	5.10E-03

120	GOTERM_CC_DIRECT	nuclear envelope	1.4	1.50E-02	39	0.6	1.00E+00	1.50E-01	1.40E-01	8.70E-03
121	GOTERM_CC_DIRECT	microtubule organizing center	1.4	1.60E-02	41	0.6	1.00E+00	1.60E-01	1.50E-01	9.20E-03
122	GOTERM_CC_DIRECT	RISC complex	2.3	1.60E-02	9	0.1	1.00E+00	1.60E-01	1.50E-01	3.50E-03
123	GOTERM_CC_DIRECT	extracellular exosome	1.5	1.70E-02	29	0.4	1.00E+00	1.70E-01	1.60E-01	8.90E-03
124	GOTERM_CC_DIRECT	transcription elongation factor complex	2.1	1.90E-02	11	0.2	1.00E+00	1.80E-01	1.70E-01	5.30E-03
125	GOTERM_CC_DIRECT	AMPA glutamate receptor complex	1.9	1.90E-02	13	0.2	1.00E+00	1.80E-01	1.70E-01	6.20E-03
126	GOTERM_CC_DIRECT	lysosomal membrane	1.3	1.90E-02	72	1.1	1.00E+00	1.90E-01	1.70E-01	1.30E-02
127	GOTERM_CC_DIRECT	Golgi cisterna membrane	1.6	2.20E-02	20	0.3	1.00E+00	2.10E-01	2.00E-01	1.00E-02
128	GOTERM_CC_DIRECT	myelin sheath	1.9	2.30E-02	12	0.2	1.00E+00	2.20E-01	2.00E-01	7.50E-03
129	GOTERM_CC_DIRECT	Cul2-RING ubiquitin ligase complex	1.9	2.30E-02	12	0.2	1.00E+00	2.20E-01	2.00E-01	7.50E-03
130	GOTERM_CC_DIRECT	cell junction	1.3	2.30E-02	48	0.7	1.00E+00	2.20E-01	2.00E-01	1.50E-02
131	GOTERM_CC_DIRECT	mitochondrial outer membrane	1.3	2.30E-02	49	0.7	1.00E+00	2.20E-01	2.00E-01	1.50E-02
132	GOTERM_CC_DIRECT	nuclear pore	1.6	2.40E-02	21	0.3	1.00E+00	2.20E-01	2.00E-01	1.10E-02
133	GOTERM_CC_DIRECT	autophagosome	1.5	2.40E-02	26	0.4	1.00E+00	2.20E-01	2.00E-01	1.20E-02
134	GOTERM_CC_DIRECT	Z disc	1.4	2.50E-02	37	0.5	1.00E+00	2.30E-01	2.10E-01	1.50E-02
135	GOTERM_CC_DIRECT	cytoplasmic microtubule	1.5	2.60E-02	23	0.3	1.00E+00	2.40E-01	2.20E-01	1.30E-02
136	GOTERM_CC_DIRECT	cytoskeleton of presynaptic active zone	2.9	2.70E-02	6	0.1	1.00E+00	2.40E-01	2.20E-01	3.40E-03
137	GOTERM_CC_DIRECT	actin filament	1.4	2.70E-02	30	0.4	1.00E+00	2.40E-01	2.20E-01	1.50E-02
138	GOTERM_CC_DIRECT	podosome	1.8	2.80E-02	13	0.2	1.00E+00	2.40E-01	2.20E-01	9.90E-03
139	GOTERM_CC_DIRECT	cell leading edge	1.7	2.80E-02	16	0.2	1.00E+00	2.50E-01	2.30E-01	1.20E-02
140	GOTERM_CC_DIRECT	intermediate filament cytoskeleton	1.7	2.80E-02	16	0.2	1.00E+00	2.50E-01	2.30E-01	1.20E-02
141	GOTERM_CC_DIRECT	neuronal cell body membrane	2.2	2.80E-02	9	0.1	1.00E+00	2.50E-01	2.30E-01	7.30E-03
142	GOTERM_CC_DIRECT	interstitial matrix	2.2	2.80E-02	9	0.1	1.00E+00	2.50E-01	2.30E-01	7.30E-03
143	GOTERM_CC_DIRECT	cell body	1.6	2.90E-02	20	0.3	1.00E+00	2.50E-01	2.30E-01	1.40E-02
144	GOTERM_CC_DIRECT	transcription factor AP-1 complex	3.4	2.90E-02	5	0.1	1.00E+00	2.50E-01	2.30E-01	2.20E-03
145	GOTERM_CC_DIRECT	intracellular membrane-bounded organelle	1.2	3.10E-02	100	1.5	1.00E+00	2.60E-01	2.40E-01	2.30E-02
146	GOTERM_CC_DIRECT	trans-Golgi network membrane	1.6	3.30E-02	18	0.3	1.00E+00	2.80E-01	2.50E-01	1.50E-02
147	GOTERM_CC_DIRECT	filopodium membrane	2.3	3.50E-02	8	0.1	1.00E+00	2.90E-01	2.70E-01	8.50E-03
148	GOTERM_CC_DIRECT	Flemming body	2	3.60E-02	10	0.1	1.00E+00	3.00E-01	2.80E-01	1.10E-02
149	GOTERM_CC_DIRECT	mediator complex	1.5	3.70E-02	20	0.3	1.00E+00	3.00E-01	2.80E-01	1.80E-02
150	GOTERM_CC_DIRECT	cortical actin cytoskeleton	1.5	3.70E-02	20	0.3	1.00E+00	3.00E-01	2.80E-01	1.80E-02
151	GOTERM_CC_DIRECT	lysosome	1.2	4.00E-02	77	1.1	1.00E+00	3.30E-01	3.00E-01	2.90E-02
152	GOTERM_CC_DIRECT	axon terminus	1.7	4.30E-02	14	0.2	1.00E+00	3.50E-01	3.20E-01	1.80E-02
153	GOTERM_CC_DIRECT	TORC2 complex	2.4	4.40E-02	7	0.1	1.00E+00	3.50E-01	3.20E-01	9.60E-03
154	GOTERM_CC_DIRECT	melanosome	1.4	4.70E-02	31	0.5	1.00E+00	3.80E-01	3.50E-01	2.80E-02
155	GOTERM_CC_DIRECT	autophagosome membrane	1.6	5.00E-02	16	0.2	1.00E+00	3.90E-01	3.60E-01	2.30E-02

Table S2C. Gene ontology (GO) enrichment analysis based on DE miRNAs in the serum of LSDV-infected bovines compared to their expression in the non-infected control. Criteria: P < 0.05, number of genes in each GO term > 5, and fold change of Log2-treatment/control ≥ 2. (C) molecular function.

No	Category	Term	Fold Enri	P-Value	Count	%	Bonferron	Benjamini	FDR	Fisher Exact
1	GOTERM_MF_DIRECT	RNA polymerase II core promoter	1.7	5.70E-47	524	7.7	1.40E-43	1.40E-43	1.30E-43	2.40E-47
2	GOTERM_MF_DIRECT	RNA polymerase II transcription factor activity	1.7	2.40E-46	495	7.3	5.70E-43	2.80E-43	2.70E-43	9.50E-47
3	GOTERM_MF_DIRECT	transcriptional activator activity, RNA polymerase II	2	2.00E-37	247	3.7	4.90E-34	1.60E-34	1.60E-34	5.70E-38
4	GOTERM_MF_DIRECT	metal ion binding	1.4	1.70E-22	661	9.8	4.10E-19	1.00E-19	9.90E-20	1.10E-22
5	GOTERM_MF_DIRECT	protein serine/threonine kinase activity	1.8	7.40E-19	168	2.5	1.80E-15	3.60E-16	3.40E-16	2.70E-19
6	GOTERM_MF_DIRECT	ATP binding	1.3	2.00E-18	570	8.4	5.00E-15	8.30E-16	7.90E-16	1.30E-18
7	GOTERM_MF_DIRECT	transcriptional repressor activity, RNA polymerase II	1.8	3.50E-18	150	2.2	8.50E-15	1.20E-15	1.20E-15	1.20E-18
8	GOTERM_MF_DIRECT	identical protein binding	1.4	5.20E-18	439	6.5	1.30E-14	1.60E-15	1.50E-15	3.10E-18
9	GOTERM_MF_DIRECT	protein kinase binding	1.7	2.30E-16	185	2.7	5.40E-13	6.30E-14	6.10E-14	5.20E-17
10	GOTERM_MF_DIRECT	protein serine/threonine/tyrosine kinase activity	1.8	3.20E-16	144	2.1	8.10E-13	7.80E-14	7.50E-14	7.70E-17
11	GOTERM_MF_DIRECT	GTPase activator activity	1.8	4.10E-13	107	1.6	9.80E-10	9.00E-11	8.60E-11	1.40E-13
12	GOTERM_MF_DIRECT	transcription factor activity, sequence-specific DNA binding	1.7	4.90E-13	140	2.1	1.20E-09	9.90E-11	9.60E-11	2.00E-13
13	GOTERM_MF_DIRECT	beta-catenin binding	2.3	4.50E-11	51	0.8	1.10E-07	8.50E-09	8.10E-09	9.30E-12
14	GOTERM_MF_DIRECT	protein dimerization activity	1.9	1.00E-10	81	1.2	2.40E-07	1.70E-08	1.70E-08	3.30E-11
15	GOTERM_MF_DIRECT	guanyl-nucleotide exchange factor activity	1.7	1.40E-10	106	1.6	3.30E-07	2.20E-08	2.20E-08	5.50E-11
16	GOTERM_MF_DIRECT	RNA polymerase II regulatory	1.7	1.70E-10	101	1.5	4.00E-07	2.50E-08	2.40E-08	6.50E-11
17	GOTERM_MF_DIRECT	transcription cofactor activity	1.9	3.90E-09	68	1	9.40E-06	5.50E-07	5.30E-07	1.30E-09
18	GOTERM_MF_DIRECT	small GTPase binding	1.6	6.10E-09	104	1.5	1.50E-05	8.30E-07	8.00E-07	2.70E-09
19	GOTERM_MF_DIRECT	actin binding	1.6	7.00E-09	110	1.6	1.70E-05	8.90E-07	8.60E-07	3.20E-09
20	GOTERM_MF_DIRECT	zinc ion binding	1.3	8.80E-09	287	4.2	2.10E-05	1.10E-06	1.00E-06	5.60E-09
21	GOTERM_MF_DIRECT	growth factor activity	1.8	1.60E-08	72	1.1	4.00E-05	1.90E-06	1.80E-06	6.00E-09
22	GOTERM_MF_DIRECT	phosphatidylinositol binding	1.9	2.60E-08	57	0.8	6.40E-05	2.90E-06	2.80E-06	8.30E-09
23	GOTERM_MF_DIRECT	protein homodimerization activity	1.4	3.80E-08	237	3.5	9.30E-05	4.00E-06	3.90E-06	2.40E-08
24	GOTERM_MF_DIRECT	SMAD binding	2.5	7.50E-08	30	0.4	1.80E-04	7.30E-06	7.10E-06	1.20E-08
25	GOTERM_MF_DIRECT	chromatin binding	1.4	7.60E-08	164	2.4	1.80E-04	7.30E-06	7.10E-06	4.30E-08
26	GOTERM_MF_DIRECT	GDP binding	2.1	9.80E-08	42	0.6	2.40E-04	9.10E-06	8.80E-06	2.50E-08
27	GOTERM_MF_DIRECT	RNA polymerase II sequence-specific DNA binding transcription factor binding	1.8	1.60E-07	66	1	4.00E-04	1.50E-05	1.40E-05	6.20E-08
28	GOTERM_MF_DIRECT	histone deacetylase binding	1.9	2.20E-07	53	0.8	5.30E-04	1.90E-05	1.80E-05	7.20E-08
29	GOTERM_MF_DIRECT	ubiquitin protein ligase binding	1.5	2.40E-07	112	1.7	5.80E-04	2.00E-05	1.90E-05	1.20E-07
30	GOTERM_MF_DIRECT	ion channel binding	1.8	4.40E-07	54	0.8	1.10E-03	3.60E-05	3.40E-05	1.50E-07
31	GOTERM_MF_DIRECT	chromatin DNA binding	2.1	6.50E-07	37	0.5	1.60E-03	5.10E-05	4.90E-05	1.70E-07
32	GOTERM_MF_DIRECT	PDZ domain binding	2.1	1.20E-06	37	0.5	2.80E-03	8.80E-05	8.50E-05	3.10E-07
33	GOTERM_MF_DIRECT	macromolecular complex binding	1.7	1.50E-06	71	1	3.70E-03	1.10E-04	1.10E-04	6.60E-07
34	GOTERM_MF_DIRECT	sequence-specific double-stranded DNA binding	2.2	3.10E-06	30	0.4	7.50E-03	2.20E-04	2.10E-04	7.30E-07
35	GOTERM_MF_DIRECT	protein tyrosine phosphatase activity	1.7	4.80E-06	54	0.8	1.20E-02	3.30E-04	3.20E-04	1.90E-06
36	GOTERM_MF_DIRECT	RNA polymerase II transcription factor activity, ligand-activated sequence-specific DNA	2	6.60E-06	33	0.5	1.60E-02	4.40E-04	4.30E-04	1.80E-06
37	GOTERM_MF_DIRECT	calmodulin binding	1.6	8.80E-06	65	1	2.10E-02	5.80E-04	5.50E-04	3.90E-06
38	GOTERM_MF_DIRECT	mRNA 3'-UTR binding	1.9	9.30E-06	37	0.5	2.20E-02	6.00E-04	5.70E-04	2.90E-06
39	GOTERM_MF_DIRECT	protein binding	1.5	1.20E-05	95	1.4	2.90E-02	7.70E-04	7.40E-04	6.60E-06
40	GOTERM_MF_DIRECT	protein domain specific binding	1.6	1.40E-05	72	1.1	3.30E-02	8.30E-04	8.00E-04	6.50E-06
41	GOTERM_MF_DIRECT	histone binding	1.6	1.50E-05	70	1	3.60E-02	8.80E-04	8.50E-04	7.10E-06

42	GOTERM_MF_DIRECT	transcription corepressor binding	2.2	1.90E-05	26	0.4	4.40E-02	1.10E-03	1.00E-03	4.40E-06
43	GOTERM_MF_DIRECT	extracellular matrix structural constituent	1.9	2.60E-05	34	0.5	6.00E-02	1.40E-03	1.40E-03	8.10E-06
44	GOTERM_MF_DIRECT	transcription coactivator activity	1.5	2.80E-05	85	1.3	6.50E-02	1.50E-03	1.50E-03	1.50E-05
45	GOTERM_MF_DIRECT	mRNA binding	1.4	5.10E-05	93	1.4	1.20E-01	2.80E-03	2.70E-03	2.90E-05
46	GOTERM_MF_DIRECT	calcium ion binding	1.2	6.50E-05	235	3.5	1.50E-01	3.40E-03	3.30E-03	4.60E-05
47	GOTERM_MF_DIRECT	signaling receptor activity	1.6	6.80E-05	54	0.8	1.50E-01	3.50E-03	3.40E-03	3.10E-05
48	GOTERM_MF_DIRECT	potassium channel regulator activity	2.4	8.20E-05	19	0.3	1.80E-01	4.10E-03	3.90E-03	1.60E-05
49	GOTERM_MF_DIRECT	phosphoprotein phosphatase activity	2.4	8.20E-05	19	0.3	1.80E-01	4.10E-03	3.90E-03	1.60E-05
50	GOTERM_MF_DIRECT	voltage-gated potassium channel activity	1.9	9.20E-05	32	0.5	2.00E-01	4.50E-03	4.30E-03	3.10E-05
51	GOTERM_MF_DIRECT	transcription cofactor binding	2.6	1.00E-04	15	0.2	2.10E-01	4.70E-03	4.60E-03	1.40E-05
52	GOTERM_MF_DIRECT	sequence-specific DNA binding	1.5	1.20E-04	75	1.1	2.60E-01	5.70E-03	5.50E-03	6.60E-05
53	GOTERM_MF_DIRECT	transcription corepressor activity	1.5	1.30E-04	68	1	2.70E-01	5.90E-03	5.70E-03	6.70E-05
54	GOTERM_MF_DIRECT	phosphatidylinositol-3-phosphate binding	2.1	1.40E-04	24	0.4	3.00E-01	6.40E-03	6.20E-03	4.00E-05
55	GOTERM_MF_DIRECT	SH3 domain binding	1.7	1.50E-04	38	0.6	3.00E-01	6.40E-03	6.20E-03	5.70E-05
56	GOTERM_MF_DIRECT	ligand-dependent nuclear receptor transcription coactivator activity	2	1.70E-04	26	0.4	3.40E-01	7.40E-03	7.10E-03	5.20E-05
57	GOTERM_MF_DIRECT	receptor binding	1.4	1.70E-04	88	1.3	3.40E-01	7.40E-03	7.10E-03	9.90E-05
58	GOTERM_MF_DIRECT	bHLH transcription factor binding	2.4	2.10E-04	17	0.3	3.90E-01	8.70E-03	8.30E-03	4.00E-05
59	GOTERM_MF_DIRECT	protein kinase activity	1.4	2.30E-04	79	1.2	4.20E-01	9.30E-03	8.90E-03	1.30E-04
60	GOTERM_MF_DIRECT	scaffold protein binding	1.9	2.30E-04	27	0.4	4.30E-01	9.30E-03	9.00E-03	7.40E-05
61	GOTERM_MF_DIRECT	alpha-tubulin binding	2.1	2.30E-04	21	0.3	4.30E-01	9.30E-03	9.00E-03	5.90E-05
62	GOTERM_MF_DIRECT	magnesium ion binding	1.4	2.40E-04	86	1.3	4.40E-01	9.30E-03	9.00E-03	1.40E-04
63	GOTERM_MF_DIRECT	heparin binding	1.5	2.60E-04	60	0.9	4.70E-01	9.90E-03	9.60E-03	1.30E-04
64	GOTERM_MF_DIRECT	ubiquitin protein ligase activity	1.3	2.60E-04	114	1.7	4.70E-01	1.00E-02	9.60E-03	1.70E-04
65	GOTERM_MF_DIRECT	GTPase activity	1.3	3.60E-04	127	1.9	5.80E-01	1.30E-02	1.30E-02	2.30E-04
66	GOTERM_MF_DIRECT	enzyme binding	1.4	3.90E-04	72	1.1	6.10E-01	1.40E-02	1.40E-02	2.20E-04
67	GOTERM_MF_DIRECT	GTP binding	1.3	4.60E-04	148	2.2	6.70E-01	1.60E-02	1.60E-02	3.10E-04
68	GOTERM_MF_DIRECT	DNA binding	1.2	5.00E-04	307	4.5	7.00E-01	1.80E-02	1.70E-02	3.90E-04
69	GOTERM_MF_DIRECT	inward rectifier potassium channel activity	2.4	5.20E-04	15	0.2	7.20E-01	1.80E-02	1.80E-02	1.00E-04
70	GOTERM_MF_DIRECT	protein kinase inhibitor activity	2.5	5.70E-04	14	0.2	7.50E-01	2.00E-02	1.90E-02	1.00E-04
71	GOTERM_MF_DIRECT	SNAP receptor activity	2	5.80E-04	22	0.3	7.50E-01	2.00E-02	1.90E-02	1.70E-04
72	GOTERM_MF_DIRECT	glycosaminoglycan binding	2.7	6.30E-04	12	0.2	7.80E-01	2.10E-02	2.00E-02	8.50E-05
73	GOTERM_MF_DIRECT	protein C-terminus binding	1.5	7.90E-04	55	0.8	8.50E-01	2.60E-02	2.50E-02	4.10E-04
74	GOTERM_MF_DIRECT	voltage-gated ion channel activity	2.1	9.80E-04	19	0.3	9.10E-01	3.20E-02	3.10E-02	2.70E-04
75	GOTERM_MF_DIRECT	transmembrane receptor protein tyrosine kinase activity	1.8	1.00E-03	26	0.4	9.10E-01	3.30E-02	3.10E-02	3.60E-04
76	GOTERM_MF_DIRECT	zinc ion transmembrane transporter activity	2.3	1.00E-03	15	0.2	9.20E-01	3.30E-02	3.20E-02	2.30E-04
77	GOTERM_MF_DIRECT	microtubule binding	1.3	1.10E-03	93	1.4	9.30E-01	3.50E-02	3.40E-02	7.10E-04
78	GOTERM_MF_DIRECT	semaphorin receptor binding	2.3	1.20E-03	14	0.2	9.40E-01	3.70E-02	3.60E-02	2.40E-04
79	GOTERM_MF_DIRECT	thiol-dependent ubiquitin-specific protease activity	1.6	1.20E-03	43	0.6	9.50E-01	3.70E-02	3.60E-02	5.90E-04
80	GOTERM_MF_DIRECT	transcription regulatory region sequence-specific DNA binding	1.6	1.20E-03	40	0.6	9.50E-01	3.80E-02	3.60E-02	5.90E-04
81	GOTERM_MF_DIRECT	protein serine/threonine kinase activator activity	2	1.30E-03	20	0.3	9.60E-01	4.00E-02	3.90E-02	4.10E-04
82	GOTERM_MF_DIRECT	chemorepellent activity	2.1	1.40E-03	17	0.3	9.70E-01	4.10E-02	4.00E-02	3.70E-04
83	GOTERM_MF_DIRECT	phosphatidylinositol-3-phosphatase activity	2.5	1.50E-03	12	0.2	9.70E-01	4.30E-02	4.10E-02	2.50E-04
84	GOTERM_MF_DIRECT	protein tyrosine/threonine phosphatase activity	3	1.50E-03	9	0.1	9.70E-01	4.40E-02	4.20E-02	1.40E-04
85	GOTERM_MF_DIRECT	receptor tyrosine kinase binding	1.7	1.70E-03	27	0.4	9.90E-01	5.00E-02	4.80E-02	6.90E-04
86	GOTERM_MF_DIRECT	actin filament binding	1.3	1.80E-03	82	1.2	9.90E-01	5.00E-02	4.90E-02	1.10E-03

87	GOTERM_MF_DIRECT	ubiquitin binding	1.6	1.80E-03	38	0.6	9.90E-01	5.00E-02	4.90E-02	8.60E-04
88	GOTERM_MF_DIRECT	protein phosphatase 1 binding	2.2	1.90E-03	15	0.2	9.90E-01	5.40E-02	5.20E-02	4.70E-04
89	GOTERM_MF_DIRECT	protein tyrosine kinase activity	1.7	2.00E-03	28	0.4	9.90E-01	5.50E-02	5.30E-02	8.30E-04
90	GOTERM_MF_DIRECT	14-3-3 protein binding	2.2	2.30E-03	14	0.2	1.00E+00	6.10E-02	5.80E-02	5.30E-04
91	GOTERM_MF_DIRECT	voltage-gated sodium channel activity	2.2	2.30E-03	14	0.2	1.00E+00	6.10E-02	5.80E-02	5.30E-04
92	GOTERM_MF_DIRECT	Wnt-protein binding	2	2.40E-03	17	0.3	1.00E+00	6.20E-02	6.00E-02	6.70E-04
93	GOTERM_MF_DIRECT	manganese ion binding	1.7	2.50E-03	27	0.4	1.00E+00	6.40E-02	6.20E-02	1.00E-03
94	GOTERM_MF_DIRECT	p53 binding	1.6	2.60E-03	30	0.4	1.00E+00	6.70E-02	6.50E-02	1.10E-03
95	GOTERM_MF_DIRECT	integrin binding	1.5	2.70E-03	48	0.7	1.00E+00	6.70E-02	6.50E-02	1.40E-03
96	GOTERM_MF_DIRECT	E-box binding	1.8	2.70E-03	23	0.3	1.00E+00	6.70E-02	6.50E-02	9.90E-04
97	GOTERM_MF_DIRECT	delayed rectifier potassium channel activity	2.1	2.80E-03	16	0.2	1.00E+00	7.10E-02	6.80E-02	7.80E-04
98	GOTERM_MF_DIRECT	frizzled binding	1.9	3.30E-03	20	0.3	1.00E+00	8.10E-02	7.80E-02	1.10E-03
99	GOTERM_MF_DIRECT	ephrin receptor binding	2.1	3.40E-03	15	0.2	1.00E+00	8.30E-02	8.00E-02	9.10E-04
100	GOTERM_MF_DIRECT	tropomyosin binding	2.5	3.50E-03	11	0.2	1.00E+00	8.30E-02	8.00E-02	6.30E-04
101	GOTERM_MF_DIRECT	1-phosphatidylinositol-3-kinase regulator activity	2.5	3.50E-03	11	0.2	1.00E+00	8.30E-02	8.00E-02	6.30E-04
102	GOTERM_MF_DIRECT	myosin V binding	2.6	3.80E-03	10	0.1	1.00E+00	9.10E-02	8.80E-02	6.20E-04
103	GOTERM_MF_DIRECT	epidermal growth factor receptor binding	2.1	4.10E-03	14	0.2	1.00E+00	9.20E-02	8.90E-02	1.00E-03
104	GOTERM_MF_DIRECT	activin binding	2.7	4.10E-03	9	0.1	1.00E+00	9.20E-02	8.90E-02	5.50E-04
105	GOTERM_MF_DIRECT	acetylgalactosaminyltransferase activity	2.7	4.10E-03	9	0.1	1.00E+00	9.20E-02	8.90E-02	5.50E-04
106	GOTERM_MF_DIRECT	transmembrane receptor protein serine/threonine kinase activity	2.7	4.10E-03	9	0.1	1.00E+00	9.20E-02	8.90E-02	5.50E-04
107	GOTERM_MF_DIRECT	armadillo repeat domain binding	3	4.10E-03	8	0.1	1.00E+00	9.20E-02	8.90E-02	4.10E-04
108	GOTERM_MF_DIRECT	platelet-derived growth factor binding	3	4.10E-03	8	0.1	1.00E+00	9.20E-02	8.90E-02	4.10E-04
109	GOTERM_MF_DIRECT	phosphotyrosine binding	1.8	4.70E-03	22	0.3	1.00E+00	1.00E-01	1.00E-01	1.80E-03
110	GOTERM_MF_DIRECT	ATPase binding	1.6	5.50E-03	32	0.5	1.00E+00	1.20E-01	1.20E-01	2.70E-03
111	GOTERM_MF_DIRECT	phosphatidylinositol-3,5-bisphosphate binding	2	5.60E-03	15	0.2	1.00E+00	1.20E-01	1.20E-01	1.70E-03
112	GOTERM_MF_DIRECT	calmodulin-dependent protein kinase activity	2.2	5.70E-03	12	0.2	1.00E+00	1.20E-01	1.20E-01	1.30E-03
113	GOTERM_MF_DIRECT	fibroblast growth factor binding	2.2	5.70E-03	12	0.2	1.00E+00	1.20E-01	1.20E-01	1.30E-03
114	GOTERM_MF_DIRECT	beta-amyloid binding	1.7	6.20E-03	24	0.4	1.00E+00	1.30E-01	1.30E-01	2.60E-03
115	GOTERM_MF_DIRECT	protein kinase A regulatory subunit binding	2	6.80E-03	14	0.2	1.00E+00	1.40E-01	1.40E-01	1.90E-03
116	GOTERM_MF_DIRECT	cyclin binding	1.9	7.20E-03	16	0.2	1.00E+00	1.50E-01	1.50E-01	2.40E-03
117	GOTERM_MF_DIRECT	transcription coactivator binding	1.8	7.30E-03	18	0.3	1.00E+00	1.50E-01	1.50E-01	2.60E-03
118	GOTERM_MF_DIRECT	magnesium ion transmembrane transporter activity	2.4	7.90E-03	10	0.1	1.00E+00	1.60E-01	1.50E-01	1.60E-03
119	GOTERM_MF_DIRECT	Wnt-activated receptor activity	2.4	7.90E-03	10	0.1	1.00E+00	1.60E-01	1.50E-01	1.60E-03
120	GOTERM_MF_DIRECT	BMP receptor binding	2.4	7.90E-03	10	0.1	1.00E+00	1.60E-01	1.50E-01	1.60E-03
121	GOTERM_MF_DIRECT	cadherin binding	1.6	8.30E-03	27	0.4	1.00E+00	1.70E-01	1.60E-01	3.90E-03
122	GOTERM_MF_DIRECT	ionotropic glutamate receptor binding	2.5	9.10E-03	9	0.1	1.00E+00	1.80E-01	1.70E-01	1.60E-03
123	GOTERM_MF_DIRECT	clathrin adaptor activity	2.1	1.00E-02	12	0.2	1.00E+00	1.90E-01	1.80E-01	2.70E-03
124	GOTERM_MF_DIRECT	G-protein beta/gamma-subunit complex binding	2.1	1.00E-02	12	0.2	1.00E+00	1.90E-01	1.80E-01	2.70E-03
125	GOTERM_MF_DIRECT	insulin receptor binding	2.1	1.00E-02	12	0.2	1.00E+00	1.90E-01	1.80E-01	2.70E-03
126	GOTERM_MF_DIRECT	AP-2 adaptor complex binding	2.7	1.00E-02	8	0.1	1.00E+00	1.90E-01	1.80E-01	1.50E-03
127	GOTERM_MF_DIRECT	chloride channel inhibitor activity	2.7	1.00E-02	8	0.1	1.00E+00	1.90E-01	1.80E-01	1.50E-03
128	GOTERM_MF_DIRECT	platelet-derived growth factor receptor binding	2.7	1.00E-02	8	0.1	1.00E+00	1.90E-01	1.80E-01	1.50E-03
129	GOTERM_MF_DIRECT	mitogen-activated protein kinase kinase kinase binding	2.7	1.00E-02	8	0.1	1.00E+00	1.90E-01	1.80E-01	1.50E-03
130	GOTERM_MF_DIRECT	ubiquitin conjugating enzyme binding	1.8	1.10E-02	18	0.3	1.00E+00	2.00E-01	1.90E-01	4.00E-03
131	GOTERM_MF_DIRECT	fibroblast growth factor-activated receptor activity	3.4	1.10E-02	6	0.1	1.00E+00	2.00E-01	1.90E-01	7.00E-04

132	GOTERM_MF_DIRECT	fatty acid elongase activity	2.9	1.10E-02	7	0.1	1.00E+00	2.00E-01	1.90E-01	1.20E-03
133	GOTERM_MF_DIRECT	calcium-dependent protein kinase C activity	2.2	1.20E-02	11	0.2	1.00E+00	2.20E-01	2.10E-01	3.10E-03
134	GOTERM_MF_DIRECT	microtubule plus-end binding	2.2	1.20E-02	11	0.2	1.00E+00	2.20E-01	2.10E-01	3.10E-03
135	GOTERM_MF_DIRECT	tau-protein kinase activity	2.2	1.20E-02	11	0.2	1.00E+00	2.20E-01	2.10E-01	3.10E-03
136	GOTERM_MF_DIRECT	protein binding, bridging involved in substrate recognition for ubiquitination	1.7	1.20E-02	19	0.3	1.00E+00	2.20E-01	2.10E-01	4.90E-03
137	GOTERM_MF_DIRECT	G-protein coupled receptor binding	1.6	1.30E-02	26	0.4	1.00E+00	2.30E-01	2.20E-01	6.30E-03
138	GOTERM_MF_DIRECT	poly(U) RNA binding	2	1.30E-02	13	0.2	1.00E+00	2.30E-01	2.20E-01	4.10E-03
139	GOTERM_MF_DIRECT	protein phosphatase 2A binding	2	1.30E-02	13	0.2	1.00E+00	2.30E-01	2.20E-01	4.10E-03
140	GOTERM_MF_DIRECT	ubiquitin conjugating enzyme activity	1.7	1.50E-02	18	0.3	1.00E+00	2.60E-01	2.50E-01	5.90E-03
141	GOTERM_MF_DIRECT	lysine-acetylated histone binding	2	1.60E-02	12	0.2	1.00E+00	2.80E-01	2.70E-01	4.80E-03
142	GOTERM_MF_DIRECT	protein serine/threonine phosphatase activity	1.7	1.70E-02	19	0.3	1.00E+00	2.90E-01	2.80E-01	7.00E-03
143	GOTERM_MF_DIRECT	cysteine-type endopeptidase activity	1.5	1.70E-02	26	0.4	1.00E+00	2.90E-01	2.80E-01	8.40E-03
144	GOTERM_MF_DIRECT	MAP kinase tyrosine/serine/threonine phosphatase activity	2.3	1.80E-02	9	0.1	1.00E+00	2.90E-01	2.80E-01	3.80E-03
145	GOTERM_MF_DIRECT	1-phosphatidylinositol binding	2.3	1.80E-02	9	0.1	1.00E+00	2.90E-01	2.80E-01	3.80E-03
146	GOTERM_MF_DIRECT	chaperone binding	1.4	1.90E-02	34	0.5	1.00E+00	3.20E-01	3.10E-01	1.10E-02
147	GOTERM_MF_DIRECT	transmitter-gated ion channel activity involved in regulation of postsynaptic membrane po	1.8	2.00E-02	15	0.2	1.00E+00	3.20E-01	3.10E-01	7.30E-03
148	GOTERM_MF_DIRECT	ligand-dependent nuclear receptor binding	1.8	2.00E-02	15	0.2	1.00E+00	3.20E-01	3.10E-01	7.30E-03
149	GOTERM_MF_DIRECT	protein phosphatase binding	1.5	2.10E-02	30	0.4	1.00E+00	3.30E-01	3.20E-01	1.10E-02
150	GOTERM_MF_DIRECT	cyclin-dependent protein serine/threonine kinase inhibitor activity	2.4	2.10E-02	8	0.1	1.00E+00	3.30E-01	3.20E-01	4.10E-03
151	GOTERM_MF_DIRECT	translation repressor activity	2.4	2.10E-02	8	0.1	1.00E+00	3.30E-01	3.20E-01	4.10E-03
152	GOTERM_MF_DIRECT	cullin family protein binding	1.7	2.20E-02	16	0.2	1.00E+00	3.60E-01	3.50E-01	8.90E-03
153	GOTERM_MF_DIRECT	promoter-specific chromatin binding	1.5	2.30E-02	23	0.3	1.00E+00	3.70E-01	3.50E-01	1.10E-02
154	GOTERM_MF_DIRECT	growth factor receptor binding	2.6	2.50E-02	7	0.1	1.00E+00	3.70E-01	3.60E-01	4.10E-03
155	GOTERM_MF_DIRECT	GDP-dissociation inhibitor activity	2.6	2.50E-02	7	0.1	1.00E+00	3.70E-01	3.60E-01	4.10E-03
156	GOTERM_MF_DIRECT	SUMO ligase activity	2.6	2.50E-02	7	0.1	1.00E+00	3.70E-01	3.60E-01	4.10E-03
157	GOTERM_MF_DIRECT	deacetylase activity	2.6	2.50E-02	7	0.1	1.00E+00	3.70E-01	3.60E-01	4.10E-03
158	GOTERM_MF_DIRECT	calcium-transporting ATPase activity	2.6	2.50E-02	7	0.1	1.00E+00	3.70E-01	3.60E-01	4.10E-03
159	GOTERM_MF_DIRECT	H4 histone acetyltransferase activity	2.6	2.50E-02	7	0.1	1.00E+00	3.70E-01	3.60E-01	4.10E-03
160	GOTERM_MF_DIRECT	core promoter proximal region sequence-specific DNA binding	2.1	2.50E-02	10	0.1	1.00E+00	3.70E-01	3.60E-01	6.80E-03
161	GOTERM_MF_DIRECT	RNA polymerase binding	2.1	2.50E-02	10	0.1	1.00E+00	3.70E-01	3.60E-01	6.80E-03
162	GOTERM_MF_DIRECT	R-SMAD binding	1.9	2.50E-02	12	0.2	1.00E+00	3.70E-01	3.60E-01	8.30E-03
163	GOTERM_MF_DIRECT	mRNA 3'-UTR AU-rich region binding	1.9	2.50E-02	12	0.2	1.00E+00	3.70E-01	3.60E-01	8.30E-03
164	GOTERM_MF_DIRECT	motor activity	1.7	2.50E-02	17	0.3	1.00E+00	3.70E-01	3.60E-01	1.10E-02
165	GOTERM_MF_DIRECT	phosphatidylinositol-3,4,5-trisphosphate binding	1.7	2.50E-02	17	0.3	1.00E+00	3.70E-01	3.60E-01	1.10E-02
166	GOTERM_MF_DIRECT	beta-tubulin binding	1.6	2.80E-02	18	0.3	1.00E+00	4.00E-01	3.80E-01	1.20E-02
167	GOTERM_MF_DIRECT	vascular endothelial growth factor receptor 2 binding	2.9	2.80E-02	6	0.1	1.00E+00	4.00E-01	3.80E-01	3.70E-03
168	GOTERM_MF_DIRECT	BMP receptor activity	2.9	2.80E-02	6	0.1	1.00E+00	4.00E-01	3.80E-01	3.70E-03
169	GOTERM_MF_DIRECT	interleukin-6 receptor binding	2.9	2.80E-02	6	0.1	1.00E+00	4.00E-01	3.80E-01	3.70E-03
170	GOTERM_MF_DIRECT	clathrin light chain binding	2.9	2.80E-02	6	0.1	1.00E+00	4.00E-01	3.80E-01	3.70E-03
171	GOTERM_MF_DIRECT	protein binding involved in cell-cell adhesion	1.8	3.00E-02	13	0.2	1.00E+00	4.10E-01	3.90E-01	1.10E-02
172	GOTERM_MF_DIRECT	transforming growth factor beta receptor activity, type I	3.4	3.00E-02	5	0.1	1.00E+00	4.10E-01	3.90E-01	2.40E-03
173	GOTERM_MF_DIRECT	collagen binding involved in cell-matrix adhesion	3.4	3.00E-02	5	0.1	1.00E+00	4.10E-01	3.90E-01	2.40E-03
174	GOTERM_MF_DIRECT	UDP-galactose:beta-N-acetylglucosamine beta-1,3-galactosyltransferase activity	3.4	3.00E-02	5	0.1	1.00E+00	4.10E-01	3.90E-01	2.40E-03
175	GOTERM_MF_DIRECT	PH domain binding	3.4	3.00E-02	5	0.1	1.00E+00	4.10E-01	3.90E-01	2.40E-03
176	GOTERM_MF_DIRECT	RNA strand annealing activity	3.4	3.00E-02	5	0.1	1.00E+00	4.10E-01	3.90E-01	2.40E-03

177	GOTERM_MF_DIRECT	activin receptor activity, type I	3.4	3.00E-02	5	0.1	1.00E+00	4.10E-01	3.90E-01	2.40E-03
178	GOTERM_MF_DIRECT	insulin binding	3.4	3.00E-02	5	0.1	1.00E+00	4.10E-01	3.90E-01	2.40E-03
179	GOTERM_MF_DIRECT	I-SMAD binding	2.2	3.00E-02	9	0.1	1.00E+00	4.10E-01	3.90E-01	7.90E-03
180	GOTERM_MF_DIRECT	channel activity	2.2	3.00E-02	9	0.1	1.00E+00	4.10E-01	3.90E-01	7.90E-03
181	GOTERM_MF_DIRECT	phosphatidylinositol 3-kinase binding	2.2	3.00E-02	9	0.1	1.00E+00	4.10E-01	3.90E-01	7.90E-03
182	GOTERM_MF_DIRECT	kinesin binding	1.7	3.10E-02	16	0.2	1.00E+00	4.10E-01	4.00E-01	1.30E-02
183	GOTERM_MF_DIRECT	microtubule motor activity	1.6	3.20E-02	20	0.3	1.00E+00	4.20E-01	4.10E-01	1.50E-02
184	GOTERM_MF_DIRECT	voltage-gated calcium channel activity	1.6	3.20E-02	20	0.3	1.00E+00	4.20E-01	4.10E-01	1.50E-02
185	GOTERM_MF_DIRECT	ligand-gated ion channel activity	1.8	3.70E-02	12	0.2	1.00E+00	4.80E-01	4.60E-01	1.30E-02
186	GOTERM_MF_DIRECT	fibronectin binding	1.8	3.70E-02	12	0.2	1.00E+00	4.80E-01	4.60E-01	1.30E-02
187	GOTERM_MF_DIRECT	HMG box domain binding	2.2	3.70E-02	8	0.1	1.00E+00	4.80E-01	4.70E-01	9.10E-03
188	GOTERM_MF_DIRECT	transferase activity, transferring glycosyl groups	1.5	3.90E-02	24	0.4	1.00E+00	4.90E-01	4.80E-01	2.00E-02
189	GOTERM_MF_DIRECT	low-density lipoprotein particle receptor binding	2	3.90E-02	10	0.1	1.00E+00	4.90E-01	4.80E-01	1.20E-02
190	GOTERM_MF_DIRECT	syntaxin-1 binding	2	3.90E-02	10	0.1	1.00E+00	4.90E-01	4.80E-01	1.20E-02
191	GOTERM_MF_DIRECT	kinase activity	1.4	4.00E-02	34	0.5	1.00E+00	5.00E-01	4.90E-01	2.40E-02
192	GOTERM_MF_DIRECT	androgen receptor binding	1.7	4.20E-02	13	0.2	1.00E+00	5.30E-01	5.10E-01	1.70E-02
193	GOTERM_MF_DIRECT	C-C chemokine receptor activity	1.7	4.20E-02	13	0.2	1.00E+00	5.30E-01	5.10E-01	1.70E-02
194	GOTERM_MF_DIRECT	signaling adaptor activity	1.7	4.20E-02	13	0.2	1.00E+00	5.30E-01	5.10E-01	1.70E-02
195	GOTERM_MF_DIRECT	cysteine-type endopeptidase activity involved in execution phase of apoptosis	2.3	4.60E-02	7	0.1	1.00E+00	5.60E-01	5.40E-01	1.00E-02
196	GOTERM_MF_DIRECT	phosphatidylinositol phosphate binding	1.8	4.60E-02	11	0.2	1.00E+00	5.60E-01	5.40E-01	1.60E-02
197	GOTERM_MF_DIRECT	cAMP binding	1.8	4.60E-02	11	0.2	1.00E+00	5.60E-01	5.40E-01	1.60E-02
198	GOTERM_MF_DIRECT	histone acetyltransferase binding	1.8	4.60E-02	11	0.2	1.00E+00	5.60E-01	5.40E-01	1.60E-02
199	GOTERM_MF_DIRECT	clathrin binding	1.5	4.70E-02	18	0.3	1.00E+00	5.70E-01	5.50E-01	2.30E-02
200	GOTERM_MF_DIRECT	syntaxin binding	1.4	4.80E-02	24	0.4	1.00E+00	5.80E-01	5.60E-01	2.60E-02
201	GOTERM_MF_DIRECT	steroid hormone receptor activity	2	4.80E-02	9	0.1	1.00E+00	5.80E-01	5.60E-01	1.50E-02
202	GOTERM_MF_DIRECT	BMP binding	2	4.80E-02	9	0.1	1.00E+00	5.80E-01	5.60E-01	1.50E-02

Table S3A. (A) KEGG pathway enrichment analyses based on DE miRNAs in the serum of LSDV-infected bovines compared to their expression in the non-infected controls. Criteria: P < 0.05, number of genes in each pathway term >5, and fold change of Log2-treatment/control ≥ 2.

No	Category	Term	Fold Enri	P-Value	Count	%	Bonferron	Benjamin	FDR	Fisher Exact
1	KEGG_PATHWAY	Focal adhesion	1.9	8.30E-19	127	1.9	2.90E-16	1.40E-16	9.80E-17	2.40E-19
2	KEGG_PATHWAY	MAPK signaling pathway	1.7	4.90E-17	166	2.5	1.70E-14	5.30E-15	3.60E-15	1.90E-17
3	KEGG_PATHWAY	Axon guidance	2	6.00E-17	112	1.7	3.90E-14	5.30E-15	3.60E-15	1.70E-17
4	KEGG_PATHWAY	Signaling pathways regulating pluripotency of stem cells	2	8.60E-15	92	1.4	3.00E-12	5.00E-13	3.40E-13	2.30E-15
5	KEGG_PATHWAY	PI3K-Akt signaling pathway	1.6	2.60E-14	195	2.9	9.10E-12	1.30E-12	8.80E-13	1.20E-14
6	KEGG_PATHWAY	Endocytosis	1.7	1.70E-13	135	2	5.90E-11	7.40E-12	5.00E-12	6.60E-14
7	KEGG_PATHWAY	Rap1 signaling pathway	1.7	1.30E-12	120	1.8	4.70E-10	5.20E-11	3.50E-11	5.00E-13
8	KEGG_PATHWAY	Regulation of actin cytoskeleton	1.7	4.00E-12	124	1.8	1.40E-09	1.30E-10	8.70E-11	1.60E-12
9	KEGG_PATHWAY	Hippo signaling pathway	1.8	3.70E-11	91	1.3	1.30E-08	1.10E-09	7.30E-10	1.30E-11
10	KEGG_PATHWAY	Ras signaling pathway	1.6	6.90E-11	128	1.9	2.40E-08	1.90E-09	1.30E-09	3.00E-11
11	KEGG_PATHWAY	Calcium signaling pathway	1.6	1.00E-10	135	2	3.50E-08	2.50E-09	1.70E-09	4.50E-11
12	KEGG_PATHWAY	Wnt signaling pathway	1.7	1.50E-10	98	1.4	5.10E-08	3.40E-09	2.30E-09	5.40E-11
13	KEGG_PATHWAY	Neurotrophin signaling pathway	1.9	2.70E-10	74	1.1	9.30E-08	5.80E-09	3.90E-09	8.30E-11
14	KEGG_PATHWAY	ErbB signaling pathway	2.1	3.70E-10	56	0.8	1.30E-07	7.60E-09	5.10E-09	8.90E-11
15	KEGG_PATHWAY	FoxO signaling pathway	1.8	8.70E-10	77	1.1	3.10E-07	1.60E-08	1.10E-08	2.90E-10
16	KEGG_PATHWAY	Thyroid hormone signaling pathway	1.8	1.60E-09	72	1.1	5.50E-07	2.80E-08	1.90E-08	5.10E-10
17	KEGG_PATHWAY	mTOR signaling pathway	1.7	2.00E-09	88	1.3	7.10E-07	3.40E-08	2.30E-08	7.60E-10
18	KEGG_PATHWAY	TGF-beta signaling pathway	1.8	1.50E-07	62	0.9	5.40E-05	1.70E-06	1.20E-06	5.40E-08
19	KEGG_PATHWAY	Melanogenesis	1.8	7.70E-08	60	0.9	2.70E-05	9.30E-07	6.30E-07	2.60E-08
20	KEGG_PATHWAY	Longevity regulating pathway	1.9	1.50E-08	56	0.8	5.20E-06	2.00E-07	1.40E-07	4.40E-09
21	KEGG_PATHWAY	Insulin signaling pathway	1.7	3.40E-07	74	1.1	1.20E-04	3.30E-06	2.20E-06	1.40E-07
22	KEGG_PATHWAY	Adherens junction	1.8	4.20E-07	54	0.8	1.50E-04	3.80E-06	2.60E-06	1.40E-07
23	KEGG_PATHWAY	Sphingolipid signaling pathway	1.7	4.30E-07	67	1	1.50E-04	3.80E-06	2.60E-06	1.70E-07
24	KEGG_PATHWAY	Phospholipase D signaling pathway	1.6	7.00E-07	79	1.2	2.50E-04	6.10E-06	4.10E-06	3.10E-07
25	KEGG_PATHWAY	Relaxin signaling pathway	1.7	8.90E-07	70	1	3.10E-04	7.60E-06	5.10E-06	3.70E-07
26	KEGG_PATHWAY	Autophagy - animal	1.6	9.40E-07	86	1.3	3.30E-04	7.80E-06	5.30E-06	4.30E-07
27	KEGG_PATHWAY	Cellular senescence	1.6	2.00E-06	81	1.2	6.80E-04	1.50E-05	1.00E-05	9.00E-07
28	KEGG_PATHWAY	Oxytocin signaling pathway	1.6	2.20E-06	78	1.2	7.70E-04	1.70E-05	1.10E-05	1.00E-06
29	KEGG_PATHWAY	Cholinergic synapse	1.7	4.70E-06	61	0.9	1.60E-03	3.40E-05	2.30E-05	1.90E-06
30	KEGG_PATHWAY	Parathyroid hormone synthesis, secretion and action	1.7	5.60E-06	57	0.8	2.00E-03	4.00E-05	2.70E-05	2.30E-06
31	KEGG_PATHWAY	cGMP-PKG signaling pathway	1.5	5.70E-06	84	1.2	2.00E-03	4.00E-05	2.70E-05	2.80E-06
32	KEGG_PATHWAY	ECM-receptor interaction	1.7	6.70E-06	50	0.7	2.30E-03	4.60E-05	3.10E-05	2.50E-06
33	KEGG_PATHWAY	AMPK signaling pathway	1.6	8.70E-06	64	0.9	3.10E-03	5.60E-05	3.70E-05	3.80E-06
34	KEGG_PATHWAY	cAMP signaling pathway	1.4	8.90E-06	113	1.7	3.10E-03	5.60E-05	3.80E-05	5.00E-06
35	KEGG_PATHWAY	Fc gamma R-mediated phagocytosis	1.7	1.00E-05	53	0.8	3.50E-03	6.00E-05	4.00E-05	3.90E-06
36	KEGG_PATHWAY	T cell receptor signaling pathway	1.6	1.20E-05	64	0.9	4.30E-03	7.10E-05	4.80E-05	5.40E-06
37	KEGG_PATHWAY	Platelet activation	1.6	1.40E-05	63	0.9	4.90E-03	8.10E-05	5.50E-05	6.30E-06
38	KEGG_PATHWAY	Tight junction	1.5	1.90E-05	85	1.3	6.80E-03	1.10E-04	7.40E-05	1.00E-05

39	KEGG_PATHWAY	Adipocytokine signaling pathway	1.8	2.30E-05	41	0.6	7.90E-03	1.20E-04	8.20E-05	7.90E-06
40	KEGG_PATHWAY	Circadian rhythm	2.1	4.80E-05	24	0.4	1.70E-02	2.50E-04	1.70E-04	1.10E-05
41	KEGG_PATHWAY	Dopaminergic synapse	1.5	5.80E-05	66	1	2.00E-02	3.00E-04	2.00E-04	2.80E-05
42	KEGG_PATHWAY	Efferocytosis	1.5	7.80E-05	77	1.1	2.70E-02	4.00E-04	2.70E-04	4.10E-05
43	KEGG_PATHWAY	Endocrine and other factor-regulated calcium reabsorption	1.9	8.00E-05	31	0.5	2.80E-02	4.00E-04	2.70E-04	2.50E-05
44	KEGG_PATHWAY	Leukocyte transendothelial migration	1.6	1.00E-04	58	0.9	3.60E-02	5.10E-04	3.50E-04	4.90E-05
45	KEGG_PATHWAY	Adrenergic signaling in cardiomyocytes	1.5	1.10E-04	73	1.1	3.70E-02	5.20E-04	3.50E-04	5.60E-05
46	KEGG_PATHWAY	Polycomb repressive complex	1.7	1.10E-04	44	0.7	3.90E-02	5.40E-04	3.60E-04	4.60E-05
47	KEGG_PATHWAY	Glycosaminoglycan biosynthesis - heparan sulfate / heparin	2.3	1.20E-04	18	0.3	4.10E-02	5.60E-04	3.80E-04	2.10E-05
48	KEGG_PATHWAY	HIF-1 signaling pathway	1.6	1.60E-04	55	0.8	5.50E-02	7.60E-04	5.10E-04	7.60E-05
49	KEGG_PATHWAY	Aldosterone-regulated sodium reabsorption	2	1.70E-04	24	0.4	5.80E-02	7.90E-04	5.30E-04	4.60E-05
50	KEGG_PATHWAY	Chemokine signaling pathway	1.4	1.80E-04	86	1.3	6.20E-02	8.40E-04	5.60E-04	1.00E-04
51	KEGG_PATHWAY	p53 signaling pathway	1.6	1.90E-04	43	0.6	6.30E-02	8.40E-04	5.70E-04	7.80E-05
52	KEGG_PATHWAY	Glutamatergic synapse	1.5	2.50E-04	56	0.8	8.40E-02	1.10E-03	7.30E-04	1.20E-04
53	KEGG_PATHWAY	Insulin secretion	1.6	3.20E-04	44	0.7	1.10E-01	1.40E-03	9.10E-04	1.40E-04
54	KEGG_PATHWAY	Longevity regulating pathway - multiple species	1.7	3.30E-04	34	0.5	1.10E-01	1.40E-03	9.20E-04	1.20E-04
55	KEGG_PATHWAY	Apoptosis - multiple species	2	3.70E-04	22	0.3	1.20E-01	1.50E-03	1.00E-03	1.00E-04
56	KEGG_PATHWAY	Gastric acid secretion	1.6	4.10E-04	40	0.6	1.30E-01	1.70E-03	1.10E-03	1.80E-04
57	KEGG_PATHWAY	Aldosterone synthesis and secretion	1.6	4.50E-04	48	0.7	1.50E-01	1.80E-03	1.20E-03	2.10E-04
58	KEGG_PATHWAY	Phosphatidylinositol signaling system	1.5	6.10E-04	48	0.7	1.90E-01	2.40E-03	1.60E-03	2.90E-04
59	KEGG_PATHWAY	SNARE interactions in vesicular transport	2	7.30E-04	21	0.3	2.30E-01	2.80E-03	1.90E-03	2.10E-04
60	KEGG_PATHWAY	Hedgehog signaling pathway	1.7	7.50E-04	31	0.5	2.30E-01	2.80E-03	1.90E-03	2.90E-04
61	KEGG_PATHWAY	Estrogen signaling pathway	1.4	9.10E-04	63	0.9	2.70E-01	3.40E-03	2.30E-03	5.00E-04
62	KEGG_PATHWAY	Circadian entrainment	1.5	9.20E-04	49	0.7	2.80E-01	3.40E-03	2.30E-03	4.60E-04
63	KEGG_PATHWAY	Growth hormone synthesis, secretion and action	1.5	9.40E-04	56	0.8	2.80E-01	3.50E-03	2.30E-03	5.00E-04
64	KEGG_PATHWAY	C-type lectin receptor signaling pathway	1.5	1.30E-03	50	0.7	3.80E-01	4.80E-03	3.20E-03	6.90E-04
65	KEGG_PATHWAY	TNF signaling pathway	1.4	1.50E-03	58	0.9	4.00E-01	5.10E-03	3.40E-03	7.90E-04
66	KEGG_PATHWAY	VEGF signaling pathway	1.7	1.60E-03	31	0.5	4.20E-01	5.40E-03	3.70E-03	6.50E-04
67	KEGG_PATHWAY	Long-term potentiation	1.6	2.30E-03	35	0.5	5.60E-01	7.70E-03	5.20E-03	1.10E-03
68	KEGG_PATHWAY	Fc epsilon RI signaling pathway	1.6	3.10E-03	35	0.5	6.70E-01	1.00E-02	6.90E-03	1.50E-03
69	KEGG_PATHWAY	Synaptic vesicle cycle	1.5	3.50E-03	38	0.6	7.10E-01	1.10E-02	7.50E-03	1.70E-03
70	KEGG_PATHWAY	Apelin signaling pathway	1.4	3.70E-03	62	0.9	7.30E-01	1.20E-02	7.80E-03	2.20E-03
71	KEGG_PATHWAY	GnRH signaling pathway	1.4	4.70E-03	43	0.6	8.10E-01	1.50E-02	9.90E-03	2.50E-03
72	KEGG_PATHWAY	GnRH secretion	1.6	4.90E-03	32	0.5	8.20E-01	1.50E-02	1.00E-02	2.30E-03
73	KEGG_PATHWAY	Apoptosis	1.3	5.40E-03	62	0.9	8.50E-01	1.60E-02	1.10E-02	3.30E-03
74	KEGG_PATHWAY	Mitophagy - animal	1.4	6.30E-03	49	0.7	8.90E-01	1.90E-02	1.30E-02	3.60E-03
75	KEGG_PATHWAY	Long-term depression	1.5	8.90E-03	30	0.4	9.60E-01	2.60E-02	1.80E-02	4.30E-03
76	KEGG_PATHWAY	Glycosaminoglycan biosynthesis - keratan sulfate	2.2	1.40E-02	10	0.1	9.90E-01	4.00E-02	2.70E-02	3.00E-03
77	KEGG_PATHWAY	Mucin type O-glycan biosynthesis	1.7	1.40E-02	19	0.3	9.90E-01	4.10E-02	2.80E-02	5.70E-03
78	KEGG_PATHWAY	Other types of O-glycan biosynthesis	1.6	1.50E-02	23	0.3	9.90E-01	4.20E-02	2.80E-02	6.50E-03
79	KEGG_PATHWAY	Sphingolipid metabolism	1.5	1.60E-02	28	0.4	1.00E+00	4.40E-02	3.00E-02	7.80E-03
80	KEGG_PATHWAY	Inositol phosphate metabolism	1.4	2.00E-02	33	0.5	1.00E+00	5.50E-02	3.70E-02	1.10E-02

81	KEGG_PATHWAY	Protein digestion and absorption	1.3	2.10E-02	54	0.8	1.00E+00	5.70E-02	3.80E-02	1.30E-02
82	KEGG_PATHWAY	GABAergic synapse	1.4	2.10E-02	40	0.6	1.00E+00	5.80E-02	3.90E-02	1.20E-02
83	KEGG_PATHWAY	Prolactin signaling pathway	1.4	2.10E-02	37	0.5	1.00E+00	5.80E-02	3.90E-02	1.20E-02
84	KEGG_PATHWAY	Vasopressin-regulated water reabsorption	1.5	2.20E-02	24	0.4	1.00E+00	5.90E-02	4.00E-02	1.10E-02
85	KEGG_PATHWAY	Hippo signaling pathway - multiple species	1.7	2.30E-02	16	0.2	1.00E+00	6.00E-02	4.00E-02	8.80E-03
86	KEGG_PATHWAY	Mineral absorption	1.4	2.60E-02	28	0.4	1.00E+00	6.60E-02	4.50E-02	1.40E-02
87	KEGG_PATHWAY	Notch signaling pathway	1.4	2.60E-02	28	0.4	1.00E+00	6.60E-02	4.50E-02	1.40E-02
88	KEGG_PATHWAY	Glucagon signaling pathway	1.3	2.60E-02	44	0.7	1.00E+00	6.70E-02	4.50E-02	1.60E-02
89	KEGG_PATHWAY	Gap junction	1.3	2.90E-02	39	0.6	1.00E+00	7.30E-02	4.90E-02	1.70E-02
90	KEGG_PATHWAY	Nucleotide metabolism	1.3	3.10E-02	37	0.5	1.00E+00	7.90E-02	5.30E-02	1.90E-02
91	KEGG_PATHWAY	Osteoclast differentiation	1.3	3.60E-02	57	0.8	1.00E+00	8.80E-02	6.00E-02	2.40E-02
92	KEGG_PATHWAY	Autophagy - other	1.6	3.80E-02	17	0.3	1.00E+00	9.30E-02	6.30E-02	1.70E-02
93	KEGG_PATHWAY	Ubiquitin mediated proteolysis	1.2	3.90E-02	60	0.9	1.00E+00	9.40E-02	6.40E-02	2.60E-02
94	KEGG_PATHWAY	Glycosaminoglycan biosynthesis - chondroitin sulfate / dermatan sulfate	1.8	4.40E-02	12	0.2	1.00E+00	1.10E-01	7.20E-02	1.60E-02
95	KEGG_PATHWAY	Vascular smooth muscle contraction	1.2	4.50E-02	56	0.8	1.00E+00	1.10E-01	7.30E-02	3.10E-02

Table S3B. (B) Reactome pathway enrichment analyses based on DE miRNAs in the serum of LSDV-infected bovines compared to their expression in the non-infected controls. Criteria: P < 0.05, number of genes in each pathway term >5, and fold change of Log2-treatment/control ≥ 2.

No	Category	Term	Fold Enri	P-Value	Count	%	Bonferroni	Benjamini	FDR	Fisher Exact
1	REACTOME_PATHWAY	Signal Transduction	1.2	4.30E-12	656	9.7	6.60E-09	6.60E-09	6.50E-09	3.10E-12
2	REACTOME_PATHWAY	Axon guidance	1.6	5.30E-07	78	1.2	8.10E-04	4.10E-04	4.00E-04	2.10E-07
3	REACTOME_PATHWAY	Nervous system development	1.6	8.40E-07	78	1.2	1.30E-03	4.30E-04	4.20E-04	3.40E-07
4	REACTOME_PATHWAY	RHO GTPase cycle	1.4	2.80E-06	125	1.8	4.30E-03	1.10E-03	1.10E-03	1.50E-06
5	REACTOME_PATHWAY	Neuronal System	1.4	9.30E-06	111	1.6	1.40E-02	2.40E-03	2.40E-03	5.00E-06
6	REACTOME_PATHWAY	MAPK1/MAPK3 signaling	1.5	9.40E-06	82	1.2	1.40E-02	2.40E-03	2.40E-03	4.40E-06
7	REACTOME_PATHWAY	RAF/MAP kinase cascade	1.5	2.20E-05	78	1.2	3.30E-02	4.80E-03	4.70E-03	1.00E-05
8	REACTOME_PATHWAY	Membrane Trafficking	1.3	4.30E-05	175	2.6	6.40E-02	8.30E-03	8.10E-03	2.80E-05
9	REACTOME_PATHWAY	MAPK family signaling cascades	1.3	1.10E-04	101	1.5	1.50E-01	1.50E-02	1.50E-02	6.10E-05
10	REACTOME_PATHWAY	Signaling by Receptor Tyrosine Kinases	1.3	1.10E-04	135	2	1.60E-01	1.50E-02	1.50E-02	7.10E-05
11	REACTOME_PATHWAY	PI5P, PP2A and IER3 Regulate PI3K/AKT Signaling	1.6	1.20E-04	45	0.7	1.70E-01	1.50E-02	1.50E-02	4.60E-05
12	REACTOME_PATHWAY	Negative regulation of the PI3K/AKT network	1.6	1.20E-04	45	0.7	1.70E-01	1.50E-02	1.50E-02	4.60E-05
13	REACTOME_PATHWAY	Developmental Biology	1.3	2.60E-04	114	1.7	3.30E-01	2.90E-02	2.80E-02	1.60E-04
14	REACTOME_PATHWAY	RAC1 GTPase cycle	1.5	2.80E-04	51	0.8	3.40E-01	2.90E-02	2.80E-02	1.20E-04
15	REACTOME_PATHWAY	Vesicle-mediated transport	1.2	2.80E-04	176	2.6	3.50E-01	2.90E-02	2.80E-02	1.90E-04
16	REACTOME_PATHWAY	Neurotransmitter receptors and postsynaptic signal	1.5	3.80E-04	54	0.8	4.40E-01	3.60E-02	3.50E-02	1.70E-04
17	REACTOME_PATHWAY	Cardiac conduction	1.6	4.80E-04	40	0.6	5.20E-01	4.30E-02	4.20E-02	1.90E-04
18	REACTOME_PATHWAY	Potassium Channels	1.6	7.50E-04	39	0.6	6.80E-01	6.30E-02	6.20E-02	3.00E-04
19	REACTOME_PATHWAY	Clathrin-mediated endocytosis	1.5	8.20E-04	52	0.8	7.10E-01	6.60E-02	6.50E-02	3.90E-04
20	REACTOME_PATHWAY	VEGFA-VEGFR2 Pathway	1.6	1.20E-03	30	0.4	8.30E-01	8.30E-02	8.10E-02	4.20E-04
21	REACTOME_PATHWAY	Signaling by NTRKs	1.6	1.20E-03	31	0.5	8.40E-01	8.30E-02	8.10E-02	4.30E-04
22	REACTOME_PATHWAY	MET promotes cell motility	2.1	1.20E-03	15	0.2	8.40E-01	8.30E-02	8.10E-02	2.00E-04
23	REACTOME_PATHWAY	Semaphorin interactions	1.9	1.20E-03	20	0.3	8.50E-01	8.30E-02	8.20E-02	3.10E-04
24	REACTOME_PATHWAY	RHOF GTPase cycle	2	1.50E-03	16	0.2	9.00E-01	9.20E-02	9.00E-02	2.90E-04
25	REACTOME_PATHWAY	RHOQ GTPase cycle	1.8	1.50E-03	22	0.3	9.00E-01	9.20E-02	9.00E-02	4.30E-04
26	REACTOME_PATHWAY	CDC42 GTPase cycle	1.7	1.80E-03	26	0.4	9.40E-01	9.60E-02	9.40E-02	6.10E-04
27	REACTOME_PATHWAY	RAB geranylgeranylation	1.7	1.80E-03	26	0.4	9.40E-01	9.60E-02	9.40E-02	6.10E-04
28	REACTOME_PATHWAY	Assembly of collagen fibrils and other multimeric structures	2.2	1.80E-03	13	0.2	9.40E-01	9.60E-02	9.40E-02	2.60E-04
29	REACTOME_PATHWAY	RAC2 GTPase cycle	1.6	1.90E-03	28	0.4	9.40E-01	9.60E-02	9.40E-02	6.70E-04
30	REACTOME_PATHWAY	Signaling by VEGF	1.6	1.90E-03	33	0.5	9.40E-01	9.60E-02	9.40E-02	7.60E-04
31	REACTOME_PATHWAY	RHOC GTPase cycle	1.8	2.60E-03	21	0.3	9.80E-01	1.30E-01	1.30E-01	7.80E-04
32	REACTOME_PATHWAY	Fcgamma receptor (FCGR) dependent phagocytosis	1.5	2.90E-03	32	0.5	9.90E-01	1.40E-01	1.40E-01	1.20E-03
33	REACTOME_PATHWAY	Platelet activation, signaling and aggregation	1.3	3.10E-03	78	1.2	9.90E-01	1.40E-01	1.40E-01	1.90E-03
34	REACTOME_PATHWAY	Transmission across Chemical Synapses	1.3	3.20E-03	68	1	9.90E-01	1.40E-01	1.40E-01	1.80E-03
35	REACTOME_PATHWAY	RAC3 GTPase cycle	1.5	4.50E-03	31	0.5	1.00E+00	2.00E-01	1.90E-01	1.90E-03
36	REACTOME_PATHWAY	Signaling by MET	1.6	4.70E-03	28	0.4	1.00E+00	2.00E-01	1.90E-01	1.90E-03
37	REACTOME_PATHWAY	Signaling by NTRK1 (TRKA)	1.6	4.80E-03	27	0.4	1.00E+00	2.00E-01	1.90E-01	1.90E-03
38	REACTOME_PATHWAY	FCERI mediated MAPK activation	2	5.40E-03	14	0.2	1.00E+00	2.20E-01	2.10E-01	1.20E-03
39	REACTOME_PATHWAY	RHOA GTPase cycle	1.5	5.60E-03	36	0.5	1.00E+00	2.20E-01	2.10E-01	2.70E-03
40	REACTOME_PATHWAY	MET activates PTK2 signaling	2.3	5.70E-03	10	0.1	1.00E+00	2.20E-01	2.10E-01	7.00E-04
41	REACTOME_PATHWAY	Hemostasis	1.2	6.40E-03	145	2.1	1.00E+00	2.40E-01	2.30E-01	4.70E-03

42	REACTOME_PATHWAY	Signaling by Rho GTPases, Miro GTPases and RHOBTB3	1.2	7.00E-03	181	2.7	1.00E+00	2.50E-01	2.40E-01	5.30E-03
43	REACTOME_PATHWAY	Extra-nuclear estrogen signaling	1.5	7.20E-03	28	0.4	1.00E+00	2.50E-01	2.40E-01	3.00E-03
44	REACTOME_PATHWAY	Signaling by PDGF	1.7	7.30E-03	18	0.3	1.00E+00	2.50E-01	2.40E-01	2.30E-03
45	REACTOME_PATHWAY	RHOD GTPase cycle	1.7	7.30E-03	18	0.3	1.00E+00	2.50E-01	2.40E-01	2.30E-03
46	REACTOME_PATHWAY	Ca2+ pathway	1.6	7.80E-03	21	0.3	1.00E+00	2.60E-01	2.50E-01	2.80E-03
47	REACTOME_PATHWAY	L1CAM interactions	2	8.90E-03	12	0.2	1.00E+00	2.90E-01	2.80E-01	1.80E-03
48	REACTOME_PATHWAY	Opioid Signalling	1.5	9.60E-03	31	0.5	1.00E+00	3.00E-01	3.00E-01	4.40E-03
49	REACTOME_PATHWAY	Signaling by Rho GTPases	1.2	9.90E-03	176	2.6	1.00E+00	3.10E-01	3.00E-01	7.60E-03
50	REACTOME_PATHWAY	Activation of NMDA receptors and postsynaptic events	1.9	1.00E-02	13	0.2	1.00E+00	3.10E-01	3.00E-01	2.40E-03
51	REACTOME_PATHWAY	Signaling by TGFB family members	1.4	1.00E-02	37	0.5	1.00E+00	3.10E-01	3.10E-01	5.30E-03
52	REACTOME_PATHWAY	Regulation of actin dynamics for phagocytic cup formation	1.5	1.10E-02	25	0.4	1.00E+00	3.20E-01	3.20E-01	4.90E-03
53	REACTOME_PATHWAY	RND3 GTPase cycle	1.8	1.20E-02	15	0.2	1.00E+00	3.20E-01	3.20E-01	3.30E-03
54	REACTOME_PATHWAY	Disassembly of the destruction complex and recruitment of AXIN to the membrane	1.8	1.20E-02	15	0.2	1.00E+00	3.20E-01	3.20E-01	3.30E-03
55	REACTOME_PATHWAY	PI3K events in ERBB2 signaling	2.3	1.20E-02	9	0.1	1.00E+00	3.20E-01	3.20E-01	1.60E-03
56	REACTOME_PATHWAY	N-Glycan antennae elongation	2.3	1.20E-02	9	0.1	1.00E+00	3.20E-01	3.20E-01	1.60E-03
57	REACTOME_PATHWAY	RHOU GTPase cycle	1.7	1.20E-02	16	0.2	1.00E+00	3.20E-01	3.20E-01	3.70E-03
58	REACTOME_PATHWAY	Intracellular signaling by second messengers	1.2	1.40E-02	76	1.1	1.00E+00	3.80E-01	3.70E-01	9.50E-03
59	REACTOME_PATHWAY	CRMPs in Sema3A signaling	2.1	1.50E-02	10	0.1	1.00E+00	3.80E-01	3.70E-01	2.70E-03
60	REACTOME_PATHWAY	Extracellular matrix organization	1.3	1.60E-02	58	0.9	1.00E+00	4.20E-01	4.10E-01	1.00E-02
61	REACTOME_PATHWAY	CD28 co-stimulation	2	1.70E-02	11	0.2	1.00E+00	4.20E-01	4.10E-01	3.70E-03
62	REACTOME_PATHWAY	Non-integrin membrane-ECM interactions	1.9	1.80E-02	12	0.2	1.00E+00	4.50E-01	4.40E-01	4.60E-03
63	REACTOME_PATHWAY	Ion homeostasis	1.6	2.00E-02	18	0.3	1.00E+00	4.70E-01	4.70E-01	7.60E-03
64	REACTOME_PATHWAY	G-protein mediated events	1.6	2.00E-02	18	0.3	1.00E+00	4.70E-01	4.70E-01	7.60E-03
65	REACTOME_PATHWAY	Inwardly rectifying K+ channels	1.6	2.00E-02	17	0.3	1.00E+00	4.70E-01	4.70E-01	7.40E-03
66	REACTOME_PATHWAY	RND1 GTPase cycle	1.7	2.00E-02	16	0.2	1.00E+00	4.70E-01	4.70E-01	7.00E-03
67	REACTOME_PATHWAY	Rab regulation of trafficking	1.3	2.30E-02	38	0.6	1.00E+00	5.30E-01	5.20E-01	1.30E-02
68	REACTOME_PATHWAY	Post NMDA receptor activation events	2.2	2.40E-02	8	0.1	1.00E+00	5.30E-01	5.20E-01	3.70E-03
69	REACTOME_PATHWAY	VEGFR2 mediated vascular permeability	2.2	2.40E-02	8	0.1	1.00E+00	5.30E-01	5.20E-01	3.70E-03
70	REACTOME_PATHWAY	Reduction of cytosolic Ca++ levels	2.2	2.40E-02	8	0.1	1.00E+00	5.30E-01	5.20E-01	3.70E-03
71	REACTOME_PATHWAY	Muscle contraction	1.3	2.60E-02	53	0.8	1.00E+00	5.60E-01	5.60E-01	1.60E-02
72	REACTOME_PATHWAY	Cargo recognition for clathrin-mediated endocytosis	1.4	2.70E-02	35	0.5	1.00E+00	5.70E-01	5.60E-01	1.50E-02
73	REACTOME_PATHWAY	O-linked glycosylation	1.4	2.80E-02	29	0.4	1.00E+00	5.80E-01	5.70E-01	1.40E-02
74	REACTOME_PATHWAY	Netrin-1 signaling	2.1	2.80E-02	9	0.1	1.00E+00	5.80E-01	5.70E-01	5.70E-03
75	REACTOME_PATHWAY	Collagen chain trimerization	2.1	2.80E-02	9	0.1	1.00E+00	5.80E-01	5.70E-01	5.70E-03
76	REACTOME_PATHWAY	Integration of energy metabolism	1.4	2.90E-02	28	0.4	1.00E+00	5.80E-01	5.70E-01	1.50E-02
77	REACTOME_PATHWAY	Collagen formation	1.6	3.10E-02	18	0.3	1.00E+00	6.10E-01	6.00E-01	1.30E-02
78	REACTOME_PATHWAY	Aquaporin-mediated transport	1.6	3.20E-02	17	0.3	1.00E+00	6.10E-01	6.00E-01	1.30E-02
79	REACTOME_PATHWAY	Collagen degradation	1.8	3.20E-02	11	0.2	1.00E+00	6.10E-01	6.00E-01	8.90E-03
80	REACTOME_PATHWAY	RET signaling	1.6	3.30E-02	15	0.2	1.00E+00	6.10E-01	6.00E-01	1.20E-02
81	REACTOME_PATHWAY	RHOV GTPase cycle	1.6	3.30E-02	15	0.2	1.00E+00	6.10E-01	6.00E-01	1.20E-02
82	REACTOME_PATHWAY	PLC beta mediated events	1.6	3.30E-02	15	0.2	1.00E+00	6.10E-01	6.00E-01	1.20E-02
83	REACTOME_PATHWAY	Nuclear Events (kinase and transcription factor activation)	1.8	3.30E-02	12	0.2	1.00E+00	6.10E-01	6.00E-01	1.00E-02
84	REACTOME_PATHWAY	Downstream signal transduction	1.7	3.40E-02	13	0.2	1.00E+00	6.10E-01	6.00E-01	1.10E-02
85	REACTOME_PATHWAY	GABA receptor activation	1.4	3.70E-02	23	0.3	1.00E+00	6.60E-01	6.50E-01	1.80E-02
86	REACTOME_PATHWAY	PIP3 activates AKT signaling	1.2	3.80E-02	67	1	1.00E+00	6.70E-01	6.60E-01	2.60E-02

87	REACTOME_PATHWAY	Ionotropic activity of kainate receptors	2.5	4.00E-02	6	0.1	1.00E+00	6.80E-01	6.70E-01	4.00E-03
88	REACTOME_PATHWAY	Activation of Ca-permeable Kainate Receptor	2.5	4.00E-02	6	0.1	1.00E+00	6.80E-01	6.70E-01	4.00E-03
89	REACTOME_PATHWAY	MET receptor recycling	2.5	4.00E-02	6	0.1	1.00E+00	6.80E-01	6.70E-01	4.00E-03
90	REACTOME_PATHWAY	Linoleic acid (LA) metabolism	2.5	4.00E-02	6	0.1	1.00E+00	6.80E-01	6.70E-01	4.00E-03
91	REACTOME_PATHWAY	Glycosaminoglycan metabolism	1.3	4.20E-02	40	0.6	1.00E+00	7.10E-01	7.00E-01	2.60E-02
92	REACTOME_PATHWAY	Degradation of the extracellular matrix	1.4	4.30E-02	26	0.4	1.00E+00	7.10E-01	7.00E-01	2.20E-02
93	REACTOME_PATHWAY	Integrin cell surface interactions	1.5	4.40E-02	19	0.3	1.00E+00	7.10E-01	7.00E-01	2.00E-02
94	REACTOME_PATHWAY	Signal amplification	1.5	4.70E-02	17	0.3	1.00E+00	7.10E-01	7.00E-01	2.00E-02
95	REACTOME_PATHWAY	Platelet homeostasis	1.3	4.80E-02	29	0.4	1.00E+00	7.10E-01	7.00E-01	2.60E-02
96	REACTOME_PATHWAY	GRB2 events in ERBB2 signaling	2.2	4.90E-02	7	0.1	1.00E+00	7.10E-01	7.00E-01	8.20E-03
97	REACTOME_PATHWAY	CD28 dependent Vav1 pathway	2.2	4.90E-02	7	0.1	1.00E+00	7.10E-01	7.00E-01	8.20E-03
98	REACTOME_PATHWAY	Synthesis of pyrophosphates in the cytosol	2.2	4.90E-02	7	0.1	1.00E+00	7.10E-01	7.00E-01	8.20E-03
99	REACTOME_PATHWAY	Crosslinking of collagen fibrils	2.2	4.90E-02	7	0.1	1.00E+00	7.10E-01	7.00E-01	8.20E-03
100	REACTOME_PATHWAY	Sphingolipid de novo biosynthesis	1.5	4.90E-02	16	0.2	1.00E+00	7.10E-01	7.00E-01	2.00E-02

Table S4: Identification of novel bovine miRNAs based on seed sequence alignments.

ID	Mature_ID	miRNA	Chr	Start	End	Strand	Length	Read_Count							Precursor				Sequences	
								Total count	Control-01	Control-02	Control-03	HT10-01	HT10-02	HT10-03	ID	Located	Length	Total_Count	miRNA sequence	Precursor sequence
1	Mature_1	bta-miR-1_20	chr1	2898457	2898476	+	20	10	10	0	0	0	0	0	Precursor_1	chr1_20|chr1:2898457-2898508:+	52	10	AGAGGAGCGCATCAGCAGGG	AGAGGAGCGCATCAGCAGGGAAAAGTGTCAGGTTGTTGGTGTGTTATCCACA
2	Mature_2	bta-miR-1_1311	chr1	4020696	4020716	-	21	9	0	0	0	9	0	0	Precursor_2	chr1_1311|chr1:4020696-4020782:-	87	15	TCTCACAGTTCTGGAGGCTGG	CTGTCCTCTAGGGCTGTCATAATAAAGAACCATAGGCTATGGGGCTTAAATGGCATAAATTTATTTTCTCACAGTTCTGGAGGCTGG
3	Mature_3	bta-miR-1_1374	chr1	7315468	7315490	-	23	23	0	0	23	0	0	0	Precursor_3	chr1_1374|chr1:7315468-7315516:-	49	23	CACACGAGTGGTGCAGCGTAGTA	CTATGCATGCATGCAGTGAACTAGATCACACGAGTGGTGCAGCGTAGTA
4	Mature_4	bta-miR-1_1291	chr1	22708039	22708057	-	19	15	15	0	0	0	0	0	Precursor_4	chr1_1291|chr1:22707998-22708057:-	60	15	GGAGGAAGGGGGAAAAGAG	GGAGGAAGGGGGAAAAGAGGAAACTCCATGAACTGTTCCTGCCTTCAAAACTTCTGCCAA
5	Mature_5	bta-miR-1_201	chr1	26097343	26097365	+	23	41	0	0	0	41	0	0	Precursor_5	chr1_201|chr1:26097286-26097365:+	80	43	TCCCCGCCGCTCCTTCCCCCTCC	GGCGGGGGGAGGGGGGGCGGTGAGGGGGGGGGAGGTGGGCGCCCACACCCCTTCCCCTCCCCGCCGCTCCTTCCCCCTCC
6	Mature_6	bta-miR-1_357	chr1	44594975	44594993	+	19	21	0	0	21	0	0	0	Precursor_6	chr1_357|chr1:44594939-44594993:+	55	21	CCAATTAGCTTGAATTTGT	AAATTCAAGCTAATTGTATATTTACAAATACAAGCCCCAATTAGCTTGAATTTGT
7	Mature_7	bta-miR-1_366	chr1	45260536	45260553	+	18	10	0	0	10	0	0	0	Precursor_7	chr1_366|chr1:45260517-45260553:+	37	10	GGGGGAGGAAAAGAAGGG	CTTTGTTTTCCCAGTGTTGGGGGAGGAAAAGAAGGG
8	Mature_8	bta-miR-1_1654	chr1	55641368	55641387	-	20	138	0	0	138	0	0	0	Precursor_8	chr1_1654|chr1:55641368-55641418:-	51	138	GGGGAGGGAGGAAAGGAAAG	TTTTATGACTGTCTTCCCAGTGTGCATATCGGGGGAGGGAGGAAAGGAAAG
9	Mature_9	bta-miR-1_1683	chr1	57415826	57415843	-	18	13	0	0	13	0	0	0	Precursor_9	chr1_1683|chr1:57415778-57415843:-	66	13	GAAGGGAAGAGAAGAAAG	GAAGGGAAGAGAAGAAAGCCTGAGTCCTGAAGACACAGCTCAGGGTTGGGCTGGCTTCCCCTTAAG
10	Mature_10	bta-miR-1_1702	chr1	59605835	59605857	-	23	13	0	0	13	0	0	0	Precursor_10	chr1_1702|chr1:59605835-59605882:-	48	13	GGCTCAAAAGTTTGGGAAGTGGG	CACTCTCCAAAGTTTTGAGAAATATGGCTCAAAAGTTTGGGAAGTGGG
11	Mature_11	bta-miR-1_529	chr1	68092318	68092335	+	18	11	0	0	11	0	0	0	Precursor_11	chr1_529|chr1:68092271-68092335:+	65	11	GGGAGAGGGAGAAAGGGC	CAGCCCCAGGGTCCTGGTTTGCACTTCGTAAAACAGGGGTGGGCATCGGGAGAGGGAGAAAGGGC
12	Mature_12	bta-miR-1_494	chr1	69827440	69827457	+	18	78	0	78	0	0	0	0	Precursor_12	chr1_494|chr1:69827440-69827522:+	83	78	AGGAGGGGAAGGGGGGAG	AGGAGGGGAAGGGGGGAGGGGGGAGAACTTTTGCTGTGGCAGCTTCGGCAACAGCTCCCACCCCTGCGCCTTTCTCCTCTTCC
13	Mature_13	bta-miR-1_551	chr1	70420824	70420843	+	20	12	0	0	12	0	0	0	Precursor_13	chr1_551|chr1:70420779-70420843:+	65	12	GCCGGGTGACAAAAAGAACC	TTTCAATCCCTGGTTGGGGACAGTTGGGGAACTAGGACCCCACATGCCGGGTGACAAAAAGAACC
14	Mature_14	bta-miR-1_623	chr1	79218921	79218938	+	18	26	0	0	26	0	0	0	Precursor_14	chr1_623|chr1:79218871-79218938:+	68	26	GGGAAAGAAAGGAAGAGG	GTTTCAGTTTTCTCACTTATGTCAAAGTAAGAAGTAAGGTGGGGGAGGGTGGGAAAGAAAGGAAGAGG
15	Mature_15	bta-miR-1_1708	chr1	80469837	80469854	-	18	127	44	0	83	0	0	0	Precursor_15	chr1_1708|chr1:80469837-80469897:-	61	127	AACTTCAGGCTATTTGGG	CAAAAGTTTGTTATTGCGGGAAGGTTTTTTAATGGCCTTTACCAACTTCAGGCTATTTGGG
16	Mature_16	bta-miR-1_1709	chr1	80470563	80470580	-	18	12	12	0	0	0	0	0	Precursor_16	chr1_1709|chr1:80470563-80470615:-	53	18	CACAAGAAGAAGAGGAAC	GCCTTGGTTTTTGTAATAATGCTAGCAGAGTATTACACAAGAAGAAGAGGAAC
17	Mature_17	bta-miR-1_1746	chr1	81068463	81068484	-	22	11	0	11	0	0	0	0	Precursor_17	chr1_1746|chr1:81068463-81068533:-	71	11	CCCATCCCCACTCCAATGATAC	GCTGGGCAGCTGGGGACTGAGTTCCTAAGGAATGTCCCTCCTATTCTTACCCATCCCCACTCCAATGATAC
18	Mature_18	bta-miR-1_600	chr1	82631541	82631560	+	20	97	0	81	16	0	0	0	Precursor_18	chr1_600|chr1:82631541-82631614:+	74	97	GGCCGCGCCGGGCCGCGCCG	GGCCGCGCCGGGCCGCGCCGGCGGGGCTGCGGGCACCCGCAGGAGGATCCGCTCGGCGGGCGCGAGAAGGCTCG
19	Mature_19	bta-miR-1_705	chr1	83235702	83235721	+	20	9	0	0	9	0	0	0	Precursor_19	chr1_705|chr1:83235673-83235721:+	49	9	GAAAAAAGAGGGGAGAGGGC	CCCTCCCTGTAGGCTTCAGTAGATCAACTGAAAAAAGAGGGGAGAGGGC
20	Mature_20	bta-miR-1_1798	chr1	88149314	88149336	-	23	25	0	25	0	0	0	0	Precursor_20	chr1_1798|chr1:88149314-88149385:-	72	30	CCTACATACTGTGCAGCATGGCC	CTTCTACTGCAGGGGGTGTGGGTTCAATCCCTGGTCAGGGAACTAAGATCCTACATACTGTGCAGCATGGCC
21	Mature_21	bta-miR-1_2030	chr1	88237554	88237573	-	20	15	0	0	15	0	0	0	Precursor_21	chr1_2030|chr1:88237554-88237628:-	75	15	CAACAGCAAGGAAAGGAAGC	TTCCATTCTGGCTGGCACACATTAGGCTCCCTCTGCATCCCGGAGATGCTAATGCCAACAGCAAGGAAAGGAAGC
22	Mature_22	bta-miR-1_672	chr1	94242427	94242445	+	19	562	0	365	0	197	0	0	Precursor_22	chr1_672|chr1:94242427-94242495:+	69	562	TGATTTTTGGAGAAGGGAG	TGATTTTTGGAGAAGGGAGAATACATGCAATCAAGCACATACTTTTCCAGAAGGTCTCCACTAATCTCA
23	Mature_23	bta-miR-1_678	chr1	103951619	103951637	+	19	444	158	0	286	0	0	0	Precursor_23	chr1_678|chr1:103951619-103951661:+	43	444	GAAGGAAGAAGGAAGAAGG	GAAGGAAGAAGGAAGAAGGAGCAACTTCACCTTCTTTCTTGGA
24	Mature_24	bta-miR-1_1936	chr1	115610860	115610879	-	20	86	16	70	0	0	0	0	Precursor_24	chr1_1936|chr1:115610861-115610911:-	53	86	GAAGTGGGCACAGATAGGGG	TCTCCCATCACAAAACTGCTCCTCTTATCTGGGGAAGTGGGCACAGATAGGGG
25	Mature_25	bta-miR-1_2230	chr1	115941154	115941172	-	19	20	0	0	20	0	0	0	Precursor_25	chr1_2230|chr1:115941154-115941212:-	59	20	CAGGGAGATGTTGATGAAT	TCATCAACCCTGTCTTCTATTTCCTTTTGGGAGATCACTTCAGGGAGATGTTGATGAAT
26	Mature_26	bta-miR-1_872	chr1	118097436	118097454	+	19	1006	0	1006	0	0	0	0	Precursor_26	chr1_872|chr1:118097436-118097505:+	70	1006	AGGAGGGGAAGGGGAAAAG	AGGAGGGGAAGGGGAAAAGTTTGAGAATTTCCCTTCCAGGAGCTTCTTTTTGTCCCTCACTCTCTCCTCC
27	Mature_27	bta-miR-1_782	chr1	118139267	118139284	+	18	9	9	0	0	0	0	0	Precursor_27	chr1_782|chr1:118139267-118139318:+	52	9	GAAGAAAAAGAGAAGAAC	GAAGAAAAAGAGAAGAACTAGAAGCTCCCTTGGGAGTTCTTTTCATCTTTTC
28	Mature_28	bta-miR-1_929	chr1	126134358	126134378	+	21	12	0	12	0	0	0	0	Precursor_28	chr1_929|chr1:126134291-126134378:+	88	12	TCCATTGGAGGGCGGTTAGGC	CTTTCATAAATGTCTCCCAGTGGTGGATAGAAAATCAAAGGTAGTACTCACCTCTGACTACTTTATATCCATTGGAGGGCGGTTAGGC
29	Mature_29	bta-miR-1_1068	chr1	127235095	127235113	+	19	48	0	0	0	0	48	0	Precursor_29	chr1_1068|chr1:127235095-127235161:+	67	48	AGGGCTCTTTTATTCTCTT	AGGGCTCTTTTATTCTCTTTGTTTCAGGCACGGTTTCCTGTGTTGGAGAATTGCTTCATGTCTCTGG
30	Mature_30	bta-miR-1_2405	chr1	137580345	137580363	-	19	16	0	0	16	0	0	0	Precursor_30	chr1_2405|chr1:137580285-137580363:-	79	32	AGGAAAGTAGGAAGAGAAG	AGGAAAGTAGGAAGAGAAGTGGGGAGGAGGAGAGAGAGATCTTACTTTACTTTTTCAGTCCATTCACTTCCTACTCTTT
31	Mature_31	bta-miR-1_1215	chr1	139992784	139992804	+	21	13	0	0	0	0	0	13	Precursor_31	chr1_1215|chr1:139992753-139992804:+	52	13	CTGGACTGCTATGGAATTCCC	GCCTAGCAGTGAAAGTGCTGAGTCCTAGCAACTGGACTGCTATGGAATTCCC
32	Mature_32	bta-miR-1_2380	chr1	142739422	142739439	-	18	15	0	0	0	15	0	0	Precursor_32	chr1_2380|chr1:142739422-142739465:-	44	15	AGGTACAGAAAGAGTCAG	GATCTTTGTGTTTCCTATTTGAAAATAGGTACAGAAAGAGTCAG
33	Mature_33	bta-miR-1_1228	chr1	151883775	151883792	+	18	11	0	0	11	0	0	0	Precursor_33	chr1_1228|chr1:151883775-151883831:+	57	11	AGAATCTGAATTTATTGC	AGAATCTGAATTTATTGCTTACTTAAGTAAAGTAGGGTTGTGGAGATCAGATTTAGT
34	Mature_34	bta-miR-1_1240	chr1	152655462	152655480	+	19	26	0	0	26	0	0	0	Precursor_34	chr1_1240|chr1:152655462-152655506:+	45	26	GGGAGAAGGAAAGGAGGGG	GGGAGAAGGAAAGGAGGGGGAAAAACACCTGTTTACTTCTTAAGA
35	Mature_35	bta-miR-1_1147	chr1	153179160	153179177	+	18	36	0	11	0	0	25	0	Precursor_35	chr1_1147|chr1:153179097-153179177:+	81	36	CAGGTTAAGTGAGCTGGC	CAGTGGCAGCCGGTCTTACACACGCTGCTGGCAAGTGTCAGCTGCGAGAAAAAAAAAAAGGACCAGGTTAAGTGAGCTGGC
36	Mature_36	bta-miR-1_2291	chr1	154611424	154611443	-	20	11	0	11	0	0	0	0	Precursor_36	chr1_2291|chr1:154611359-154611443:-	85	11	AGGGGAAGTTTGTGTGGAGG	AGGGGAAGTTTGTGTGGAGGGGAAACTTAAGTAGAGTGAAGAAATTCTCTCAAAATTGTACTTACTTCACACATTCTTTCCACTT
37	Mature_37	bta-miR-1_2300	chr1	156362192	156362210	-	19	293	0	293	0	0	0	0	Precursor_37	chr1_2300|chr1:156362192-156362245:-	54	293	GAAATCCGAACGAACTTTT	AAGTTCGTTCAGGTTTATCCACACCAGCTTACAGAGAAATCCGAACGAACTTTT
38	Mature_38	bta-miR-2_4086	chr2	919400	919422	-	23	34	0	0	34	0	0	0	Precursor_38	chr2_4086|chr2:919400-919448:-	49	34	GGGAAACAGAGCAAAACCAGATG	CCTGGCCTCTGTTGTTCTAGGCCCTTGGGAAACAGAGCAAAACCAGATG
39	Mature_39	bta-miR-2_2224	chr2	2007285	2007303	+	19	7307	1399	0	5908	0	0	0	Precursor_39	chr2_2224|chr2:2007285-2007350:+	66	7307	AGAGAAGAGAAGAGAAGAG	AGAGAAGAGAAGAGAAGAGAAGCCAGAAAAGAGGAAGCCTGAGTCCCCTGATTTTCTCATTTCTCC
40	Mature_40	bta-miR-2_2267	chr2	6777716	6777737	+	22	8	8	0	0	0	0	0	Precursor_40	chr2_2267|chr2:6777687-6777737:+	51	8	CAGCAGGAGCAGCAGGAACAGC	GTTTCTTGCACTCCTGTTGACAAACACCTCAGCAGGAGCAGCAGGAACAGC
41	Mature_41	bta-miR-2_2697	chr2	6787372	6787392	+	21	8	0	0	8	0	0	0	Precursor_41	chr2_2697|chr2:6787332-6787392:+	61	10	GGTTGTTGCAGGAGGAGACAG	GTACTGCTACTGCAATCACAATCCAAAGGGATTGGATTGTGGTTGTTGCAGGAGGAGACAG
42	Mature_42	bta-miR-2_3496	chr2	7073343	7073360	-	18	52	52	0	0	0	0	0	Precursor_42	chr2_3496|chr2:7073343-7073407:-	65	52	GAAGAGAAAAAAGAAGAC	CTTCCTTAATTTCTCTTTCTATTTTCTCATCCTTGAGAGCTTGAATAGAAGAGAAAAAAGAAGAC
43	Mature_43	bta-miR-2_2760	chr2	14934343	14934361	+	19	43	0	0	43	0	0	0	Precursor_43	chr2_2760|chr2:14934343-14934419:+	77	43	GGAAGAAGAGAAGAGGAAC	GGAAGAAGAGAAGAGGAACTGGTTTCTGCATTGTATGATTTGAGCCACTGGATCAGTTCTCTTCATAATCTATCCCC
44	Mature_44	bta-miR-2_2948	chr2	19070328	19070346	+	19	18	0	0	18	0	0	0	Precursor_44	chr2_2948|chr2:19070328-19070401:+	74	21	AGGGGAAAAAGAAGGAGGG	AGGGGAAAAAGAAGGAGGGAGGAAAGAATGGAAGGAAGGGATTTTTGCTCTCCCTCTGAGATAGTCCTGCTTT
45	Mature_45	bta-miR-2_3726	chr2	34030564	34030582	-	19	21	21	0	0	0	0	0	Precursor_45	chr2_3726|chr2:34030564-34030609:-	46	21	GGAGGGTGAGTGAGGGCCT	GTGATAAGGACACACTTTCCTGGTGGAGGAGGGTGAGTGAGGGCCT
46	Mature_46	bta-miR-2_3126	chr2	36940082	36940106	+	25	84	0	0	84	0	0	0	Precursor_46	chr2_3126|chr2:36940082-36940175:+	94	84	GATGAGGAAGATGAGGAAGATGAGG	GATGAGGAAGATGAGGAAGATGAGGAAGACGATGACTCTGATGACAGCCAATCAGGTTGTTTTCTATTCTTAAGTTTAAAGTGTTTCTCTAAAT
47	Mature_47	bta-miR-2_4446	chr2	37302282	37302299	-	18	15	0	0	15	0	0	0	Precursor_47	chr2_4446|chr2:37302282-37302348:-	67	15	GAAGAAGAGCCGAGAGGG	CTCTTCTCTTTAGAAGCAAAGGAGTGTTGAAAATGTTAAAGGCTGTGCTGAAGAAGAGCCGAGAGGG
48	Mature_48	bta-miR-2_3620	chr2	38955116	38955140	-	25	17	0	17	0	0	0	0	Precursor_48	chr2_3620|chr2:38955116-38955165:-	50	17	GATCCCACATGCCAAATGGTGTGGT	CACACTTCCCTGGTTGGAGAACAAAGATCCCACATGCCAAATGGTGTGGT
49	Mature_49	bta-miR-2_2685	chr2	43798671	43798688	+	18	19	19	0	0	0	0	0	Precursor_49	chr2_2685|chr2:43798611-43798688:+	78	19	GGGAGAAAAAAGAGAAAG	TTTACTGAGAACTCCCTGAGGGTAGCAGGCATTTAGGGGAAATGGCATGCTCTATCCTAAGGGAGAAAAAAGAGAAAG
50	Mature_50	bta-miR-2_4510	chr2	48502090	48502107	-	18	52	0	0	52	0	0	0	Precursor_50	chr2_4510|chr2:48502090-48502135:-	46	52	GGAAGAGAAAAGGAAGAG	CTTCTTGGAGTTTGGTTAGATTTAAATAGGAAGAGAAAAGGAAGAG
51	Mature_51	bta-miR-2_4557	chr2	53625120	53625137	-	18	12	0	0	12	0	0	0	Precursor_51	chr2_4557|chr2:53625086-53625137:-	52	12	GAAGGAGGAAAGAAGAAT	GAAGGAGGAAAGAAGAATCAAAACCACTAGAGATTTCTTTCCTGTCCTTGCC
52	Mature_52	bta-miR-2_4129	chr2	59382439	59382461	-	23	26	0	0	0	0	26	0	Precursor_52	chr2_4129|chr2:59382400-59382461:-	62	26	TCCACTTTAAGCCTTAAACACTC	TCCACTTTAAGCCTTAAACACTCAAAAAGCAAAAACAAAGGAGAGAAAGGGGTGGCTGGGGG
53	Mature_53	bta-miR-2_3764	chr2	59423586	59423607	-	22	19	0	19	0	0	0	0	Precursor_53	chr2_3764|chr2:59423586-59423666:-	81	19	TGTCACCAATATTGCTTTAAGG	ATGGAGCAGGTGGCAGATTAGAAGGTGAATCTATATGACTCCAGAACTTTCTTCTGAACTGTCACCAATATTGCTTTAAGG
54	Mature_54	bta-miR-2_2741	chr2	62089073	62089095	+	23	22	0	22	0	0	0	0	Precursor_54	chr2_2741|chr2:62089007-62089095:+	89	48	CTGGTGCCACTTGGGAGGCCTGT	TTGCCGACCCAGGGGTTGAACTCACATCTCCCACATCTCCTGCATTGGCAGGCAGATTCTTTACCACTGGTGCCACTTGGGAGGCCTGT
55	Mature_55	bta-miR-2_4004	chr2	69833441	69833459	-	19	12	12	0	0	0	0	0	Precursor_55	chr2_4004|chr2:69833441-69833495:-	55	12	AGAAATGGGAAGGAAAGTG	CTTCTCTTTAAGCTCATCAGCAGGGCCACAGTCCTGAGAAATGGGAAGGAAAGTG
56	Mature_56	bta-miR-2_4274	chr2	76932111	76932134	-	24	37	0	0	0	0	37	0	Precursor_56	chr2_4274|chr2:76932111-76932165:-	55	37	CATGCTCCGGGGTTTGGCCAAGGG	TATGGTTGGATCCCTGGTCAGGAAAGTTCCACATGCTCCGGGGTTTGGCCAAGGG
57	Mature_57	bta-miR-2_4726	chr2	78577981	78578005	-	25	19	0	0	19	0	0	0	Precursor_57	chr2_4726|chr2:78577981-78578065:-	85	19	TACTGAATACAGTTTTCTGTGCTGT	AGCAAAGTGATGTGTATATTCAGATGTGTATATTCTTTTCCACTATAGGTTATTACAAGATACTGAATACAGTTTTCTGTGCTGT
58	Mature_58	bta-miR-2_2962	chr2	83166484	83166507	+	24	43	43	0	0	0	0	0	Precursor_58	chr2_2962|chr2:83166484-83166555:+	72	43	GAAGTGAAGAGAAGAGAAGAGAAA	GAAGTGAAGAGAAGAGAAGAGAAAGCTCAGCTTTGGCTCCTCTTTTTATATGTTTTTTTTTTTTTTTTTCCT
59	Mature_59	bta-miR-2_3538	chr2	86098450	86098470	+	21	123	0	0	123	0	0	0	Precursor_59	chr2_3538|chr2:86098450-86098532:+	83	123	CGCTTCGGCAGCACATAAACT	CGCTTCGGCAGCACATAAACTAGAAAGCCTGGACTTCAAATTTCCCAGATGTCCGTGTAGGTGGTTCTTGGCTGCCCTGCCCA
60	Mature_60	bta-miR-2_4939	chr2	100740249	100740267	-	19	20	0	0	20	0	0	0	Precursor_60	chr2_4939|chr2:100740206-100740267:-	62	20	ACAGAAGAAGGGAAGGATC	ACAGAAGAAGGGAAGGATCAAGGGTAAGGTCTTACCTTTATTTCTAAACCTTCTTGATGTCT
61	Mature_61	bta-miR-2_4958	chr2	104019408	104019432	-	25	19	0	0	19	0	0	0	Precursor_61	chr2_4958|chr2:104019408-104019468:-	61	19	GGAGCCACGCTGGCCAGGGATGATG	TTATCCCGTAGTGGTAGCAATCCATATTCTTCGTTTGGAGCCACGCTGGCCAGGGATGATG
62	Mature_62	bta-miR-2_4960	chr2	104237950	104237969	-	20	24	0	0	24	0	0	0	Precursor_62	chr2_4960|chr2:104237950-104238027:-	78	24	CTTCCTTTTATCACATGACC	GAATGGAAGGAGAAGGGAGTATGGATTGAGACAGAGGTTGGTTCTGGGGGAGATTCTGCTTCCTTTTATCACATGACC
63	Mature_63	bta-miR-2_4267	chr2	107218009	107218027	-	19	11	11	0	0	0	0	0	Precursor_63	chr2_4267|chr2:107217963-107218027:-	65	11	GCAAAGGGCAGAGAGAAGG	GCAAAGGGCAGAGAGAAGGGTATCATGTCTGGACATGATTCCTGCCTTCCTTCCTTGAGTGGTTC
64	Mature_64	bta-miR-2_4307	chr2	112694185	112694202	-	18	39	13	0	26	0	0	0	Precursor_64	chr2_4307|chr2:112694142-112694202:-	61	39	GCCCAGGGGAGGAGGAAG	GCCCAGGGGAGGAGGAAGCTAGTGGATATGTGCCCCAGCTTCCTTGCCCTTAGGTGGGACA
65	Mature_65	bta-miR-2_4131	chr2	118270785	118270807	-	23	12	0	12	0	0	0	0	Precursor_65	chr2_4131|chr2:118270728-118270807:-	80	12	ATACAACCAGGGGAAATCATGGC	ATACAACCAGGGGAAATCATGGCAAAAAAAAAAAACCTCATGGTGATGACACAGGAGGCCCAGTTCTCCTGGGTGGGGCT
66	Mature_66	bta-miR-2_3183	chr2	121571253	121571272	+	20	10	0	10	0	0	0	0	Precursor_66	chr2_3183|chr2:121571207-121571272:+	66	10	CCAAGCCAGCTTTGAATCCT	GTGTTCTGAGCTGGCGGCGGGGGGCGAGGGGCGCGACTTGCTGTCCCCAAGCCAGCTTTGAATCCT
67	Mature_67	bta-miR-2_3188	chr2	121960529	121960546	+	18	787	0	787	0	0	0	0	Precursor_67	chr2_3188|chr2:121960529-121960602:+	74	787	GAGGAGGAGAAGGAGGAG	GAGGAGGAGAAGGAGGAGGAGGAAGCCGCGGGGTTGGAGCTCTGTGGTGGCTCTGGCCCCTTCACCTTCCTGCA
68	Mature_68	bta-miR-2_3314	chr2	122833915	122833934	+	20	47	21	0	26	0	0	0	Precursor_68	chr2_3314|chr2:122833915-122833967:+	53	47	GGAAGAGGTTTCACTGAGCT	GGAAGAGGTTTCACTGAGCTGAAGGAAGAAAGGACAGCAGTGAGCACAGTGGG
69	Mature_69	bta-miR-2_3897	chr2	123839911	123839928	+	18	13	0	0	13	0	0	0	Precursor_69	chr2_3897|chr2:123839863-123839928:+	66	13	GAGAAGAAAAAGGAAGGG	CATCCATCCTCCCAGATGGCTTTTTAAGTGAAGATTTTTGGTAACTGGGAGAAGAAAAAGGAAGGG
70	Mature_70	bta-miR-2_3931	chr2	125393917	125393936	+	20	20	0	0	20	0	0	0	Precursor_70	chr2_3931|chr2:125393871-125393936:+	66	20	ACAGATCAGGCATCAAGGGC	CCTTGATGGCAGACCTCTGTCTTAACTCTGGTTTCCAGTACCTGGCACAGATCAGGCATCAAGGGC
71	Mature_71	bta-miR-2_3217	chr2	126332108	126332132	+	25	10	0	10	0	0	0	0	Precursor_71	chr2_3217|chr2:126332076-126332132:+	57	10	AGAGCAGAAGGCAGAGAGGAAGGGG	GATTGAGCTCTGCTTTCTTAGTTTGGCCATCAGAGCAGAAGGCAGAGAGGAAGGGG
72	Mature_72	bta-miR-2_3988	chr2	128328189	128328206	+	18	36	0	0	36	0	0	0	Precursor_72	chr2_3988|chr2:128328189-128328264:+	76	36	GGAGGGAAAAAAGGAGGG	GGAGGGAAAAAAGGAGGGGGTGGGAAATCCATCATGGAGTGACTTGTTTTCCTTTCCTTCCTGGGAAGTCCAGTGG
73	Mature_73	bta-miR-2_4278	chr2	129449644	129449662	-	19	293	0	293	0	0	0	0	Precursor_73	chr2_4278|chr2:129449644-129449696:-	53	293	GAAATCCGAACGAACTTTT	AAGTTCATTAGGTTTTTCTATAGGACATAACTGAGAAATCCGAACGAACTTTT
74	Mature_74	bta-miR-2_3269	chr2	131064491	131064510	+	20	13	0	13	0	0	0	0	Precursor_74	chr2_3269|chr2:131064445-131064510:+	66	13	CCCCAGGTTTCCTCATTTGG	ATGTGAGGTGCCCTGCGGACGCCTCACACATGTGCAGTGTGAGGGGCCCCAGGTTTCCTCATTTGG
75	Mature_75	bta-miR-2_3302	chr2	133582478	133582501	+	24	11	0	11	0	0	0	0	Precursor_75	chr2_3302|chr2:133582478-133582567:+	90	11	CAGGCTCGGGCAGTCTCTCCAGGT	CAGGCTCGGGCAGTCTCTCCAGGTGGAGCCACCAGGTTGGTACTTGCTTCGGGAAGCATCTGCTTGGGAGGTTGTTGCCTGGGCTCTGAA
76	Mature_76	bta-miR-2_3667	chr2	133725661	133725685	+	25	19	0	0	0	0	19	0	Precursor_76	chr2_3667|chr2:133725617-133725685:+	69	19	GATCCCTCATGCTGAGCAGTGCAGT	CGTGCTCCTCCTGCAGGGGGCTCAGTCTCCGTCCGGGGAGCTGAGATCCCTCATGCTGAGCAGTGCAGT
77	Mature_77	bta-miR-2_3307	chr2	133957627	133957646	+	20	387	0	77	0	98	107	105	Precursor_77	chr2_3307|chr2:133957579-133957646:+	68	387	TGAGGGGAAGGGGGGAAAAG	CATCTTCTCTTCCCAAGACTGGAAACCAACTCAGGCATTTTCCAGTACTGAGGGGAAGGGGGGAAAAG
78	Mature_78	bta-miR-3_4369	chr3	4460296	4460316	+	21	45	0	45	0	0	0	0	Precursor_78	chr3_4369|chr3:4460258-4460316:+	59	45	CACATGCTACATGATGCGGCC	CTGCAGGGTGTGTGGGTTCAATCGGGGAACTGAGATCTCACATGCTACATGATGCGGCC
79	Mature_79	bta-miR-3_6823	chr3	6213509	6213526	-	18	1007	0	0	1007	0	0	0	Precursor_79	chr3_6823|chr3:6213446-6213526:-	81	1007	CGGAGGAGGGGGAGGAGG	CGGAGGAGGGGGAGGAGGGAAAGGAAGTGATAAACAGACATTACACATTCTTTTCCTGGAAAGATCACCCAGTTTTCCCAA
80	Mature_80	bta-miR-3_5410	chr3	7422394	7422411	+	18	16	0	0	16	0	0	0	Precursor_80	chr3_5410|chr3:7422394-7422434:+	41	16	TGAGGAGGAGAGGAAAGC	TGAGGAGGAGAGGAAAGCGGTCAGCTTCCTCTCACCCCATC
81	Mature_81	bta-miR-3_6854	chr3	8804728	8804746	-	19	13	0	0	13	0	0	0	Precursor_81	chr3_6854|chr3:8804728-8804779:-	52	13	GGAAAGAGACAGAAAAGGA	CTTTTCTGAATCCTGTTTCTGGATTCAAGTGGGGGAAAGAGACAGAAAAGGA
82	Mature_82	bta-miR-3_5485	chr3	13749544	13749561	+	18	18	0	0	18	0	0	0	Precursor_82	chr3_5485|chr3:13749476-13749561:+	86	18	GGGCTGTGAGGACTGACC	AAAGTCAGCACCTGTAGCAGGCCCAGGGGACAGGGCATTTATCTCTGGACAAACAGATTCCTGGGGCTGGGCTGTGAGGACTGACC
83	Mature_83	bta-miR-3_4685	chr3	14799773	14799797	+	25	8	8	0	0	0	0	0	Precursor_83	chr3_4685|chr3:14799729-14799797:+	69	8	GGGAGGAGAGGGACTGGGATGTGGC	CTATCCCCCCTCACTTCATCTCCTGCTTGAAGTAAGGGGGAGGCGGGAGGAGAGGGACTGGGATGTGGC
84	Mature_84	bta-miR-3_5544	chr3	16463735	16463756	+	22	20	0	0	20	0	0	0	Precursor_84	chr3_5544|chr3:16463735-16463809:+	75	20	CCCATCTAAATAATTAATGAGC	CCCATCTAAATAATTAATGAGCAGAATAAAAAGAGTAAAATCATTTCCTTCCCTATCTGTAATTTGGTTTGGGGC
85	Mature_85	bta-miR-3_5430	chr3	16484049	16484066	-	18	160	0	160	0	0	0	0	Precursor_85	chr3_5430|chr3:16484049-16484121:-	73	160	CGGTGGCGGGGGGAGGCC	CCTTCTGGCGTCGGCTGGGCCCGCGGGCAAACTCAGGGGACTGAGGGGCACACGGCGGTGGCGGGGGGAGGCC
86	Mature_86	bta-miR-3_4529	chr3	19988642	19988662	+	21	10	0	10	0	0	0	0	Precursor_86	chr3_4529|chr3:19988610-19988662:+	53	10	GGAGCCCTGGAAGCACAAGTG	CTTGTGGTTCTTGCTGGGCTTTCAGTGCTGTGGGAGCCCTGGAAGCACAAGTG
87	Mature_87	bta-miR-3_5465	chr3	20140585	20140605	-	21	15	0	15	0	0	0	0	Precursor_87	chr3_5465|chr3:20140517-20140605:-	89	15	GGAGTTAGGAGGAAAAGAAAC	GGAGTTAGGAGGAAAAGAAACAGGGTGAGAGGCTGAGGTTACTTTCTTCCTCCCTCCCCTTGCCTTGATTCTTGGTTGCCTGACTCTTT
88	Mature_88	bta-miR-3_4609	chr3	28374059	28374080	+	22	973056	0	973056	0	0	0	0	Precursor_88	chr3_4609|chr3:28373995-28374080:+	86	973070	CCCCACTGCTAAATTTGACTGG	GGTCTGATGGTAATGGGTTACCAGAACTTATTAACATTAGTGTCACTAAAATTAGTATACAACTCCCCACTGCTAAATTTGACTGG
89	Mature_89	bta-miR-3_5546	chr3	29178337	29178356	-	20	33	0	33	0	0	0	0	Precursor_89	chr3_5546|chr3:29178337-29178394:-	58	64	GGGTTCAATCCTGGCTGGGG	CCAGTGGTTAGGAATCCATCTTCCAGTGCAGGGGATGTGGGTTCAATCCTGGCTGGGG
90	Mature_90	bta-miR-3_6055	chr3	34456087	34456104	-	18	73	8	65	0	0	0	0	Precursor_90	chr3_6055|chr3:34456039-34456104:-	66	73	GTCCCTTGGGAAGGTCTG	GTCCCTTGGGAAGGTCTGGGACTAGGGCCATAGCGCGGCCCTAAAAGGGCCTTCCCTGAGCTTTGG
91	Mature_91	bta-miR-3_6165	chr3	34636993	34637013	-	21	13	0	0	0	0	13	0	Precursor_91	chr3_6165|chr3:34636993-34637082:-	90	21	CCCCAGGTTGGGAAGATCCCC	CTTTCCCAGTGGCTCAGTGATAAAGAATCTGCCTGCAATGCAAGAGAGTTGCAGGTGACACAAATCAATCCCCAGGTTGGGAAGATCCCC

92	Mature_92	bta-miR-3_4979	chr3	37346307	37346324	+	18	42	42	0	0	0	0	0	Precursor_92	chr3_4979|chr3:37346246-37346324:+	79	42	GGAGAAAGAAGAGGAGAT	CTCCCACAATTTCTCAAAGTGATGCAAGCCACTCTTCAGAGAAGGAGGAGGGTGAGCAGGAGGAGAAAGAAGAGGAGAT
93	Mature_93	bta-miR-3_4993	chr3	40729461	40729480	+	20	20	10	0	10	0	0	0	Precursor_93	chr3_4993|chr3:40729435-40729479:+	47	20	AGGAAGAGAACAAGAGAACT	GGCTCTTGTGAGGTTATTCCTCAGAAAAGGAAGAGAACAAGAGAACT
94	Mature_94	bta-miR-3_5254	chr3	44062506	44062525	+	20	27	0	0	0	0	0	27	Precursor_94	chr3_5254|chr3:44062506-44062544:+	39	27	TTCCCTTCTTTTTCATATAC	TTCCCTTCTTTTTCATATACAAACAGAGAAAAGGGAATA
95	Mature_95	bta-miR-3_5239	chr3	46973157	46973180	+	24	171	0	0	0	0	171	0	Precursor_95	chr3_5239|chr3:46973131-46973180:+	50	171	CAGCCCTGACTTGAGCTTTAGAGC	TACTTCAGCCCAATTAGCTGAGCTCACAGCCCTGACTTGAGCTTTAGAGC
96	Mature_96	bta-miR-3_7293	chr3	49194145	49194162	-	18	10	0	0	10	0	0	0	Precursor_96	chr3_7293|chr3:49194115-49194162:-	48	10	GGTAAAGGAAAAGGGAAG	GGTAAAGGAAAAGGGAAGGGGACGGCTGTCTTCCTGTGTTTGGATGAC
97	Mature_97	bta-miR-3_6012	chr3	51650440	51650458	+	19	30	0	0	30	0	0	0	Precursor_97	chr3_6012|chr3:51650440-51650516:+	77	30	AGGGGAAGGAGGAGAAGAG	AGGGGAAGGAGGAGAAGAGCCTTGAGCTGATAAAATCAAGACCTGAGACTTGAGGTACTATTATCCCTATCTCCACC
98	Mature_98	bta-miR-3_4861	chr3	57355930	57355952	+	23	36	0	36	0	0	0	0	Precursor_98	chr3_4861|chr3:57355880-57355952:+	73	36	CCTGCAAGATATATGGCATGGCC	CACTCTCGCTGGTGTTGCCTGGGTTCAGTTCCTATTTGGGGAAGTGAGATCCTGCAAGATATATGGCATGGCC
99	Mature_99	bta-miR-3_4865	chr3	58263566	58263585	+	20	12	0	12	0	0	0	0	Precursor_99	chr3_4865|chr3:58263515-58263585:+	71	17	GTCTCTTCCCTTTCTACTAC	GGAAAGGGGCAGGAAGAGATAGGAAGGGGTGGGAAGCAAGGGCTTCCATGTGTCTCTTCCCTTTCTACTAC
100	Mature_100	bta-miR-3_5257	chr3	66144898	66144916	+	19	3266	0	0	0	1030	1028	1208	Precursor_101	chr3_5257|chr3:66144872-66144916:+	45	3266	CCATGGGAGTGGGTTGCAT	GTCTCTTCCCTTTCTACTACAGTAGCCTTTACTTTATGAGTTAGGAATTTTCACTGTTCTCTGAGATTGGAGGGGAGGAAAAAA
101	Mature_101	bta-miR-3_4933	chr3	66884972	66884993	+	22	75	0	75	0	0	0	0	Precursor_102	chr3_4933|chr3:66884908-66884993:+	86	75	CCATATACTGTGTGGTGCAGCC	GCCAGGCTCCTCTTCCATGGAATTTTCCATGGGAGTGGGTTGCAT
102	Mature_102	bta-miR-3_6193	chr3	70145573	70145592	+	20	20	0	0	20	0	0	0	Precursor_103	chr3_6193|chr3:70145545-70145592:+	48	20	GGAGAGAGGAAAGGGGAGAC	TTGCCATTTCCTACTCCAGAGGATCTTCCAGACTCAGGGATCTAATCCTGAGGGAACTAAGATCCCATATACTGTGTGGTGCAGCC
103	Mature_103	bta-miR-3_5057	chr3	87184279	87184302	+	24	21	0	21	0	0	0	0	Precursor_104	chr3_5057|chr3:87184218-87184302:+	85	21	CAGGAGGTTGAGGAAAGCATTGGG	CTCCTATGATTAACTTCTGTCCTTCCTGGGAGAGAGGAAAGGGGAGAC
104	Mature_104	bta-miR-3_7657	chr3	88129882	88129900	-	19	28	0	0	28	0	0	0	Precursor_105	chr3_7657|chr3:88129854-88129900:-	47	28	GAGAGGAAAGAAGAGGAAG	CAGGAAGCAGCCTCAAATGTTGAGAAGGGAGAGGAGAGTTGTGATTTCTTTCCCTTTCCCTCAGGAGGTTGAGGAAAGCATTGGG
105	Mature_105	bta-miR-3_6352	chr3	88859533	88859551	+	19	25	0	0	25	0	0	0	Precursor_106	chr3_6352|chr3:88859533-88859605:+	73	25	GTATCAGAAAACACAGCCC	GAGAGGAAAGAAGAGGAAGCCTGACATTTATTCCCTGGCTCTCTCCA
106	Mature_106	bta-miR-3_5985	chr3	89058421	89058441	-	21	11	0	11	0	0	0	0	Precursor_107	chr3_5985|chr3:89058385-89058441:-	57	11	GAGGGAGAGAGTGGAAGAAAG	GTATCAGAAAACACAGCCCTTCCTCAGATCCAGTGAATGGGCATCCACAGGTAGGGACTGAGCATCTGTATGC
107	Mature_107	bta-miR-3_6606	chr3	99751390	99751408	-	19	163	0	0	0	0	0	163	Precursor_108	chr3_6606|chr3:99751390-99751432:-	43	163	ATCTGGGAGTCTGGGGTGC	GAGGGAGAGAGTGGAAGAAAGGCAGGTTTTGGTTGACCTTCTTCCACCCTTCCTTTG
108	Mature_108	bta-miR-3_5675	chr3	100644229	100644247	+	19	141	0	0	0	0	0	141	Precursor_109	chr3_5675|chr3:100644171-100644247:+	77	141	CATGCCATGAGGTATGGCC	AACCCGGAGCTTTGGGAAAGAGTGATCTGGGAGTCTGGGGTGC
109	Mature_109	bta-miR-3_6490	chr3	101525820	101525837	+	18	20	0	0	20	0	0	0	Precursor_110	chr3_6490|chr3:101525820-101525897:+	78	20	AGGGAGAAAAAAGAAGGG	CTGTGCTTCCATTGCAGGGGGTATGGGTTCAATCCTTGGTTAGGGAACTAATATCCCCCATGCCATGAGGTATGGCC
110	Mature_110	bta-miR-3_6599	chr3	108287116	108287133	+	18	81	0	0	81	0	0	0	Precursor_111	chr3_6599|chr3:108287086-108287133:+	48	81	GGGCAGAAGAGGAGGAAG	AGGGAGAAAAAAGAAGGGGGAGTGTGTGTGGGGGGGCACCTCCACACAAAAGAGCATTCTGAGCCCTTGGTTCCCTGG
111								0							Precursor_100	chr3_4866|chr3:58263566-58263649:+	84	12		TTGTTGACTCTTCCTGCTGTTGGGGTTCCAGGGCAGAAGAGGAGGAAG
112	Mature_111	bta-miR-3_5583	chr3	109484609	109484628	+	20	10	10	0	0	0	0	0	Precursor_112	chr3_5583|chr3:109484582-109484628:+	47	10	AACAAAAGGAGGATGCAGAG	CTTGCAATTGTGTTTGTTTTCTCATGAAACAAAAGGAGGATGCAGAG
113	Mature_112	bta-miR-3_5603	chr3	110688894	110688914	+	21	17	17	0	0	0	0	0	Precursor_113	chr3_5603|chr3:110688856-110688914:+	59	17	GAAAGGGAAAGGAGAAAGAAG	TGTCATACTCCTTTCCCAATTTTGAACCAGGCACAGAAGAAAGGGAAAGGAGAAAGAAG
114	Mature_113	bta-miR-3_6156	chr3	111601974	111601995	-	22	18	0	18	0	0	0	0	Precursor_114	chr3_6156|chr3:111601974-111602063:-	90	18	CCTGCGGGAGGAGTCCGCTTCC	TGGTGGGCTCTTCCCTGGAGTGGGGCGGGGAAGGGGGGAGCCCTGGCTCCTAAACTTGGGCCTTCTCTCCTGCGGGAGGAGTCCGCTTCC
115								0							Precursor_115	chr3_6161|chr3:112357013-112357082:-	70	11		CCAGGAGGTGGACAAAAGGGCAGCTGCGGGGGGGACTAACCCAGAACTGACCAGGCCACCTTTAAGGGCA
116	Mature_114	bta-miR-3_6162	chr3	112357063	112357082	-	20	11	0	11	0	0	0	0	Precursor_116	chr3_6162|chr3:112357063-112357108:-	46	11	CCAGGAGGTGGACAAAAGGG	CAAACCACCCTGACCTTCTGGCCCTGCCAGGAGGTGGACAAAAGGG
117								0							Precursor_117	chr3_6757|chr3:114561990-114562035:-	46	37		CCCGCGAGGGGGCGGGGCGATGGGGATCCAAGCCCCCGCAAGGGCC
118	Mature_115	bta-miR-3_6180	chr3	114562015	114562035	-	21	268	0	160	0	27	37	44	Precursor_118	chr3_6180|chr3:114562015-114562067:-	54	271	CCCGCGAGGGGGCGGGGCGAT	AGTCCCAGCCTCCCCACCAGGAGAGGTGGCCTCCCCGCGAGGGGGCGGGGCGAT
119	Mature_116	bta-miR-4_8162	chr4	21028927	21028947	+	21	16	0	0	16	0	0	0	Precursor_119	chr4_8162|chr4:21028927-21028988:+	62	16	GGGGACCCAGGAAGAGCAAGG	GGGGACCCAGGAAGAGCAAGGAAAAGATAACTCACAGCCTGTTCCTTTCCTGAGGTTCCGGT
120	Mature_117	bta-miR-4_7364	chr4	32088077	32088095	-	19	16	0	16	0	0	0	0	Precursor_120	chr4_7364|chr4:32088077-32088129:-	53	16	TCCCCCCTCCTTCCTCTCC	AGAGGGTTTGGGGAGGAAAAGACAAAAAGTAGTTTCCCCCCTCCTTCCTCTCC
121	Mature_118	bta-miR-4_8215	chr4	32397022	32397042	+	21	14	0	0	14	0	0	0	Precursor_121	chr4_8215|chr4:32396958-32397042:+	85	14	GGTCTGCCAACGAGCAACAAG	AGTGGCTTGAGCAGAGTGTACATGCCCCCTGGTAGATCAGATGGGAAAGGAGTCCAGGGGGCCTGGTCTGCCAACGAGCAACAAG
122	Mature_119	bta-miR-4_6538	chr4	45950256	45950280	+	25	19	0	19	0	0	0	0	Precursor_122	chr4_6538|chr4:45950256-45950332:+	77	19	GGGAGTCTAGCAGGCTTAGGAGAGC	GGGAGTCTAGCAGGCTTAGGAGAGCTTGTCCTCACTGACAAGGAACTCAGTTGCTCCTAAGTTTATCCCTTTTACTG
123	Mature_120	bta-miR-4_6586	chr4	52065675	52065694	+	20	19	0	19	0	0	0	0	Precursor_123	chr4_6586|chr4:52065675-52065740:+	66	19	TCTCTGCTCAGTATCTATGC	TCTCTGCTCAGTATCTATGCAAAAAAAAAAAAAAAAAATGGGGATGGATAAAATGGGTAGGGGAC
124	Mature_121	bta-miR-4_7977	chr4	57134865	57134889	-	25	46	0	0	0	46	0	0	Precursor_124	chr4_7977|chr4:57134826-57134889:-	64	46	CAACCCAGTCTTTCTATCCTAGCCC	CAACCCAGTCTTTCTATCCTAGCCCAAAAAAAAAAAAAAAAAATAGGAGGGTAGATTGGGATGTG
125	Mature_122	bta-miR-4_8276	chr4	63838262	63838279	-	18	32	0	0	0	0	0	32	Precursor_125	chr4_8276|chr4:63838262-63838336:-	75	69	CCTCCGAGCAGTGTGGCC	CCTCCACTGTAGGAGGCATAGGTTCCATCCCTCATCAGGGAACTAAGATTCCACAAGCCTCCGAGCAGTGTGGCC
126	Mature_123	bta-miR-4_9587	chr4	66568824	66568841	-	18	26	0	0	26	0	0	0	Precursor_126	chr4_9587|chr4:66568783-66568841:-	59	26	GGGGAAGAGAAAAAAAGG	GGGGAAGAGAAAAAAAGGCACCTGCATAAGCATCTTTGTCTACAAGTCACATTCCCCTG
127	Mature_124	bta-miR-4_7606	chr4	69774553	69774571	-	19	14	0	14	0	0	0	0	Precursor_127	chr4_7606|chr4:69774553-69774600:-	48	14	CATACCTCAGGGTGTAGCC	TCTCAATCCCTGGTTGGGAATTAAGATCCCATACCTCAGGGTGTAGCC
128	Mature_125	bta-miR-4_8644	chr4	80168837	80168855	+	19	12	0	0	12	0	0	0	Precursor_128	chr4_8644|chr4:80168837-80168890:+	54	12	GAGTTAGAAGAGGAGGAAT	GAGTTAGAAGAGGAGGAATCTGCCCCAGGTTCTAGTCATACATGATGACATCTG
129	Mature_126	bta-miR-4_8257	chr4	82404048	82404066	-	19	11	11	0	0	0	0	0	Precursor_129	chr4_8257|chr4:82403992-82404066:-	75	11	AGGAGAGAGCAGAGAAGGG	AGGAGAGAGCAGAGAAGGGTGTCCAGACCCAGACAAGCTGGGCTCTTGGATAGTTCTCACGTAAGTCTCTCTTCA
130	Mature_127	bta-miR-4_7725	chr4	89038544	89038562	-	19	2016	0	2016	0	0	0	0	Precursor_130	chr4_7725|chr4:89038485-89038562:-	78	2016	GGGCGCGCGCCGCGGCTGG	GGGCGCGCGCCGCGGCTGGAGCGGCGGGGAGAGGCCGGGCCACCTCCCCCCTTCCCAGCCATGCACCGCCTCGCCCGC
131	Mature_128	bta-miR-4_8783	chr4	95215190	95215207	+	18	24	0	0	24	0	0	0	Precursor_131	chr4_8783|chr4:95215128-95215207:+	80	24	AGGACAGGAGAGAAGGAC	CTGGTATTCTTGTTCTACTTCCTGACTCTGTCTTCCCTTATGGCTTTGGTAGGTAGCTAGGTAGGACAGGAGAGAAGGAC
132								0							Precursor_132	chr4_8784|chr4:95215190-95215242:+	53	24		AGGACAGGAGAGAAGGACAGAAGGGAAAGAATCTGCCATTTCATGTCTTCTAC
133	Mature_129	bta-miR-4_6937	chr4	98386894	98386918	+	25	13	0	13	0	0	0	0	Precursor_133	chr4_6937|chr4:98386831-98386918:+	88	13	TTCCCACTCTGTCCTCCTGTAGGTG	GCACAGGAGGGGTGGGGGGCACTGGGTTGTCTGAACATTTCAACCTCACCGCCTGTTTACACTTTCCCACTCTGTCCTCCTGTAGGTG
134	Mature_130	bta-miR-4_8413	chr4	99827109	99827126	-	18	12	12	0	0	0	0	0	Precursor_134	chr4_8413|chr4:99827050-99827126:-	77	12	GCTGGGCCCGGTGGCCGC	GCTGGGCCCGGTGGCCGCAAAAAAAAAAAAACAACACTTTTCTTTCTCCCGCCCTAACGGTCGCCGCGGCCAACTGC
135	Mature_131	bta-miR-4_7493	chr4	100714440	100714462	+	23	43	43	0	0	0	0	0	Precursor_135	chr4_7493|chr4:100714400-100714462:+	63	43	GAGGAAAGGAAAGAGAAAGGAGC	TGATACCTTCTCTATCACAGATCCCAGTAGAATAGGACAGGAGGAAAGGAAAGAGAAAGGAGC
136	Mature_132	bta-miR-4_7663	chr4	104749817	104749840	+	24	35	0	0	0	0	35	0	Precursor_136	chr4_7663|chr4:104749755-104749840:+	86	39	CCCACATGCTATGTGGAGCAGGCC	TTAAGACTCCACACTTCTGCTGTAGGGGCTGTGGGTTTGATCCCTTGTCAGGAAACTAAGGTCCCACATGCTATGTGGAGCAGGCC
137	Mature_133	bta-miR-4_7601	chr4	112160143	112160165	+	23	311796	127707	0	184089	0	0	0	Precursor_137	chr4_7601|chr4:112160079-112160165:+	87	312703	CCCCACTGCTAAATTTGACTGGC	GGTCCGATGGTAGTGGGTTACCAGAACTTATTAACGTTAGTGTCACTAAAGTTGGTATACAACCCCCCACTGCTAAATTTGACTGGC
138	Mature_134	bta-miR-4_7042	chr4	112652396	112652414	+	19	86	0	39	0	0	47	0	Precursor_138	chr4_7042|chr4:112652396-112652469:+	74	90	GGGGTGGGGGGGCGGCGAG	GGGGTGGGGGGGCGGCGAGGGGCGGGGTGGGGAGCGGTGCCCACGCTGCCTCTCTCCTCAGGTCCCCCTCCAAC
139	Mature_135	bta-miR-4_7931	chr4	113322612	113322629	-	18	322	0	322	0	0	0	0	Precursor_139	chr4_7931|chr4:113322566-113322629:-	64	322	GGTGGCGGGGGGAGGTCT	GGTGGCGGGGGGAGGTCTCTCCCTTCCTCTCTCTCTCTTTCTCTCCCTCCCTCTTTCTCTCTCT
140	Mature_136	bta-miR-4_8786	chr4	117584417	117584440	-	24	89	0	0	0	0	0	89	Precursor_140	chr4_8786|chr4:117584417-117584485:-	69	89	GTGTCCCCAAGTCAGGCTGAAGAC	CCTCAGCCTTGGAGGGGACGCCCTGGGTGGCCAAGGAAGACGCCAGTGTCCCCAAGTCAGGCTGAAGAC
141	Mature_137	bta-miR-5_9601	chr5	4505901	4505918	-	18	24	24	0	0	0	0	0	Precursor_141	chr5_9601|chr5:4505872-4505918:-	47	24	GGAAAAGGAAGAGAAAGA	GGAAAAGGAAGAGAAAGATGGCAGTATAGTTTTCTTCCTCAAATCCC
142	Mature_138	bta-miR-5_11325	chr5	12725027	12725044	-	18	213	0	0	213	0	0	0	Precursor_142	chr5_11325|chr5:12725002-12725044:-	43	224	GGAGTATGGTCAAGGCTG	GGAGTATGGTCAAGGCTGTATATTGTCACCCTGCTTATTTAAC
143	Mature_139	bta-miR-5_10104	chr5	24788557	24788580	-	24	54	0	0	0	0	54	0	Precursor_143	chr5_10104|chr5:24788557-24788603:-	47	54	CTGCCCACAAGGAGCCTTGAATCT	ATGGTATCAGATGTGGGCAGGCCCTGCCCACAAGGAGCCTTGAATCT
144	Mature_140	bta-miR-5_8178	chr5	26076705	26076724	+	20	16	0	16	0	0	0	0	Precursor_144	chr5_8178|chr5:26076705-26076766:+	62	16	ATATCCACTGGCCTTTCTTT	ATATCCACTGGCCTTTCTTTAAAAAAAAAAAAAAGTAGGGAATGGGGCCCTGTGGGAAGGTGG
145	Mature_141	bta-miR-5_10244	chr5	28321532	28321549	+	18	28	0	0	28	0	0	0	Precursor_145	chr5_10244|chr5:28321532-28321568:+	37	28	GAAGAGAAAAAAGAAGAA	GAAGAGAAAAAAGAAGAAAATGTTCTCTCTGCTTCCT
146	Mature_142	bta-miR-5_10284	chr5	30699881	30699900	+	20	20	0	0	20	0	0	0	Precursor_146	chr5_10284|chr5:30699881-30699955:+	75	20	GCGCGCGCGGGCTCTCCCCG	GCGCGCGCGGGCTCTCCCCGCCCTCACTTCCTCCTCAGCATGAAAACCGGAGGCGGGAAGGAACTGCGCACGCGC
147	Mature_143	bta-miR-5_8214	chr5	30702703	30702726	+	24	451	0	451	0	0	0	0	Precursor_147	chr5_8214|chr5:30702703-30702763:+	61	451	GGAAGGAGAGGGAGAGGAAGAAGG	GGAAGGAGAGGGAGAGGAAGAAGGAGAGGAATATTAATTACCATTCCTTTCAGCCTTGCAG
148	Mature_144	bta-miR-5_10302	chr5	30948828	30948850	+	23	20	0	0	20	0	0	0	Precursor_148	chr5_10302|chr5:30948828-30948888:+	61	20	GAGGGGACAGAGTGAGCTGGCTG	GAGGGGACAGAGTGAGCTGGCTGAGTCCATCTTGAAGTCAGTGAAATTTCTGTTTTCTATT
149	Mature_145	bta-miR-5_8941	chr5	47624947	47624966	+	20	11	11	0	0	0	0	0	Precursor_149	chr5_8941|chr5:47624927-47624966:+	40	11	GAGACGAGACTGAAGAGGCC	CTTTCGGTTCCTCCGGCTTTGAGACGAGACTGAAGAGGCC
150	Mature_146	bta-miR-5_9383	chr5	48885510	48885528	-	19	22	0	22	0	0	0	0	Precursor_150	chr5_9383|chr5:48885447-48885528:-	82	22	GAGAGAGAGGGGCCAAAAC	GAGAGAGAGGGGCCAAAACAGAAGCTGTGATCTTTTATAACCTCATCTTGGAAGAGACATCCTGTCGCTTCTCTCTATTCTG
151	Mature_147	bta-miR-5_9994	chr5	49510431	49510453	-	23	108	0	0	0	60	48	0	Precursor_151	chr5_9994|chr5:49510431-49510514:-	84	108	CCACGGTGACACTTTCTATCCTC	GGATGGGGTGGTGAAATCTAAAGTGGGAAGGGAAATGAACACGTTAAGTGGAAATGTGACCCCACGGTGACACTTTCTATCCTC
152	Mature_148	bta-miR-5_8429	chr5	54791148	54791166	+	19	29	0	29	0	0	0	0	Precursor_152	chr5_8429|chr5:54791097-54791166:+	70	29	GGGGAAAAGGGAAAAGAAG	TCTTAACCTTTTGCTCTGAAAATCTGAATCGACTAAGGAAATGAATGAGAGGGGGAAAAGGGAAAAGAAG
153	Mature_149	bta-miR-5_10131	chr5	57282853	57282873	-	21	47	47	0	0	0	0	0	Precursor_153	chr5_10131|chr5:57282826-57282873:-	48	47	CTCTCTCGCCTCGGCGCTTCC	CTCTCTCGCCTCGGCGCTTCCAAGATGGTGAGTGCTAGCGCGTGGGGA
154	Mature_150	bta-miR-5_10159	chr5	61184718	61184736	-	19	9	9	0	0	0	0	0	Precursor_154	chr5_10159|chr5:61184679-61184736:-	58	9	AAGTCAAGGAGCCAAGACA	AAGTCAAGGAGCCAAGACAAGGAGCCGGAGTTCTTGAACCTCAGCCCTGTCGGCTTCC
155	Mature_151	bta-miR-5_9095	chr5	64520148	64520168	+	21	519	0	0	0	519	0	0	Precursor_155	chr5_9095|chr5:64520148-64520246:+	99	527	AGGCAAGAACACTGGAGTGGG	AGGCAAGAACACTGGAGTGGGTTGCCATTGCAGTGGAAATAGATTTTGGTGAAAGGTACTACTTGCAGGTGGCCGAGAATCTCCAGGTTTAAAAACATT
156	Mature_152	bta-miR-5_9528	chr5	66573723	66573746	-	24	18	0	18	0	0	0	0	Precursor_156	chr5_9528|chr5:66573723-66573778:-	56	18	CCCTGTGCTGCCCTACCGGTCCAC	GGACTTTCAGAGGAGCAGCAGCAGGGAGCTTCCCCTGTGCTGCCCTACCGGTCCAC
157	Mature_153	bta-miR-5_11969	chr5	67602557	67602577	-	21	17	0	0	17	0	0	0	Precursor_157	chr5_11969|chr5:67602534-67602577:-	44	17	GGGGAAAGGGAGAAAGAAGAG	GGGGAAAGGGAGAAAGAAGAGAAAATGTCTCTCCCCCTTCCCAC
158	Mature_154	bta-miR-5_8532	chr5	67698468	67698491	+	24	12	0	12	0	0	0	0	Precursor_158	chr5_8532|chr5:67698468-67698528:+	61	12	TCCTGAAGGCAGCATGGCATGGCC	TCCTGAAGGCAGCATGGCATGGCCAAAAAAAAAAAAAGCCAACAAGGTCATCCTTCAGAAGC
159	Mature_155	bta-miR-5_9109	chr5	68729660	68729677	+	18	440	187	0	253	0	0	0	Precursor_159	chr5_9109|chr5:68729616-68729677:+	62	440	GAGAAGGAGAAGGAGAAC	ATTCTGGCTCTGTGTTCTGGATGTGAGCCACATGCACACATCCAGAGAAGGAGAAGGAGAAC
160	Mature_156	bta-miR-5_9577	chr5	73232058	73232076	-	19	11	0	11	0	0	0	0	Precursor_160	chr5_9577|chr5:73232019-73232076:-	58	11	AACTAAGTGAAAGCTGGTG	AACTAAGTGAAAGCTGGTGGCTCACAAAGAGACTGCTTGTGAGCTGGCATGAAGTTGC
161	Mature_157	bta-miR-5_10855	chr5	88554735	88554753	+	19	15	0	0	15	0	0	0	Precursor_161	chr5_10855|chr5:88554683-88554753:+	71	15	GAAGATAAACTCAAAGGAG	CCTTTTGTGTTTATAGTCTCTGACTGACGAGCTTGCTCTTGTGGATGTTTTGGAAGATAAACTCAAAGGAG
162	Mature_158	bta-miR-5_8775	chr5	97121547	97121567	+	21	15	0	15	0	0	0	0	Precursor_162	chr5_8775|chr5:97121547-97121597:+	51	15	AGATCCCACATACTACAACTG	AGATCCCACATACTACAACTGAGAGTTTGCATGTTGCAACTGAAGATCTGG
163	Mature_159	bta-miR-5_10425	chr5	98484599	98484617	-	19	13	13	0	0	0	0	0	Precursor_163	chr5_10425|chr5:98484571-98484617:-	47	13	GGGAAGAAGAAGAGAGGGG	GGGAAGAAGAAGAGAGGGGGAAATGTGGACTCTAAGCTACTTCCTTT
164	Mature_160	bta-miR-5_10812	chr5	100879557	100879575	-	19	97	0	0	0	0	97	0	Precursor_164	chr5_10812|chr5:100879557-100879629:-	73	128	CATACTGCATAGTGTGGCC	CCTTTCAATGCAAGTGGTGTGGGTTCGATCCCTGGTCAGGGAACTAGGATCCCACATACTGCATAGTGTGGCC
165	Mature_161	bta-miR-5_12317	chr5	105126811	105126829	-	19	14	0	0	14	0	0	0	Precursor_165	chr5_12317|chr5:105126750-105126829:-	80	14	GAAGAAGGAGGAAAGAAGC	GAAGAAGGAGGAAAGAAGCAGCAGGAGAAGATGCTTCTTTTGGGGTGTTACCGACTAGGGTTTTTGGCCTCCTTAATCAA
166	Mature_162	bta-miR-5_9488	chr5	107635316	107635335	+	20	130	0	0	0	53	42	35	Precursor_166	chr5_9488|chr5:107635316-107635377:+	62	130	TCCCTGGGCCTCAGTTCTCC	TCCCTGGGCCTCAGTTCTCCATGTGTAAAATGCATGGTTGGACTAGCTGATCTCTAGGATTT
167	Mature_163	bta-miR-5_11083	chr5	109617399	109617416	+	18	123	0	0	123	0	0	0	Precursor_167	chr5_11083|chr5:109617345-109617416:+	72	120	GGGAGAAAGGGAGAAAGG	TATCCAGTTCTCCCTTACAAGGGGAGGGGCAGAGAATCTCGGCCCCCGCCCAGAGGGAGAAAGGGAGAAAGG
168	Mature_164	bta-miR-5_10916	chr5	109789357	109789374	-	18	11	0	0	0	0	11	0	Precursor_168	chr5_10916|chr5:109789357-109789426:-	70	13	TATGCCTCGTGGTATGGC	TTCTACTGCAGTGGGCGGGAGTTCGATCCCTGGTCGGGGAACGAAGATCCTGTATGCCTCGTGGTATGGC
169	Mature_165	bta-miR-5_8934	chr5	112925400	112925419	+	20	18	0	18	0	0	0	0	Precursor_169	chr5_8934|chr5:112925400-112925453:+	54	18	CCCCCTTTGACAGTACACTT	CCCCCTTTGACAGTACACTTCACAGGTACTGTAAGTGTCTGTTCAAAAGTCTAG
170	Mature_166	bta-miR-5_8946	chr5	113413109	113413129	+	21	12	0	12	0	0	0	0	Precursor_170	chr5_8946|chr5:113413109-113413161:+	53	12	GAGAGGTGGTGTACCGGCAGC	GAGAGGTGGTGTACCGGCAGCAAAAACAAGAGATGAGGAGGTACGCGTCTCAC
171	Mature_167	bta-miR-6_13492	chr6	3491240	3491259	-	20	12	0	0	12	0	0	0	Precursor_171	chr6_13492|chr6:3491240-3491312:-	73	12	AGCAGCAGAAGGAGCAGCAG	GGTGGGCTGCCGTCTATGGGGTCGCACAGAGTCAGACACGACTGAAGCGACTTAGCAGCAGAAGGAGCAGCAG
172	Mature_168	bta-miR-6_10850	chr6	12668917	12668940	-	24	16	0	16	0	0	0	0	Precursor_172	chr6_10850|chr6:12668917-12668965:-	49	16	TCCCGCATGCTGCCTGGGATAGCC	TTCATACCGGGTCAGGGATCGAAGATCCCGCATGCTGCCTGGGATAGCC
173	Mature_169	bta-miR-6_10878	chr6	16865081	16865102	-	22	11	0	11	0	0	0	0	Precursor_173	chr6_10878|chr6:16865081-16865161:-	81	11	GAGATACCAATGGGAACAGACC	TCAGTTCCCTGGGTTTTCTTCTTGCCTCATATATCCTGGACTAGGTGCTGCAGCAGTTGGAGATACCAATGGGAACAGACC
174	Mature_170	bta-miR-6_10705	chr6	17083877	17083896	+	20	303	40	149	114	0	0	0	Precursor_174	chr6_10705|chr6:17083837-17083895:+	61	303	GGAGAAGGAAAACCGAAGCG	CTCCGCCCCCCACCTTCTCCTCCGGCAGATTGAATGCGCCTGGAGAAGGAAAACCGAAGCG
175	Mature_171	bta-miR-6_10707	chr6	17124222	17124242	+	21	10	10	0	0	0	0	0	Precursor_175	chr6_10707|chr6:17124185-17124242:+	58	10	GTCAGGAATTGGAGAAAGGAC	CCTTTCTTCTGCCCTTGAGAGCAGCAGGTTTGATGATGTCAGGAATTGGAGAAAGGAC
176	Mature_172	bta-miR-6_12617	chr6	22459391	22459410	+	20	65	0	0	65	0	0	0	Precursor_176	chr6_12617|chr6:22459358-22459410:+	53	65	AGAAAGAAAGGAAGGGGAGG	TCACCCCTTCTAAGTTTGCAGATGGAATTAATCAGAAAGAAAGGAAGGGGAGG
177	Mature_173	bta-miR-6_11286	chr6	42246307	42246327	+	21	255	0	0	0	0	0	255	Precursor_177	chr6_11286|chr6:42246250-42246327:+	78	351	CTTCCCAGGTGGCTTAGTGGT	ACAGTGATTTTGGATACCCCGATACAAAAGGTTTTTGGTGTTAAAAAAAAAAAAAGGCTTCCCAGGTGGCTTAGTGGT
178	Mature_174	bta-miR-6_12775	chr6	45782207	45782225	+	19	21	0	0	21	0	0	0	Precursor_178	chr6_12775|chr6:45782178-45782225:+	48	21	GGCAGAAGAGGAGATAGAG	CTATTTCCAAAACTTCTGGAAGAGAAAATGGCAGAAGAGGAGATAGAG
179	Mature_175	bta-miR-6_11073	chr6	75204430	75204452	+	23	311254	127502	0	183752	0	0	0	Precursor_179	chr6_11073|chr6:75204366-75204452:+	87	312105	CCCCACTGCTAAATTTGACTGGC	GGTCCGATGGTAGTGGGTTACCAGAACTTATTAACATTAGTGTCACTAAAGTTGGTATACAACCCCCCACTGCTAAATTTGACTGGC
180	Mature_176	bta-miR-6_12030	chr6	82586106	82586123	-	18	34	0	0	0	34	0	0	Precursor_180	chr6_12030|chr6:82586106-82586156:-	51	34	CTGAGCAGAAAAGTGGGG	CTGCTCTTTTCTGCTCTACATCAGAGGAGACATCTGAGCAGAAAAGTGGGG
181	Mature_177	bta-miR-6_11684	chr6	83132969	83132989	+	21	36	0	0	0	0	36	0	Precursor_181	chr6_11684|chr6:83132969-83133025:+	57	36	CCCTGAATCAATCACTGTAGC	CCCTGAATCAATCACTGTAGCAAAAAAAAAAGTGAAAGTGCTATTGTTCAATTTGGT
182	Mature_178	bta-miR-6_12542	chr6	88905156	88905180	-	25	50	0	0	0	0	0	50	Precursor_182	chr6_12542|chr6:88905156-88905228:-	73	50	ACCTCAGTGAACACCCAGCACAGCC	CTTGCTGGAATTGCTGAGTCCGTGTGCTTCAACAAGAGAAGCCAGTGCACCTCAGTGAACACCCAGCACAGCC
183	Mature_179	bta-miR-6_11247	chr6	90432382	90432399	+	18	163	0	0	0	106	57	0	Precursor_183	chr6_11247|chr6:90432318-90432399:+	82	170	ATGCCACATGGTGTGGGC	TCACAGCAGAGGGCATGAGTTTGGGTTTGATTCCTGGTCAGGGAACCAAGATCCAACATCACACATGCCACATGGTGTGGGC
184	Mature_180	bta-miR-6_14151	chr6	91667795	91667812	-	18	9	0	0	9	0	0	0	Precursor_184	chr6_14151|chr6:91667737-91667812:-	76	9	GGGAGAGGGGCAGGAAGG	GGGAGAGGGGCAGGAAGGAAGCCTCTTCAGAGGGCATGCTGTAATGCGTTCCTGCTTTCCACGTGCACTCACTGGT
185	Mature_181	bta-miR-6_12097	chr6	98289444	98289465	-	22	28	28	0	0	0	0	0	Precursor_185	chr6_12097|chr6:98289381-98289465:-	85	28	GGGGAAAAAGAAGAGAGGAAGG	GGGGAAAAAGAAGAGAGGAAGGAAAAAAAAAAAAAAAACTTTCAGAAGGACAGCAATTTCCTTCCTTCCTCTTCGGGCTCCTCCT

186	Mature_182	bta-miR-6_11279	chr6	98573349	98573366	+	18	295	116	0	179	0	0	0	Precursor_186	chr6_11279|chr6:98573290-98573366:+	77	295	CATATGAGTAAGTGGAGG	TGCCTTTTACAAATGTGGAGACCTTGGCCTCAAGAAATTAAATACCTTGCCCAAGATCCCATATGAGTAAGTGGAGG
187	Mature_183	bta-miR-6_13333	chr6	102014487	102014511	+	25	149	0	0	149	0	0	0	Precursor_187	chr6_13333|chr6:102014426-102014511:+	86	149	GGATGAGGGGGGAAGAAAGAGAGAG	TTCTCATGACTTCTAACAACTTAGAAAATATGACAAAGTAACCTAACTTTGTCATAGGAGTGGATGAGGGGGGAAGAAAGAGAGAG
188	Mature_184	bta-miR-6_13351	chr6	103411297	103411315	+	19	31	0	0	31	0	0	0	Precursor_188	chr6_13351|chr6:103411264-103411315:+	52	31	GAGGCAGGCAAGGAGAAGC	GGGTCCCCTGCCTTTTCTTACAGCTTGAGGGAGGAGGCAGGCAAGGAGAAGC
189	Mature_185	bta-miR-6_10712	chr6	110290122	110290140	+	19	40	0	40	0	0	0	0	Precursor_189	chr6_10712|chr6:110290122-110290173:+	52	40	AAGGAGGAGAAGGGGGTTT	AAGGAGGAGAAGGGGGTTTCAAGAGAGGATGGGACTCCTAAGTCACCCTTCC
190	Mature_186	bta-miR-6_10720	chr6	111996408	111996426	+	19	14	0	14	0	0	0	0	Precursor_190	chr6_10720|chr6:111996408-111996487:+	80	14	AGGGCCTGAGTGGCAGGGC	AGGGCCTGAGTGGCAGGGCGATCTGATGAGGCAGCGACTCGTGTATCTTGAGAAAGTCACACCATAAAATCAGGCCCAGA
191	Mature_187	bta-miR-6_12209	chr6	113163871	113163890	-	20	10	10	0	0	0	0	0	Precursor_191	chr6_12209|chr6:113163805-113163890:-	86	10	AGGAGGAGAAGGGGGGGGGG	AGGAGGAGAAGGGGGGGGGGGGTGGTCAAAAGGAGAGAGACAGATCTAGCCAATAATCAGTTCCCAAAGTGTTCTCCATAGTCTGG
192	Mature_188	bta-miR-6_12326	chr6	116169091	116169113	-	23	41	0	0	0	41	0	0	Precursor_192	chr6_12326|chr6:116169091-116169165:-	75	41	TCCCCGCCGCTCCTTCCCCCTCC	AGGCGGGGGGGGGCGGTGAGGGGGGGGGAGGAGGGCGCCCACACCCCTCCCCCTCCCCGCCGCTCCTTCCCCCTCC
193	Mature_189	bta-miR-7_13982	chr7	5383316	5383333	-	18	26	0	0	0	0	26	0	Precursor_193	chr7_13982|chr7:5383316-5383389:-	74	89	TCTGCCTTGCAGTGGGGC	TTCTAATGCAGGGGGTGTGGGTTTGATCCCTGGTCAGGGAACTAAGATCCCACATATCTGCCTTGCAGTGGGGC
194	Mature_190	bta-miR-7_13249	chr7	6059492	6059509	-	18	20	20	0	0	0	0	0	Precursor_194	chr7_13249|chr7:6059448-6059509:-	62	20	GGAAGAAAAAAAGGGAAG	GGAAGAAAAAAAGGGAAGGAAGAAAATCTAGGTCAGTCTCCTGGTCTTTCCCCTTCTTGCCT
195	Mature_191	bta-miR-7_12379	chr7	6071229	6071253	+	25	33	0	0	0	33	0	0	Precursor_195	chr7_12379|chr7:6071203-6071253:+	51	33	ATCCTAAAAGTCAGGTGGTGTGGCC	GCATATTCCCTGATCGGGAAACTAAGATCCTAAAAGTCAGGTGGTGTGGCC
196	Mature_192	bta-miR-7_12309	chr7	6398071	6398091	+	21	123	34	0	89	0	0	0	Precursor_196	chr7_12309|chr7:6398014-6398090:+	79	123	TGGGGGAAGAAAAAAGGGGGA	GCCTGTAAGCCTTCCCTTAAGCCATTTTAAAGAGTTTTCACATCTTGAAAACGGGCTTTGGGGGAAGAAAAAAGGGGGA
197	Mature_193	bta-miR-7_13266	chr7	6880690	6880710	-	21	34	34	0	0	0	0	0	Precursor_197	chr7_13266|chr7:6880690-6880732:-	43	34	GACAAGAGACAAGTTTCCAAG	TGGGAACAAGTGCGATGTGAATGACAAGAGACAAGTTTCCAAG
198	Mature_194	bta-miR-7_15631	chr7	7729778	7729795	-	18	16	0	0	16	0	0	0	Precursor_198	chr7_15631|chr7:7729739-7729795:-	57	16	GGAGCCAGGGAAAGGGGG	GGAGCCAGGGAAAGGGGGGTTGGACACCAACTCCAACCCACCTGACTCACGCTCCCA
199	Mature_195	bta-miR-7_11665	chr7	11574530	11574550	+	21	43	0	43	0	0	0	0	Precursor_199	chr7_11665|chr7:11574480-11574550:+	71	43	CGCAAGCCATGCAGTGGAGCC	CTTTCACTGCTGTGGCCCAGGTTCAATCCCCAGTCAGGGAACTGAGATCCCGCAAGCCATGCAGTGGAGCC
200	Mature_196	bta-miR-7_12457	chr7	14144247	14144267	+	21	372	0	0	0	372	0	0	Precursor_200	chr7_12457|chr7:14144216-14144267:+	52	372	CAGGGCAACATAACTGGGAGG	TGCCAAGTCTGTGGCGAGCCTTGGAACCTCACAGGGCAACATAACTGGGAGG
201	Mature_197	bta-miR-7_12496	chr7	16788495	16788514	+	20	32	0	0	0	14	18	0	Precursor_201	chr7_12496|chr7:16788495-16788582:+	88	36	CATTTGGGTGGTGATTAGGG	CATTTGGGTGGTGATTAGGGTTAGATAAGGTCATGAGGGTGGGGCCCTCATGATGGGATTGGTGCCCTTGTAAGAGACATCAGAGAGA
202	Mature_198	bta-miR-7_14609	chr7	17067414	17067436	+	23	36	0	0	36	0	0	0	Precursor_202	chr7_14609|chr7:17067381-17067436:+	56	36	TGCTCAGAATGAGAAGAGGAAGG	CTCCTCTTTTGAATTCAGTGAAGGAGAACGACCTGCTCAGAATGAGAAGAGGAAGG
203	Mature_199	bta-miR-7_13389	chr7	18318731	18318751	-	21	20	11	0	9	0	0	0	Precursor_203	chr7_13389|chr7:18318707-18318751:-	45	20	GGAGGAGAGAAGAGGAGGAAG	GGAGGAGAGAAGAGGAGGAAGCCAGAGGCTCTCTTTCTCCTCTTT
204	Mature_200	bta-miR-7_12531	chr7	19008117	19008141	+	25	116	0	0	0	116	0	0	Precursor_204	chr7_12531|chr7:19008070-19008141:+	72	116	CCGTGCCCCTTTTTGCTAGAACTGG	GGTGGCCGCAGCAAGGGGGCAAGGGGAGTCAGTCAATTGGCAAGACACCGTGCCCCTTTTTGCTAGAACTGG
205	Mature_201	bta-miR-7_13515	chr7	20288333	20288353	-	21	646	0	0	0	189	174	283	Precursor_205	chr7_13515|chr7:20288286-20288353:-	68	646	GGGAATCCTGGGTGCTGTGGG	GGGAATCCTGGGTGCTGTGGGGTGCAGAGCAACATCACTGCCCCACCTCTTCAGTGCCAGAAGCCCCC
206								0							Precursor_206	chr7_14154|chr7:20288333-20288413:-	81	174		CAACCTCAGGGTCACCCCCACCCCCAGCACTGCTGACTTGGGGCTAGTCACTCTCTGTGGGGGAATCCTGGGTGCTGTGGG
207	Mature_202	bta-miR-7_13522	chr7	30860538	30860557	-	20	15	15	0	0	0	0	0	Precursor_207	chr7_13522|chr7:30860538-30860576:-	39	15	CCAGGGAGAAGGAAGAGGAG	CCTCTTTCCTTCTGGAGGTCCAGGGAGAAGGAAGAGGAG
208	Mature_203	bta-miR-7_12934	chr7	37693705	37693723	-	19	16	0	16	0	0	0	0	Precursor_208	chr7_12934|chr7:37693705-37693757:-	53	16	TCCCCCCTCCTTCCTCTCC	AGAGGGTTTGGGGAGGAAAAGACAAAAAGTAGTTTCCCCCCTCCTTCCTCTCC
209	Mature_204	bta-miR-7_12605	chr7	38964511	38964528	+	18	14	14	0	0	0	0	0	Precursor_209	chr7_12605|chr7:38964461-38964528:+	68	14	GAGGAGGGGGGGGAGGAG	CAGCCCCTCCCTCAAGCTCCCTTTCCCTGACTGCCAAGGCAGGGTGCTAGGAGGAGGGGGGGGAGGAG
210	Mature_205	bta-miR-7_11971	chr7	43632358	43632379	+	22	18	0	18	0	0	0	0	Precursor_210	chr7_11971|chr7:43632330-43632379:+	50	18	CCCAACCCAATGGGGTGTGGCC	GTTCAATCCGTGGTTGGGAAACTAAGATCCCAACCCAATGGGGTGTGGCC
211	Mature_206	bta-miR-7_13646	chr7	44910193	44910211	-	19	10	10	0	0	0	0	0	Precursor_211	chr7_13646|chr7:44910193-44910261:-	69	10	AGGCTTGAGAAGAAGGAGG	TTCGGAGGCTCTGGCCTGTGAGATGGGAGTGGAGGACAGGTCGAGCTGAAAGGCTTGAGAAGAAGGAGG
212	Mature_207	bta-miR-7_14997	chr7	50721398	50721416	+	19	107	0	0	107	0	0	0	Precursor_212	chr7_14997|chr7:50721348-50721416:+	69	107	AGGAAGGAGAAGGAAGGAA	CCTTCGAGAGTATCTCCTTTCTGATTAAGGAGATAGAGCCTGAAAGGAGGAGGAAGGAGAAGGAAGGAA
213	Mature_208	bta-miR-7_12780	chr7	53420967	53420990	+	24	12	12	0	0	0	0	0	Precursor_213	chr7_12780|chr7:53420967-53421035:+	69	12	AGTGACATCTGCATTAATGAGGGG	AGTGACATCTGCATTAATGAGGGGGAAAAGAAAAAGGATCCCTCATTGATGGAGAAAGAGTCCCCCTGG
214	Mature_209	bta-miR-7_16223	chr7	60892440	60892463	-	24	12	0	0	12	0	0	0	Precursor_214	chr7_16223|chr7:60892382-60892463:-	82	12	GGGGCGGGGGGAGTCCAGGACAAC	GGGGCGGGGGGAGTCCAGGACAACAAAAAAAGATTTAGAACTCCTGATGCAAAACTAGTGACTCCAACTCCCACCCACCCAC
215	Mature_210	bta-miR-7_16251	chr7	62692654	62692671	-	18	40	0	0	40	0	0	0	Precursor_215	chr7_16251|chr7:62692623-62692671:-	49	40	GGAAAGATAGAGAAAGAG	GGAAAGATAGAGAAAGAGCACTTTGGCTCCTCTTATTATATGTTTCTTC
216	Mature_211	bta-miR-7_14585	chr7	65803739	65803762	-	24	54	0	0	0	0	54	0	Precursor_216	chr7_14585|chr7:65803686-65803762:-	77	54	CCTGCTGTGCGGTGTGGCCAAGGG	CCTGCTGTGCGGTGTGGCCAAGGGAAAAAAAAAAAAAAAAGGCATAGCCTCCTAACTGGTCACCCTGTCACCAGGCT
217	Mature_212	bta-miR-7_13865	chr7	71032115	71032132	-	18	8	8	0	0	0	0	0	Precursor_217	chr7_13865|chr7:71032096-71032132:-	37	8	AGGGAAGCCAGGAAGCAG	AGGGAAGCCAGGAAGCAGTAGCTACCTGCTTCTTCAG
218	Mature_213	bta-miR-7_15230	chr7	71768354	71768373	+	20	17	0	0	17	0	0	0	Precursor_218	chr7_15230|chr7:71768354-71768424:+	71	17	AGGGAACCAGAAAAAGAGAA	AGGGAACCAGAAAAAGAGAACCAGGCATGGCTTTCTTGACTTAAAAGAAGCCTCTCTTTTTTCTTGGCTCT
219	Mature_214	bta-miR-7_13294	chr7	80617103	80617126	-	24	72	0	72	0	0	0	0	Precursor_219	chr7_13294|chr7:80617103-80617152:-	50	72	TACCACATGCTGCACAGCACAGCC	CTGTATTGTGGGTTGGGGAACTAAGATACCACATGCTGCACAGCACAGCC
220	Mature_215	bta-miR-7_12333	chr7	80822680	80822700	+	21	18	0	18	0	0	0	0	Precursor_220	chr7_12333|chr7:80822635-80822700:+	66	18	CTGAAAAAACCTTTTACATAG	AAGCAGAGGGTTTTTCTTCTGGGAGGACAAAAGTCGTCTCCTCCTCTGAAAAAACCTTTTACATAG
221	Mature_216	bta-miR-7_13302	chr7	82208643	82208665	-	23	12	0	12	0	0	0	0	Precursor_221	chr7_13302|chr7:82208643-82208728:-	86	12	CCTACATGCCGTGCAGCGTGACC	TTAAGACTCTGCACTGCCAATGTTGGGGGCCTGGGTTCGATCCCTGGTCAAGTAAACTAAGATCCTACATGCCGTGCAGCGTGACC
222	Mature_217	bta-miR-7_13472	chr7	104024117	104024140	-	24	14	0	14	0	0	0	0	Precursor_222	chr7_13472|chr7:104024117-104024196:-	80	14	TCCCACCTGCTGTGGAGTGTGGCC	CTTCCACTGCAGGGGGCGCGAGTTCCATATCTGGTCCAGGGACTAAGATCTCACATTCCCACCTGCTGTGGAGTGTGGCC
223	Mature_218	bta-miR-7_13128	chr7	104053683	104053700	+	18	13	13	0	0	0	0	0	Precursor_223	chr7_13128|chr7:104053683-104053738:+	56	13	GACTGAAGAGGAGAAAAG	GACTGAAGAGGAGAAAAGCCACCAGCCCAATTTTGGCTTCTCTTTTTATATGTTTT
224	Mature_219	bta-miR-8_14233	chr8	17579921	17579940	+	20	65	65	0	0	0	0	0	Precursor_224	chr8_14233|chr8:17579859-17579940:+	82	65	GAAATGGGAAGTGAAAGTTG	ACTTTTCTTCTCCTTTTCCCTTCATTTCCTGTTTGAACTCTAAAATGAAGTAGGTTAAGGGTGAAATGGGAAGTGAAAGTTG
225	Mature_220	bta-miR-8_14522	chr8	18137748	18137771	-	24	17	0	17	0	0	0	0	Precursor_225	chr8_14522|chr8:18137748-18137818:-	71	17	AGCTGTGAGAAACAAACAAAGGGC	CAGGCATTGTTTCCAGGCACTGGGACACAGCACTGAACTGATGTTCCAGCTGTGAGAAACAAACAAAGGGC
226	Mature_221	bta-miR-8_16160	chr8	30180581	30180598	-	18	33	0	0	0	0	0	33	Precursor_226	chr8_16160|chr8:30180581-30180654:-	74	33	GGAGGAAGGTGGGAAAAT	AAATGTGCATTTCCTCTCCTTTCTCCCAAAGTGCCTTGTGTGGGGTAGGGGAGTGGGGAGGAAGGTGGGAAAAT
227	Mature_222	bta-miR-8_16864	chr8	34413865	34413882	+	18	79	0	0	79	0	0	0	Precursor_227	chr8_16864|chr8:34413865-34413916:+	52	79	GAGAGAGAGAAAAGAAAG	GAGAGAGAGAAAAGAAAGGTGGTCAGTAAAACTCTGTGATATCTCTCCTTCT
228	Mature_223	bta-miR-8_16928	chr8	41527770	41527787	+	18	11	0	0	11	0	0	0	Precursor_228	chr8_16928|chr8:41527770-41527820:+	51	11	AGGAGGGGAGAGGAAAAG	AGGAGGGGAGAGGAAAAGTTACCACAGGTCTTTCCTCTCTGAACCTTCTCA
229	Mature_224	bta-miR-8_17906	chr8	44767167	44767185	-	19	39	0	0	39	0	0	0	Precursor_229	chr8_17906|chr8:44767167-44767247:-	81	39	GGGGAAAGGAAGAGGGGAG	TTCCAGGGGATCTTCCTGACCCAGTGATCGAACCTGCATCTCTTGCATTGGTAGGTTACTGAGGGGAAAGGAAGAGGGGAG
230	Mature_225	bta-miR-8_16946	chr8	44935985	44936005	+	21	18	0	0	18	0	0	0	Precursor_230	chr8_16946|chr8:44935985-44936056:+	72	18	GAGACACAGCACCTGGGAAGC	GAGACACAGCACCTGGGAAGCAAAACGAAGAGAAAACCTATAAAAAGGTGAATGAGTGTGTTGTTTTCTCTT
231	Mature_226	bta-miR-8_15314	chr8	55032253	55032271	-	19	83	83	0	0	0	0	0	Precursor_231	chr8_15314|chr8:55032253-55032329:-	77	83	GGAGAAGAAGAAGGAAGAG	GTTCTTTCTCTCTGTTCCCTTGTATGAGGAAGGAAGATTGCCCCTTTTGGGGTTACTGGGAGAAGAAGAAGGAAGAG
232	Mature_227	bta-miR-8_18036	chr8	59863182	59863206	-	25	18	0	0	18	0	0	0	Precursor_232	chr8_18036|chr8:59863182-59863235:-	54	18	GGAAGAGGAGCAGCAGGCCCTCCAG	TGGCTGACTGATGTTTTTCTCTCCCAGCTGGAAGAGGAGCAGCAGGCCCTCCAG
233	Mature_228	bta-miR-8_14866	chr8	63198403	63198423	-	21	18	0	18	0	0	0	0	Precursor_233	chr8_14866|chr8:63198403-63198473:-	71	18	CGCTAGCCACATGATGAGGCC	CACTCTCACTGCCTTGGCCCGGGTTCAGTCCCTGGTGGGAAATGAGATCCCGCTAGCCACATGATGAGGCC
234	Mature_229	bta-miR-8_14868	chr8	64109110	64109132	-	23	51	0	51	0	0	0	0	Precursor_234	chr8_14868|chr8:64109110-64109162:-	53	51	CCTGCATGTCATGCAGTGTGGCC	CCACGCTTCTACTGCAGGGGGAACCAAAATCCTGCATGTCATGCAGTGTGGCC
235	Mature_230	bta-miR-8_17123	chr8	64881855	64881875	+	21	27	0	0	27	0	0	0	Precursor_235	chr8_17123|chr8:64881802-64881875:+	74	27	GAAACAGGCAGCCAGAAGAGG	TCTTCAGCAGATCTGATTTTTACAGTGTTTCAGTGAAAATACTGAAAGGCAGTGAAACAGGCAGCCAGAAGAGG
236	Mature_231	bta-miR-8_15415	chr8	68717695	68717714	-	20	2586	727	0	1859	0	0	0	Precursor_236	chr8_15415|chr8:68717673-68717714:-	42	2586	GGAGGAGAAAGGAAAGGAGT	GGAGGAGAAAGGAAAGGAGTAAGCCTTTCCAAGTCTCCCCCC
237	Mature_232	bta-miR-8_16576	chr8	73688533	73688557	-	25	21	0	0	0	0	21	0	Precursor_237	chr8_16576|chr8:73688533-73688609:-	77	28	CCCATGTGCCGCTTGGAGTGGACAG	GTGCTTCCCATGCAGGGGGTGCATGGTAAATCCCTGGTTGGGGAACTAGGATCCCATGTGCCGCTTGGAGTGGACAG
238	Mature_233	bta-miR-8_17212	chr8	73726285	73726302	+	18	23	0	0	23	0	0	0	Precursor_238	chr8_17212|chr8:73726285-73726350:+	66	23	AGAGGAGGAAAGAGGAAG	AGAGGAGGAAAGAGGAAGATGTATGCTCTAGTTGTAGGGGAGCAGTCTTTCTGCTTCTTCTTTCTG
239	Mature_234	bta-miR-8_16607	chr8	76682532	76682554	-	23	72	0	0	0	0	72	0	Precursor_239	chr8_16607|chr8:76682470-76682554:-	85	72	CTCCCCTTGCCTCTTGTTTGGGG	CTCCCCTTGCCTCTTGTTTGGGGGAAAAAAAAAAAAAGCTTTGGCTTCCTGGACCTTTCCTGAATTCTAAAGAGCAAGAGGAGTG
240	Mature_235	bta-miR-8_14141	chr8	80003885	80003902	+	18	253	0	253	0	0	0	0	Precursor_240	chr8_14141|chr8:80003834-80003902:+	69	253	GGAGGGGAAGGGGAAAAG	CATCTCTCCCCTCCCTCCAATTTCCCTCTTATAACTAAGAAATGGGGTTGGGGAGGGGAAGGGGAAAAG
241	Mature_236	bta-miR-8_15084	chr8	88927193	88927210	-	18	18	0	18	0	0	0	0	Precursor_241	chr8_15084|chr8:88927193-88927251:-	59	18	CTACATCCCACCTTGTGC	ATGGGGCTGGGGTTTCTATCCCTGGTCAAGGAACTAAGGTCCTACATCCCACCTTGTGC
242	Mature_237	bta-miR-8_15833	chr8	95735405	95735428	+	24	54	0	0	0	0	54	0	Precursor_242	chr8_15833|chr8:95735339-95735428:+	90	54	GAGGACTAATTACAAGGCAGAAAG	GACTGCCGGTGAAAGACAGAAAGTTCCTGGCTTCTTCATGCCCCATAGCAAAAGGCTTTAGAGCCAGAGGACTAATTACAAGGCAGAAAG
243	Mature_238	bta-miR-8_18492	chr8	105000426	105000443	-	18	71	0	0	71	0	0	0	Precursor_243	chr8_18492|chr8:105000426-105000513:-	88	71	AGAAGGAAAGGAAGAGGG	CCATCTCTCCGAAACTCTTCTTCCTCAACTGCAGGGCATGCATTTGCTTCTGTGAAGAAGATGGAAGAGAAGAAGGAAAGGAAGAGGG
244	Mature_239	bta-miR-8_15230	chr8	108994668	108994691	-	24	13	0	13	0	0	0	0	Precursor_244	chr8_15230|chr8:108994668-108994724:-	57	13	CCAGCCAGCTGGGCCCGTACCTGC	AGGAAGACGTGCCCCTGGGGGATCCTGTGTCCCCCAGCCAGCTGGGCCCGTACCTGC
245	Mature_240	bta-miR-8_15245	chr8	111231235	111231252	-	18	70	0	70	0	0	0	0	Precursor_245	chr8_15245|chr8:111231182-111231252:-	71	70	AAGGAGGGGAAGGGGGCG	AAGGAGGGGAAGGGGGCGCTGGATGGGGGCTGAGACCCTCACAACAAAAGCTGTTCCTTTCTCTTCATTCT
246	Mature_241	bta-miR-9_16064	chr9	8075912	8075931	-	20	28	0	28	0	0	0	0	Precursor_246	chr9_16064|chr9:8075912-8075984:-	73	28	ACTCCACAAGAAGAGGAAGA	TACGCCTTCTTGAGGGGGTTAAAGATGGGGGAGAATCCACCCCCGACTGAACTACTCCACAAGAAGAGGAAGA
247	Mature_242	bta-miR-9_15349	chr9	12611607	12611624	+	18	14	0	14	0	0	0	0	Precursor_247	chr9_15349|chr9:12611568-12611624:+	57	14	CAGATTGCTAGCCAAGAG	CTGTATATAGTCATCTGTCCACTGCAAATTAAGCATCCACAGATTGCTAGCCAAGAG
248	Mature_243	bta-miR-9_19583	chr9	25519330	25519347	-	18	8	0	0	8	0	0	0	Precursor_248	chr9_19583|chr9:25519286-25519347:-	62	8	CCCGCGGGGCGCGCGCCT	CCCGCGGGGCGCGCGCCTGGGCGCTCCTCCCTCCTGCCCCAGCTCCGCGTCACCGCGCGGTC
249	Mature_244	bta-miR-9_18033	chr9	36281145	36281162	-	18	19	0	0	0	0	0	19	Precursor_249	chr9_18033|chr9:36281126-36281162:-	37	19	GGAGAGGGAAGTGGCAGC	GGAGAGGGAAGTGGCAGCTTGCTTATTTTCTTTTTTT
250	Mature_245	bta-miR-9_16048	chr9	36522575	36522593	+	19	62	62	0	0	0	0	0	Precursor_250	chr9_16048|chr9:36522575-36522632:+	58	62	GGGAAGAGAAAGAAAGAGG	GGGAAGAGAAAGAAAGAGGAAAAGAAAAAAAAAAAAATCCTGGCTTTTTCTTTTTAT
251	Mature_246	bta-miR-9_16052	chr9	37036021	37036038	+	18	34	15	0	19	0	0	0	Precursor_251	chr9_16052|chr9:37036021-37036065:+	45	34	GAGAGAAGACAAGAAAGG	GAGAGAAGACAAGAAAGGCATAAAACGTGGCCTTGCTTCCCTTAC
252	Mature_247	bta-miR-9_18811	chr9	37087394	37087414	+	21	11	0	0	11	0	0	0	Precursor_252	chr9_18811|chr9:37087326-37087414:+	89	11	AGGGGCCCAGTGAAGAGGATG	ACCTTGGAACCCACTGGGGATTTGCTTTTATGGACATCCATCTGCTTTCTGATGGCTTTAATGAGAGTAGGGGCCCAGTGAAGAGGATG
253	Mature_248	bta-miR-9_16329	chr9	44468118	44468137	+	20	26	0	0	0	13	13	0	Precursor_253	chr9_16329|chr9:44468069-44468137:+	69	26	AGAGGAGTCATTCTGTGGCA	CCACTGAACTTCCTCAGAGCTCTTTGAAGTCAGCAATTACTTTGTTCTCAGAGGAGTCATTCTGTGGCA
254	Mature_249	bta-miR-9_18924	chr9	50370033	50370053	+	21	16	0	0	16	0	0	0	Precursor_254	chr9_18924|chr9:50370033-50370085:+	53	16	GACAGTGAAGATGAGAGGAGC	GACAGTGAAGATGAGAGGAGCAACCGAGGTTCTGAATCATCTGACACTGATGA
255	Mature_250	bta-miR-9_16363	chr9	50597908	50597930	-	23	34	0	34	0	0	0	0	Precursor_255	chr9_16363|chr9:50597861-50597930:-	70	34	GTCCTCCTGGCATACACACAAGC	GTCCTCCTGGCATACACACAAGCAAGGAAAAAGAAAAAATTCCTTATTGTGGTGTCTGGTTTGAGATTGT
256	Mature_251	bta-miR-9_17239	chr9	50750488	50750506	-	19	23	0	0	0	23	0	0	Precursor_256	chr9_17239|chr9:50750433-50750506:-	74	23	CTGCTTATTGCTGAATTCC	CTGCTTATTGCTGAATTCCTACCACTCAGAAGAGTGCATGGCTCATAATAGGAATTCCATCAGCATCTGCTAAG
257	Mature_252	bta-miR-9_16401	chr9	56676036	56676054	-	19	1609	0	1138	0	471	0	0	Precursor_257	chr9_16401|chr9:56675983-56676054:-	72	1609	GGGGTGGGGGGGCGGCGGG	GGGGTGGGGGGGCGGCGGGAATGTGGTCTTGTTCTGGTGGGCAGGGCCATTCTCAGTAAAATTTCATTGTGG
258	Mature_253	bta-miR-9_15713	chr9	60624064	60624087	+	24	19	0	19	0	0	0	0	Precursor_258	chr9_15713|chr9:60624012-60624087:+	76	20	CCACAAGCCATGAAGCAAGGCATC	TGCTTTCACTTTCAAGGGCTTGGGTTCAATTCCTGGTCTAGGAACTAAGATCCCACAAGCCATGAAGCAAGGCATC
259	Mature_254	bta-miR-9_16291	chr9	65233255	65233272	+	18	17	17	0	0	0	0	0	Precursor_259	chr9_16291|chr9:65233228-65233272:+	45	17	GAAAGAGAGGGGGAAAAG	CTTCTCTCTCTTAACTTGGTATCTATAGAAAGAGAGGGGGAAAAG
260	Mature_255	bta-miR-9_16342	chr9	71552864	71552882	+	19	20	20	0	0	0	0	0	Precursor_260	chr9_16342|chr9:71552864-71552910:+	47	20	AAGAGAGGAAGAGAGAGAG	AAGAGAGGAAGAGAGAGAGGCCAGTTTTTTAAATCCCTTTGATTTCT
261	Mature_256	bta-miR-9_16389	chr9	76864025	76864044	+	20	14	14	0	0	0	0	0	Precursor_261	chr9_16389|chr9:76863977-76864044:+	68	14	AGGTAAAGCGGATGCAGGAT	CCTGCCGCTGCGGCCAGGGCCACCTGAAGAAGGACACGGACTGGTACCAGGTAAAGCGGATGCAGGAT
262	Mature_257	bta-miR-9_18572	chr9	86246600	86246624	-	25	62	0	0	0	0	62	0	Precursor_262	chr9_18572|chr9:86246600-86246658:-	59	62	GTGGTATAACCTTCCTCTTCAGTGC	GGTGAAGGGGCAGCGGTATCATTAAAAAGTCATAGTGGTATAACCTTCCTCTTCAGTGC
263	Mature_258	bta-miR-9_20157	chr9	91048886	91048904	-	19	14	0	0	14	0	0	0	Precursor_263	chr9_20157|chr9:91048825-91048904:-	80	14	GAGGGAAGAGGGAAAAGGA	GAGGGAAGAGGGAAAAGGATGCCACGAGATTTCTTATTTTTAGATAAGGCTGATTTGTGCTTCTTGCCCTCTCCTCTCAT
264	Mature_259	bta-miR-9_18675	chr9	96299288	96299309	-	22	74	0	0	0	0	74	0	Precursor_264	chr9_18675|chr9:96299247-96299309:-	63	74	CCTGGAATCGAAGACATCATGC	CCTGGAATCGAAGACATCATGCAAAAAAAAAAAAAAGGCTGCAGCTGTTACTGACTCGGGAGG
265	Mature_260	bta-miR-10_20345	chr10	6134082	6134104	+	23	30	0	0	30	0	0	0	Precursor_265	chr10_20345|chr10:6134057-6134104:+	48	30	GGGAAGGAAGAGAAGAAAGGGGG	CAAGTTCTTAATTCCTGTGGCCCTGGGGAAGGAAGAGAAGAAAGGGGG
266	Mature_261	bta-miR-10_16819	chr10	6394881	6394903	+	23	37	0	37	0	0	0	0	Precursor_266	chr10_16819|chr10:6394834-6394903:+	70	37	CCCTGTAATAGGAGAGTGCTGGC	CTTCTGCACTGCAGGGAAATTCTTTACCATGGAGCCATGGGGGAGGCCCCTGTAATAGGAGAGTGCTGGC
267	Mature_262	bta-miR-10_17651	chr10	7057631	7057650	-	20	186	0	186	0	0	0	0	Precursor_267	chr10_17651|chr10:7057598-7057650:-	53	186	AAAACTAATTTTTTGAGGCC	AAAACTAATTTTTTGAGGCCTTGCTTCGGCAAGGCTACTTATATTGGTTAATG
268	Mature_263	bta-miR-10_18402	chr10	10807642	10807665	-	24	9	9	0	0	0	0	0	Precursor_268	chr10_18402|chr10:10807642-10807726:-	85	9	GGGAGGGAAAGAGGAAGGGAAAAG	TGTCCTTATCCTCTGTGCTTTTCTGCTTCTCCCAGGAGACTCTGGGCTGGACTGGGATGGAGGGAGGGAAAGAGGAAGGGAAAAG
269	Mature_264	bta-miR-10_18598	chr10	34134054	34134075	-	22	12	12	0	0	0	0	0	Precursor_269	chr10_18598|chr10:34134054-34134128:-	75	12	CTCATTCCTCCCTTTTGTGCCC	GCACTGAAGGGATGAATAACATGTGTCATAAAGAAATATAAAATCATCTCATTCTCATTCCTCCCTTTTGTGCCC
270	Mature_265	bta-miR-10_18607	chr10	36379451	36379468	-	18	29	29	0	0	0	0	0	Precursor_270	chr10_18607|chr10:36379423-36379468:-	46	29	AGGAGGAAAGAGGAGGAG	AGGAGGAAAGAGGAGGAGCCGTGGCCCCACTTTGCCCTTATCCTGG
271	Mature_266	bta-miR-10_17083	chr10	36847271	36847295	+	25	14	0	14	0	0	0	0	Precursor_271	chr10_17083|chr10:36847210-36847295:+	86	16	CAGGCTGTGTGATGCAGCAAAAATT	AATTTGGTGCTGTCACTGCTGTGGCTTGGGTTTGATCTCTGGTTGGGGAACTGAGATCTTACAGGCTGTGTGATGCAGCAAAAATT
272	Mature_267	bta-miR-10_17923	chr10	37668953	37668973	-	21	13	0	13	0	0	0	0	Precursor_272	chr10_17923|chr10:37668894-37668973:-	80	13	CCACATGTCAAGTGGCACAGC	CCACATGTCAAGTGGCACAGCTGGAAAAAAAAAAGGTCCAACTTATCTGTTTTTTTCTTCTGTTGCTCATGATTCTGGTG
273	Mature_268	bta-miR-10_17945	chr10	41330949	41330970	-	22	884	0	884	0	0	0	0	Precursor_273	chr10_17945|chr10:41330919-41330970:-	52	889	GGGGGGCCCAAGTCCTTCTGAT	GGGGGGCCCAAGTCCTTCTGATCAAGGCCTAGCCCGTGGATGGTGTGAGGCC
274	Mature_269	bta-miR-10_17753	chr10	42629564	42629584	+	21	9	9	0	0	0	0	0	Precursor_274	chr10_17753|chr10:42629529-42629584:+	56	9	GCGCGCGCCTGTAATCCCAGC	TGTCTTGCGCAGGTTTGCGATCGCCGGGCGCGGTGGCGCGCGCCTGTAATCCCAGC
275	Mature_270	bta-miR-10_20652	chr10	42888515	42888539	+	25	342	0	0	342	0	0	0	Precursor_275	chr10_20652|chr10:42888515-42888574:+	60	342	CCTGGTCTAGTGGTTAGGATTTGGC	CCTGGTCTAGTGGTTAGGATTTGGCACTGCCATGGTCCAGGTTCAATTCCTGACTAGGGA
276	Mature_271	bta-miR-10_17164	chr10	45384271	45384288	+	18	357	0	187	0	0	61	109	Precursor_276	chr10_17164|chr10:45384271-45384357:+	87	357	CCCCGGGGAGCCCGGCGC	CCCCGGGGAGCCCGGCGCTGGCCTTGGGACTCCGGCCCTCTGCCTGACAGGCAGCCCAAGGCCACGGTGGGCAGATGCCTTGGTCTC
277	Mature_272	bta-miR-10_20173	chr10	45853895	45853917	-	23	19	0	0	0	0	19	0	Precursor_277	chr10_20173|chr10:45853839-45853917:-	79	19	CAGCCAGAGAGGATTATAACTCC	CAGCCAGAGAGGATTATAACTCCAAAATAGAAAAAGGGCTGAGAATTTCAGCCTTGGAGAAAGTTATTTATGAAGTGTC
278	Mature_273	bta-miR-10_17183	chr10	48189610	48189628	+	19	15	0	15	0	0	0	0	Precursor_278	chr10_17183|chr10:48189576-48189628:+	53	23	CACCTGGATCTTAGGGAGC	CTCACCTAGAGCCAGACATCCTGGATTCCAAAGTCACCTGGATCTTAGGGAGC
279	Mature_274	bta-miR-10_17191	chr10	50097831	50097850	+	20	881	0	881	0	0	0	0	Precursor_279	chr10_17191|chr10:50097773-50097850:+	78	881	AAGGAGGGGAAGGGGAAAAT	TTTGCTTTTTCTCTGCTCTTGTGAACATACATCCTAGTGTTGACATAGCATCAGGCAAAAGGAGGGGAAGGGGAAAAT

280	Mature_275	bta-miR-10_21885	chr10	52134338	52134358	-	21	13	0	0	13	0	0	0	Precursor_280	chr10_21885|chr10:52134280-52134358:-	79	13	GGGGAGAAGGGCGTTGGAAGG	GGGGAGAAGGGCGTTGGAAGGAAAAGAGAGAGAAAATAAAAGTCCAAGATTCTTCTGATTCTAGTCACTCTTTACTGAA
281	Mature_276	bta-miR-10_18179	chr10	57814009	57814028	+	20	30	0	0	0	30	0	0	Precursor_281	chr10_18179|chr10:57813985-57814028:+	44	30	CTGTAACAGTCAGCAGGGTT	TCCTGCTACTGTGACGGACAAGCCCTGTAACAGTCAGCAGGGTT
282	Mature_277	bta-miR-10_20871	chr10	58821280	58821297	+	18	15	0	0	15	0	0	0	Precursor_282	chr10_20871|chr10:58821236-58821297:+	62	15	CAGGATCATCATGAAGTG	AAGTGTGGTATCCTATGACTGGTTGGAAGTCTTCAATTATACCTCAGGATCATCATGAAGTG
283	Mature_278	bta-miR-10_20885	chr10	59706248	59706266	+	19	21	0	0	21	0	0	0	Precursor_283	chr10_20885|chr10:59706186-59706266:+	81	21	GGGAAGGCTGAATATGAGG	TCAAGACTTAGACCTTTTGTAGCTCACACATGTACGCAGATGTTGTTATCTGATATGTGGATGGGAAGGCTGAATATGAGG
284	Mature_279	bta-miR-10_19378	chr10	71460963	71460985	+	23	47	0	0	0	0	47	0	Precursor_284	chr10_19378|chr10:71460963-71461040:+	78	47	CCCTTTTATTGTCTTGGGGAAGG	CCCTTTTATTGTCTTGGGGAAGGAAAAAAAAAAAAAACTCTGTCAAGCAGCTCCTTTCCCCAGGACACAGAATTTTAT
285	Mature_280	bta-miR-10_22067	chr10	72362539	72362558	-	20	14	0	0	14	0	0	0	Precursor_285	chr10_22067|chr10:72362480-72362558:-	79	16	CCTCTTATTTTAGAAGCGTG	CCTCTTATTTTAGAAGCGTGAAAAAAAAACATGACTGAAGGAGTATTTGCATTTTTCAAGCCACTGGAAAGAGAAATGG
286	Mature_281	bta-miR-10_18094	chr10	73836728	73836752	+	25	808	199	0	609	0	0	0	Precursor_286	chr10_18094|chr10:73836728-73836809:+	82	808	GGAGGGCGCCGGGGGCGCGAACGAC	GGAGGGCGCCGGGGGCGCGAACGACAAGAAAAAGTAAGCCCACTCCCCTCCGCCGGCCGCCTTCTCCCCCCGGCGCCCCCGC
287	Mature_282	bta-miR-10_17388	chr10	77482075	77482093	+	19	11	0	11	0	0	0	0	Precursor_287	chr10_17388|chr10:77482075-77482136:+	62	11	TCCATATCAATTAAATCTA	TCCATATCAATTAAATCTAATTTCTGATACCACCAAGAAATTGGATTTGATTGATCTGGGGT
288	Mature_283	bta-miR-10_17411	chr10	80810316	80810333	+	18	11	0	11	0	0	0	0	Precursor_288	chr10_17411|chr10:80810283-80810333:+	51	11	CTAGGGGTGTTCTGATGG	TTCAGGCCTCAGGACTCAGGTTAAAACCTAGTTCTAGGGGTGTTCTGATGG
289	Mature_284	bta-miR-10_21145	chr10	83640648	83640666	+	19	118	0	0	118	0	0	0	Precursor_289	chr10_21145|chr10:83640616-83640666:+	51	118	GCACTGGGAAGGAAAGATG	TCTGTCCCTGGTGCCTGAGGTCAGCAGAGCAGGCACTGGGAAGGAAAGATG
290	Mature_285	bta-miR-10_21180	chr10	85560380	85560397	+	18	13	0	0	13	0	0	0	Precursor_290	chr10_21180|chr10:85560380-85560436:+	57	36	CCTTTATCTTAAATACGG	CCTTTATCTTAAATACGGTTAAAGAATGTAAAAGATGGGGAGAGAGAAGGAAGGAGG
291	Mature_286	bta-miR-10_17462	chr10	86084696	86084718	+	23	70	0	15	0	0	55	0	Precursor_291	chr10_17462|chr10:86084697-86084767:+	73	70	CTCCTTTCCCTTTTTCATTCTGC	CTCCTTTCCCTTTTTCATTCTGCAAAGGAAAAAAAAAAGTTGTTTCCAGCATGAGAGAAGACTGAGAAGAGAC
292	Mature_287	bta-miR-10_19087	chr10	88431698	88431715	-	18	8	8	0	0	0	0	0	Precursor_292	chr10_19087|chr10:88431698-88431733:-	36	8	GGAAACTTGGTGAGGCAG	GCCAGACTGGTCAGCATTGGAAACTTGGTGAGGCAG
293	Mature_288	bta-miR-10_17504	chr10	90354187	90354206	+	20	18	0	18	0	0	0	0	Precursor_293	chr10_17504|chr10:90354126-90354206:+	81	54	CTGGTAAAGAATCCACCTGC	AGGCATGGGTTCGAATCCCACTCCTGACAAGCCAGCCTTTTGGGCTTCCCTGGTAGCTCAGCTGGTAAAGAATCCACCTGC
294	Mature_289	bta-miR-10_20319	chr10	91732710	91732728	-	19	485	0	0	0	0	0	485	Precursor_294	chr10_20319|chr10:91732689-91732728:-	40	485	GGGTGTGGCTGGTGGGTTT	GGGTGTGGCTGGTGGGTTTGTGACCCACACAGCCCAGCAG
295	Mature_290	bta-miR-10_22315	chr10	100608540	100608557	-	18	13	0	0	13	0	0	0	Precursor_295	chr10_22315|chr10:100608493-100608557:-	65	13	GAGGAGGAGAAGGAAAGG	GAGGAGGAGAAGGAAAGGGGATCCCCACTGCCTGACTTCTAGCCCCCACATTTCTCCTTCCCCAA
296	Mature_291	bta-miR-11_19550	chr11	9473938	9473957	+	20	43	0	0	0	43	0	0	Precursor_296	chr11_19550|chr11:9473938-9473994:+	57	56	CTGCACCAAGTTAGTTAGGG	CTGCACCAAGTTAGTTAGGGAAAAAAAAAAAAAAATTACTGAACTGAACTGAAGCAG
297	Mature_292	bta-miR-11_23580	chr11	14183537	14183556	-	20	24	0	0	24	0	0	0	Precursor_297	chr11_23580|chr11:14183537-14183586:-	50	24	AGAAGCCAGGAGCAGAGAGG	TTTCTACCTCCTCACATCTGCTGGTAGAACAGAAGCCAGGAGCAGAGAGG
298	Mature_293	bta-miR-11_19278	chr11	14531915	14531933	+	19	54	54	0	0	0	0	0	Precursor_298	chr11_19278|chr11:14531915-14531970:+	56	54	TAGAAAGGAGTTTATATAC	TAGAAAGGAGTTTATATACTCATCTGGAGCACAGATTTGTGTAACTGTTGCCTAGG
299	Mature_294	bta-miR-11_19594	chr11	15869579	15869598	+	20	313	0	0	0	128	88	97	Precursor_299	chr11_19594|chr11:15869579-15869619:+	41	313	CTTGCTTTGTGTTTTCATTG	CTTGCTTTGTGTTTTCATTGTCCTGTCACACAAACCAAAGC
300	Mature_295	bta-miR-11_19645	chr11	20510206	20510224	+	19	510	0	0	0	180	160	170	Precursor_300	chr11_19645|chr11:20510148-20510224:+	77	510	CATGCCTTGAGGTGCAGCC	CTGTGCTTCCACTGCAGGGGGCAGAGGTTTGACCCCTGGTCAGAAAAGTAAGGTCCCCCATGCCTTGAGGTGCAGCC
301	Mature_296	bta-miR-11_19354	chr11	22615281	22615299	+	19	468	192	0	276	0	0	0	Precursor_301	chr11_19354|chr11:22615281-22615358:+	78	468	GGAAAGAAAGGAAGGAAAG	GGAAAGAAAGGAAGGAAAGAAAACTAAGCCATGAGATCAACATACAGGGTTTTTTCTTTCCAAGACATCTGTGTCCTC
302	Mature_297	bta-miR-11_20653	chr11	25358733	25358754	+	22	21	0	0	0	0	0	21	Precursor_302	chr11_20653|chr11:25358699-25358754:+	56	21	GAGCCAGGTCGCTAGAGTCCCC	GGACCCAAAGCATCCTGGTTTCCTTACATTTAAGGAGCCAGGTCGCTAGAGTCCCC
303	Mature_298	bta-miR-11_22665	chr11	37373088	37373109	+	22	9	0	0	9	0	0	0	Precursor_303	chr11_22665|chr11:37373056-37373109:+	54	9	GGCGACAAGGCCGACGACATCC	AAGACGCGGCCCGCCTCCAGGCAGCCTACGCGGGCGACAAGGCCGACGACATCC
304	Mature_299	bta-miR-11_19640	chr11	43027091	43027110	-	20	17	0	17	0	0	0	0	Precursor_304	chr11_19640|chr11:43027091-43027176:-	86	17	GCAGGCCTTGCAGTGTGGCC	GCACTCTGCACGTCCAATGCAGGGGGTCTGGCCGAGTTTGATCCCTGGTCAGGGAGCTAAAATCCCGCAGGCCTTGCAGTGTGGCC
305	Mature_300	bta-miR-11_22763	chr11	46000317	46000334	+	18	251	0	0	251	0	0	0	Precursor_305	chr11_22763|chr11:46000253-46000334:+	82	251	GGAAGACAGAGGTACAGG	TGACAACTTCCTGTCTTGCTCAGGGAGCTGGAGAAGGCGGCTGTCATCTCCCTGAGCTTGCTGGGGAAGACAGAGGTACAGG
306	Mature_301	bta-miR-11_19541	chr11	46209262	46209279	+	18	8	8	0	0	0	0	0	Precursor_306	chr11_19541|chr11:46209198-46209279:+	82	8	GGCGGCCCGGGCGCGCGG	GGCGCCCGGGCTGGTCCCTAGTTGAGCCGCCGCCCGACCGGCTGGTCCCTGCGGCGGCTGCCGGGGCGGCCCGGGCGCGCGG
307	Mature_302	bta-miR-11_22766	chr11	46261573	46261594	+	22	22	0	0	22	0	0	0	Precursor_307	chr11_22766|chr11:46261573-46261653:+	81	22	TCTGAGAGGGAGGAGTAGTGGG	TCTGAGAGGGAGGAGTAGTGGGAAAAAGTGCAGAGGAAGAGAACTGCAGAAACTCCCTCAATAGCTTGCTAACTGCAGACA
308	Mature_303	bta-miR-11_18974	chr11	64676191	64676212	+	22	21	0	21	0	0	0	0	Precursor_308	chr11_18974|chr11:64676191-64676273:+	83	21	ACAGGGTATTTACAGCTCAAAG	ACAGGGTATTTACAGCTCAAAGAAAAAAAAAAAAAAAGAATGAAAGCAGATTGCTTCCAGAAGCTGTGCATCTGCTGCCTGGTA
309	Mature_304	bta-miR-11_19720	chr11	67083295	67083312	+	18	61	61	0	0	0	0	0	Precursor_309	chr11_19720|chr11:67083295-67083342:+	48	61	GAAAGAGAGGGAGAAATC	GAAAGAGAGGGAGAAATCAAGGGGCAAAGGATTCTCTCTAGCAGACCT
310	Mature_305	bta-miR-11_19037	chr11	72078448	72078470	+	23	22	0	22	0	0	0	0	Precursor_310	chr11_19037|chr11:72078401-72078470:+	70	22	TCCCCATGCCATGTGATGAGGCC	CACTCACAATGCAAGGGGCACTGGTTCCCTGTTCGGAGAACTAAGATTCCCCATGCCATGTGATGAGGCC
311	Mature_306	bta-miR-11_19165	chr11	86859658	86859675	+	18	375	0	134	0	0	0	241	Precursor_311	chr11_19165|chr11:86859604-86859675:+	72	375	GGGGCGCGGCGCGGCGGT	TGCCGCCCCGCCCCGCGCTTACCTCGCGAGCCGAGGGCGGTACGAGGGGCGGTGGGGGCGCGGCGCGGCGGT
312	Mature_307	bta-miR-11_21221	chr11	87291532	87291549	-	18	16	0	0	0	16	0	0	Precursor_312	chr11_21221|chr11:87291472-87291549:-	78	16	CTGGGGCAAGATGAATGC	CTGGGGCAAGATGAATGCAACCTGGTGGTAATTCACACTAGAGTGTGAAGGACTGATGGCACCATATCTCCCCCAGAG
313	Mature_308	bta-miR-11_20069	chr11	99276654	99276672	-	19	85	0	39	0	0	0	46	Precursor_313	chr11_20069|chr11:99276603-99276672:-	70	88	GGGGTGGGGGGGCGGCGAG	GGGGTGGGGGGGCGGCGAGGGGGGAAGTCCCGATTCCTAGCCACATTCGCTCGCCCCACCTCCACCCCTG
314	Mature_309	bta-miR-11_24439	chr11	99981013	99981032	-	20	12	0	0	12	0	0	0	Precursor_314	chr11_24439|chr11:99980988-99981032:-	45	12	AGCCGGGGAGGGAGAGAGAA	AGCCGGGGAGGGAGAGAGAAATGAGTTTTCCTGCCGCCCGGTGGC
315	Mature_310	bta-miR-11_20083	chr11	101092539	101092557	-	19	82	0	82	0	0	0	0	Precursor_315	chr11_20083|chr11:101092487-101092557:-	71	82	GAAGGGGAGGGAAAGGGAG	GAAGGGGAGGGAAAGGGAGGCTTCACCTCCAGGAAAAACATCCCAGGGAAGCTTCCCACCTTCTTCTTGGT
316	Mature_311	bta-miR-11_20995	chr11	102765173	102765190	-	18	19	19	0	0	0	0	0	Precursor_316	chr11_20995|chr11:102765136-102765190:-	55	19	AGGAAGGAAAGGAAGGAC	AGGAAGGAAAGGAAGGACAAAATCCACCAGACCCAGGAGGCCTCTTCCTTCCACA
317	Mature_312	bta-miR-11_20451	chr11	103124971	103124988	+	18	377	0	0	0	117	105	155	Precursor_317	chr11_20451|chr11:103124943-103124988:+	46	377	GGGGGCCGGGGGTGGGGC	CCCGCCCCCTCCTCCCGCGGCGCTCCACGGGGGCCGGGGGTGGGGC
318	Mature_313	bta-miR-11_20106	chr11	104130050	104130071	-	22	16	0	16	0	0	0	0	Precursor_318	chr11_20106|chr11:104130050-104130099:-	50	16	GGGCAGAGAGGAAGCGGCTATT	TGGCCGCCCTGCTGCGCCAGTGCCGTTGGGGCAGAGAGGAAGCGGCTATT
319	Mature_314	bta-miR-11_21040	chr11	106373016	106373033	-	18	7	7	0	0	0	0	0	Precursor_319	chr11_21040|chr11:106373016-106373080:-	65	7	GGGAGGGCAGGTGAGGAG	CCCGTCTGGTCTCCAGACAGCTTGGCCTATGTGACCACATGGGGGAGGGGAGGGCAGGTGAGGAG
320	Mature_315	bta-miR-12_25356	chr12	14303960	14303978	-	19	12	0	0	12	0	0	0	Precursor_320	chr12_25356|chr12:14303960-14304019:-	60	12	AGGGACAGAGATCCCCTCC	AGGGGAGATGGTGTTCTCAGTGAAGCGGGCAGCTTCCACCGAGGGACAGAGATCCCCTCC
321	Mature_316	bta-miR-12_20862	chr12	15475162	15475181	-	20	12	0	12	0	0	0	0	Precursor_321	chr12_20862|chr12:15475162-15475205:-	44	12	GGAGGCGGAGCTGAGCCTGG	GGGCTCGGGTTTTTCTAGAAAACTGGAGGCGGAGCTGAGCCTGG
322	Mature_317	bta-miR-12_24688	chr12	19194789	19194810	+	22	33	0	0	33	0	0	0	Precursor_322	chr12_24688|chr12:19194789-19194844:+	56	33	GAGGACTCGGAAGGCAGAAAGC	GAGGACTCGGAAGGCAGAAAGCAAGAAAAAGCAAGACTGCTGTCTAAGGAGCTCTG
323	Mature_318	bta-miR-12_22588	chr12	19473118	19473136	+	19	45	0	0	0	0	0	45	Precursor_323	chr12_22588|chr12:19473118-19473183:+	66	46	CTCTTTTGTATATATATAT	CTCTTTTGTATATATATATATAAAATATACATACATTATATAATATATATAATATACATATTTTAG
324	Mature_319	bta-miR-12_24693	chr12	20386010	20386028	+	19	9	0	0	9	0	0	0	Precursor_324	chr12_24693|chr12:20385962-20386028:+	67	9	CCTCTTCCTCCCCAAACCC	GTCAAGGGGAGGGAGAAGAGGGACAGAAGCAAGTAGACCATCCCTCCCCCTCTTCCTCCCCAAACCC
325	Mature_320	bta-miR-12_21167	chr12	20486873	20486892	+	20	2453	696	0	1757	0	0	0	Precursor_325	chr12_21167|chr12:20486818-20486892:+	75	2453	GAAGGAGAAAGGAAAGGAGG	GCCCCTCCTTTCTCTCCAGGCCCTGGCTCACTGTATGCTCCAAGCTGGGGAGAAGGAAGGAGAAAGGAAAGGAGG
326	Mature_321	bta-miR-12_20960	chr12	24180627	24180649	-	23	11	0	11	0	0	0	0	Precursor_326	chr12_20960|chr12:24180627-24180699:-	73	11	AGGGATAGGAAATGATAGAAAGT	TTTTTATCCCTGTTGTCTGCCACAGGGCTGAATCTCTAGCAGTGTGGAGGAGGGATAGGAAATGATAGAAAGT
327	Mature_322	bta-miR-12_21873	chr12	25340437	25340457	-	21	18	18	0	0	0	0	0	Precursor_327	chr12_21873|chr12:25340373-25340457:-	85	18	GAGGAGGGCGGCGGCGGCGGC	GAGGAGGGCGGCGGCGGCGGCGGGCGCGCTCCCTTTTCTTGTGGCTCACTCGCTCGCCCGCCCCGCTCCTCCTTCTCCTCCTCCG
328	Mature_323	bta-miR-12_24765	chr12	26987622	26987643	+	22	22	0	0	22	0	0	0	Precursor_328	chr12_24765|chr12:26987577-26987643:+	67	22	TACGGAAGGTGTGGTATTGAGG	TCATTACCGCTTTTTTCTCTAGGCAGTCAGAGACCTCTAACTCCTTACGGAAGGTGTGGTATTGAGG
329	Mature_324	bta-miR-12_23437	chr12	28088356	28088373	-	18	142	0	0	0	0	0	142	Precursor_329	chr12_23437|chr12:28088313-28088373:-	61	144	GGGGCGCGGCGCGGCGCC	GGGGCGCGGCGCGGCGCCCGTGCGCCCGGCTCCCGCGCAGCATGCCCGCCCGCGCCCCGCC
330	Mature_325	bta-miR-12_25535	chr12	29027120	29027138	-	19	10	0	0	10	0	0	0	Precursor_330	chr12_25535|chr12:29027066-29027138:-	73	10	GGAGTGAGGGTGAGGAGAG	GGAGTGAGGGTGAGGAGAGGGGTGGGATGCATTTCTCCTGCTTGAACCTCCTTCACCAAGATCTGGTGGCCCA
331	Mature_326	bta-miR-12_21294	chr12	34693264	34693284	+	21	8	8	0	0	0	0	0	Precursor_331	chr12_21294|chr12:34693264-34693328:+	65	8	AGGAAGGGGAGAGAAAGAGGG	AGGAAGGGGAGAGAAAGAGGGAGGAGGAATACTTTGACTCTCAGCCCCGGGTCTTTTTTTCCTTG
332	Mature_327	bta-miR-12_20406	chr12	38294897	38294914	+	18	195	0	195	0	0	0	0	Precursor_332	chr12_20406|chr12:38294897-38294968:+	72	195	AGGAGGAGAAGGGGGAAT	AGGAGGAGAAGGGGGAATCAAAAGGGGCGAGACCAATCTAGCCAGTAATTAGTTTCCTAAGTGTTCTCCACA
333	Mature_328	bta-miR-12_20495	chr12	52120926	52120945	+	20	31	0	31	0	0	0	0	Precursor_333	chr12_20495|chr12:52120861-52120945:+	85	31	AAGCAGTGGGAGGAGAATGT	ATGTGCTCACCTACTGCCCAGAAAGTTGACAAGACAGAGGTCACCAGTAAGCCAAAGCTTTTACAAAGCAGTGGGAGGAGAATGT
334	Mature_329	bta-miR-12_20604	chr12	67359923	67359942	+	20	11	0	11	0	0	0	0	Precursor_334	chr12_20604|chr12:67359923-67359978:+	56	15	TCTTCTCTTGCTCTCATGAC	TCTTCTCTTGCTCTCATGACAAAAAAAAAAAAAAAACAGAGAGAGAGAGGAGAGAG
335	Mature_330	bta-miR-12_25075	chr12	70095843	70095862	+	20	15	0	0	15	0	0	0	Precursor_335	chr12_25075|chr12:70095821-70095862:+	42	15	AAGTCAGATTCTGCTATTGG	AGTGGCAGTACCTGAAAGATGCAAGTCAGATTCTGCTATTGG
336	Mature_331	bta-miR-12_22097	chr12	83569176	83569196	+	21	662	0	0	0	662	0	0	Precursor_336	chr12_22097|chr12:83569176-83569266:+	91	739	GGACAGAGGAGCCTGGCGGGC	GGACAGAGGAGCCTGGCGGGCTACAGTCTACAGAGTTGCAATGAGTTGGACACAACTGAGTGACTTAGCATGCACACACATTCTCTCTCCC
337	Mature_332	bta-miR-12_22234	chr12	85192161	85192178	-	18	14	14	0	0	0	0	0	Precursor_337	chr12_22234|chr12:85192161-85192218:-	58	14	GAAAGCAGCAAGTGGGGG	TACCACTGGGCTACCAGGGAAGTCCCAAGGAAAATATCCTGAAAGCAGCAAGTGGGGG
338	Mature_333	bta-miR-13_21417	chr13	367460	367482	+	23	14	0	14	0	0	0	0	Precursor_338	chr13_21417|chr13:367435-367482:+	48	14	GCAGTGCCCTGTAAGCCCCCTCC	AGGGGGCTCGCCAGGGCGCCCAGCTGCAGTGCCCTGTAAGCCCCCTCC
339	Mature_334	bta-miR-13_26834	chr13	3838170	3838187	-	18	18	0	0	18	0	0	0	Precursor_339	chr13_26834|chr13:3838170-3838236:-	67	18	GGAAAAAAGCAGGAAAGC	TCTCAAAGCTTTGCTTCTTTTCTCTCATGAGTAAACAGCAGTGAAAAGGGGAAAAAAGCAGGAAAGC
340	Mature_335	bta-miR-13_22269	chr13	17399076	17399096	-	21	14	0	14	0	0	0	0	Precursor_340	chr13_22269|chr13:17399036-17399096:-	61	14	GGAGCTGATTTGAAAGCAGGG	GGAGCTGATTTGAAAGCAGGGTGAAGAAGAAAGTATGGCCTTGCCAAGAGAAGCTTTCCCA
341								0							Precursor_341	chr13_22270|chr13:17399076-17399126:-	51	14		CTGCGCGAGAGCTTGACAGTCAAACTGAAGGGAGCTGATTTGAAAGCAGGG
342	Mature_336	bta-miR-13_23037	chr13	22668801	22668822	+	22	26	0	0	0	26	0	0	Precursor_342	chr13_23037|chr13:22668752-22668822:+	71	26	CCTACAAGCCATGCAGTGTGGT	TCTCACTGCCATGGCCCTCAGGTTCAGTTTCCGGTCAGGAAACTAAGACCCTACAAGCCATGCAGTGTGGT
343	Mature_337	bta-miR-13_21619	chr13	26924865	26924889	+	25	13	0	13	0	0	0	0	Precursor_343	chr13_21619|chr13:26924817-26924889:+	73	13	AAGTCAGGGTTCAGAGTTGGCTTCC	AAGTGATAACTCCAGAGATGCTTCTTGAGTAATGAGTAGAAATTAGTCAAGTCAGGGTTCAGAGTTGGCTTCC
344	Mature_338	bta-miR-13_23089	chr13	27111006	27111025	-	20	738	174	0	564	0	0	0	Precursor_344	chr13_23089|chr13:27110980-27111025:-	46	738	AGGAGAAAGGAAGAGGGACG	AGGAGAAAGGAAGAGGGACGTCATCCCAGATCTCCTTCTTTCCTTT
345	Mature_339	bta-miR-13_22360	chr13	28076860	28076880	-	21	4747	0	3432	0	471	472	372	Precursor_345	chr13_22360|chr13:28076860-28076944:-	85	4762	GGATTTTTGGTGCAGGGAAAC	CCCCCTGCATTGGGAACTCAGAGTCTTAGCCACTGGACTACCAGGGAAGTTCCAAGATCATTGAGGATTTTTGGTGCAGGGAAAC
346	Mature_340	bta-miR-13_25512	chr13	28733262	28733280	-	19	22	0	0	0	0	22	0	Precursor_346	chr13_25512|chr13:28733262-28733300:-	39	26	GGGTGTGGGTTCAGCCCCT	CGCCACACTTCCACTGCAGGGGGTGTGGGTTCAGCCCCT
347	Mature_341	bta-miR-13_22444	chr13	29317669	29317686	+	18	311	311	0	0	0	0	0	Precursor_347	chr13_22444|chr13:29317669-29317724:+	56	311	GAAGAGAAGAGAAAAGCC	GAAGAGAAGAGAAAAGCCATCTCCTGTTACGGTGATGATTTTATTTAGCTTTTTTG
348	Mature_342	bta-miR-13_21662	chr13	32646892	32646911	+	20	12	0	12	0	0	0	0	Precursor_348	chr13_21662|chr13:32646892-32646969:+	78	12	CTACCATGTGGGGACACAGC	CTACCATGTGGGGACACAGCAAAAAGGCAGTCTCTGAACCAGGACTGGACCCGTGCTGGCACCCTGATCATGGACTTC
349	Mature_343	bta-miR-13_22476	chr13	33302080	33302099	+	20	36	36	0	0	0	0	0	Precursor_349	chr13_22476|chr13:33302050-33302099:+	50	72	CCACTCCAGTACTCTTGCCT	GGAGGAGGAAATGGAAATGGAAATGGAAATCCACTCCAGTACTCTTGCCT
350	Mature_344	bta-miR-13_27210	chr13	41853104	41853121	-	18	88	0	0	88	0	0	0	Precursor_350	chr13_27210|chr13:41853104-41853159:-	56	88	GGAGGAGAGGAAAGGGGG	CTCGCCCCTCTTCTTTCACTGCTTCTGCACTTGGCTATGGAGGAGAGGAAAGGGGG
351	Mature_345	bta-miR-13_27284	chr13	47719002	47719019	-	18	26	0	0	26	0	0	0	Precursor_351	chr13_27284|chr13:47719002-47719068:-	67	26	GGAAAGAAGAGGAACAGG	TGTGCCTTTTTAGTTCCAGCTCCATTTTGAAGACTTGCAGATCATGAATGGAAAGAAGAGGAACAGG
352	Mature_346	bta-miR-13_25697	chr13	47859609	47859629	-	21	23	0	0	0	0	23	0	Precursor_352	chr13_25697|chr13:47859574-47859629:-	56	23	CTGCCTTTGTTAAGTTACCCC	CTGCCTTTGTTAAGTTACCCCAAAAAGCAAAATTTGAGAGAAAGACATGGGTGCTG
353	Mature_347	bta-miR-13_21793	chr13	48122027	48122044	+	18	16	0	16	0	0	0	0	Precursor_353	chr13_21793|chr13:48122000-48122044:+	45	16	CCTGCATGCGGTGCAGCC	TTCCATCCTTGGTCAGGAAACTAAGATCCTGCATGCGGTGCAGCC
354	Mature_348	bta-miR-13_22608	chr13	53695385	53695402	+	18	31	31	0	0	0	0	0	Precursor_354	chr13_22608|chr13:53695385-53695471:+	87	31	AAAGAAGAAGAGAGGAAG	AAAGAAGAAGAGAGGAAGGAAAGAGGGAAGAGGTGGGTGGGGAAGAGCATTCCAAGCCCACAGGCCTGAGGGTCTCATTGCATTTCT
355	Mature_349	bta-miR-13_21841	chr13	54343833	54343850	+	18	121	0	121	0	0	0	0	Precursor_355	chr13_21841|chr13:54343793-54343850:+	58	121	GGGTGGGGGGGCGGCGGG	AGTGGTCCTTTCCCAGCTTCATCCTATCCAGAGGGGCAGTGGGTGGGGGGGCGGCGGG
356	Mature_350	bta-miR-13_22558	chr13	55190522	55190540	-	19	12	0	12	0	0	0	0	Precursor_356	chr13_22558|chr13:55190522-55190589:-	68	12	CCAGTTTCCTCATCTGATT	TCAGCGGGGGAGAGGTCAGGATGGAGGCGTGAGAACGTTCATCCCAAGTCCAGTTTCCTCATCTGATT
357	Mature_351	bta-miR-13_24953	chr13	59445979	59445998	+	20	15	0	0	0	0	15	0	Precursor_357	chr13_24953|chr13:59445915-59445998:+	84	15	CCAGCTTCTGAAAGTGACAC	GCCGCGGACAGGAGCCGCCGCCAGGAACACAACCAGGCCCACAGGCCACTGCGTTCAAAAACCACCAGCTTCTGAAAGTGACAC
358	Mature_352	bta-miR-13_22640	chr13	64800425	64800448	-	24	36	0	36	0	0	0	0	Precursor_358	chr13_22640|chr13:64800425-64800479:-	55	36	AAGGCTAATAAGCACATTCAAAAG	TTTGAATGTGCTTATTAGCCTTGTGTGTGTCAAGGCTAATAAGCACATTCAAAAG
359	Mature_353	bta-miR-13_21941	chr13	64800458	64800481	+	24	36	0	36	0	0	0	0	Precursor_359	chr13_21941|chr13:64800427-64800481:+	55	36	AAGGCTAATAAGCACATTCAAAAG	TTTGAATGTGCTTATTAGCCTTGACACACACAAGGCTAATAAGCACATTCAAAAG
360	Mature_354	bta-miR-13_23514	chr13	66909509	66909531	-	23	8	8	0	0	0	0	0	Precursor_360	chr13_23514|chr13:66909509-66909581:-	73	8	CGGCGCGGCGGAGGAGGCGGGAG	CCCGCAGAACCTTTCTGGCTCTGGGCGGTGCGCGAATTACGCGTCACTTCCGGCGCGGCGGAGGAGGCGGGAG
361	Mature_355	bta-miR-13_21968	chr13	67695439	67695462	+	24	12	0	12	0	0	0	0	Precursor_361	chr13_21968|chr13:67695439-67695491:+	53	12	AGGGGCTTGAAGACAGAAGAGAAG	AGGGGCTTGAAGACAGAAGAGAAGCCCCTTCTCCCTTACCTTCTAGTTCCATA
362	Mature_356	bta-miR-13_21979	chr13	68699367	68699390	+	24	13	0	13	0	0	0	0	Precursor_362	chr13_21979|chr13:68699302-68699390:+	89	13	TGGCCACACTAAATTGTTCTCAGG	TAGGAGAGAGCTCCTGGGTGGCTGACTTGGCTCCATGCTGATTTAGAAATTAGCTGGGCTTTTATTGGCCACACTAAATTGTTCTCAGG
363	Mature_357	bta-miR-13_24175	chr13	69563383	69563401	-	19	125	0	0	0	41	35	49	Precursor_363	chr13_24175|chr13:69563352-69563401:-	50	125	CCCCAAATCTCTTTCCAGC	CCCCAAATCTCTTTCCAGCTGATGTCCCATGTCTAGGGAGGTCTGGGTTT
364	Mature_358	bta-miR-13_26709	chr13	73010849	73010867	+	19	8	0	0	8	0	0	0	Precursor_364	chr13_26709|chr13:73010794-73010867:+	74	8	GGAGAAGAAAGGGGAGAAA	TCATTGCTTTCTTTTCCTTCCTTCAGAAATGCCTTGTTATGCGTTGCGGGATAGAGGAGAAGAAAGGGGAGAAA
365	Mature_359	bta-miR-13_26755	chr13	76839787	76839805	+	19	14	0	0	14	0	0	0	Precursor_365	chr13_26755|chr13:76839756-76839805:+	50	14	GAAAGGAGGGAAGAGAGAG	TTCACAGAGCTCAGTGTTTTCCAAAGTGTGGGAAAGGAGGGAAGAGAGAG
366	Mature_360	bta-miR-13_22757	chr13	76959584	76959602	-	19	11	0	11	0	0	0	0	Precursor_366	chr13_22757|chr13:76959515-76959602:-	88	12	GGGTCGTTGGTCAGGGACG	GGGTCGTTGGTCAGGGACGCTGAGAGCACAAGCCTAGGTCAGAGGGAAACAGCGGAAGGCCAGTGTCCAGACTGGTGGCCCAGTGGTC
367	Mature_361	bta-miR-13_25150	chr13	77617989	77618007	+	19	18	0	0	0	0	18	0	Precursor_367	chr13_25150|chr13:77617989-77618058:+	70	18	CATCACAGTAAATGAGGGG	CATCACAGTAAATGAGGGGTCGGGAAAGGCCTCCCTGAGGAGGTGACCCTTGAGACAAGACTTGAATGAA
368	Mature_362	bta-miR-13_22770	chr13	77732442	77732462	-	21	34	0	34	0	0	0	0	Precursor_368	chr13_22770|chr13:77732442-77732519:-	78	34	TATGTGCCCAGAGGTGTGGCC	CCACACTTCCTCGCAGGGAGCACAGTTCCCTGATCCCTGGTCAGGGAACTAAAATCCTATGTGCCCAGAGGTGTGGCC
369	Mature_363	bta-miR-13_22861	chr13	78094863	78094880	+	18	10	10	0	0	0	0	0	Precursor_369	chr13_22861|chr13:78094836-78094880:+	45	10	GGAGAAACGGGGGCACAG	GTTGCCCCAGCCCTTGCCCCTGCAGGTGGAGAAACGGGGGCACAG
370	Mature_364	bta-miR-13_22063	chr13	78207718	78207738	+	21	13	0	13	0	0	0	0	Precursor_370	chr13_22063|chr13:78207665-78207738:+	74	15	GGCTCGGGGGAGGGGCAGAGG	GCCCCCCAGCCCCGCCCCCAGGGCCTGTGCTTGGAGATCGGGGTATCAGGCCTGGCTCGGGGGAGGGGCAGAGG
371	Mature_365	bta-miR-13_22066	chr13	78592618	78592640	+	23	13	0	13	0	0	0	0	Precursor_371	chr13_22066|chr13:78592618-78592672:+	55	13	AGAGCCCCGTTAAATGCCCCCTG	AGAGCCCCGTTAAATGCCCCCTGCAGCCTGGAGAGGTAAAGGAAGAGGGTCCTGG
372	Mature_366	bta-miR-13_25201	chr13	82009270	82009291	+	22	31	0	0	0	0	31	0	Precursor_372	chr13_25201|chr13:82009201-82009291:+	91	31	CTGGTGTCATCACAAGGTCCCC	GGACTTTGCAGTTGTGATGCAGTTAAAGCTCCTGAGATGGAGAGATTGTGCAGGATTATTCCAGTGGGCCTGGTGTCATCACAAGGTCCCC
373	Mature_367	bta-miR-13_22110	chr13	82117938	82117956	+	19	10	0	10	0	0	0	0	Precursor_373	chr13_22110|chr13:82117938-82118009:+	72	10	GGGCTCCGGAGCGCAGGCT	GGGCTCCGGAGCGCAGGCTCAGCCGGTGTGGCGCGCGCGCTTGGTTGCTCCTTGGGGTCTCCTTGGACCCGG

374	Mature_368	bta-miR-14_24327	chr14	2031835	2031855	+	21	62	0	0	0	62	0	0	Precursor_374	chr14_24327|chr14:2031783-2031855:+	73	62	CTTCCACTGAGTCACAGCCCC	GGACTGGGGTCATGGAGGTCAGCTCACCCCATCCATGGGCTGTGCAGGTAAGCTTCCACTGAGTCACAGCCCC
375	Mature_369	bta-miR-14_23512	chr14	2398899	2398917	-	19	10	0	10	0	0	0	0	Precursor_375	chr14_23512|chr14:2398899-2398955:-	57	10	CAGGTGGCTCTGGGAGAGC	TGTCCCTGAAGCTGGCTTGTCCTCAGCTCCCTGCAGCACAGGTGGCTCTGGGAGAGC
376	Mature_370	bta-miR-14_22834	chr14	2865286	2865303	+	18	231	0	231	0	0	0	0	Precursor_376	chr14_22834|chr14:2865286-2865324:+	39	231	GGGGGGGGCCGGGCCGCC	GGGGGGGGCCGGGCCGCCTCCCACCTTCCCTCCTCCTGA
377	Mature_371	bta-miR-14_28508	chr14	5836979	5836996	-	18	16	0	0	16	0	0	0	Precursor_377	chr14_28508|chr14:5836979-5837039:-	61	16	GAGGAAAGAGAGAGGAGG	TTTTCCTTTCTTTCCTTCCGATTTTTCCCCATTTAGAAAAACTGAGGAAAGAGAGAGGAGG
378	Mature_372	bta-miR-14_27835	chr14	9054126	9054144	+	19	27	0	0	27	0	0	0	Precursor_378	chr14_27835|chr14:9054076-9054144:+	69	27	AAGAGAGGAGAGAAAAGGG	CATTTTTCTCCTGAAGGCTTTTCTCTTCCCAGTGTCCAGCCTCAGAGAAGAAGAGAGGAGAGAAAAGGG
379	Mature_373	bta-miR-14_23566	chr14	9638159	9638183	-	25	11	0	11	0	0	0	0	Precursor_379	chr14_23566|chr14:9638159-9638244:-	86	11	CTAGAGTCCCACAAGCCATGAGGCC	CCAGTGGTTAGGACTCTGTACTCACTTGCCAAGAACCCAGGTTCAATCCCTGGTCAGGAAACTAGAGTCCCACAAGCCATGAGGCC
380	Mature_374	bta-miR-14_26995	chr14	12334157	12334174	-	18	9	0	0	0	0	9	0	Precursor_380	chr14_26995|chr14:12334122-12334174:-	53	9	TTCCAGGCCTTCTTTTTG	TTCCAGGCCTTCTTTTTGGCTGAGCCCAGTCAGAAACCAGGAAGCAAGGGAGA
381	Mature_375	bta-miR-14_24507	chr14	16597463	16597480	-	18	70	15	0	55	0	0	0	Precursor_381	chr14_24507|chr14:16597399-16597480:-	82	70	AAGAGAAGATGAAGGAGG	AAGAGAAGATGAAGGAGGAGGGAGGCCTTCTCTTATTTTCCATAAGGACAATCAGGCCTCTCTCTGTTCATGTGTATTTGCC
382	Mature_376	bta-miR-14_26445	chr14	16950101	16950118	-	18	60	0	0	0	0	0	60	Precursor_382	chr14_26445|chr14:16950079-16950118:-	40	60	GGAGGAAGGTGGGGAAAC	GGAGGAAGGTGGGGAAACCAAGACTTTCCCTGGACCCCTC
383	Mature_377	bta-miR-14_28652	chr14	19309699	19309720	-	22	19	0	0	19	0	0	0	Precursor_383	chr14_28652|chr14:19309699-19309789:-	91	19	GGAAGACAGGTCACAGAGAGAC	GGCTCTGAGAGCGTCTTGCTCATTGATTCAGAATCCTCTCACAAATACCACATGGATTTTGGATCAGATGGAAGACAGGTCACAGAGAGAC
384	Mature_378	bta-miR-14_26562	chr14	29066731	29066753	-	23	32	0	0	0	0	0	32	Precursor_384	chr14_26562|chr14:29066731-29066788:-	58	32	TCCTGACCTTATTTCAATTCTTC	AAAGTTGGTCCAGGTACAGGATGGTTCTTCAGCCATCCTGACCTTATTTCAATTCTTC
385	Mature_379	bta-miR-14_23958	chr14	33050603	33050622	+	20	14	14	0	0	0	0	0	Precursor_385	chr14_23958|chr14:33050603-33050659:+	57	14	GAGAAAAGAAAGGAAAGAAG	GAGAAAAGAAAGGAAAGAAGCAGAAAAGATCCTCTTCTTAATCCCACATTTTCTCCC
386	Mature_380	bta-miR-14_25321	chr14	35419478	35419495	-	18	7973	0	0	0	4099	3874	0	Precursor_386	chr14_25321|chr14:35419409-35419495:-	87	7991	GGATTTTTGGAGCAGGAG	GGATTTTTGGAGCAGGAGTTATTCAAGGAAGGACCAGTGATGATCAGTTCATTTGAACTTCCAACAATCCTGCGAGAAATAAATGGG
387	Mature_381	bta-miR-14_23817	chr14	44236077	44236094	-	18	159	0	47	112	0	0	0	Precursor_387	chr14_23817|chr14:44236038-44236094:-	57	159	GGAGAAAGGGGAGAAAGG	GGAGAAAGGGGAGAAAGGGGAAGCTGTATTTTTTTTCTTCTTTTCAGTGTCTCTCA
388	Mature_382	bta-miR-14_25975	chr14	45930811	45930829	+	19	59	0	0	0	0	0	59	Precursor_388	chr14_25975|chr14:45930776-45930829:+	54	59	GGAGGAAGGTGGGGAAACT	GTTCCCTACTCCTGCTTCTCTTTGGATTTCTGAAAGGAGGAAGGTGGGGAAACT
389	Mature_383	bta-miR-14_23850	chr14	47674782	47674800	-	19	12	0	12	0	0	0	0	Precursor_389	chr14_23850|chr14:47674782-47674829:-	48	12	TGCATGATTCCGTCAGGGG	CCTGACTGGAGAATTATGACATTCAGAGATGCATGATTCCGTCAGGGG
390	Mature_384	bta-miR-14_23192	chr14	47857323	47857342	+	20	28	0	28	0	0	0	0	Precursor_390	chr14_23192|chr14:47857323-47857399:+	77	28	CCTGTTGTCGTTACAGGATG	CCTGTTGTCGTTACAGGATGAATGTCCCAAGTCCAGCTCCCTTTGCTTTAAGACATAGGTATGTATATACATAGGTT
391	Mature_385	bta-miR-14_23918	chr14	54933614	54933632	-	19	10	0	10	0	0	0	0	Precursor_391	chr14_23918|chr14:54933614-54933688:-	75	10	GAAAACGAACTTTTTGGCC	CCAAAAAGCTCGTTCATTCAGACTTTTCTTCCCCCATACTATCTTATGAAAAAACTGAAAACGAACTTTTTGGCC
392	Mature_386	bta-miR-14_28217	chr14	56949419	56949442	+	24	15	0	0	15	0	0	0	Precursor_392	chr14_28217|chr14:56949389-56949442:+	54	15	GGGTTTGACAGGCACTGGATCAGG	CGATCAAACACCTGACTCCAAGCCCCTCCTGGGTTTGACAGGCACTGGATCAGG
393	Mature_387	bta-miR-14_23354	chr14	64343671	64343689	+	19	85	0	85	0	0	0	0	Precursor_393	chr14_23354|chr14:64343671-64343716:+	46	85	GGGGGGGGGGCCGGGGCCC	GGGGGGGGGGCCGGGGCCCCCAGGGGGGGGTGCCCCCCACAGTCCA
394	Mature_388	bta-miR-14_24224	chr14	66964300	66964323	+	24	42	42	0	0	0	0	0	Precursor_394	chr14_24224|chr14:66964300-66964388:+	89	42	AGGAGGAAAGAGAGGAGGAAGATG	AGGAGGAAAGAGAGGAGGAAGATGTTGCTCAGGAGCAGCTGCAAGCTCTCCTGAGCTAAGTTTAGCCCCTTCCTGCATCTTTCCCTGGG
395	Mature_389	bta-miR-14_24249	chr14	69105914	69105931	+	18	8	8	0	0	0	0	0	Precursor_395	chr14_24249|chr14:69105892-69105931:+	40	8	AGGGAGGGGGACACAGCC	GTGGGTTGCCTAGCACCATGGGAGGGAGGGGGACACAGCC
396	Mature_390	bta-miR-14_25661	chr14	69446597	69446615	-	19	46	0	0	0	46	0	0	Precursor_396	chr14_25661|chr14:69446554-69446615:-	62	49	GGGCCCGGGTGGAGCCGCC	GGGCCCGGGTGGAGCCGCCGGGGGAGGCCGGGAGACCCGGCGCGGCGGCGCGGCCCGGGGCC
397	Mature_391	bta-miR-14_23427	chr14	73584957	73584975	+	19	22	0	22	0	0	0	0	Precursor_397	chr14_23427|chr14:73584897-73584975:+	79	22	TACCTTAATCGGGCTTGTC	TAAGTTGGGACTGAGATGGTCGGGAGGGAAGACTGCCCTGTCGTTTTCCCTTGAATTGGCTACCTTAATCGGGCTTGTC
398	Mature_392	bta-miR-15_30192	chr15	24693131	24693148	-	18	12	0	0	12	0	0	0	Precursor_398	chr15_30192|chr15:24693131-24693194:-	64	12	GGCAGGATGCAGGGCAGC	TGCCTCCGCTCCTGTCTCCCCGTCCAGACGGCTGACCCTTAGGGCTGGCAGGATGCAGGGCAGC
399	Mature_393	bta-miR-15_26674	chr15	26255133	26255155	-	23	74	0	0	0	34	40	0	Precursor_399	chr15_26674|chr15:26255133-26255206:-	74	74	CTGCCTAGGAAGTCAGGACAGTG	ATGCCTGCTTCTCTGGGTCATGTGAGGATTAAATGTATTAACATTTGTACACTGCCTAGGAAGTCAGGACAGTG
400	Mature_394	bta-miR-15_25810	chr15	28857473	28857495	-	23	51	51	0	0	0	0	0	Precursor_400	chr15_25810|chr15:28857473-28857549:-	77	52	GGGCCCTGGGAAGGGAAGAAAGG	CAGCTACTGCTTCCAGGGCTGGAGTAGGAGAAGAAGGAAGAGATTCCAGAATCTGGGCCCTGGGAAGGGAAGAAAGG
401	Mature_395	bta-miR-15_26701	chr15	29310249	29310273	-	25	83	0	0	0	83	0	0	Precursor_401	chr15_26701|chr15:29310190-29310273:-	84	83	CCTCCTCTGGCCTCCAGTTCCCGGC	CCTCCTCTGGCCTCCAGTTCCCGGCTTCGTCCCTTCTGGCTCTAAGCAAGGCACGAGTGGAAGGGGGAGGGCTGGATGGGGGAG
402	Mature_396	bta-miR-15_29415	chr15	29968972	29968990	+	19	16	0	0	16	0	0	0	Precursor_402	chr15_29415|chr15:29968941-29968990:+	50	16	CAGCTGAGAGAGAAGATGG	CTCTTTTCCTCAGTGAATTAGGCAGCAAGGTCAGCTGAGAGAGAAGATGG
403	Mature_397	bta-miR-15_25840	chr15	30559835	30559852	-	18	17	17	0	0	0	0	0	Precursor_403	chr15_25840|chr15:30559835-30559902:-	68	17	GAGAAAAGAGAGAAGGAG	CCTTCTTCTGAATTTTCTATGCTCTGCAATGCCTAATGCAGAAGGGGTGGGAGAAAAGAGAGAAGGAG
404	Mature_398	bta-miR-15_28696	chr15	31293331	31293348	-	18	144	0	0	0	0	144	0	Precursor_404	chr15_28696|chr15:31293331-31293405:-	75	144	GGGGAGGCAGCAGTGGGG	CCTCCTCCATGCCCCACCCCCAGCCCCAACTCTGAACTTTCCAGCTTGCCCCAGCGAGGGGAGGCAGCAGTGGGG
405	Mature_399	bta-miR-15_25172	chr15	33368898	33368921	+	24	7	7	0	0	0	0	0	Precursor_405	chr15_25172|chr15:33368898-33368971:+	74	10	CTGCCCAAGCAGAGGGGGTTCACC	CTGCCCAAGCAGAGGGGGTTCACCAAGGAAAAAGGAAAGGGTTCAGGGGCTGGGCCCCTTTTGCTTGGGCAGCA
406	Mature_400	bta-miR-15_28158	chr15	34284983	34285006	-	24	117	0	0	0	0	0	117	Precursor_406	chr15_28158|chr15:34284983-34285053:-	71	117	CCCTGCATGTTATACTATGAGGCC	CTTCCATTGGCAGGGAAAGGGTTCAGTCTCTGGTTGAGAAATTAGGACCCTGCATGTTATACTATGAGGCC
407	Mature_401	bta-miR-15_25109	chr15	34690279	34690298	-	20	14	0	14	0	0	0	0	Precursor_407	chr15_25109|chr15:34690220-34690298:-	79	14	ACAAGCTAGATGGCAGAGTC	ACAAGCTAGATGGCAGAGTCAAAAAAAAAAAAAAATACCAGGGTTGGTTTCTGGTGAGGCCTGTCTTCCTGGCTTGCAG
408	Mature_402	bta-miR-15_28754	chr15	36561253	36561271	-	19	24	0	0	0	0	24	0	Precursor_408	chr15_28754|chr15:36561253-36561331:-	79	24	GAGGAGGAGAAGACGAAGG	TTCCTACCCTCCTGGTACCTGGAATATCAGGCTAGATGCTTAGTTTCTCTGCAGGGATGGAGGAGGAGAAGACGAAGG
409	Mature_403	bta-miR-15_27395	chr15	36829157	36829181	+	25	50	0	0	0	0	0	50	Precursor_409	chr15_27395|chr15:36829131-36829181:+	51	50	GGGGGTTAGAAGCTCTCAGAATAGC	TATCAGGGCTTTGTCCTTCTTATGGAGGGGGTTAGAAGCTCTCAGAATAGC
410	Mature_404	bta-miR-15_24384	chr15	37194515	37194532	+	18	50	0	34	16	0	0	0	Precursor_410	chr15_24384|chr15:37194515-37194560:+	46	50	CCCCTGTGGTCTAGTGGT	CCCCTGTGGTCTAGTGGTAAGACTCCACCCTTCCAATGCAGGGGGC
411	Mature_405	bta-miR-15_27417	chr15	38345204	38345227	+	24	109	0	0	0	0	0	109	Precursor_411	chr15_27417|chr15:38345162-38345227:+	66	109	CAGGCAGTTAGCAGGTTTTTATTT	ATAAGAGATGTTGGGAACTGTGGCAATGGTTTCCTAGGCACACAGGCAGTTAGCAGGTTTTTATTT
412	Mature_406	bta-miR-15_26810	chr15	40500898	40500922	-	25	324	0	0	0	103	111	110	Precursor_412	chr15_26810|chr15:40500898-40500960:-	63	324	CTCATGTGTCTGTAATAAAGCCTCC	AGGCTAGAGATGGAGCTCATTGACCAGTGATTTAATCACTCATGTGTCTGTAATAAAGCCTCC
413	Mature_407	bta-miR-15_29731	chr15	55353227	55353250	+	24	18	0	0	18	0	0	0	Precursor_413	chr15_29731|chr15:55353186-55353250:+	65	18	GAATAAATTAGGCATTGCCAAAGG	TTTGGCAATGCCTGATTTATTCATTCATTAACTCATTAAATGAATAAATTAGGCATTGCCAAAGG
414	Mature_408	bta-miR-15_25272	chr15	56071494	56071511	-	18	13	0	13	0	0	0	0	Precursor_414	chr15_25272|chr15:56071494-56071576:-	83	13	CAGGCTGTGTGGCCTGGC	TAGGAATCCACACTCTCACTGTTGAGGGCCCAGGTTCAACCCCTGGTGGGGGAACTAAGATTCCACAGGCTGTGTGGCCTGGC
415	Mature_409	bta-miR-15_24589	chr15	61450663	61450681	+	19	11	0	11	0	0	0	0	Precursor_415	chr15_24589|chr15:61450629-61450681:+	53	11	CTGAGGGCATGTCTGCTGG	AGCAGTCATGTGACTCATTTCTGGCCAATAGACTCTGAGGGCATGTCTGCTGG
416	Mature_410	bta-miR-15_24624	chr15	64771332	64771356	+	25	17	0	17	0	0	0	0	Precursor_416	chr15_24624|chr15:64771332-64771412:+	81	17	TCTGGACCAAGTCCTCTTTCTGCCC	TCTGGACCAAGTCCTCTTTCTGCCCAGGAAAAAAAAAAAAAACATATACTTTTCAAGGGCACAGTGTAGACTAGTTAAGAGT
417	Mature_411	bta-miR-15_24709	chr15	74643377	74643396	+	20	10	0	10	0	0	0	0	Precursor_417	chr15_24709|chr15:74643351-74643396:+	46	14	TGAGCTTGCACCTCCGAGCA	GGACTGGGGCGCAAGCGGGCGACTCTTGAGCTTGCACCTCCGAGCA
418								0							Precursor_418	chr15_24710|chr15:74643377-74643435:+	59	10		TGAGCTTGCACCTCCGAGCAAAAAAAAAAAAAAAAAAGAGCAGAGGCAGCAGGTGAGAA
419	Mature_412	bta-miR-15_29911	chr15	76147050	76147069	+	20	14	0	0	14	0	0	0	Precursor_419	chr15_29911|chr15:76147016-76147069:+	54	14	CGCGAACGTGGTCAGGGGAG	CCCCTGCCAGGACCACCTCGCTGCTACCCACCATCGCGAACGTGGTCAGGGGAG
420	Mature_413	bta-miR-15_29919	chr15	77137843	77137860	+	18	17	0	0	17	0	0	0	Precursor_420	chr15_29919|chr15:77137819-77137860:+	42	17	GGAGGAGGGGCGAGAGGG	CATCAGCATCTTCTCTGCAGGTGTGGAGGAGGGGCGAGAGGG
421	Mature_414	bta-miR-15_25618	chr15	80928318	80928337	+	20	11	11	0	0	0	0	0	Precursor_421	chr15_25618|chr15:80928318-80928399:+	82	11	AGGGGAGGAAGCACCAAAAG	AGGGGAGGAAGCACCAAAAGGGAGAGAAGAGAAGCAAAGGCGTATTGCTCCTTGTCGGCACTGGTGGTGCTGAGTCTCCTTG
422	Mature_415	bta-miR-16_30751	chr16	1446269	1446289	+	21	28	0	0	28	0	0	0	Precursor_422	chr16_30751|chr16:1446223-1446289:+	67	28	AGGAAGAGAAACGAGAGACCC	GTCTTAATCGCATCTGGACTCTCAGAGAAGAAAGAAGTAAGAAGTGAGGAAGAGAAACGAGAGACCC
423	Mature_416	bta-miR-16_30757	chr16	1581612	1581632	+	21	21	0	0	21	0	0	0	Precursor_423	chr16_30757|chr16:1581582-1581632:+	51	21	GGGCGGCAGCGGCTAAGAAAG	TTCTGCTGGTGTTGCTGCTGAGAAGACGGCGGGCGGCAGCGGCTAAGAAAG
424	Mature_417	bta-miR-16_25515	chr16	4365615	4365639	+	25	13	0	13	0	0	0	0	Precursor_424	chr16_25515|chr16:4365586-4365639:+	54	13	AGAGAAGCTGCAGCTGGCCCTGGCC	CCAAAGCTGAGGTTACCTGAGAGATTTCCAGAGAAGCTGCAGCTGGCCCTGGCC
425	Mature_418	bta-miR-16_27259	chr16	36739113	36739131	-	19	66	66	0	0	0	0	0	Precursor_425	chr16_27259|chr16:36739042-36739131:-	90	66	GGGGAAGGAGAGGAGAAGG	GGGGAAGGAGAGGAGAAGGAGATCAACAATAATCCTGGGAGAGGGTTAACGGCCTCTGTCAGCATTCTCATCGGTGTCTCAGTTTCCCTT
426	Mature_419	bta-miR-16_27308	chr16	41500266	41500285	-	20	7	7	0	0	0	0	0	Precursor_426	chr16_27308|chr16:41500266-41500321:-	56	7	GGGCCCGCTGAGATCCTGGG	CAGGTCCCAGCGGAGCCTCAGAGCCGCTTTGGTACTGGGCCCGCTGAGATCCTGGG
427	Mature_420	bta-miR-16_31882	chr16	42668942	42668962	-	21	45	0	0	45	0	0	0	Precursor_427	chr16_31882|chr16:42668942-42669013:-	72	45	AGGGAAGGAAGAAAAGGAAAG	TTCTAATTAAGTATGACTGTCCCGGTTTTGAGCCGATGCGGAGCAGAGCGAAGGGAAGGAAGAAAAGGAAAG
428	Mature_421	bta-miR-16_26438	chr16	43998022	43998043	-	22	15	0	15	0	0	0	0	Precursor_428	chr16_26438|chr16:43998022-43998077:-	56	16	CACAAAGACAGGCAGCGCTGCC	CTGGGTTCAATCCCTGTTCAGGGAACTAAGATCCCACAAAGACAGGCAGCGCTGCC
429	Mature_422	bta-miR-16_28297	chr16	44117555	44117576	-	22	15	0	0	0	15	0	0	Precursor_429	chr16_28297|chr16:44117529-44117576:-	48	15	AGGGAAGTCCTTTAATGCCTGC	AGGGAAGTCCTTTAATGCCTGCTCTGTGCAGACAGAGGAGTGAACTGT
430	Mature_423	bta-miR-16_29646	chr16	44729077	44729099	+	23	60	0	0	0	0	60	0	Precursor_430	chr16_29646|chr16:44729077-44729141:+	65	60	AGGCCTCCTCTCAATCATATGGC	AGGCCTCCTCTCAATCATATGGCAATTAAAAAAAAAATGGTATGTGAATGAGCACCGAGGCCAGT
431	Mature_424	bta-miR-16_31189	chr16	50991767	50991791	+	25	115	0	0	115	0	0	0	Precursor_431	chr16_31189|chr16:50991702-50991791:+	90	115	CCCAACTGGGAGAAAGAGAGAAACC	TTTTGATCCCCGGTTGGGAAATTAAGATCCTGCACGCTTCATGGTGCAGCCAAAAAAGAAACCATCCCAACTGGGAGAAAGAGAGAAACC
432	Mature_425	bta-miR-16_26691	chr16	51178587	51178607	+	21	8	8	0	0	0	0	0	Precursor_432	chr16_26691|chr16:51178540-51178607:+	68	8	AGGAGAGAGAGGTTCAAGGGC	TACCAGCCTCTGTCCTGTGACTTGGGCTGAGAGTTAGGTGATCAGGTAGGAGAGAGAGGTTCAAGGGC
433	Mature_426	bta-miR-16_31974	chr16	52071540	52071559	-	20	17	0	0	17	0	0	0	Precursor_433	chr16_31974|chr16:52071540-52071623:-	84	17	GGAGGGAGAAGAAAGGGGGC	CTTCTGGGGTCCTCCAGTTCATCCATTCAGTGGTCATCAGCAGATTCTTCCTCAGGGTGGAGATGGAGGGAGAAGAAAGGGGGC
434	Mature_427	bta-miR-16_32039	chr16	57437827	57437844	-	18	54	0	0	54	0	0	0	Precursor_434	chr16_32039|chr16:57437761-57437844:-	84	54	GACTGAAGAGGAGAAAGG	GACTGAAGAGGAGAAAGGGCACAGATCCCCACATGTCACCAGAGGTAGGGCAAAGGGATGTGTGTGCTTAAGTCTCTCAGTCAT
435	Mature_428	bta-miR-16_27455	chr16	62412657	62412674	-	18	746	232	0	514	0	0	0	Precursor_435	chr16_27455|chr16:62412588-62412674:-	87	756	GAAAGAGAGGGAGAAAAA	GAAAGAGAGGGAGAAAAAGAGGAAAAGGGAATGGGAAAAGGAAAAGAAACCTGGCTCAGATTTCATCTAAACTTTCCCTGCTTTCCC
436	Mature_429	bta-miR-16_27529	chr16	70055574	70055591	-	18	86	86	0	0	0	0	0	Precursor_436	chr16_27529|chr16:70055515-70055591:-	77	86	GGAGCTATGGCAGGAGGG	GGAGCTATGGCAGGAGGGTGAGCATAACTCCTAAGTATGAGTTTATGCTGTGCATCCCCAACTGTTCTAGATTCCTG
437	Mature_430	bta-miR-16_27558	chr16	72383804	72383821	-	18	43	43	0	0	0	0	0	Precursor_437	chr16_27558|chr16:72383804-72383872:-	69	43	GAGAAAGAAAGGGAAGAG	CTTCTTGCTCACTATTGGGTCTACAGTGGCTGGCATGTGGAGGATGCTCGAGAGAAAGAAAGGGAAGAG
438	Mature_431	bta-miR-16_26052	chr16	74821114	74821131	+	18	11	0	11	0	0	0	0	Precursor_438	chr16_26052|chr16:74821114-74821194:+	81	11	GGAGCAGAAGGGCAAAGG	GGAGCAGAAGGGCAAAGGTGTCCAAGAAAATTGGCAGAGCGAAGCCTTTGCTTCTTCTCAGACCCTATCCCTCTGCTTCTT
439	Mature_432	bta-miR-16_26939	chr16	79637130	79637148	+	19	653	653	0	0	0	0	0	Precursor_439	chr16_26939|chr16:79637091-79637148:+	58	653	GGGGAGGAGGAGGAGGAGG	GCCTTCTCCCGCTCCTTGGTGAATTCCCTGCAGGGGGCCGGGGAGGAGGAGGAGGAGG
440	Mature_433	bta-miR-16_26103	chr16	79825049	79825068	+	20	22	0	22	0	0	0	0	Precursor_440	chr16_26103|chr16:79825024-79825068:+	45	22	AGGGCCAGGTGAGCTGCGGG	TGTGGAAGGCAGGCCCTGGAAGTGCAGGGCCAGGTGAGCTGCGGG
441	Mature_434	bta-miR-16_26116	chr16	80754650	80754668	+	19	55	0	55	0	0	0	0	Precursor_441	chr16_26116|chr16:80754650-80754709:+	60	55	GAGAGGGACGGGAGAAAGG	GAGAGGGACGGGAGAAAGGGGGGGAGCCCTGGGAGCCACCCCTTCCCCACCCTCCATCTC
442	Mature_435	bta-miR-17_32335	chr17	4886504	4886521	+	18	14	0	0	14	0	0	0	Precursor_442	chr17_32335|chr17:4886449-4886521:+	73	14	GGAGAAAAGGAAGGGAGG	TTCCATCTTTTTCTTCCTTAAGAACCGACTCAGAGAAATTACATGTTTTAATCCAGGAGAAAAGGAAGGGAGG
443	Mature_436	bta-miR-17_28287	chr17	5497708	5497727	-	20	73	36	0	37	0	0	0	Precursor_443	chr17_28287|chr17:5497647-5497727:-	81	73	GGAGGAAGACAAAGAGGAGG	GGAGGAAGACAAAGAGGAGGCTGGGGAGGGGGAGTTCCTTCTTCCCACCTTGCTCCAGGGCTCACTACATTGTCCTCAAG
444	Mature_437	bta-miR-17_26863	chr17	12731496	12731518	+	23	49	0	49	0	0	0	0	Precursor_444	chr17_26863|chr17:12731447-12731518:+	72	49	CTCACATGCCATGAAGCATAGCC	CTTCCACTGTAAGGGGTGTAGGTTTGATTCCTGGTCAGGGAAATAAGACCTCACATGCCATGAAGCATAGCC
445	Mature_438	bta-miR-17_31610	chr17	13269421	13269440	-	20	15	0	0	0	0	15	0	Precursor_445	chr17_31610|chr17:13269421-13269508:-	88	15	CAAGCATGTAGCACAGCCAC	GGTTAGGACTCCATGCTTTCACTGCCAAGGGTCCAGGTTTGAGCCCTGATTGGGGAACTAACAGCTCACAAGCATGTAGCACAGCCAC
446	Mature_439	bta-miR-17_28741	chr17	17469080	17469098	+	19	187	0	0	0	52	60	75	Precursor_446	chr17_28741|chr17:17469035-17469098:+	64	190	CATGCCTCGAGGTGTGACC	CAATACAGCGGGGCATGGGTTCCATCCCTGGTTGGGGAAGTTCAACATGCCTCGAGGTGTGACC
447	Mature_440	bta-miR-17_29449	chr17	23099205	23099227	-	23	74	0	0	0	74	0	0	Precursor_447	chr17_29449|chr17:23099164-23099227:-	64	74	CTTCTGGGAAAAGTCTTTTTCTC	CTTCTGGGAAAAGTCTTTTTCTCTTAAAAAAAAAAAGAGAGAGAGAGAGACTTTCTGGGAAAGA
448	Mature_441	bta-miR-17_32595	chr17	39745128	39745145	+	18	24	0	0	24	0	0	0	Precursor_448	chr17_32595|chr17:39745093-39745145:+	53	24	GGAAGAAGGAAAAAGAGG	TTTCCATTTTCTCCTAGAATCTATATGGGGATTAAGGAAGAAGGAAAAAGAGG
449	Mature_442	bta-miR-17_31828	chr17	39830110	39830129	-	20	235	0	0	0	0	122	113	Precursor_449	chr17_31828|chr17:39830110-39830186:-	77	235	CAGGGGGCATGTATAGCTCC	AAAGAGTGCCACCCCGGGCCCAAGGTTTAACTGTGTCCTTAGAGGAGGACTGGGCACAGGGGGCATGTATAGCTCC
450	Mature_443	bta-miR-17_28516	chr17	43944851	43944871	-	21	612	358	0	254	0	0	0	Precursor_450	chr17_28516|chr17:43944851-43944934:-	84	612	GGGGGTATGGTTGCAAAGCTG	GCTGTGTTGTTCTTCCATATCCTGCTGGACAGCTGAAGGGAAATGAAACTTTTTGGTTTCTTGGGGGGTATGGTTGCAAAGCTG
451	Mature_444	bta-miR-17_31069	chr17	48201891	48201908	-	18	258	0	0	0	0	0	258	Precursor_451	chr17_31069|chr17:48201822-48201908:-	87	258	GCTGGTGAGGGTGAAAAT	GCTGGTGAGGGTGAAAATTGCAGCCATTCCTCTGGAAAGCAATTTGCCAGAGTCTTTGCTATCAATTGTGTTTGCCCGTAACAGAGA
452	Mature_445	bta-miR-17_27998	chr17	52195269	52195290	+	22	94	82	12	0	0	0	0	Precursor_452	chr17_27998|chr17:52195269-52195346:+	78	94	CAGGGGCAGCAATGCCAACAGC	CAGGGGCAGCAATGCCAACAGCAACAAGGAGGAGAAGACAATCATAAGGAAACTGTAAGTTGACTTGCTGCCCCACGT
453	Mature_446	bta-miR-17_28039	chr17	53485996	53486014	+	19	12	12	0	0	0	0	0	Precursor_453	chr17_28039|chr17:53485954-53486014:+	61	12	GAGGATGATGATGAAGAGG	TCTTCCTCATCCTCCCCAGCTGGCAGCTCTTCTTCATCTTCTGAGGATGATGATGAAGAGG
454	Mature_447	bta-miR-17_28063	chr17	56034897	56034914	+	18	17	17	0	0	0	0	0	Precursor_454	chr17_28063|chr17:56034847-56034914:+	68	17	GAGAGGAGAAGCAGATAG	ATTGCCTACCTCTCAGTGTTGTAGAGTGAATGGAATTGGTCAGTTCATCAGAGAGGAGAAGCAGATAG
455	Mature_448	bta-miR-17_31327	chr17	57120064	57120081	+	18	11	0	0	0	0	11	0	Precursor_455	chr17_31327|chr17:57120035-57120081:+	47	11	CTGTCCTGTGATTTTTCA	GAGAGTGTGGGAACATGCTGTAGCTTCAGCTGTCCTGTGATTTTTCA
456	Mature_449	bta-miR-17_28649	chr17	57761340	57761357	-	18	17	17	0	0	0	0	0	Precursor_456	chr17_28649|chr17:57761323-57761357:-	35	17	GGAAAGGAAGAGAGAAAG	GGAAAGGAAGAGAGAAAGAGATCCTCCCCTTCCTC
457	Mature_450	bta-miR-17_27266	chr17	60548931	60548948	+	18	38	0	11	0	0	0	27	Precursor_457	chr17_27266|chr17:60548931-60549008:+	78	38	CCCAAGTGTCCTGAATAC	CCCAAGTGTCCTGAATACGAGGCAAAATGACTACATGCATTAGTCATACTGTGCACTCGACAGGCACGCACTTGACAA
458	Mature_451	bta-miR-17_27949	chr17	64300134	64300154	-	21	12	0	12	0	0	0	0	Precursor_458	chr17_27949|chr17:64300105-64300154:-	50	12	TGGCTCTGCACTTTTGTAAAG	TGGCTCTGCACTTTTGTAAAGAAAAAAAAAAAAGTGTCACCTATAGCCAGA
459	Mature_452	bta-miR-17_27968	chr17	66214678	66214697	-	20	11	0	11	0	0	0	0	Precursor_459	chr17_27968|chr17:66214678-66214731:-	54	11	CTGCTTTGATTGATTTAGGT	CTCCAAGATTGAGGCAGGGGGGCAGGCGTGGGCCCTGCTTTGATTGATTTAGGT
460	Mature_453	bta-miR-17_27973	chr17	67290989	67291009	-	21	43	0	43	0	0	0	0	Precursor_460	chr17_27973|chr17:67290946-67291009:-	64	43	CACCCTGCTTAGTAAAAATGC	CACCCTGCTTAGTAAAAATGCAAAAAAAAAAAAAAAAAAGTATTTTTCCCTCAAGTGGGATGAA
461	Mature_454	bta-miR-17_27351	chr17	69509825	69509847	+	23	21	0	21	0	0	0	0	Precursor_461	chr17_27351|chr17:69509781-69509847:+	67	24	TCCCACATGCCCTGAGGTTTGGC	CAGTGCAGGGGGTGCCGGTTCCATCCCTGGTTGGGGAACTAGGATCCCACATGCCCTGAGGTTTGGC
462	Mature_455	bta-miR-17_27353	chr17	69893538	69893557	+	20	24	0	24	0	0	0	0	Precursor_462	chr17_27353|chr17:69893474-69893557:+	84	24	AGGTCACAATGGAAGGGCAG	GACCTCAGGGAATTCACAGTCCTGAGGGGAAAATGGACACAAGTAATTACCAAAAATGCACCTCAGGTCACAATGGAAGGGCAG
463	Mature_456	bta-miR-17_28017	chr17	70590781	70590798	-	18	185	0	185	0	0	0	0	Precursor_463	chr17_28017|chr17:70590741-70590798:-	58	185	AGGAGGGGAAGGGGAAAC	AGGAGGGGAAGGGGAAACTGAGAGGAAAGCTGGCTGTTTAAGCACATCCACCTCCTCC
464	Mature_457	bta-miR-17_33679	chr17	71709932	71709950	-	19	23	0	0	23	0	0	0	Precursor_464	chr17_33679|chr17:71709893-71709950:-	58	23	AAGAAGGTCGGGGGCAAGG	AAGAAGGTCGGGGGCAAGGAGAAAAGAAACAGGCAAAGCTTCCAGGCTGGACTTGTGG
465	Mature_458	bta-miR-18_34594	chr18	2784028	2784045	-	18	15	0	0	15	0	0	0	Precursor_465	chr18_34594|chr18:2784028-2784109:-	82	15	GAGGAAGAGGAGGATGCC	TATTTTGTTTTTTCTTGTGATGGTGATTAGGAAGAGAAAACACAGTGGCCTCTCACCACAACACGAGGAAGAGGAGGATGCC
466	Mature_459	bta-miR-18_29470	chr18	3480469	3480486	-	18	9	9	0	0	0	0	0	Precursor_466	chr18_29470|chr18:3480469-3480527:-	59	9	ACAGACACCAGTGCAAGG	TGCCTGCCCAGGAATCTGGCCAGTGATGCTCCTTCAACTGCACAGACACCAGTGCAAGG
467	Mature_460	bta-miR-18_29497	chr18	6377307	6377324	-	18	50	10	0	40	0	0	0	Precursor_467	chr18_29497|chr18:6377282-6377324:-	43	50	GAGGAAGGGAGCAGCGGC	GAGGAAGGGAGCAGCGGCTTCTTCCGAAGCTCCCATGTCTCAG

468	Mature_461	bta-miR-18_29502	chr18	6524494	6524512	-	19	11	11	0	0	0	0	0	Precursor_468	chr18_29502|chr18:6524494-6524575:-	82	11	AGGTTGAGGGAGAGGAGAG	CTCCTTTCCTCCAGCCCGGCTGCACCTCTGTCTTGCACTTTGCACGGAGGGAGGAAGAGCGCGAGGTTGAGGGAGAGGAGAG
469	Mature_462	bta-miR-18_34648	chr18	7442125	7442148	-	24	27	0	0	27	0	0	0	Precursor_469	chr18_34648|chr18:7442125-7442203:-	79	27	AGACAAGAGGAAGAACAAGAAGGG	CTTCTCTCTCTCTCTTTCCTGATTCTCCTCCCGCATCCCAGGTCGAAAGGATTGTAGACAAGAGGAAGAACAAGAAGGG
470	Mature_463	bta-miR-18_29996	chr18	10048400	10048417	+	18	1099	0	0	0	381	299	419	Precursor_470	chr18_29996|chr18:10048400-10048481:+	82	1099	CCACCCAAGATCAGAACC	CCACCCAAGATCAGAACCCAGAAGGGACAGAGGATGATTTTCCTCATCTTCCCTCTCTCACTGGGTCTGATTCCCAAAGGAA
471	Mature_464	bta-miR-18_34688	chr18	11123542	11123563	-	22	28	0	0	28	0	0	0	Precursor_471	chr18_34688|chr18:11123542-11123628:-	87	28	GGAGAGCACACAGCCAACGAGG	TCGTTTGCGGGTTTCCCACATGCGTCTTCTCTAACTTCACGGAAGTGAGCTCTTGGAGATCATCTGGAGAGCACACAGCCAACGAGG
472	Mature_465	bta-miR-18_30696	chr18	11144870	11144887	-	18	8086	0	0	0	2701	2545	2840	Precursor_472	chr18_30696|chr18:11144870-11144929:-	60	8086	GAGGCTGGTGGGGTTGCC	CAAGTTCACAGGCTCTGTGGTGGGAATGAACTTGGCCTGGTTGAGGCTGGTGGGGTTGCC
473	Mature_466	bta-miR-18_28127	chr18	11364643	11364660	+	18	73	0	73	0	0	0	0	Precursor_473	chr18_28127|chr18:11364618-11364660:+	43	73	GCCCGGCCGGGGGGGCGG	GACCCGCCATCGGGCGCTCAGCTGGGCCCGGCCGGGGGGGCGG
474	Mature_467	bta-miR-18_29582	chr18	13774081	13774098	-	18	7	7	0	0	0	0	0	Precursor_474	chr18_29582|chr18:13774081-13774142:-	62	7	GGCATGGGGGCGAGTGCT	CCTGCCCCCCTCCCTCGAGAGCCACTCCTGGATGGCACAGGAGGGGCATGGGGGCGAGTGCT
475	Mature_468	bta-miR-18_28148	chr18	13926861	13926880	+	20	113	0	113	0	0	0	0	Precursor_475	chr18_28148|chr18:13926861-13926907:+	47	113	GGGGAGGGTCGGGGCGGGGG	GGGGAGGGTCGGGGCGGGGGCTGAGGCCCTGGCTACTGACACCCCGG
476	Mature_469	bta-miR-18_28193	chr18	18309197	18309216	+	20	19	0	19	0	0	0	0	Precursor_476	chr18_28193|chr18:18309131-18309216:+	86	19	TCGGCCGAGCCGAGTTATGC	AATGCGGCAGCCGGGGGCTCTCGGTCCCAGCGGCGGTCCCAGCGGCGCGGGGAGTCCGGAGGCCACTCGGCCGAGCCGAGTTATGC
477	Mature_470	bta-miR-18_28930	chr18	19052645	19052663	+	19	885	211	156	518	0	0	0	Precursor_477	chr18_28930|chr18:19052645-19052707:+	63	885	GGAGAAAGGAAGAGGGAGG	GGAGAAAGGAAGAGGGAGGCCACCAGATTCTCTCATTAGTCTCCCTGACATCTTTTGTCACCT
478	Mature_471	bta-miR-18_28939	chr18	19886652	19886670	+	19	20	20	0	0	0	0	0	Precursor_478	chr18_28939|chr18:19886608-19886670:+	63	20	GGGAAAAGGGAGAGAAAAG	TTTTTCCTCTTTCTTCTTTTTTATTTAAAAAAGAAGACATTATGGGGAAAAGGGAGAGAAAAG
479	Mature_472	bta-miR-18_30842	chr18	26423593	26423617	-	25	158	0	0	0	67	0	91	Precursor_479	chr18_30842|chr18:26423593-26423652:-	60	158	GATCCTGCAAGCCAACAGGTATGGC	CCGGCCCTGGCTTCAATTCTTGGATGGGAAACCAAGATCCTGCAAGCCAACAGGTATGGC
480	Mature_473	bta-miR-18_34917	chr18	32288382	32288399	-	18	504	0	0	504	0	0	0	Precursor_480	chr18_34917|chr18:32288317-32288399:-	83	504	AGGAGGAGGAGGAAGAGG	AGGAGGAGGAGGAAGAGGGAGAGAGGAGCAAGGCAAACAAAACGTCTATCCTACAAGAATTCTCTTCTCCTTGGTCCCTCTGT
481	Mature_474	bta-miR-18_29026	chr18	36106219	36106243	-	25	12	0	12	0	0	0	0	Precursor_481	chr18_29026|chr18:36106219-36106275:-	57	12	TCCCACAAGTCATGAGGTGTGGGCC	CCCAGGTTCAATCCCCGATCAGGGAACTAAAATCCCACAAGTCATGAGGTGTGGGCC
482	Mature_475	bta-miR-18_29088	chr18	39807603	39807622	+	20	11	11	0	0	0	0	0	Precursor_482	chr18_29088|chr18:39807603-39807672:+	70	11	GGGGGAAGAAAAAAGGGGAT	GGGGGAAGAAAAAAGGGGATGAGAGGAATTCCAGAGGGAGGCTCTTGTTCTGTTGCTGTTCTCTTCCTAT
483	Mature_476	bta-miR-18_29782	chr18	41916674	41916692	-	19	9	9	0	0	0	0	0	Precursor_483	chr18_29782|chr18:41916674-41916733:-	60	9	GGGACGGGGCGGGGGGTGG	ACTCCCCGCCCCCCGGCCCCGAACCCCGCGCACCTCGTCTGGGGACGGGGCGGGGGGTGG
484	Mature_477	bta-miR-18_31773	chr18	44028436	44028454	+	19	47	0	0	0	0	0	47	Precursor_484	chr18_31773|chr18:44028368-44028454:+	87	47	CCTAAGACTCGGTGCAGCC	GTGCAGAGGAGTCTGGGTTTGATCCCCAGGCAGGGAATTACATCCTGCAACTTGCAACTAAGATCCCACCTAAGACTCGGTGCAGCC
485	Mature_478	bta-miR-18_29144	chr18	48020217	48020238	-	22	11	0	11	0	0	0	0	Precursor_485	chr18_29144|chr18:48020217-48020284:-	68	11	CCACATGCCCCACTGCTCCGTC	CAAGGCAGGGGGTGTGGGTTCCAGCCCTGGTTGGGAAACTAATATCCCACATGCCCCACTGCTCCGTC
486	Mature_479	bta-miR-18_29168	chr18	50140038	50140056	+	19	9	9	0	0	0	0	0	Precursor_486	chr18_29168|chr18:50140038-50140115:+	78	9	GAATTTTGGGAGACGTGGG	GAATTTTGGGAGACGTGGGGTTCGTGTCCCTCTAGGCAGGATGCTTGGGGGACATGAGATCATGTCCTTGAAGGTCTA
487	Mature_480	bta-miR-18_29183	chr18	51022470	51022487	-	18	104	0	104	0	0	0	0	Precursor_487	chr18_29183|chr18:51022432-51022487:-	56	104	GGAGGGGAAGGGGGAAAG	GGAGGGGAAGGGGGAAAGTCCGAGTGGGAGGGGGGGTTAATCCTGCTGCCCCCCGG
488	Mature_481	bta-miR-18_29189	chr18	51333908	51333930	-	23	15	0	15	0	0	0	0	Precursor_488	chr18_29189|chr18:51333851-51333930:-	80	15	CAGTGGGGAGTTTGGTGTCATGG	CAGTGGGGAGTTTGGTGTCATGGGCCCCAGGGAAGGGGGAGTCTACCTGGGGGCCTGGGCGTTGAGCTGCACGCCTGGCC
489	Mature_482	bta-miR-18_29943	chr18	53694721	53694738	-	18	62	18	44	0	0	0	0	Precursor_489	chr18_29943|chr18:53694691-53694738:-	48	62	GGGAAGAAAAAGGGGAAG	GGGAAGAAAAAGGGGAAGAGGAAGGGATTTCTCCTTTGCCTCGCTCCC
490	Mature_483	bta-miR-18_28515	chr18	54360682	54360701	+	20	11	0	11	0	0	0	0	Precursor_490	chr18_28515|chr18:54360631-54360701:+	71	11	GGAAAGGTGGGCAGGGGAAC	TTTTCTGCCCATCTTTATCTTACGAGGCTAGCTGTGTAACAGGAACTTTCTGGAAAGGTGGGCAGGGGAAC
491	Mature_484	bta-miR-18_35182	chr18	54685718	54685737	-	20	18	0	0	18	0	0	0	Precursor_491	chr18_35182|chr18:54685718-54685796:-	79	18	GGGGGGCGGAGGGAGAGGGG	CCTGCCCTCTGCACCCCTTCCCGCTGCCTCCTGAACCCCAGGAGCCTCTAATTGGGTCTGGGGGGCGGAGGGAGAGGGG
492	Mature_485	bta-miR-18_34369	chr18	54726855	54726874	+	20	18	0	0	18	0	0	0	Precursor_492	chr18_34369|chr18:54726797-54726874:+	78	18	GGGGGGCGGAGGGAGAGGGG	CTGCCCTCTGCACCCCTTCCCGCTGCCTCCTGAACCCCAGGAGCCTCTAATTGGGTCTGGGGGGCGGAGGGAGAGGGG
493	Mature_486	bta-miR-18_29966	chr18	54881778	54881797	-	20	15	15	0	0	0	0	0	Precursor_493	chr18_29966|chr18:54881778-54881864:-	87	15	CCCCCCGGAAGCCAGCTGTC	GAGTACTTGGGAGGTGGGGGCTGTGGGAAGGAGAAAGGGTCCACTGTTTCCCTGCTTCATTCCCCAGCCCCCCGGAAGCCAGCTGTC
494	Mature_487	bta-miR-18_29998	chr18	57685750	57685767	-	18	7	7	0	0	0	0	0	Precursor_494	chr18_29998|chr18:57685750-57685822:-	73	7	GGGGAGGAGGGAGCGGGG	CCGCCCCTGCCCCGGAGCTGGCCCCGGTCCAGGGGAAAAAATTCCATCCAGCCCCGGGGAGGAGGGAGCGGGG
495	Mature_488	bta-miR-18_30005	chr18	59002747	59002765	-	19	17	17	0	0	0	0	0	Precursor_495	chr18_30005|chr18:59002704-59002765:-	62	17	GCCTGAGGAAGAAGCCAAG	GCCTGAGGAAGAAGCCAAGAAGATGAAAGAAAATGTGTCAGGAATTGCTCTTTCTCAGGTAA
496	Mature_489	bta-miR-18_30011	chr18	59196129	59196147	-	19	17	17	0	0	0	0	0	Precursor_496	chr18_30011|chr18:59196086-59196147:-	62	17	GCCTGAGGAAGAAGCCAAG	GCCTGAGGAAGAAGCCAAGAAGATGAAAGAAAATGTGTCAGGAATCCCTCTTTCTCAGGTAA
497	Mature_490	bta-miR-18_29343	chr18	59225663	59225681	+	19	15016	6703	0	8313	0	0	0	Precursor_497	chr18_29343|chr18:59225642-59225681:+	40	15155	CCCCCGCGGGGGCCCGGGC	CGGTGGCGGGGGGAGGTCTTCCCCCCGCGGGGGCCCGGGC
498	Mature_491	bta-miR-18_30025	chr18	60047970	60047988	-	19	46	17	0	29	0	0	0	Precursor_498	chr18_30025|chr18:60047917-60047988:-	72	46	AGGAAGAGGAAAAAGGAGG	AGGAAGAGGAAAAAGGAGGTCCTTATTAGAGTTGCACATTATTGTCAGGACTTCCTGTCTTCATCTTCCACA
499	Mature_492	bta-miR-18_30567	chr18	62311811	62311828	+	18	41	0	0	0	18	0	23	Precursor_499	chr18_30567|chr18:62311774-62311828:+	55	41	TTCCATTCTTTTCTTTTG	AGGGAAAGGATCAAAAGACTTCCGTTTCTATCTCGTTTTCCATTCTTTTCTTTTG
500	Mature_493	bta-miR-19_29413	chr19	10157766	10157783	+	18	251	0	251	0	0	0	0	Precursor_500	chr19_29413|chr19:10157708-10157783:+	76	256	CATATTACTAAGTGGAGG	CAGCACTAAAAAGAAATGAGCTAGCAAATCATGAAAGACATAGAATAAACTTAAGATGCATATTACTAAGTGGAGG
501	Mature_494	bta-miR-19_35417	chr19	10794864	10794883	+	20	26	0	0	26	0	0	0	Precursor_501	chr19_35417|chr19:10794810-10794883:+	74	26	GGAAGGGCAAGAGAAAAGAG	CTGGGTTCTTAAAGGTCCTCTTTACAGTGGTCTGTAAGAGCCACTCTAAAGCATGGAAGGGCAAGAGAAAAGAG
502	Mature_495	bta-miR-19_34500	chr19	18905748	18905771	-	24	63	0	0	0	0	63	0	Precursor_502	chr19_34500|chr19:18905748-18905797:-	50	65	TCCCACATGTCTCAGGGTGTAGCC	TTTGATCCCTGATCAGGGAGCTAAGATCCCACATGTCTCAGGGTGTAGCC
503	Mature_496	bta-miR-19_29607	chr19	27017736	27017753	+	18	12	0	12	0	0	0	0	Precursor_503	chr19_29607|chr19:27017672-27017753:+	82	12	GAGACAGGAGATGCAGGG	CTAGTTCTCTTCTTGACTCTAGTTGGAATCGTAGCCTCTTAAAATGGCAGATGATTTGGACTTCGAGACAGGAGATGCAGGG
504	Mature_497	bta-miR-19_30256	chr19	27787962	27787984	-	23	13	0	13	0	0	0	0	Precursor_504	chr19_30256|chr19:27787962-27788026:-	65	13	TGGAGGGGGTCCATCCCCTGGGC	TGAAGGGGGAGGGGACCCCAGGCCAGGGGAACCCTTTTGTCCTGGAGGGGGTCCATCCCCTGGGC
505	Mature_498	bta-miR-19_31096	chr19	31359952	31359969	-	18	645	22	560	63	0	0	0	Precursor_505	chr19_31096|chr19:31359952-31359989:-	38	645	CGCCGACTCCCGGGGCCG	GGGCGGGCATCAGGCGGCAGCGCCGACTCCCGGGGCCG
506	Mature_499	bta-miR-19_33053	chr19	32162983	32163000	+	18	17	0	0	0	0	0	17	Precursor_506	chr19_33053|chr19:32162948-32163000:+	53	17	CAGACACAGAATTGAGGC	CCACGTCTGTGCTGAGATATATATTCTGAATATATCAGACACAGAATTGAGGC
507	Mature_500	bta-miR-19_31595	chr19	38898511	38898535	+	25	106	0	0	0	33	73	0	Precursor_507	chr19_31595|chr19:38898444-38898534:+	93	114	GATCCCACAAGCCATGGTGCTGCCA	GGTGGTCTAGTGGTTAGGATTTGGCACTTTCACTGCCATGGCTTTCAATCCCTGGTCAGGAACTGAGAGATCCCACAAGCCATGGTGCTGCCA
508	Mature_501	bta-miR-19_29709	chr19	39066236	39066254	+	19	11	0	11	0	0	0	0	Precursor_508	chr19_29709|chr19:39066170-39066254:+	85	11	CCACCACCTACTTCATCCC	GATGGACAGGGGATGGAAGTGGAGGTCATTATCCCTGAGTTCCTTGGATCGCCCCCTCCACACACTCCACCACCTACTTCATCCC
509	Mature_502	bta-miR-19_31194	chr19	39240099	39240116	-	18	21	21	0	0	0	0	0	Precursor_509	chr19_31194|chr19:39240099-39240167:-	69	27	AGCAGCAGCGGCAGTCAC	TGGGCTGCTGTCTATGGGGTCACACAGAGTCGGACACGACTGAAGCGACTTAGCAGCAGCGGCAGTCAC
510	Mature_503	bta-miR-19_32270	chr19	39324122	39324140	-	19	52	0	0	0	31	21	0	Precursor_510	chr19_32270|chr19:39324122-39324182:-	61	52	CTCCTGGGTTTGCAAACTG	GTTGAGGATATAAAGGAGGCTTTACAAAGAATGTAAAAGGAGCTCCTGGGTTTGCAAACTG
511	Mature_504	bta-miR-19_35798	chr19	39379234	39379253	+	20	16	0	0	16	0	0	0	Precursor_511	chr19_35798|chr19:39379234-39379278:+	45	16	GGCAGCAGGAAGCAGAGAGA	GGCAGCAGGAAGCAGAGAGAACAAACTCTGCTGGACTGTTGGATC
512	Mature_505	bta-miR-19_29736	chr19	40585488	40585509	+	22	11	0	11	0	0	0	0	Precursor_512	chr19_29736|chr19:40585488-40585537:+	50	11	GGGGAGGATTCTGCTAGTGGGG	GGGGAGGATTCTGCTAGTGGGGGCTGCCCCTGCTGGCGGGGTCACCCTCC
513	Mature_506	bta-miR-19_34041	chr19	40987815	40987838	+	24	168	0	0	0	0	168	0	Precursor_513	chr19_34041|chr19:40987752-40987838:+	87	182	CCATGTCCTGTGGGGCGGATCAGC	TGAGACTTATGCTTCCACTGCAGTGGCCATGGGTTCCATCCCTGGTTAGGGAACTGAGAGCCCCCATGTCCTGTGGGGCGGATCAGC
514	Mature_507	bta-miR-19_36815	chr19	45050532	45050554	-	23	31	0	0	31	0	0	0	Precursor_514	chr19_36815|chr19:45050495-45050554:-	60	31	GGGCCCCCAAAGACAAATCCAGC	GGGCCCCCAAAGACAAATCCAGCAAGAAGAATGTGCTGGAGGTGAGTGGTGGGGGTGAGG
515	Mature_508	bta-miR-19_35904	chr19	46361642	46361659	+	18	15	0	0	15	0	0	0	Precursor_515	chr19_35904|chr19:46361642-46361718:+	77	15	GAGGAAGAAGAGGAGAAG	GAGGAAGAAGAGGAGAAGACTTAATGTCCTTCTGCCTTACAGATGATTCGAAGATTTTCTCCGTCCAAGTTCGGCAG
516	Mature_509	bta-miR-19_29857	chr19	49127900	49127920	+	21	11219	0	8242	295	856	841	985	Precursor_516	chr19_29857|chr19:49127860-49127920:+	67	11188	GGGGGGCCGGCGGCGGCGGCG	CCGCCCCCGCCTCCTCGCCCCCCCCGGCCCGGGGCGCTGGTGCTGGGGGGGGCCGGCGGCGGCGG
517	Mature_510	bta-miR-19_30593	chr19	49674234	49674253	+	20	272	77	0	195	0	0	0	Precursor_517	chr19_30593|chr19:49674182-49674253:+	72	272	GGGAGAAGAGAAGGGGAGGC	CTTCCCTTCTGTTCGTCCTTCTATCTTACTCTGTTCCTTTTCAGAGTGGACGGGGAGAAGAGAAGGGGAGGC
518	Mature_511	bta-miR-19_36964	chr19	56673861	56673878	-	18	19	0	0	19	0	0	0	Precursor_518	chr19_36964|chr19:56673861-56673909:-	49	19	GGAGGCCGTGGGACTGGG	CAGTCCCAGGGCCCATCTACAGCCTGCGTTCGGAGGCCGTGGGACTGGG
519	Mature_512	bta-miR-19_30602	chr19	58241850	58241867	-	18	20	0	20	0	0	0	0	Precursor_519	chr19_30602|chr19:58241850-58241925:-	76	20	CTGGCCTCAGCTGCCTGG	AGGCCCAGGCCCAGGCCCAGTGCTCTCTCTACCACTGTGTTGGCTCAGAGCCTGCCTCCTGGCCTCAGCTGCCTGG
520	Mature_513	bta-miR-20_31500	chr20	4801384	4801401	+	18	12	12	0	0	0	0	0	Precursor_520	chr20_31500|chr20:4801384-4801459:+	76	12	GGGAAAGGGGGGACAAAG	GGGAAAGGGGGGACAAAGAAAAAAGAAGGAGCATTAACTCTGGGTGCTGAGTCTTTTTATTTTCTCCCTTTCTTAT
521	Mature_514	bta-miR-20_31999	chr20	9028820	9028837	-	18	39	13	0	26	0	0	0	Precursor_521	chr20_31999|chr20:9028777-9028837:-	61	39	GCCCAGGGGAGGAGGAAG	GCCCAGGGGAGGAGGAAGCTAGTGGATATGTGCCCCAGCTTCCTTGCCCTTAGGAGGGACA
522	Mature_515	bta-miR-20_31623	chr20	23103887	23103904	+	18	54	54	0	0	0	0	0	Precursor_522	chr20_31623|chr20:23103839-23103904:+	66	54	GGGGAGAAGGAAAGAAAG	CTCTTAACCATTTACCCATCCTGTGTCTGGGCTCTTGGACTCTGGAAGGGGGAGAAGGAAAGAAAG
523	Mature_516	bta-miR-20_34205	chr20	23571078	23571099	+	22	31	0	0	0	0	0	31	Precursor_523	chr20_34205|chr20:23571045-23571099:+	55	31	TCGGCTGTACTGACACGTTGGC	TGCGTCTGTGCAGCCAACAGGGATCAGGAGGGCTCGGCTGTACTGACACGTTGGC
524	Mature_517	bta-miR-20_37363	chr20	35788291	35788309	+	19	22	0	0	22	0	0	0	Precursor_524	chr20_37363|chr20:35788262-35788309:+	48	22	GAAAAGAAATAGAACTGAG	TAGTTTTATTTTTTTTTTTAAGGAGTAAGAAAAGAAATAGAACTGAG
525	Mature_518	bta-miR-20_32246	chr20	38427981	38427999	-	19	31	31	0	0	0	0	0	Precursor_525	chr20_32246|chr20:38427981-38428029:-	49	31	GAAAAAGAGGAAAAAGAGG	TCTTCCTGCCTAAAATTCTTTAAGATAGAGGAAAAAGAGGAAAAAGAGG
526	Mature_519	bta-miR-20_32861	chr20	40281412	40281434	+	23	80	0	0	0	51	29	0	Precursor_526	chr20_32861|chr20:40281344-40281434:+	91	84	CATACTGTTTTCCATCACTTTCC	AGAAACATGGGGAAAGCAGTGGTTGTTTTGCTTAGTGCCTAAAGGAAGCAGGCAAAGATAAAAATGCACATACTGTTTTCCATCACTTTCC
527	Mature_520	bta-miR-20_30994	chr20	40335965	40335988	+	24	23	0	23	0	0	0	0	Precursor_527	chr20_30994|chr20:40335965-40336034:+	70	23	GTGCAGGAAAGTAGAAGCAGCTGC	GTGCAGGAAAGTAGAAGCAGCTGCAAAAAAAAAAAAAAAAAAAATCAGTTGCTGGCAGTATCCTGATGGCA
528	Mature_521	bta-miR-20_35539	chr20	53333169	53333193	+	25	82	0	0	0	0	82	0	Precursor_528	chr20_35539|chr20:53333102-53333193:+	92	82	CCCCAAAGTATGCACGATGGGAGGG	TTCCGCACGACCTTGCTACGAGGGGGCTGAAGGGTGTCCGATCTGTGTGCTGTGGAATTTCTGCAGCCCCCAAAGTATGCACGATGGGAGGG
529	Mature_522	bta-miR-20_38046	chr20	55847688	55847705	-	18	21	0	0	21	0	0	0	Precursor_529	chr20_38046|chr20:55847688-55847747:-	60	21	GAAAGAGGAAAGGGAAAG	GCCCCTTTCCGGGTTGTAAGGATGCTCATGATGAGAGAGACAGAAAGAGGAAAGGGAAAG
530	Mature_523	bta-miR-20_36189	chr20	58693725	58693749	-	25	33	0	0	0	0	33	0	Precursor_530	chr20_36189|chr20:58693658-58693749:-	92	33	TCTGGGCAAAATGAGTCTCTAGTTG	TCTGGGCAAAATGAGTCTCTAGTTGAAAAGAAAAAAATAAGATTTACTGGTTTTTTTCTTTTCAACTTAAGAGACTCAGTTTTGCCCAAATC
531	Mature_524	bta-miR-20_35578	chr20	59001256	59001273	+	18	288	0	0	0	0	288	0	Precursor_531	chr20_35578|chr20:59001256-59001309:+	54	288	GGGGAAGGGGGGGGGGGG	GGGGAAGGGGGGGGGGGGGGGGCCTTGTCCCGACCCGCAAACCCTTTCTCCAT
532	Mature_525	bta-miR-20_31146	chr20	61086454	61086478	+	25	16	0	16	0	0	0	0	Precursor_532	chr20_31146|chr20:61086454-61086538:+	85	22	ACTCTGCTGAGCATCAGGGACAATT	ACTCTGCTGAGCATCAGGGACAATTAAAAAAAAAAAAAATAGGAGGAGGAAGATTCTCTGTGTGTGTCCTTGGGCTCAGTTGTGT
533	Mature_526	bta-miR-20_31193	chr20	67567393	67567411	+	19	17	0	17	0	0	0	0	Precursor_533	chr20_31193|chr20:67567393-67567445:+	53	17	AAGCAAGAATACTGGAGTG	AAGCAAGAATACTGGAGTGAGTTGTCATTTCCTCCTCCAGTGTATCTGCTTGA
534	Mature_527	bta-miR-20_37554	chr20	70987136	70987154	+	19	25	0	0	25	0	0	0	Precursor_534	chr20_37554|chr20:70987136-70987201:+	66	25	GGGTTCCGGGGGGGGGGGG	GGGTTCCGGGGGGGGGGGGGGGCGGGTCCCTCCCCTCCCTCCCCTCCCTCCCCTCCCTCGCCCTC
535	Mature_528	bta-miR-21_35844	chr21	1995825	1995842	-	18	17	0	0	0	0	0	17	Precursor_535	chr21_35844|chr21:1995825-1995863:-	39	17	TACACACATATTTGATGC	ATATATATATGTGTGTATATATACACACATATTTGATGC
536	Mature_529	bta-miR-21_38921	chr21	3230941	3230958	-	18	13	0	0	13	0	0	0	Precursor_536	chr21_38921|chr21:3230890-3230958:-	69	13	GGGAGCCTGGGCAGCAGC	GGGAGCCTGGGCAGCAGCCCTACAGAGGGGCGATGGCCATTTTCATGGAGCTTGTGCCCTTGCTTCCTC
537	Mature_530	bta-miR-21_33214	chr21	21634563	21634582	-	20	77	77	0	0	0	0	0	Precursor_537	chr21_33214|chr21:21634563-21634645:-	83	77	GGAGGAAGAGCAGAAATCAG	GATGAGCTGGTGCTTCACTCTCATACTTGAAGTTGGTCAGACTGATGGCCAGGGTTCTGGAATGGAGGAAGAGCAGAAATCAG
538	Mature_531	bta-miR-21_32596	chr21	24246955	24246972	-	18	19	0	19	0	0	0	0	Precursor_538	chr21_32596|chr21:24246955-24247035:-	81	19	CTACCATCAAATTTCCTC	GGACACATTTGCTGGGGTGGAATTAGGAGGCCAGAAGATGGACACATTGAAGGCATTTAATTCCTACCATCAAATTTCCTC
539	Mature_532	bta-miR-21_34605	chr21	28275674	28275692	-	19	31	0	0	0	31	0	0	Precursor_539	chr21_34605|chr21:28275674-28275744:-	71	31	AGGCTCAGTAGTTGTGGCA	CCACATTAGGTCTGAGTTGCAGCGTGTAGGCTTCTCTCTAGTGATGCCACACAGGCTCAGTAGTTGTGGCA
540	Mature_533	bta-miR-21_38483	chr21	32764005	32764022	+	18	990	0	0	990	0	0	0	Precursor_540	chr21_38483|chr21:32763957-32764022:+	66	1030	AGGAGGAAGAGGAGGAGG	TTCCCTGTTTTCCTGAGTGATCCCTCCCCACAGGGGAAGAGAAGAGAAAGGAGGAAGAGGAGGAGG
541	Mature_534	bta-miR-21_32106	chr21	34641973	34641993	+	21	19	0	19	0	0	0	0	Precursor_541	chr21_32106|chr21:34641973-34642051:+	79	19	GGGGAGGGAGAGAGAAGGGAC	GGGGAGGGAGAGAGAAGGGACCAAGCAGAGGTCCCTGCATTGGGAGGGATCTGAGATTGGGCTCGGTCTGCCTTGCTGA
542	Mature_535	bta-miR-21_39331	chr21	45640492	45640509	-	18	41	0	0	41	0	0	0	Precursor_542	chr21_39331|chr21:45640471-45640509:-	39	41	AGAAGGAAGAAGAGAGGC	AGAAGGAAGAAGAGAGGCCATTCTCCCTCCCCCTTCTAA
543	Mature_536	bta-miR-21_33450	chr21	46576574	46576591	-	18	83	83	0	0	0	0	0	Precursor_543	chr21_33450|chr21:46576574-46576658:-	85	83	GGAGTTATGGCACGAGGG	TTCTCATTGCAGTGGCTTCTCTTGTCGTGGAGCTCATGGAGCATGGGCTCTAGGCACAGGGACTTCAGGAGTTATGGCACGAGGG
544	Mature_537	bta-miR-21_32878	chr21	58195886	58195909	-	24	12	0	12	0	0	0	0	Precursor_544	chr21_32878|chr21:58195886-58195975:-	90	12	CCTCCAAATCCCATGCATCTTCTC	GAGGATGCTGTGGAAGGTGTTTCCAGCTGTCACACGGATTTCCTTCCTTAGCCAATGGAATTACTACCTCCAAATCCCATGCATCTTCTC
545	Mature_538	bta-miR-21_33584	chr21	63743016	63743033	-	18	28	28	0	0	0	0	0	Precursor_545	chr21_33584|chr21:63743016-63743073:-	58	28	GGGGAGGCAGCAGTGAGG	TCCTGATGCCTCTCCCTCTTATGCGGTTGTTGGAGGGGGAGGGGAGGCAGCAGTGAGG
546	Mature_539	bta-miR-21_34281	chr21	65319348	65319365	+	18	15	0	0	0	15	0	0	Precursor_546	chr21_34281|chr21:65319282-65319365:+	84	15	CCCCCAGGAGTAGCCCCC	GTGCCCCTCCCGGTGGGCAGACCCCTCCCCGTGGCTGCTTCCTGTGCAGCGCCGGGCCTGCAGCTGCCCCCAGGAGTAGCCCCC
547	Mature_540	bta-miR-21_35808	chr21	67275672	67275689	+	18	156	0	0	0	0	0	156	Precursor_547	chr21_35808|chr21:67275672-67275746:+	75	156	GCTGGCAGGGGCCCTGGC	GCTGGCAGGGGCCCTGGCAAAGGCAGAGGCCTGAGGTGTGAATCTCAGAGCTGCAGCGCGCAGGCCAGTGGGGGA
548	Mature_541	bta-miR-22_34192	chr22	2110065	2110085	-	21	22	22	0	0	0	0	0	Precursor_548	chr22_34192|chr22:2110065-2110150:-	86	22	GGAGACAGCAAGGGGGTCGCC	TCACCTCATGTGCTAGGCCTCTGCTGTGTGCCCCTGGGGGCTAGCCGGGGGCCTCATAGGACTCAGGAGACAGCAAGGGGGTCGCC
549	Mature_542	bta-miR-22_33567	chr22	5939307	5939331	-	25	18	0	18	0	0	0	0	Precursor_549	chr22_33567|chr22:5939251-5939331:-	81	18	GGTCTATTCCATTCTCTTTTAAAAT	GGTCTATTCCATTCTCTTTTAAAATAAAAAAAAAATTTTTTTTTCCAAAGGGGAAAGGTAGGGGAGGGAGGAATAAATTAG
550	Mature_543	bta-miR-22_33031	chr22	7989065	7989087	+	23	20	0	20	0	0	0	0	Precursor_550	chr22_33031|chr22:7989034-7989087:+	54	20	TGAGGTCTTGCAAGCTGTGTGGC	TTCCACTGCAAGGGACCCAGGCTCAGGGAACTGAGGTCTTGCAAGCTGTGTGGC
551	Mature_544	bta-miR-22_40192	chr22	10132042	10132059	-	18	20	0	0	20	0	0	0	Precursor_551	chr22_40192|chr22:10132042-10132087:-	46	20	AGGGAAAAAAGAGAGAAG	TCTCTCTGGGCCTTTATTTACTCAACAGAGGGAAAAAAGAGAGAAG
552	Mature_545	bta-miR-22_35064	chr22	11830652	11830670	+	19	129	0	0	0	129	0	0	Precursor_552	chr22_35064|chr22:11830652-11830721:+	70	129	TGGGAAGGGGGGGGGGGGG	TGGGAAGGGGGGGGGGGGGTGTCCTGTTCATTGTAGGATGGTTTGCAGTATCCCTTGGCCTCTACCCACA
553	Mature_546	bta-miR-22_37785	chr22	13300945	13300963	+	19	62	0	0	0	0	62	0	Precursor_553	chr22_37785|chr22:13300892-13300963:+	72	93	CATGCTCTGTGGTGTGGCC	CTTCCACTGCAGTGGGTGTGGGTTTTATCCCTGGTCAGGGAACTAAGATCCTCCATGCTCTGTGGTGTGGCC
554	Mature_547	bta-miR-22_37792	chr22	13713458	13713480	+	23	35	0	0	0	0	35	0	Precursor_554	chr22_37792|chr22:13713458-13713530:+	73	35	TGCCAAGCACACAGTAGGCATGC	TGCCAAGCACACAGTAGGCATGCAAAAAAAAAAAAAAAAAGACATACCCAAGGCCTAGCTTAGTGCTTGGCCTG
555	Mature_548	bta-miR-22_35663	chr22	14929787	14929804	-	18	996	0	0	0	996	0	0	Precursor_555	chr22_35663|chr22:14929731-14929804:-	74	996	TTCCGATCTGGGGGGCGG	TTCCGATCTGGGGGGCGGGGGGGGATGACTCATATTTGGGGTCAAGTCTTTCTGCCTTTTAAACATTTCATGG
556	Mature_549	bta-miR-22_35091	chr22	15629723	15629742	+	20	57	0	0	0	57	0	0	Precursor_556	chr22_35091|chr22:15629655-15629742:+	88	58	CCCGCAAGCCTCATGGGGAC	CCAGTGGTTAGGACCTGGTGCTTTCACTGCTGTGGACCCGGGTTTGATCCCTGGTCACAGAACGAAGACCCGCAAGCCTCATGGGGAC
557	Mature_550	bta-miR-22_33107	chr22	17040672	17040693	+	22	36	0	36	0	0	0	0	Precursor_557	chr22_33107|chr22:17040626-17040693:+	68	36	CCCACAAGCCTTGGGGTATGAC	CGATCAAGGCCTGGGTTTGATCCTTGGTTAGGGAACTAAGTAAGGTCCCACAAGCCTTGGGGTATGAC
558	Mature_551	bta-miR-22_40267	chr22	17969991	17970008	-	18	28	0	0	28	0	0	0	Precursor_558	chr22_40267|chr22:17969947-17970008:-	62	28	GGAAAGGAAAAAAGAGAG	GGAAAGGAAAAAAGAGAGACATCTTTTGAGTACACTCAGGATGCCTGATGTTTTCTCTCGCT
559	Mature_552	bta-miR-22_38632	chr22	37740559	37740582	-	24	77	0	0	0	0	32	45	Precursor_559	chr22_38632|chr22:37740559-37740610:-	52	77	TACTGACAAGAGGATGCAGTTGCC	CTGTGTGTGCTCTTGGCAGAAAAGAGGATACTGACAAGAGGATGCAGTTGCC
560	Mature_553	bta-miR-22_35913	chr22	41627459	41627479	-	21	96	0	0	0	96	0	0	Precursor_560	chr22_35913|chr22:41627418-41627479:-	62	96	CAGGCTTTAGAACACAGGCTC	CAGGCTTTAGAACACAGGCTCAGTAGCTGTGGTGCATGGGCTTAGTTGCTATATGGTGTGTG
561	Mature_554	bta-miR-22_33341	chr22	43222603	43222625	+	23	25	0	25	0	0	0	0	Precursor_561	chr22_33341|chr22:43222545-43222625:+	81	25	TGGGCATGTCTTTCAAGCCAACC	GAGGAGTGGAAGATGTTGAGGATTACATTGCACAAAGATGGAGACATCTGGGTGCCTCTGGGCATGTCTTTCAAGCCAACC

562	Mature_555	bta-miR-22_33924	chr22	44620853	44620871	-	19	78	0	78	0	0	0	0	Precursor_562	chr22_33924|chr22:44620853-44620919:-	67	78	GAAGGGGAGGGAAAGGGAG	TAAATCCCCCTTTTCTTTGCTTGGCACACTGAAGAACTGGCGGGTGGGGAAGGGGAGGGAAAGGGAG
563	Mature_556	bta-miR-22_34022	chr22	48057218	48057235	+	18	11	11	0	0	0	0	0	Precursor_563	chr22_34022|chr22:48057218-48057302:+	85	11	TGCCCAAGTGGCCCCAGC	TGCCCAAGTGGCCCCAGCATTTGAAGGGTGTTGGTTGGTCACCGGCCATGGCCACTAAATGCCAGGGAGGGGAGGGGAGGGCAGG
564	Mature_557	bta-miR-22_33979	chr22	49926670	49926693	-	24	23	0	23	0	0	0	0	Precursor_564	chr22_33979|chr22:49926641-49926693:-	53	23	CCCTTGCCGTGTCCACTCCTACCC	CCCTTGCCGTGTCCACTCCTACCCATTCCTGGGAGAAGGAAAAGGCGAGGGGA
565	Mature_558	bta-miR-22_33421	chr22	51077139	51077162	+	24	18	0	18	0	0	0	0	Precursor_565	chr22_33421|chr22:51077075-51077162:+	88	30	CCTGGTGCCATGTGGTGCAGCCAG	GGCTTCCCTGGTGGTCCAGTGGTTAAGACTCCAAGCTTCCAATGCAGGGGGCATGGGTTTGATCCCTGGTGCCATGTGGTGCAGCCAG
566	Mature_559	bta-miR-22_34115	chr22	53230033	53230054	+	22	7	7	0	0	0	0	0	Precursor_566	chr22_34115|chr22:53229983-53230054:+	72	7	GGGTTGAGGGGATTCCAAGCTG	GCTTGGTCTCCCAGCAACTCTGTCACCTGATGTGGGATTCTAACCACATGGGGTTGAGGGGATTCCAAGCTG
567	Mature_560	bta-miR-22_40027	chr22	53234079	53234098	+	20	19	0	0	19	0	0	0	Precursor_567	chr22_40027|chr22:53234029-53234098:+	70	19	AGGTCCAGGACACTGAGAAC	CTTCAGTTGTTTGGATCTAACCACATAAGAAGTGGAATTGTTGGGTATTTAGGTCCAGGACACTGAGAAC
568	Mature_561	bta-miR-22_34023	chr22	54705064	54705082	-	19	19	0	19	0	0	0	0	Precursor_568	chr22_34023|chr22:54705012-54705082:-	71	19	GGGGTGGGGGGGCGGCGAT	GGGGTGGGGGGGCGGCGATGCTCTATTGAACTTTCAGGAGAGCCATGAGCAGTCACAGAGCCCCCTTGTAA
569	Mature_562	bta-miR-22_40581	chr22	56357166	56357183	-	18	28	0	0	28	0	0	0	Precursor_569	chr22_40581|chr22:56357106-56357183:-	78	28	AGTAGAGAGGGGAGAGAG	AGTAGAGAGGGGAGAGAGAGATGGCCAATTAATTTCCTCCACCCTGATGGGAAGTGTATTTCCTTCCCTTTCTGCAGC
570	Mature_563	bta-miR-23_38210	chr23	3921670	3921692	-	23	53	0	0	0	0	0	53	Precursor_570	chr23_38210|chr23:3921670-3921740:-	71	53	CGTGCGCGACTGTGCTTTGCCCC	GAGTGATTTTATGTCTTGTGTGCATGTGTGCATATGTGTGTGTGCGCACGTGCGCGACTGTGCTTTGCCCC
571	Mature_564	bta-miR-23_34630	chr23	7744547	7744564	-	18	41	0	41	0	0	0	0	Precursor_571	chr23_34630|chr23:7744547-7744590:-	44	41	GGGGGGAAGGAGGAGGGC	CTTCTTCTGTCTTTCTTTTCTTTTAAGGGGGGAAGGAGGAGGGC
572	Mature_565	bta-miR-23_40699	chr23	8884384	8884403	+	20	18	0	0	18	0	0	0	Precursor_572	chr23_40699|chr23:8884344-8884403:+	60	18	GAAAAGGTCTAGAGAACAGC	ACTTCTAGGGGCCTTACTTACAGAGTATGTGGTGAAAGGTGAAAAGGTCTAGAGAACAGC
573	Mature_566	bta-miR-23_40739	chr23	10125212	10125230	+	19	29	0	0	29	0	0	0	Precursor_573	chr23_40739|chr23:10125177-10125230:+	54	29	AGAGGAAGAGAAGCAGAGG	ACAGTTATCTTCCTTTCAGTGGAAGTGTGCAAGTGAGAGGAAGAGAAGCAGAGG
574	Mature_567	bta-miR-23_34732	chr23	19034059	19034076	-	18	10	0	10	0	0	0	0	Precursor_574	chr23_34732|chr23:19034059-19034101:-	43	10	CCCCAGGCATGAAACCAG	GGATGTGCCTGGGGCCCCCTGGAGGCCCCAGGCATGAAACCAG
575	Mature_568	bta-miR-23_39757	chr23	28525461	28525481	-	21	23	0	0	0	0	23	0	Precursor_575	chr23_39757|chr23:28525424-28525481:-	58	23	GGAGGAAGAAGCGCTCAGGTA	GGAGGAAGAAGCGCTCAGGTAGGGAAGGGAGGGAGGGATCTGAGTTTTCTTGTCTCAG
576	Mature_569	bta-miR-23_35425	chr23	28664841	28664861	-	21	59	13	0	46	0	0	0	Precursor_576	chr23_35425|chr23:28664787-28664861:-	75	59	GGATGAAGAAGCGCTCAGGTA	GGATGAAGAAGCGCTCAGGTAGGGAAGGGAGGGAGGGATCTGAGTTTTCTTGTCTCACTGGGGGTTTCAAGCCAG
577	Mature_570	bta-miR-23_34359	chr23	31224304	31224327	+	24	128	0	24	0	0	0	104	Precursor_577	chr23_34359|chr23:31224262-31224327:+	66	128	TAGACTCCCACAAGCTGTGCAGCC	TTGCATTGCAGGGGGCCTAGGTTTGATCCCTGGTAAGGGAACTAGACTCCCACAAGCTGTGCAGCC
578	Mature_571	bta-miR-23_34891	chr23	31725110	31725128	-	19	10	0	10	0	0	0	0	Precursor_578	chr23_34891|chr23:31725074-31725128:-	55	10	AACAACGGCCCTTTTCAGG	AACAACGGCCCTTTTCAGGGCCACCCACTTGGTCATAAGAAGGGCTGTTACTTTT
579	Mature_572	bta-miR-23_41122	chr23	36641608	36641625	+	18	15	0	0	15	0	0	0	Precursor_579	chr23_41122|chr23:36641608-36641661:+	54	15	GGGGAGAAAAGGAAGGAG	GGGGAGAAAAGGAAGGAGGGTGGGAAGGACCAAACTAATCTTCATTCTTCCAGC
580	Mature_573	bta-miR-23_41861	chr23	49042818	49042835	-	18	21	0	0	21	0	0	0	Precursor_580	chr23_41861|chr23:49042777-49042835:-	59	21	GAGCTCCAGGTGGAAATG	GAGCTCCAGGTGGAAATGCAGCCCCACCTGGGCCTGTGGGATCCTCCCTGGAGAATCCG
581	Mature_574	bta-miR-24_41968	chr24	11036038	11036056	+	19	24	0	0	24	0	0	0	Precursor_581	chr24_41968|chr24:11036038-11036100:+	63	24	GAGAAGAGGAGAGAAAGGC	GAGAAGAGGAGAGAAAGGCCCAGTGAGAGGGAAATCACAGGTTTTTTTGTCCCTAATCTCAG
582	Mature_575	bta-miR-24_42102	chr24	30238697	30238715	+	19	18	0	0	18	0	0	0	Precursor_582	chr24_42102|chr24:30238697-30238757:+	61	18	GAGAAGAGGGGAAAAGGGA	GAGAAGAGGGGAAAAGGGAAATAAATTTCAAATTTGTTTCCCTTTCTTTCCCTCTGCCCCG
583	Mature_576	bta-miR-24_42646	chr24	33070879	33070897	-	19	14	0	0	14	0	0	0	Precursor_583	chr24_42646|chr24:33070879-33070924:-	46	14	ATGGTAATGTTGTAGAGGG	CTCATGGATTACTCCTTCCCGTAGGGAATGGTAATGTTGTAGAGGG
584	Mature_577	bta-miR-24_42194	chr24	37434263	37434280	+	18	20	0	0	20	0	0	0	Precursor_584	chr24_42194|chr24:37434263-37434342:+	80	20	AAGCATGGAGCGAAAGAC	AAGCATGGAGCGAAAGACAAAGATGACTGAAGGAACTTGAGCTAAAACTTTCCAGTTACTTTGTCTTACTCTATACTGTT
585	Mature_578	bta-miR-24_42701	chr24	37544967	37544986	-	20	46	0	0	46	0	0	0	Precursor_585	chr24_42701|chr24:37544937-37544986:-	50	46	GGAAGAGAGGGGAAGAGAGG	GGAAGAGAGGGGAAGAGAGGCACTGTCATAAATCTCTACCTGCCTTTTAA
586	Mature_579	bta-miR-24_36339	chr24	38776620	38776637	-	18	69	69	0	0	0	0	0	Precursor_586	chr24_36339|chr24:38776589-38776637:-	49	69	GGGGGTGTAGCTCAGTAG	GGGGGTGTAGCTCAGTAGTAGAGCGCATGCTCTATGGGGCTTCCCCCAT
587	Mature_580	bta-miR-24_40407	chr24	40791948	40791965	+	18	36	0	0	0	0	36	0	Precursor_587	chr24_40407|chr24:40791925-40791965:+	41	36	AGGGGAAGGGGGGAAAAT	AGTCCTCACCTTTGCTAAACAAAAGGGGAAGGGGGGAAAAT
588	Mature_581	bta-miR-24_39666	chr24	41909616	41909640	-	25	91	0	0	0	0	0	91	Precursor_588	chr24_39666|chr24:41909616-41909677:-	62	91	CAGCCCTCCAGACAGCCACACTGCT	CAGACCCGTGCAGTCAAGGGCCCACCAAACCTGCACACAGCCCTCCAGACAGCCACACTGCT
589	Mature_582	bta-miR-24_40424	chr24	42080726	42080750	+	25	25	0	0	0	0	25	0	Precursor_589	chr24_40424|chr24:42080726-42080800:+	75	26	GATCCACTGCACTCAGTGTCTTAGC	GATCCACTGCACTCAGTGTCTTAGCACAAAAAAGAGAAGAGAGGATGGGAGAGGACGGGGTGGGGTGGGGGAGGG
590	Mature_583	bta-miR-24_35419	chr24	44168548	44168570	+	23	20	0	20	0	0	0	0	Precursor_590	chr24_35419|chr24:44168524-44168570:+	47	20	TGTATCTCATACAAACCACCAGT	AGGTGCTGTGCTAGGTACATTGCATGTATCTCATACAAACCACCAGT
591															Precursor_591	chr24_38029|chr24:47126221-47126307:-	87	177		CATGCCTCGAGGTGCAGCCAAAACAAACAGTCTAGGAAAGATCAAAGTGTGAGCAAAGGACTGGGGGTGGGAAGCACGGGGCATTTA
592	Mature_584	bta-miR-24_38030	chr24	47126289	47126307	-	19	177	0	0	0	96	81	0	Precursor_592	chr24_38030|chr24:47126289-47126360:-	72	177	CATGCCTCGAGGTGCAGCC	CTTCCACTGCGGGGGGTGTGGGTTTGATACCCGATTGGGAAACCAAGATTCCACATGCCTCGAGGTGCAGCC
593	Mature_585	bta-miR-24_35949	chr24	52103102	52103124	-	23	14	0	14	0	0	0	0	Precursor_593	chr24_35949|chr24:52103078-52103124:-	47	17	TCTCTTTCACAGCATTTTGTTTC	TCTCTTTCACAGCATTTTGTTTCAGAAAAAAGGAAAAGAGGGAGAAG
594	Mature_586	bta-miR-24_35957	chr24	53613504	53613524	-	21	11	0	11	0	0	0	0	Precursor_594	chr24_35957|chr24:53613452-53613524:-	73	11	ACTGGGGTGAGGGAGTGGAGG	ACTGGGGTGAGGGAGTGGAGGGAGAAACCTCGGTGCATTTTTTTCCTCTCCTTTCTCTTCCTCCTCCCACTTA
595	Mature_587	bta-miR-24_42865	chr24	56623287	56623305	-	19	67	0	0	67	0	0	0	Precursor_595	chr24_42865|chr24:56623247-56623305:-	59	67	GGAAGAGAGGAAGAGAGAG	GGAAGAGAGGAAGAGAGAGATGATATTTGCAAACCATTTTTTTCTGTTTTCATTTTGAA
596	Mature_588	bta-miR-24_42433	chr24	60730537	60730561	+	25	15	0	0	15	0	0	0	Precursor_596	chr24_42433|chr24:60730512-60730561:+	50	15	ATCCCACACGCCTCACGATGCAGCC	TTGGATCACAGGTAGGGGAACTAAGATCCCACACGCCTCACGATGCAGCC
597	Mature_589	bta-miR-24_36017	chr24	61489674	61489693	-	20	10	0	10	0	0	0	0	Precursor_597	chr24_36017|chr24:61489621-61489693:-	73	11	GTAGGAACATACACGGTAGG	GTAGGAACATACACGGTAGGCAAAGTCATACGGAAACCGATGGACTTTTTGGCCAACCCAGTGTGTCCTACGT
598	Mature_590	bta-miR-25_43458	chr25	2705448	2705466	-	19	1234	0	0	1234	0	0	0	Precursor_598	chr25_43458|chr25:2705448-2705485:-	38	1234	CGTTGGCTGGGTCCCCAGG	CAGGGACTCACCAGCGGGTCGTTGGCTGGGTCCCCAGG
599	Mature_591	bta-miR-25_36985	chr25	2705770	2705794	-	25	110	39	0	71	0	0	0	Precursor_599	chr25_36985|chr25:2705736-2705789:-	63	110	GGTCTGGAAGGTGACAAGCAGCGAC	GGTCTGGAAGGTGACAAGCAGCGACAAAGTGCTGATGGGGCTGGCTGCCCACCCTCCAGCCAG
600	Mature_592	bta-miR-25_36987	chr25	2979304	2979321	-	18	60	60	0	0	0	0	0	Precursor_600	chr25_36987|chr25:2979263-2979321:-	59	60	GTGAAAACTTAAATGGAC	GTGAAAACTTAAATGGACCACATGTGCTTTATCACCATGGAGATCCTGGGGAATCACCT
601	Mature_593	bta-miR-25_43483	chr25	3483713	3483730	-	18	1319	0	0	1319	0	0	0	Precursor_601	chr25_43483|chr25:3483662-3483730:-	69	1319	CGGAGGAGGGGGAGGAGG	CGGAGGAGGGGGAGGAGGAGGGGCGCGGGGGGCGGCCTCCCCCCCGCCTCCCTCCCCCGCTGCTTCCCT
602	Mature_594	bta-miR-25_36470	chr25	4327161	4327180	-	20	12	0	12	0	0	0	0	Precursor_602	chr25_36470|chr25:4327161-4327228:-	68	12	CTCACCTCTCTTCCCTGAGC	GCAGGGTCAAGGGTGGCCATAACAAATAGCCCATTAAATGGGATGGTACTCACCTCTCTTCCCTGAGC
603	Mature_595	bta-miR-25_36525	chr25	8204111	8204130	-	20	12	0	12	0	0	0	0	Precursor_603	chr25_36525|chr25:8204046-8204130:-	85	12	CAGGGCTGCAGGCAGGGAGG	CAGGGCTGCAGGCAGGGAGGAAAAAAAAAAAAATCACAGACTATCTCAGATTAAGTTTGCAATCAGCAAGTGCAAAGGCCCTGGG
604	Mature_596	bta-miR-25_39966	chr25	8879066	8879087	+	22	31	0	0	0	0	0	31	Precursor_604	chr25_39966|chr25:8879066-8879133:+	68	31	CATTTATGGATCCCAAACGGTT	CATTTATGGATCCCAAACGGTTAAAAAAAAAAAAAAACCTCTCCTCGTCTGTTAAGGATTCAGGTGTC
605	Mature_597	bta-miR-25_43540	chr25	10574228	10574247	-	20	18	0	0	18	0	0	0	Precursor_605	chr25_43540|chr25:10574228-10574303:-	76	18	AGAAATATGAAAGAGAAGCT	CTTTTCTTTCAACATTTTCAGGTATTTGACTGGAATGGTTGACAAAAGGACACTTGAGAAATATGAAAGAGAAGCT
606	Mature_598	bta-miR-25_36668	chr25	14480865	14480883	+	19	10	10	0	0	0	0	0	Precursor_606	chr25_36668|chr25:14480865-14480940:+	76	10	AGAGCAGCGGCCCTCTGGC	AGAGCAGCGGCCCTCTGGCCCTTTTCCCTGGAACTGGCCATGAGGAAATGGGAGAGACGCTGGGATGCAGCTCCCC
607	Mature_599	bta-miR-25_36167	chr25	16841785	16841807	+	23	34	0	34	0	0	0	0	Precursor_607	chr25_36167|chr25:16841738-16841807:+	70	34	CCCGCGTGCTGGGTGGTGGAGCC	TTCCATTGCAGGGTCACGAGTTTAATCTCTGGTCTGAGATCTAAAGTCCCGCGTGCTGGGTGGTGGAGCC
608	Mature_600	bta-miR-25_38359	chr25	17549927	17549950	+	24	37	0	0	0	37	0	0	Precursor_608	chr25_38359|chr25:17549868-17549950:+	83	38	CAGGAGTCTGTCTAAAACCATCCC	GAGTAGATCTAGTAGACAGAGTCCCTGAAGAACTATGGACAGAGGTTCATAATATTGTACAGGAGTCTGTCTAAAACCATCCC
609	Mature_601	bta-miR-25_38852	chr25	23630137	23630155	-	19	82	0	0	0	82	0	0	Precursor_609	chr25_38852|chr25:23630137-23630221:-	85	82	CATGCTGTGTGGCACTGCC	TTAAAACTACACACTTCCGCTGCAGGGGGCATGGATTAGAGCCCTGGTCAGGGAACTAAGCTCCCTCATGCTGTGTGGCACTGCC
610	Mature_602	bta-miR-25_36677	chr25	23985477	23985497	-	21	27	0	27	0	0	0	0	Precursor_610	chr25_36677|chr25:23985439-23985497:-	59	27	CTCCCCATGTTTTTCCACTGC	CTCCCCATGTTTTTCCACTGCAAAAAAAAAAAAAAAAAGCAGAATGATAGATGGGAGAA
611	Mature_603	bta-miR-25_43187	chr25	24924380	24924397	+	18	15	0	0	15	0	0	0	Precursor_611	chr25_43187|chr25:24924355-24924397:+	43	15	GGAAGAAGCAGAGAGAGC	TCTGGCTTCCCCACCCCCCAGGGAAGGAAGAAGCAGAGAGAGC
612	Mature_604	bta-miR-25_37166	chr25	25720001	25720021	-	21	15	15	0	0	0	0	0	Precursor_612	chr25_37166|chr25:25720001-25720087:-	87	15	GGACTCCAGATGCAAGGGACG	TCTTTGGATTTGGAGTTAGGAGTGGAGAGGAAGACTTGACCAAGAGAGTCATCACTTATAACAGATGGACTCCAGATGCAAGGGACG
613	Mature_605	bta-miR-25_36696	chr25	26026192	26026211	-	20	204	0	30	174	0	0	0	Precursor_613	chr25_36696|chr25:26026192-26026234:-	43	204	GGGAAGATAAAGAAGGGAAG	TCCCTGTTCTTTCCTTCAGCTATGGGAAGATAAAGAAGGGAAG
614	Mature_606	bta-miR-25_38883	chr25	26919572	26919589	-	18	76	0	0	0	11	28	37	Precursor_614	chr25_38883|chr25:26919527-26919589:-	63	76	GGGGCCGGCGGGGGACCG	GGGGCCGGCGGGGGACCGGGAGGCTGGCGCTTCCAGGAAAAACGGCCCCCCGTCCCGCGCCTC
615	Mature_607	bta-miR-25_38927	chr25	31484529	31484551	-	23	30	0	0	0	30	0	0	Precursor_615	chr25_38927|chr25:31484484-31484551:-	68	30	CTGCCTCCCTGAGGCCTCCCCCC	CTGCCTCCCTGAGGCCTCCCCCCAAAAGAAAAGCCTGAGATTGGCACTGGGGGGTTTGATTGGTAGGG
616	Mature_608	bta-miR-25_36779	chr25	33443757	33443774	-	18	124	0	28	96	0	0	0	Precursor_616	chr25_36779|chr25:33443723-33443774:-	52	124	GGAGAAAGGGGAGAAAGG	GGAGAAAGGGGAGAAAGGCGTGAACCTGCCCATGGCTTTTCCTCGTCGCCAC
617	Mature_609	bta-miR-25_36806	chr25	33980048	33980065	+	18	117	35	0	82	0	0	0	Precursor_617	chr25_36806|chr25:33980048-33980094:+	48	137	AGGAAGGAGAAGGAGGGC	AGGAAGGAGAAGGAGGGCGTGGCAGCCCACTCCAGTATTCTTGCCTGG
618	Mature_610	bta-miR-25_43317	chr25	35827320	35827337	+	18	13	0	0	13	0	0	0	Precursor_618	chr25_43317|chr25:35827287-35827337:+	51	13	GGGGTGTCGGGGGGGGGG	TGCCCCCCAAGTACCCCCAAACTGTGTCCATGAGGGGTGTCGGGGGGGGGG
619	Mature_611	bta-miR-25_43826	chr25	36143006	36143023	-	18	106	0	0	106	0	0	0	Precursor_619	chr25_43826|chr25:36143006-36143075:-	70	106	GAAGAGAGAGAAAGAAGG	ATCAGTCTCCCTTTTGGTTCCTGGGGAGGGAGGGCCTGCCCTGTGAAACACAGAAGAGAGAGAAAGAAGG
620	Mature_612	bta-miR-25_43330	chr25	36341730	36341750	+	21	13	0	0	13	0	0	0	Precursor_620	chr25_43330|chr25:36341730-36341780:+	51	13	GGGGCTGCTCCAGCTGACGGC	GGGGCTGCTCCAGCTGACGGCAAAGGTCAGTACAGCTGGGCCAGGCCTCTT
621	Mature_613	bta-miR-25_43380	chr25	38444179	38444203	+	25	14	0	0	14	0	0	0	Precursor_621	chr25_43380|chr25:38444179-38444259:+	81	14	GAGCAGCAGAAGCAGCTGCAGCGGC	GAGCAGCAGAAGCAGCTGCAGCGGCAGGAACAATCAGAGGAGAGGCAAGTGCAAGAACAGCAGCTGCCACAGCTGCAGCAG
622	Mature_614	bta-miR-25_36911	chr25	39706265	39706282	+	18	20	20	0	0	0	0	0	Precursor_622	chr25_36911|chr25:39706210-39706282:+	73	20	GGAGAGGGAAAGGAGAAG	CAATCTTTCCCTCCTCTGTCCCTGGGTCTCCAGCCTAGGAGGGCTGGGCTCGCTGGGAGAGGGAAAGGAGAAG
623	Mature_615	bta-miR-25_36394	chr25	40858929	40858950	+	22	10	0	10	0	0	0	0	Precursor_623	chr25_36394|chr25:40858929-40858997:+	69	10	CCCAGGAGTAGCGGCATGGCCG	CCCAGGAGTAGCGGCATGGCCGAAAAAAAAAAAAAAGAAGAAGACAGCCTGTGTGGCCATTTAACGGGGA
624	Mature_616	bta-miR-26_37350	chr26	2116024	2116041	+	18	17	17	0	0	0	0	0	Precursor_624	chr26_37350|chr26:2116024-2116069:+	46	17	GAGGAAGAAGAAAGGAGG	GAGGAAGAAGAAAGGAGGTTATTCTCTAATCACTCCTTTCTCTGGT
625	Mature_617	bta-miR-26_39100	chr26	9285643	9285660	+	18	16	0	0	0	16	0	0	Precursor_625	chr26_39100|chr26:9285643-9285687:+	45	16	AGGGTGTGTAAGTGGGGG	AGGGTGTGTAAGTGGGGGTGGCTACAGGGCTCCCTTTTAGCCTGG
626	Mature_618	bta-miR-26_37709	chr26	10725968	10725987	-	20	132	48	0	84	0	0	0	Precursor_626	chr26_37709|chr26:10725907-10725987:-	81	134	GAGAAGAAGGGGAAGAGGAG	GAGAAGAAGGGGAAGAGGAGAAAGCCGAGAAAATGAGGCGAGCAGGAAGGAAAGGAAAGGCTCTCCCTTCCTGGTTCGGTG
627	Mature_619	bta-miR-26_36973	chr26	13779219	13779237	+	19	16	0	16	0	0	0	0	Precursor_627	chr26_36973|chr26:13779151-13779237:+	87	16	GGGGGCCGGGCGAGGGGCC	CCCTGCCGCGCCCGGCCTCGGGGCTCTTCGGAGCGGAGCCCGGCGTGCCGGGAGGAGTTCTACGGCCTGGGGGCCGGGCGAGGGGCC
628	Mature_620	bta-miR-26_39565	chr26	15789279	15789297	-	19	26	0	0	0	26	0	0	Precursor_628	chr26_39565|chr26:15789238-15789297:-	60	26	CTCACTTCTCAATGACCCC	CTCACTTCTCAATGACCCCATAAGTAACGAATGTTAGGAGGTAATTGGAGGAGTTGAGGG
629	Mature_621	bta-miR-26_44472	chr26	17976403	17976420	-	18	13	0	0	13	0	0	0	Precursor_629	chr26_44472|chr26:17976403-17976449:-	47	13	GGTTGTCATATTCTCTTG	TGAGAAAAGTGACAACCAAAATTTGGATTGGTTGTCATATTCTCTTG
630	Mature_622	bta-miR-26_44516	chr26	21713135	21713154	-	20	55	0	0	55	0	0	0	Precursor_630	chr26_44516|chr26:21713135-21713181:-	47	55	GGAGAAGAAGAAGAGAGGAG	CCTCGCAACCAGCTTCTCCCCAGAAGGGGAGAAGAAGAAGAGAGGAG
631	Mature_623	bta-miR-26_44114	chr26	23026846	23026865	+	20	10	0	0	10	0	0	0	Precursor_631	chr26_44114|chr26:23026846-23026920:+	75	10	GGCCGGCAGGGAGGGACTGG	GGCCGGCAGGGAGGGACTGGAGTGAGGAGGGGGGAGTCCATCCTCCGTGCCGCCGGCCTGGGCCACTGCTGCCTC
632	Mature_624	bta-miR-26_37061	chr26	24513244	24513266	+	23	28	0	28	0	0	0	0	Precursor_632	chr26_37061|chr26:24513195-24513266:+	72	28	CCTACATGCCTTGCAGTGCGGCC	CTTCCAATGCATGGGGTGTGGATTTGATTCCTGGTCAGGGAACTAAGATCCTACATGCCTTGCAGTGCGGCC
633	Mature_625	bta-miR-26_39738	chr26	33495929	33495952	-	24	37	0	0	0	37	0	0	Precursor_633	chr26_39738|chr26:33495929-33495982:-	54	37	CTCATTCATTGTTTTACACCTCCC	GAGGTGGAAGGGCCGGAGAGTGTAAACCCACTCATTCATTGTTTTACACCTCCC
634	Mature_626	bta-miR-26_44200	chr26	33561824	33561843	+	20	22	0	0	22	0	0	0	Precursor_634	chr26_44200|chr26:33561824-33561876:+	53	22	AGCCAAGGGAAGGGGCTTGG	AGCCAAGGGAAGGGGCTTGGAGAACTTTTCCTCAAGCTATGTCTTGGGGCCTC
635	Mature_627	bta-miR-26_37203	chr26	40768522	40768542	+	21	23	0	23	0	0	0	0	Precursor_635	chr26_37203|chr26:40768459-40768542:+	84	23	AAGCTCTACAGAACAAGACCG	GTCTTGGATGGAAATAGTTTTTGCTTTCATTCTGTGGTGGAGTGCAGGGAGGAAGTGTGAAAAAAGCTCTACAGAACAAGACCG
636	Mature_628	bta-miR-26_37963	chr26	43005740	43005758	-	19	116	57	0	59	0	0	0	Precursor_636	chr26_37963|chr26:43005699-43005757:-	61	116	AGGGAGGAGGGGGAAGAAG	AGGGAGGAGGGGGAAGAAGGGGGCGGCGTGAGCACATCCCCATCCTGCTCCTCACCCCAGC
637	Mature_629	bta-miR-27_37786	chr27	5600959	5600977	+	19	11	0	11	0	0	0	0	Precursor_637	chr27_37786|chr27:5600959-5601036:+	78	11	CTAATGGCAGAGAAAATGC	CTAATGGCAGAGAAAATGCTTGCAGGAGCCCAAAGGAAACAGTACATCTTTGGAATTTTGAGTCTTTTCTACCAAGAT
638	Mature_630	bta-miR-27_38369	chr27	6222303	6222320	-	18	6	6	0	0	0	0	0	Precursor_638	chr27_38369|chr27:6222258-6222320:-	63	9	GGGGGCCCGCGGTGGTGG	GGGGGCCCGCGGTGGTGGCGGGGGTGGGGTCGCGCCCTCCGGTCCGCCGTCGTTCGCCGTCCC
639	Mature_631	bta-miR-27_38386	chr27	6224016	6224038	-	23	8	8	0	0	0	0	0	Precursor_639	chr27_38386|chr27:6223985-6224038:-	54	14	GCGGCGTGGCGGGCGGCGGCTCC	GCGGCGTGGCGGGCGGCGGCTCCCCGTCGCGGGGCCGCTTGCCTTGCCCCGCCG
640	Mature_632	bta-miR-27_40534	chr27	13490641	13490659	-	19	60	0	0	0	24	0	36	Precursor_640	chr27_40534|chr27:13490641-13490716:-	76	76	CATGCCATGGGGTACAGGC	CTGTACTTCCACTGTGGGGGCATAGGTTCAATCCCTGGTTGGGGAACTAAAACCCCACATGCCATGGGGTACAGGC
641	Mature_633	bta-miR-27_40586	chr27	19820225	19820246	-	22	38	0	0	0	38	0	0	Precursor_641	chr27_40586|chr27:19820225-19820279:-	55	38	GGGTCCTTGAAATGCAATGTGC	ACAGCCTCCTCAGTGGTCCTGATTCTTAAAAGAGGGTCCTTGAAATGCAATGTGC
642	Mature_634	bta-miR-27_43452	chr27	28794080	28794100	+	21	28	0	0	0	0	28	0	Precursor_642	chr27_43452|chr27:28794080-28794145:+	66	28	TCCCTGAGGGTTCCCCTCCCC	TCCCTGAGGGTTCCCCTCCCCTAGAAAAGAAAAAGAGTGAGGGGAGGGTACGAACCCTGATTTCAC
643	Mature_635	bta-miR-27_38182	chr27	32780270	32780288	+	19	32	32	0	0	0	0	0	Precursor_643	chr27_38182|chr27:32780270-32780339:+	70	39	GAAAGAGAAGGAGGAAAGG	GAAAGAGAAGGAGGAAAGGGTGGAGAAAAAGAGGAAGGAACAATTTCCCACCTTCTCCTTCTTTCTCTT
644	Mature_636	bta-miR-27_40255	chr27	34246164	34246186	+	23	79	0	0	0	79	0	0	Precursor_644	chr27_40255|chr27:34246101-34246186:+	86	79	CGGTCGAAGGGTTTAGTGTCCTC	GGGCGCTGAGTACCCAGCTGTTGCGTAGGTAGTCTCGCGAGAACGCGGCTCACCGAAGGCCAACGGTCGAAGGGTTTAGTGTCCTC
645	Mature_637	bta-miR-27_43555	chr27	35471178	35471201	+	24	92	0	0	0	0	92	0	Precursor_645	chr27_43555|chr27:35471130-35471201:+	72	92	TCCCACAAGCCAAGCGGTGAGGCC	CTTTCAATGCCGTGGCTTGGGTTTTGATCCTCGGTCAGGGAATTGAGATCCCACAAGCCAAGCGGTGAGGCC
646	Mature_638	bta-miR-27_40797	chr27	43738518	43738535	-	18	30	0	0	0	30	0	0	Precursor_646	chr27_40797|chr27:43738487-43738535:-	49	30	CTGGGCTGGGGAGAAATG	CTGGGCTGGGGAGAAATGGTTAGAACCAAGCATCTCCACAGGCTCAGGA
647	Mature_639	bta-miR-28_46062	chr28	7400558	7400575	-	18	31	0	0	31	0	0	0	Precursor_647	chr28_46062|chr28:7400558-7400600:-	43	31	GGAGGCCAACTGGTGGAG	TTACTGCTGGCCCCTTTCTCAGGCTGGAGGCCAACTGGTGGAG
648	Mature_640	bta-miR-28_38781	chr28	9363255	9363274	+	20	17	17	0	0	0	0	0	Precursor_648	chr28_38781|chr28:9363223-9363274:+	52	17	AGAAGAGGGAAGGGAGAAAG	GGCTGCCTTCTCTCTGTGTCCTCACATGGCAGAGAAGAGGGAAGGGAGAAAG
649	Mature_641	bta-miR-28_44694	chr28	10794856	10794878	-	23	20	0	0	0	0	20	0	Precursor_649	chr28_44694|chr28:10794856-10794925:-	70	20	CTCCAGTGAACTTGTATCTTCCC	TTGGATGTATCTTCATTGGATATCTAACTATTTATGGTTTCAGGACACTCCAGTGAACTTGTATCTTCCC
650	Mature_642	bta-miR-28_39216	chr28	16385239	16385257	-	19	11	11	0	0	0	0	0	Precursor_650	chr28_39216|chr28:16385239-16385281:-	43	11	GAGAAAAGAGAGGAAGGAG	CCTTCCATCTTTGTAGCCGCATGGGAGAAAAGAGAGGAAGGAG
651	Mature_643	bta-miR-28_45737	chr28	19004769	19004786	+	18	17	0	0	17	0	0	0	Precursor_651	chr28_45737|chr28:19004745-19004786:+	42	17	AGATCATGACAACCCACC	TGGGTTGGGAAGATCTCCTGGAGGAGATCATGACAACCCACC
652	Mature_644	bta-miR-28_39254	chr28	20657114	20657132	-	19	24	24	0	0	0	0	0	Precursor_652	chr28_39254|chr28:20657114-20657197:-	84	24	GGGCAGAGGGAAGAGGAGG	TTCTTTTCCCTAGAATTAACACAGCTTTTCAGTGATCTCACAAGGACCCTACTGTGAGAAGAGCTGGGCAGAGGGAAGAGGAGG
653	Mature_645	bta-miR-28_45810	chr28	25550147	25550170	+	24	34	0	0	34	0	0	0	Precursor_653	chr28_45810|chr28:25550147-25550214:+	68	34	CCTGAAATGTGAACTGAATGGGAG	CCTGAAATGTGAACTGAATGGGAGAAAAAAAAGAAAAAAAACCTCCTATATCCGTTGAGATTTAGGTT
654	Mature_646	bta-miR-28_45854	chr28	29682752	29682770	+	19	16	0	0	16	0	0	0	Precursor_654	chr28_45854|chr28:29682752-29682800:+	49	16	GGGGACCCGGCTCCAGGGG	GGGGACCCGGCTCCAGGGGGTGCGGGCCCCATGGAGCTGATGTTCGCCG
655	Mature_647	bta-miR-28_39156	chr28	30081049	30081071	-	23	20	0	20	0	0	0	0	Precursor_655	chr28_39156|chr28:30081049-30081105:-	57	20	CCTCCAAGGTGTGCACTTTGGCC	TCTGGGTTCAATCTTTGGTTGGGGGACTAAGATCCCTCCAAGGTGTGCACTTTGGCC

656	Mature_648	bta-miR-28_38819	chr28	31898690	31898708	+	19	8	0	8	0	0	0	0	Precursor_656	chr28_38819|chr28:31898638-31898708:+	71	8	GAGAGGGGAGAAAAGAAAG	TGGGTTTCTGGTCTCTCTCTTTCTCTTAGCCTATTCTGTGGATTGGGAGGCAGAGAGGGGAGAAAAGAAAG
657	Mature_649	bta-miR-28_45930	chr28	34535662	34535679	+	18	24	0	0	24	0	0	0	Precursor_657	chr28_45930|chr28:34535662-34535705:+	44	24	GAGGGAGAAAAGAGAAGG	GAGGGAGAAAAGAGAAGGGACCCCCCTCTGGTAAGTTCCTTCAA
658	Mature_650	bta-miR-28_41137	chr28	37468445	37468469	+	25	173	0	0	0	173	0	0	Precursor_658	chr28_41137|chr28:37468384-37468469:+	86	173	GAGCCAGGTTATATAGAAGTTGTGC	ACAGCGTCTCAAATAACCCTGCAGTGCTTCTCAGAGAACTGCTCTGAAGAGGCAAAGAGAAGAGCCAGGTTATATAGAAGTTGTGC
659	Mature_651	bta-miR-28_39405	chr28	44737440	44737458	-	19	26	26	0	0	0	0	0	Precursor_659	chr28_39405|chr28:44737414-44737458:-	45	26	GGAAGAAGAGAAAAGGAAG	GGAAGAAGAGAAAAGGAAGATGTTTCTGCCTTGCTTCTGCTCCAC
660	Mature_652	bta-miR-28_39411	chr28	45676166	45676183	-	18	17	17	0	0	0	0	0	Precursor_660	chr28_39411|chr28:45676116-45676183:-	68	17	GGGAAGAAGAGAGGGAAG	GGGAAGAAGAGAGGGAAGAAAAGGACACGTGCTAGTGTCTCTCCCAAGCTTTCTCAGTTAGGTTTCCA
661	Mature_653	bta-miR-29_39704	chr29	21870	21892	-	23	11	0	11	0	0	0	0	Precursor_661	chr29_39704|chr29:21870-21923:-	54	11	CGCACGACCCGCGACCGGACTCC	CCTCTCGTCGCGGGGCGTGCTGTACTTGAGCCGCACGACCCGCGACCGGACTCC
662	Mature_654	bta-miR-29_39305	chr29	5532307	5532328	+	22	14	0	14	0	0	0	0	Precursor_662	chr29_39305|chr29:5532254-5532328:+	75	14	CATCCAGAGAACACAGAACTCC	GTGTTCTGAGTTGGCTGGACCATGGACAGCTCCGAAAGTTACAGCTGCTGACCCATCCAGAGAACACAGAACTCC
663	Mature_655	bta-miR-29_39770	chr29	8936439	8936458	-	20	11	0	11	0	0	0	0	Precursor_663	chr29_39770|chr29:8936439-8936502:-	64	11	CCCTGCAAGCAGCAGTGCAG	TCACTGCTGAGGGTGCAGGTTAATCCCTGGTTGGGGATCTAAGACCCTGCAAGCAGCAGTGCAG
664	Mature_656	bta-miR-29_45646	chr29	25038972	25038990	-	19	132	0	0	0	0	132	0	Precursor_664	chr29_45646|chr29:25038972-25039046:-	75	132	CATGCCGTGTGGTGTGGTC	CCATTTCCACTGCAGGGCGCACGGGTTCCATCCCTAGCCAGGGAAAAAGGATCCCGCATGCCGTGTGGTGTGGTC
665	Mature_657	bta-miR-29_44084	chr29	28898278	28898302	-	25	127	0	0	0	0	0	127	Precursor_665	chr29_44084|chr29:28898278-28898371:-	94	127	GATTCCCAAGCTTCATGGTGCAGCC	CTCGCCATCCAGTGGTTAAGGCTTCACGCTTCCCCTGCAGGGGACTCTGATCCCTCAAGGGGGAACTAAGATTCCCAAGCTTCATGGTGCAGCC
666	Mature_658	bta-miR-29_42210	chr29	31455483	31455506	-	24	24	0	0	0	24	0	0	Precursor_666	chr29_42210|chr29:31455483-31455574:-	92	24	CTCTCATTGGCCTTCGCTCGCCAC	GGTGAGAACAGGGCTCATGACAGGCCCACAAGGTCCAGACCTGAGACTAGATGTGGTTGGTTGAGTCACTCTCATTGGCCTTCGCTCGCCAC
667	Mature_659	bta-miR-29_42272	chr29	38255325	38255344	-	20	119	0	0	0	119	0	0	Precursor_667	chr29_42272|chr29:38255325-38255392:-	68	261	TCCCAAAGAAAAAGGAATGC	TTTGTTTGTTTGTTTGTTTTTTGATCTGTACATACCACATCAAGATCATCCCAAAGAAAAAGGAATGC
668	Mature_660	bta-miR-29_47097	chr29	38356765	38356782	-	18	16	0	0	16	0	0	0	Precursor_668	chr29_47097|chr29:38356703-38356782:-	80	16	GTGGTCAAGTGGTTAAGG	GTGGTCAAGTGGTTAAGGATCTGCTTTGCAATGCAAAGTATTTGCAAATGGCAAAGCTTTGTTCCTTTAGCAGAGAACTA
669	Mature_661	bta-miR-29_45788	chr29	39051372	39051392	-	21	218	0	0	0	0	218	0	Precursor_669	chr29_45788|chr29:39051372-39051437:-	66	219	TCCACAGTTGTTTTGGAGCCC	CTTCCAAGGAGCAAGTGTGTTTTAATTTTGTGGCTGCAGCCAAGGTCCACAGTTGTTTTGGAGCCC
670	Mature_662	bta-miR-29_39559	chr29	39225356	39225378	+	23	16	0	16	0	0	0	0	Precursor_670	chr29_39559|chr29:39225287-39225378:+	92	16	GAAGGATGAGAGGGAGTCGCTAG	AGTGTCACCTTTAATTTGTCTTTGAAGGATCTAATACAAGGGCTTGGAGACACCAGCTGGGTGTTAGTTGAAGGATGAGAGGGAGTCGCTAG
671	Mature_663	bta-miR-29_39565	chr29	39805604	39805621	+	18	33	0	33	0	0	0	0	Precursor_671	chr29_39565|chr29:39805547-39805621:+	75	33	AAGGAGGGGAAGGGGGTG	CTCCAGTTTGCCTCTGCAAGGAGAAATGTGTCTGGAAAGTACTTCAACCTTCTGAAAAAGGAGGGGAAGGGGGTG
672	Mature_664	bta-miR-29_39988	chr29	40268653	40268674	-	22	19	0	19	0	0	0	0	Precursor_672	chr29_39988|chr29:40268653-40268716:-	64	19	TCCCACATGCCACATAGTGCAG	GCAGGGGTGGAGTGGGTTCAATCCCAGGTCAGGGGACTAAGATCCCACATGCCACATAGTGCAG
673	Mature_665	bta-miR-29_40110	chr29	40745637	40745654	-	18	13	13	0	0	0	0	0	Precursor_673	chr29_40110|chr29:40745637-40745698:-	62	13	GGGGAAGGAAGGGAAGGG	CTTTGACCAGATTCCTGTGTCCAGGACCAGGGGACTAGAAAAACGGGGAAGGAAGGGAAGGG
674	Mature_666	bta-miR-29_40042	chr29	43135733	43135752	-	20	18	0	18	0	0	0	0	Precursor_674	chr29_40042|chr29:43135733-43135775:-	43	18	GGTGGAAGGCTTCGTGGGCT	TCTATGGAGTCGCCACTGGAGCTGGTGGAAGGCTTCGTGGGCT
675	Mature_667	bta-miR-29_39783	chr29	43827890	43827910	+	21	8	8	0	0	0	0	0	Precursor_675	chr29_39783|chr29:43827850-43827910:+	61	8	GGAACTGCCCGGCTGCTCAGG	TGAGCTGTCAGGCGGGCCTCAGCCTGGCTGCCCATTTGCTGGAACTGCCCGGCTGCTCAGG
676	Mature_668	bta-miR-29_40181	chr29	44616657	44616674	-	18	24	24	0	0	0	0	0	Precursor_676	chr29_40181|chr29:44616598-44616674:-	77	24	GAAGAGAAGGGAAGGAGG	GAAGAGAAGGGAAGGAGGCGAGGCTGGGCAGCGGGAGAGTTTTCGCTGCCCTGCAATCTCAAGGGACACCTCTTCCC
677	Mature_669	bta-miR-29_45861	chr29	46459619	46459642	-	24	52	0	0	0	0	52	0	Precursor_677	chr29_45861|chr29:46459581-46459642:-	62	53	CCTCCAGCCTTTAACCCATAGTCC	CCTCCAGCCTTTAACCCATAGTCCAAGAAGAAGAAAAGCGTTCGGGGTCGGGGTGGGGGGTG
678	Mature_670	bta-miR-29_39836	chr29	46533927	46533944	+	18	7	7	0	0	0	0	0	Precursor_678	chr29_39836|chr29:46533927-46533998:+	72	7	CCCAGGGAAGGGGCTTGG	CCCAGGGAAGGGGCTTGGGGCGTGCCTCAGGCTCGGGGGCCTGGGTCCCCCTGGAAGCCCCGTCCTGCAGAT
679	Mature_671	bta-miR-29_45415	chr29	49511271	49511290	+	20	290	0	0	0	0	290	0	Precursor_679	chr29_45415|chr29:49511209-49511290:+	82	292	GGGCCAGCGAGGTGGAGAGG	CCCCTTCTCCTCGCTGGTCCTTGCTCTCCTGGTCTCCTGCAGAAGGAGGTTCTGGGGAGCGCGGGCCAGCGAGGTGGAGAGG
680	Mature_672	bta-miR-29_40090	chr29	50536821	50536845	-	25	14	0	14	0	0	0	0	Precursor_680	chr29_40090|chr29:50536821-50536875:-	55	14	AGGGAAGAGCCCTCCGGGCTGGGCC	CCCCCCGGGTACTGGGCCTCCTCCTCAACCAGGGAAGAGCCCTCCGGGCTGGGCC
681	Mature_673	bta-miR-29_39863	chr29	50845564	50845584	+	21	136	20	94	22	0	0	0	Precursor_681	chr29_39863|chr29:50845520-50845583:+	66	136	GCCGGGAGCCCCGGGGAGAGC	TCCCCCGGGGCTGGGGCAGGACGGAAGAAAGGGCTGGCCTCCTGCGCCGGGAGCCCCGGGGAGAGC
682	Mature_674	bta-miR-29_47274	chr29	51006540	51006558	-	19	8	0	0	8	0	0	0	Precursor_682	chr29_47274|chr29:51006540-51006579:-	40	8	GGCCGACGAGGACTGGGGG	CACCAGCCCCGGAGGCGGGACGGCCGACGAGGACTGGGGG
683	Mature_675	bta-miR-X_40120	chrX	3931232	3931250	+	19	98	0	98	0	0	0	0	Precursor_683	chrX_40120|chrX:3931232-3931281:+	50	98	GAGGGGGCGGGGCGGGGAG	GAGGGGGCGGGGCGGGGAGCGGTGGCGCTTCGGCCTGCCTGGGGTTTCTG
684	Mature_676	bta-miR-X_41163	chrX	4819704	4819721	-	18	277	76	0	201	0	0	0	Precursor_684	chrX_41163|chrX:4819645-4819721:-	77	277	GAAAAAGGAAAGGGAAGG	GAAAAAGGAAAGGGAAGGGGGGTAACGCGGCAATAACGGTTGAGATGCGAGGGCTACTAGTCCCTTTCCTCTACGTT
685	Mature_677	bta-miR-X_48359	chrX	14786322	14786342	-	21	9	0	0	9	0	0	0	Precursor_685	chrX_48359|chrX:14786264-14786342:-	79	14	GAGAATGGAGGGGAGTCAAGC	GAGAATGGAGGGGAGTCAAGCAAGAAAAGAAAGAGAACAAATGCTCCTTCAGGTAAGCTAGGCAATAATCCATGCTCTT
686	Mature_678	bta-miR-X_45956	chrX	17003277	17003300	+	24	62	0	0	0	0	62	0	Precursor_686	chrX_45956|chrX:17003277-17003351:+	75	62	ATCCAGTCTTCTACCACCACCCGC	ATCCAGTCTTCTACCACCACCCGCAGAATAAAAAGAAGAATGAAAGTGGGCCAATGGCGGATGAAGAAACCCCAG
687	Mature_679	bta-miR-X_48490	chrX	30649363	30649381	-	19	14	0	0	14	0	0	0	Precursor_687	chrX_48490|chrX:30649363-30649410:-	48	14	GAAAGAAGGAGGAGGGAAG	TCTGTTGTTCTTCCCCTCAAATGAAAAGGGAAAGAAGGAGGAGGGAAG
688	Mature_680	bta-miR-X_46815	chrX	35121002	35121023	-	22	19	0	0	0	0	19	0	Precursor_688	chrX_46815|chrX:35121002-35121049:-	48	22	TATATATATATATATATATATG	TGTATATATATATATATATATATATATATATATATATATATATATATG
689	Mature_681	bta-miR-X_48563	chrX	37504595	37504612	-	18	16	0	0	16	0	0	0	Precursor_689	chrX_48563|chrX:37504554-37504612:-	59	16	GGGTTCAGTGGCCGGCAG	GGGTTCAGTGGCCGGCAGAGTGGTTGGGGCCCGCTCTTCCGGAGAGCCACTGAACCCGG
690	Mature_682	bta-miR-X_45252	chrX	52284244	52284266	-	23	28	0	0	0	0	0	28	Precursor_690	chrX_45252|chrX:52284244-52284296:-	53	28	CTGCCATTTAACAAAATGAAGAC	TTTTATTTTATTGAGGGTGGGATCCAATACCTGCCATTTAACAAAATGAAGAC
691	Mature_683	bta-miR-X_40556	chrX	54825323	54825341	+	19	40	16	0	24	0	0	0	Precursor_691	chrX_40556|chrX:54825323-54825392:+	70	40	GGGGAAAGGAAGAGGGAAC	GGGGAAAGGAAGAGGGAACATATGGCTATTAAAACACCAAGCTCATGATTCCCTCCCTTTCTGTCTCCTT
692	Mature_684	bta-miR-X_42609	chrX	56187346	56187364	+	19	234	0	0	0	234	0	0	Precursor_692	chrX_42609|chrX:56187293-56187364:+	72	302	CAGGCCATGTGGTGCAGCC	CTGTTGCTGCCAAGGCCCTGGGTTCGATCCCTGGTTGGGGAACTAAGATCTCACAGGCCATGTGGTGCAGCC
693	Mature_685	bta-miR-X_41294	chrX	59946346	59946368	-	23	18	0	18	0	0	0	0	Precursor_693	chrX_41294|chrX:59946346-59946435:-	90	18	TGGTAGCACTTGGGCAAGAAAGT	ATTCTCTGCATGAAGCTGCTGCTGTTGCATCTGTGGCACATAGACCATGGAGTGCCAGAAGCTGTGGTGGTAGCACTTGGGCAAGAAAGT
694	Mature_686	bta-miR-X_47008	chrX	76432685	76432708	-	24	14	0	0	0	0	14	0	Precursor_694	chrX_47008|chrX:76432685-76432746:-	62	14	CTGTAGCTACTGTTTAGTATTCTG	AAGTATCATGTAGCTGCATAGGGAGCTCTGCTTCACGACTGTAGCTACTGTTTAGTATTCTG
695	Mature_687	bta-miR-X_42703	chrX	79706441	79706458	+	18	264	0	0	0	77	87	100	Precursor_695	chrX_42703|chrX:79706387-79706458:+	72	287	ATGCCATGCAGTGGAGCC	CTTCCACTGCAGGCGGCATGGGTTTGATTCCTAGTCAGGGAACTAAGATCCCAAATGCCATGCAGTGGAGCC
696	Mature_688	bta-miR-X_41446	chrX	90932312	90932332	-	21	16	0	16	0	0	0	0	Precursor_696	chrX_41446|chrX:90932312-90932375:-	64	16	CCTCTTAGTACTTATATGCCC	GCAATGAGGACTATGTCTGCAGCCCAGAGGATTATCTGGGGTGCCTCTTAGTACTTATATGCCC
697	Mature_689	bta-miR-X_41714	chrX	90972409	90972426	-	18	8	8	0	0	0	0	0	Precursor_697	chrX_41714|chrX:90972409-90972471:-	63	12	AGGGAGAGAAGGAGAAGG	TGAATCTCTCCTCTGTGTGCTGTCTAGGCTCAGATGGAACTCCACAGGGAGAGAAGGAGAAGG
698	Mature_690	bta-miR-X_41456	chrX	90991562	90991580	-	19	19	0	19	0	0	0	0	Precursor_698	chrX_41456|chrX:90991562-90991626:-	65	19	GATGAGGAGGAGCACACTC	ATGTGCTCCCTTTGTTTTAGATTCCATGAATATCCTGGACCCTGAGGATGAGGAGGAGCACACTC
699	Mature_691	bta-miR-X_47976	chrX	94879188	94879205	+	18	23	0	0	23	0	0	0	Precursor_699	chrX_47976|chrX:94879188-94879249:+	62	23	GGAAAGAGAAAAGAAAGG	GGAAAGAGAAAAGAAAGGGAGTGAGGCTAGTCAGCTTGATCCCTAGATTCTCATTGGGACCT
700	Mature_692	bta-miR-X_41811	chrX	100798629	100798650	-	22	37	37	0	0	0	0	0	Precursor_700	chrX_41811|chrX:100798575-100798650:-	76	37	AGATAGAGGAAGAGAGAGAGAC	AGATAGAGGAAGAGAGAGAGAGACACGAGCATTGGGGTTCTTCTGGTTACCTCTGGTCAATCATCTTGCTTTGTCTGG
701	Mature_693	bta-miR-X_41546	chrX	104665791	104665809	-	19	298	0	298	0	0	0	0	Precursor_701	chrX_41546|chrX:104665791-104665843:-	53	298	GAAATCCGAACGAACTTTT	AAGTTCATTCGGATTCATCTGTAAGATGGTATGGGAAATCCGAACGAACTTTT
702	Mature_694	bta-miR-X_40899	chrX	104695614	104695632	+	19	14	14	0	0	0	0	0	Precursor_702	chrX_40899|chrX:104695579-104695632:+	54	14	AAGAAAAAGGAGGGGGGGA	CCCTTCTCCTCCTGCCCCTCATTACTGTTAGGAAAAAGAAAAAGGAGGGGGGGA
703	Mature_695	bta-miR-X_41551	chrX	108256949	108256967	-	19	27	0	27	0	0	0	0	Precursor_703	chrX_41551|chrX:108256928-108256967:-	40	27	CCTTGGAGGCGTGGGTTCT	CCTTGGAGGCGTGGGTTCTGTATCCATACCTCTCATCCAC
704	Mature_696	bta-miR-X_40716	chrX	112341577	112341594	+	18	146	0	69	0	0	77	0	Precursor_704	chrX_40716|chrX:112341577-112341622:+	46	146	GGGGTGGGGGGGCAAAAT	GGGGTGGGGGGGCAAAATCACATTTAGAACCAAACCCCATACCCGC
705	Mature_697	bta-miR-X_40954	chrX	112891783	112891800	+	18	13	13	0	0	0	0	0	Precursor_705	chrX_40954|chrX:112891783-112891841:+	59	13	AGAGGAGGAAAGAAGAGG	AGAGGAGGAAAGAAGAGGGTAGTGGGGATAGCCTACTTTTCTATCTATCATTTCTCTGA
706	Mature_698	bta-miR-X_48109	chrX	118771543	118771564	+	22	24	0	0	24	0	0	0	Precursor_706	chrX_48109|chrX:118771490-118771564:+	75	24	AGACAAGATGAAGGAGAAGGTC	CTTTCTCCTTTAATTTTTGACCAGGAGAAGAAGTACCGCTTCCAAAACCAGGTAGACAAGATGAAGGAGAAGGTC
707	Mature_699	bta-miR-X_41646	chrX	122509013	122509033	-	21	19	0	19	0	0	0	0	Precursor_707	chrX_41646|chrX:122509013-122509100:-	88	19	AGCTGCAGGGCACAGTCAATT	AGGACCAATGTGCTTTCAGTGGTAGGGACACAGGTTCAATCCCTGGTAGGGGAACTAAGATCCAGCAAGCTGCAGGGCACAGTCAATT
708	Mature_700	bta-miR-X_49292	chrX	132315933	132315950	-	18	41	0	0	41	0	0	0	Precursor_708	chrX_49292|chrX:132315933-132315977:-	45	41	GAAAAAGGGAAGAGGAGG	TCTCTCTCTCCTTCTTCTGGGGTAAGAGAAAAAGGGAAGAGGAGG
709	Mature_701	bta-miR-X_48233	chrX	136311365	136311382	+	18	18	0	0	18	0	0	0	Precursor_709	chrX_48233|chrX:136311318-136311382:+	65	18	AGGAGAGGGGGGGAAAAG	CTTCCTCTTATTTTCTTGGAGACGGGCAGATTAGTGCAAATGAATAAAGGAGAGGGGGGGAAAAG
710	Mature_702	bta-miR-X_41116	chrX	138380987	138381005	+	19	8	8	0	0	0	0	0	Precursor_710	chrX_41116|chrX:138380987-138381055:+	69	8	TGTTACCGACAGAACAAGG	TGTTACCGACAGAACAAGGAGACTGGGAAATATGAAGGTGAGTGTTTGTATCCTTGTGTGTGGAACGGT
